# Computational Study of SmI_2_-Catalyzed Intermolecular Couplings of Cyclopropyl Ketones: Links between the Structure and Reactivity

**DOI:** 10.1021/acs.joc.4c01996

**Published:** 2024-10-22

**Authors:** Song Yu, Ciro Romano, David J. Procter, Nikolas Kaltsoyannis

**Affiliations:** Department of Chemistry, School of Natural Sciences, The University of Manchester, ManchesterM13 9PL, U.K.

## Abstract

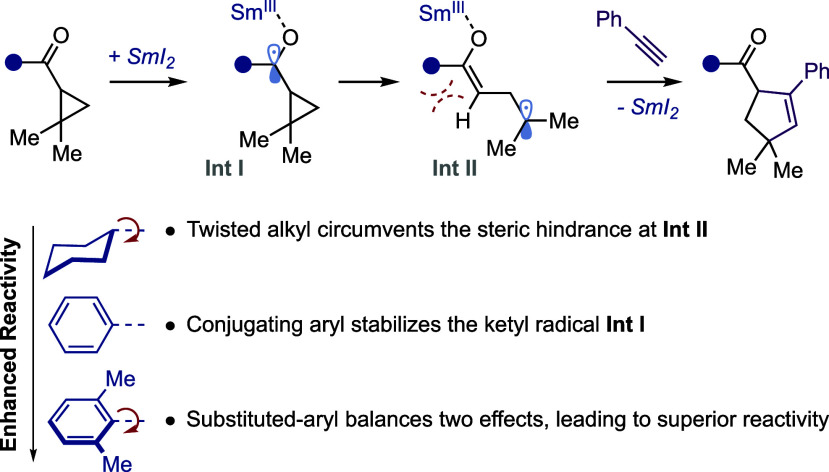

SmI_2_-catalyzed intermolecular coupling reactions of cyclopropyl ketones with alkenes or alkynes offer an efficient strategy for furnishing diverse five-membered ring-containing molecular architectures. This study presents a systematic computational investigation to reveal the structure–reactivity relationships in these reactions. The reactivity of aryl cyclopropyl ketones is enhanced by the stabilized ketyl radical and cyclopropyl fragmentation, arising from the conjugation effect of the aryl ring, despite an obstacle emerging from the *gauche* styrene intermediate that elevates the energy barrier for radical trapping. By contrast, alkyl cyclopropyl ketones lack conjugation and exhibit high barriers for reduction and fragmentation but undergo facile radical trapping due to the minimal steric hindrance. Interestingly, *ortho*-substituted phenyl cyclopropyl ketones exhibit superior reactivity due to a balance between the moderate conjugation, promoting cyclopropyl fragmentation, and the pretwisted nature of the *ortho*-substituted phenyl that circumvents the hindrance posed by the *gauche* intermediate and facilitates the radical trapping. The markedly enhanced reactivity of bicyclo[1.1.0]butyl (BCB) ketones arises from facile fragmentation of the strained BCB motif. Bicyclo[2.1.0]pentyl (BCP) ketones, less strained than BCB ketones, are computationally verified to undergo efficient couplings with various partners, and this can be attributed to their stable fragmentation intermediates that facilitate radical trapping. Our findings provide insights that can aid in designing related reactions.

## Introduction

**Scheme 1:**
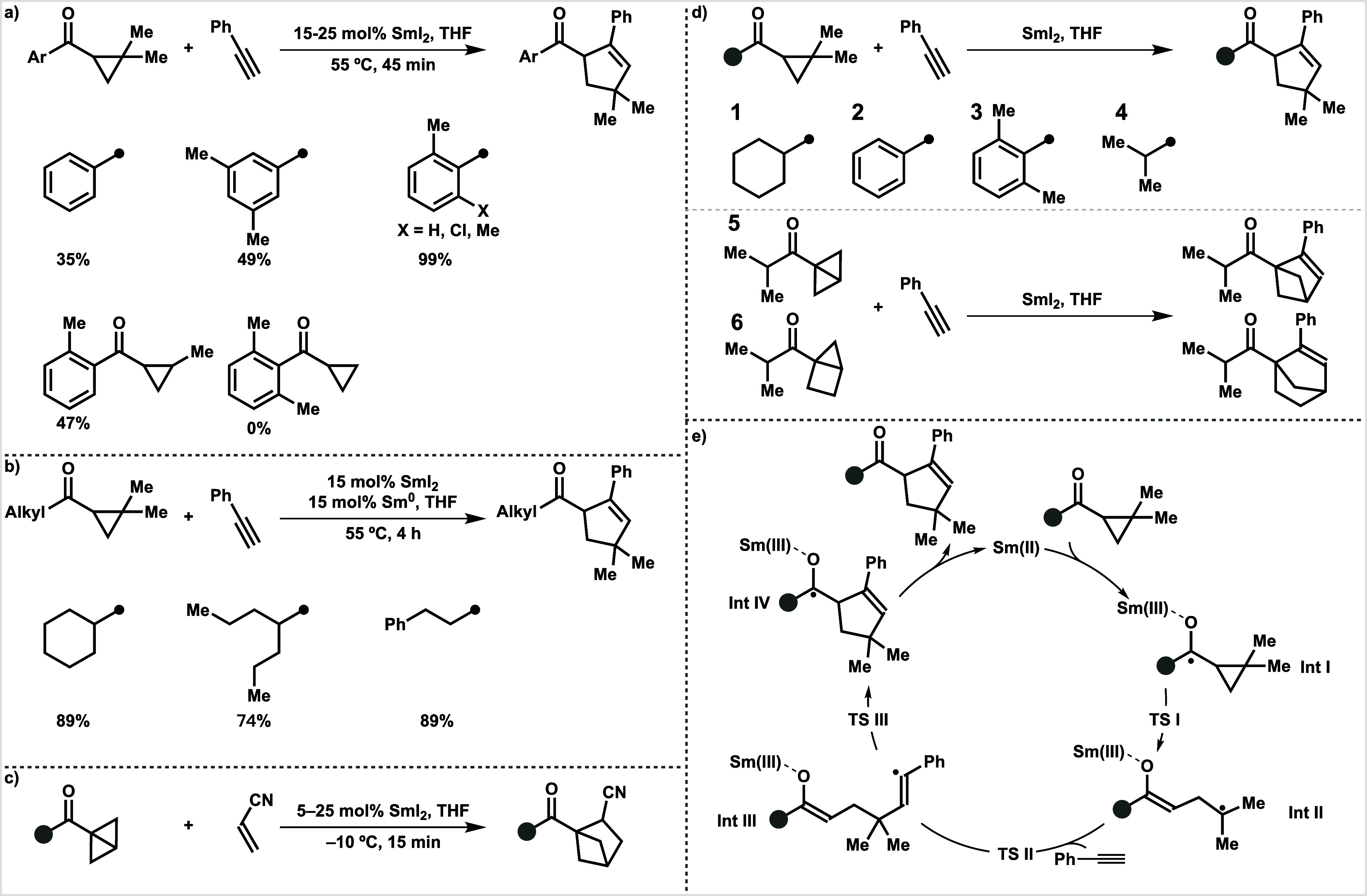
SmI_2_-Catalyzed Intermolecular Coupling Reactions of Ketones: (a) Aryl Cyclopropyl Ketones, (b) Alkyl Cyclopropyl Ketones, and (c) Bicyclo[1.1.0]butyl ketones; (d) This work: Reactions and Substrates Studied; (e) Catalytic Cycle of the SmI_2_-Catalyzed Coupling between Dimethylcyclopropyl Ketones with Phenylacetylene

Samarium(II) diiodide (SmI_2_, Kagan’s reagent) has long held a pivotal role in radical chemistry due to its efficiency as a single electron transfer (SET) reductant.^1^ However, the conventional stoichiometric utilization of SmI_2_ or, in rare cases, the superstoichiometric application of coreductants and additives in conjunction with catalytic SmI_2_ has impeded its widespread application.^2^ Recently, the Procter group reported the SmI_2_-catalyzed radical cyclization of cyclopropyl ketones that proceeds by a radical-relay mechanism, utilizing catalytic SmI_2_ and obviating the need for coreductants.^3^ Significantly, this catalytic methodology has been extended to intermolecular C–C bond formation, facilitating efficient cross-coupling reactions between aryl cyclopropyl ketones and alkyne partners, with SmI_2_ loadings as low as 15 mol %, and thereby affording diverse decorated cyclopentenes (Scheme 1a).^4a^ The proposed catalytic mechanism was supported by combined experimental and computational studies, and an extensive exploration of substrates revealed key structure–reactivity relationships: (i) *ortho*-substituents on the phenyl group are imperative for achieving high yields with aryl cyclopropyl ketones and (ii) the absence of gem-dialkyl substitution on the cyclopropyl group significantly diminishes the reactivity (Scheme 1a). The scope of the SmI_2_-catalyzed coupling reactions has recently been expanded to embrace alkyl cyclopropyl ketones and bicyclic alkyl ketones by optimizing the reaction conditions, including the addition of Sm(0) to prevent SmI_2_ catalyst degradation over longer reaction times (Scheme 1b).^4b^ Moreover, the SmI_2_-catalyzed intermolecular coupling reactions between bicyclo[1.1.0]butyl ketones and alkenes (Scheme 1c) have demonstrated remarkable efficiency under mild conditions, yielding diverse C(*sp*^3^)-rich bicyclic hydrocarbon scaffolds, which serve as notable bioisosteres of benzenoids in medicinal chemistry.^4c^

To support the growing number of experimental investigations of SmI_2_-catalyzed couplings, we have conducted a thorough computational study of such processes, involving a range of cyclopropyl ketones, with the aim of elucidating the relationship between the reactivity and structure and thus attaining a deeper understanding that will facilitate the design of future catalytic processes. We have chosen to focus on the SmI_2_-catalyzed coupling reactions of two categories of cyclopropyl ketone (Scheme 1d): (i) cyclohexyl (**1**), phenyl (**2**), and 2,6-dimethylphenyl (**3**) dimethylcyclopropyl ketones and (ii) dimethylcyclopropyl (**4**), bicyclo[1.1.0]butyl (BCB, **5**), and bicyclo[2.1.0]pentyl (BCP, **6**) *i*-propyl ketones. These substrates were chosen to probe reactivity differences between alkyl and aryl cyclopropyl ketones, the experimentally observed impact of aryl substitutions, and the different reactivities of cyclopropyl and bicycloalkyl ketones. We have identified all stationary points on the potential energy surface (PES) of the catalytic cycle (Scheme 1e) and investigated all possible pathways for each SmI_2_-catalyzed coupling to determine the most plausible mechanism (see Figures S1 and S2 and the corresponding discussions for more details). In the main text, we focus on the optimal pathways to understand the chemical reactivity of these reactions and apply the Energetic Span Model^5^ to gain further insight into the kinetics of catalysis. Energy Decomposition Analysis (EDA)^6^ has been applied to pivotal reactions to reveal the primary factors dictating the electronic interactions between reactants.

## Results and Discussion

### Reactivity Differences between Alkyl and Aryl Cyclopropyl Ketones

**Figure 1:**
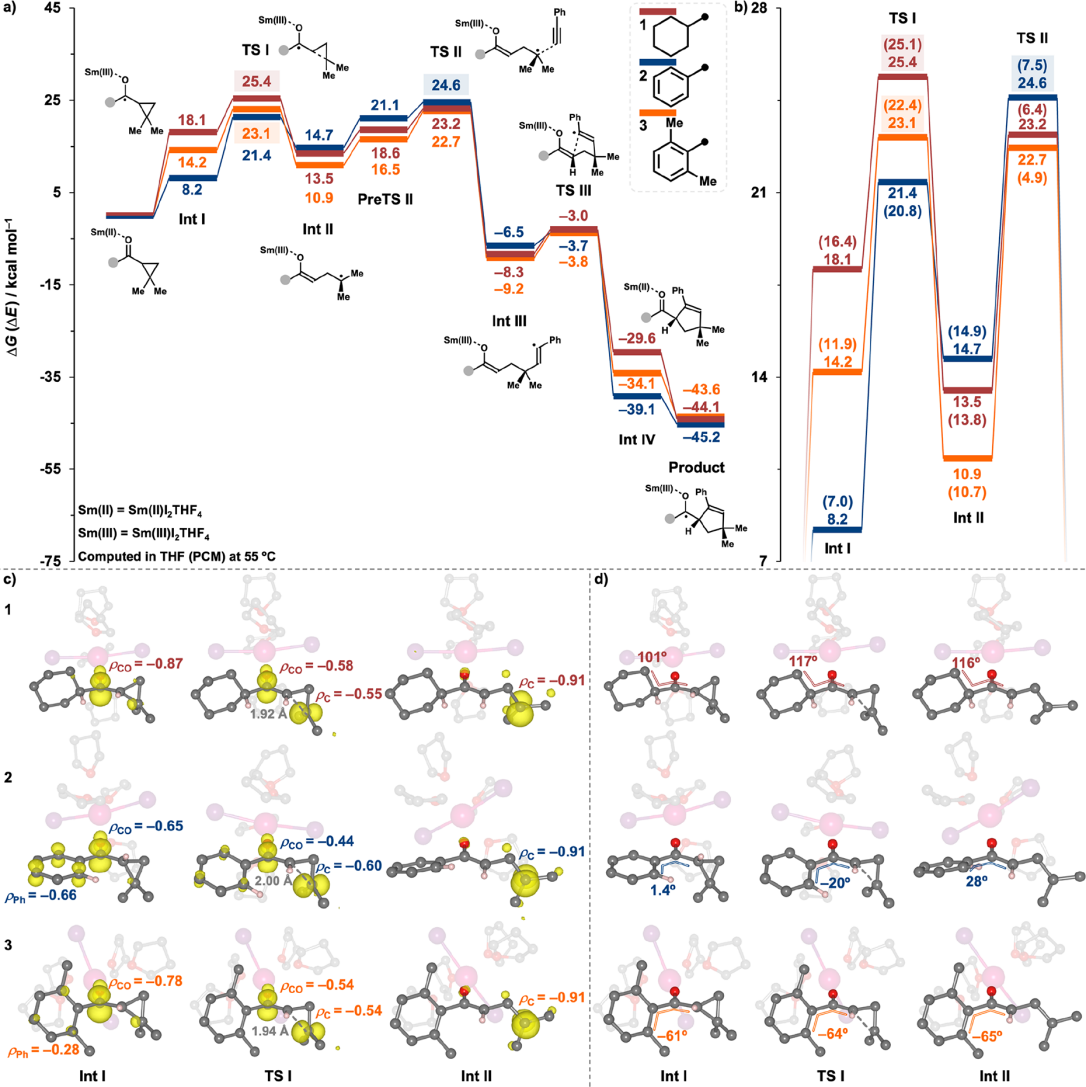
(a) Gibbs energy profiles, Δ*G*, for SmI_2_-catalyzed coupling reactions of cyclohexyl **1** (red), phenyl **2** (blue), and 2,6-dimethylphenyl **3** (orange) cyclopropyl ketones with phenylacetylene. (b) Gibbs energy profiles, Δ*G*, and, in parentheses, electronic (SCF) energies, Δ*E*, of critical reaction steps. (c) Spin density distributions (isovalue = 0.01 e^–^/au^3^) with numerical values (e^–^) of key atoms and (d) dihedral angles of labeled backbones at **Int I**, **TS I**, and **Int II** of SmI_2_-catalyzed couplings of cyclohexyl **1** (top), phenyl **2** (middle), and 2,6-dimethylphenyl **3** (bottom) cyclopropyl ketones with phenylacetylene.

Initially, we studied the PESs of alkyl and aryl cyclopropyl ketones in their SmI_2_-catalyzed coupling reactions with phenylacetylene (Figure 1a). Note that the ketone with dimethyl substitution on the cyclopropyl ring was chosen as the target, rather than the unsubstituted analogue, as both experiments (Scheme 1a) and computations (Figure S4a) have identified the significant beneficial impact of gem-dialkyl substitution of the cyclopropyl on the reaction efficiency. This substitution is crucial for stabilizing the radical intermediate as it effectively disperses the spin density distribution of the radical (Figure S4b). We here discuss reactivity differences based on the traditional approach of focusing on single rate-determining steps, with a view to identifying the key factors dictating reactivity. Later, we also explore the kinetics from the perspective of the entire PES by applying the Energetic Span Model.

The highest stationary point along the PES of cyclohexyl cyclopropyl ketone **1** (in red) is the transition state associated with the cyclopropyl-fragmentation **TS I**, whereas for phenyl cyclopropyl ketone **2** (in blue), the radical-trapping step **TS II** determines the overall reaction barrier. Overall, the activation barrier for phenyl cyclopropyl ketone **2** is determined to be 24.6 kcal mol^–1^, while that for cyclohexyl cyclopropyl ketone **1** is slightly higher at 25.4 kcal mol^–1^. This agrees with general experimental observations indicating the higher efficiency of the SmI_2_-catalyzed coupling reactions of aryl cyclopropyl ketones and relatively slower kinetics observed for alkyl cyclopropyl ketones, the latter often necessitating the addition of Sm(0) to stabilize the SmI_2_ catalyst over the longer reaction times (Scheme 1b). The overall reaction energies for both substrates exhibit pronounced exergonic characteristics, which is in agreement with the smooth progression of both reactions.

Given that the reactivity difference originates from **TS I** and **TS II**, our focus shifted toward scrutinizing the partial PES ranging from **Int I** to **TS II** (Figure 1b). The reaction of phenyl cyclopropyl ketone **2** benefits from substantial stabilization of the intermediate ketyl radical **Int I**, attributed to the ability to disperse the spin density by conjugation between the π-orbital of the aromatic ring and the *p*-orbital of the ketyl radical. Conversely, this effect is absent in cyclohexyl analogue **1**, resulting in a higher energy **Int I**. This contrast is clearly visualized by the spin density distributions in the ketyl radicals **Int I** derived from cyclohexyl and phenyl cyclopropyl ketones, shown in Figure 1c (top and middle, respectively). This conjugation effect is, to some extent, weakened at **TS I**, as the spin density partially migrates to a new radical center, consequently narrowing the energy gap at **TS I** between the cyclohexyl and phenyl cyclopropyl ketones. However, this stabilization is entirely absent at **Int II** as the radical center is remote to the aromatic ring (Figure 1c). Moreover, phenyl cyclopropyl ketone **2** leads to **Int II** that experiences additionally more destabilization than that derived from cyclohexyl cyclopropyl ketone **1**. This is because the ring-opening process of phenyl cyclopropyl ketone **2** leads to the formation of a new conjugated system, the styrene moiety. However, this system is forced into a distorted conformation due to the repulsion between the terminal hydrogens of the butadiene fragment, which partially disrupts the conjugation within the styrene, further increasing the overall energy.^7^ This is supported by the dihedral angle changes shown in Figure 1d: cyclohexyl ketone **1** (top) undergoes moderate structural deformation from **Int I** to **Int II**, whereas phenyl ketone **2** (middle) shifts from a planar geometry at **Int I** to a *gauche* geometry at **TS I**, subsequently inverting at **Int II**. This conformational twist, frequently associated with the formation of 1,3-butadiene and its derivatives,^7^ destabilizes **Int II** and subsequent stationary points, including **TS II**, in the reaction of phenyl ketone **2** (Figure 1b). In summary, the SmI_2_-catalyzed coupling of cyclohexyl cyclopropyl ketone **1** proceeds with high energies at ketyl radical **Int I** and rate-determining **TS I**, whereas the reaction of phenyl cyclopropyl ketone **2** benefits from a conjugation effect, resulting in more stable **Int I** and **TS I** but encountering steric hindrance during the formation of **Int II**, thereby elevating energies of subsequent stationary points and switching the rate-determining step from **TS I** to **TS II**.

We then shifted our attention to 2,6-dimethylphenyl cyclopropyl ketone **3**, a substrate found experimentally to be even more reactive than unsubstituted phenyl cyclopropyl ketone **1** (Scheme 1a). In a previous study,^4a^ the disparity in reactivity between the unsubstituted and substituted phenyl cyclopropyl ketones was rationalized computationally by evaluating the energy changes from the ketyl radical **Int I** to **TS I**; energy changes associated with **Int I** generation and overall barrier energies were not considered. Here, we revisited the SmI_2_-catalyzed coupling of 2,6-dimethylphenyl cyclopropyl ketone **3**, focusing on the PES ranging from **Int I** to **TS II** (orange, Figure 1). Interestingly, this *ortho*-disubstituted phenyl ketone adopts a twisted conformation that diminishes the conjugation between the π-system of the aromatic ring with the radical center in **Int I** and **TS I**; as evidenced by the spin density distributions shown in Figure 1c, the spin density is more localized at the carbon of the ketyl radical. Consequently, **Int I** and **TS I** in the reaction of 2,6-dimethylphenyl cyclopropyl ketone **3** experience less stabilization as compared to those involved in the unsubstituted phenyl cyclopropyl ketone **1** yet remain lower in energy than those associated with cyclohexyl cyclopropyl ketone **2** (Figure 1b). Notably, this twisted aryl moiety of 2,6-dimethylphenyl cyclopropyl ketone **3** circumvents any need to rotate to avoid steric clashes upon the formation of **Int II**, as demonstrated by the nearly unchanged dihedral angles as going from **Int I** to **Int II** (Figure 1d). Consequently, the SmI_2_-catalyzed coupling reaction of 2,6-dimethylphenyl cyclopropyl ketone **3** proceeds with a lower **TS II**, switching the overall reaction barrier back to **TS I**, with a value of 23.1 kcal mol^–1^, the lowest for all cyclopropyl ketone substrates **1**–**3** and consistent with experimental findings (Scheme 1).

To summarize, the *ortho*-disubstituted phenyl cyclopropyl ketone exhibits high reactivity in SmI_2_-catalyzed couplings due to a delicate balance between moderate conjugation of the aryl group with the ketyl radical, stabilized **Int I** and **TS I**, and a pretwisted conformation of the substrate facilitating the formation of **Int II** and a lower energy **TS II**. This interplay between **TS I** and **TS II** upon structural modification of the aryl or alkyl groups of substrates is pivotal for understanding and designing SmI_2_-catalyzed coupling reactions of cyclopropyl ketones. This rationale is further validated by an additional study involving *ortho*-monomethyl-substituted phenyl cyclopropyl ketone, which exhibits a considerable conjugation effect at the ketyl radical **Int I** and **TS I** while undergoing favorable formation of **Int II** and facile subsequent radical-trapping **TS II** (see Figure S3).

The kinetics community has recently questioned the traditional concept of the rate-determining step (RD-step) and proposed the Energetic Span Model for a better understanding of the chemical kinetics of serial catalytic cycles.^5^ This model posits that rate-determining states, instead of a single RD-step, provide a more accurate interpretation of the kinetics in catalytic cycles. The turnover frequency (TOF)-determining transition states (**TDTS**) and TOF-determining intermediates (**TDI**) collectively gauge the apparent activation energy and determine the kinetics and TOF of the catalytic reaction. In addition, the degree of rate control (*X*_rc_), traditionally used in physical organic chemistry to evaluate the effect of various factors, such as substituents and solvents, has been adapted within the Energetic Span Model as a degree of TOF control (*X*_TOF_): a higher *X*_TOF_ value indicates a greater influence of the corresponding state, either the transition state or intermediate, on the overall TOF. Therefore, the **TDI** and **TDTS** can be identified by larger *X*_TOF_ values. It is worth noting that, within this framework, the **TDTS** is not necessarily the highest energy state, and multiple states may affect the kinetics.^5^

The normalized *X*_TOF_ of intermediates (*X*_TOF, I_) and transition states (*X*_TOF, T_) for SmI_2_-catalyzed coupling reactions between cyclohexyl **1**, phenyl **2**, and 2,6-dimethylphenyl **3** cyclopropyl ketones with phenylacetylene are presented in Table 1. The results indicate that the initial **Reactant** functions as **TDI** for all reactions (*X*_TOF, I_ = 1.00). For cyclohexyl cyclopropyl ketone **1**, cyclopropyl-fragmentation **TS I** is the **TDTS** (*X*_TOF, T_ = 0.97); for phenyl cyclopropyl ketone **2**, radical-trapping **TS II** is the **TDTS** (*X*_TOF, T_ = 0.99); and for 2,6-dimethylphenyl cyclopropyl ketone **3**, both **TS I** and **TS II** are important, with **TS I** (*X*_TOF, T_ = 0.65) being more influential than **TS II** (*X*_TOF, T_ = 0.35). These results reinforce and expand upon our findings derived from the RD-steps. Furthermore, while it is technically challenging to accurately compute Gibbs energies and TOFs, the relative TOFs of reactions with similar molecularities can be obtained with minimal loss of accuracy. The relative TOFs show that the SmI_2_-catalyzed coupling reaction of phenyl cyclopropyl ketone **2** is 3.5 times faster than that of the cyclohexyl counterpart **1** and that *ortho*-disubstitution of the aryl ring further promotes the reaction (Table 1). Again, these results are consistent with those in the discussions above.

**Table 1:** Degree of TOF Control (*X*_TOF_) for Intermediates and Transition States of the SmI_2_-Catalyzed Couplings of Cyclohexyl **1**, Phenyl **2**, and 2,6-Dimethylphenyl **3** Cyclopropyl Ketones with Phenylacetylene and Relative Overall TOF

	cyclohexyl **1**		phenyl **2**		2,6-dimethylphenyl **3**	
	*X*_TOF, I_	*X*_TOF, T_	*X*_TOF, I_	*X*_TOF, T_	*X*_TOF, I_	*X*_TOF, T_
Step **Reactant**–**Int I**	**1.00**	0.00	**1.00**	0.00	**1.00**	0.00
Step **Int I**–**TS I**	0.00	**0.97**	0.00	0.01	0.00	**0.65**
Step **Int II**–**PreTS II**	0.00	0.00	0.00	0.00	0.00	0.00
Step **PreTS II–TSII**	0.00	0.03	0.00	**0.99**	0.00	**0.35**
Step **Int III–TS III**	0.00	0.00	0.00	0.00	0.00	0.00
Step **Int IV**–**Product**	0.00	0.00	0.00	0.00	0.00	0.00
TOF (55 °C)	1		3.5		22.8	

### Enhanced Reactivity of Bicycloalkyl Ketones

**Figure 2:**
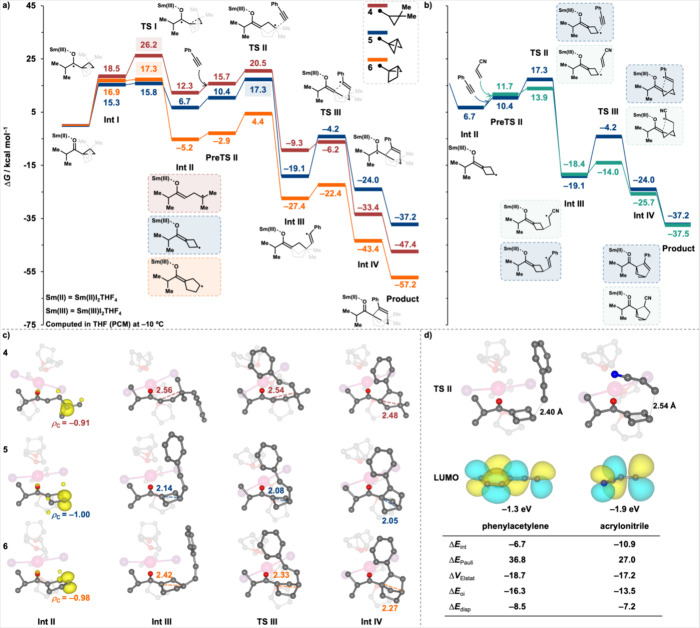
(a) Gibbs energy profiles, Δ*G*, for SmI_2_-catalyzed coupling reactions of *i*-propyl dimethylcyclopropyl ketone **4** (red), *i*-propyl bicyclo[1.1.0]butyl (BCB) ketone **5** (blue), and *i*-propyl bicyclo[2.1.0]pentyl (BCP) ketone **6** (orange) with phenylacetylene. (b) Gibbs energy profiles, Δ*G*, for the radical-trapping and subsequent steps involved in the SmI_2_-catalyzed coupling reactions of *i*-propyl BCB ketone **5** with phenylacetylene and acrylonitrile. (c) Spin density distributions (isovalue = 0.01 e^–^/au^3^) with numerical values (e^–^) of key atoms at **Int II** and key distances (Å) at **Int III**, **TS III**, and **Int IV** for the SmI_2_-catalyzed couplings of *i*-propyl dimethylcyclopropyl ketone **4** (top), *i*-propyl BCB ketone **5** (mid), and *i*-propyl BCP ketone **6** (bottom) with phenylacetylene. (d) Optimized geometries, LUMOs, and energy decomposition analyses for **TS II** associated with SmI_2_-catalyzed coupling reactions of *i*-propyl BCB ketone **5** with phenylacetylene and with acrylonitrile.

Next, we moved to understand the reactivity differences between alkyl cyclopropyl and bicyclobutyl ketones. Figure 2a depicts the full PESs for SmI_2_-catalyzed couplings of *i*-propyl dimethylcyclopropyl ketone **4** (in red) and *i*-propyl bicyclo[1.1.0]butyl (BCB) ketone **5** (in blue) with phenylacetylene. Figure 2a illustrates that *i*-propyl BCB ketone **5** exhibits a significantly lower energy **TS I** (15.8 kcal mol^–1^), which can be attributed to the high strain within the bicycloalkyl motif facilitating the ring-opening process. However, **Int II** of *i*-propyl BCB ketone **5** is marginally lower in energy than that of *i*-propyl dimethylcyclopropyl ketone **4**. This arises because the newly formed four-membered ring in **Int II** of *i*-propyl BCB ketone **5** still retains some strain, i.e., the transition from **Int I** to **Int II** does not relieve all ring strain. Consequently, radical-trapping **TS II** emerges as the crucial determinant of the reaction kinetics for *i*-propyl BCB ketone **5**. The overall barrier energy for the SmI_2_-catalyzed coupling reaction of *i*-propyl BCB ketone **5** with phenylacetylene, calculated at 17.3 kcal mol^–1^, is significantly lower than that calculated for dimethylcyclopropyl ketone **4** at 26.2 kcal mol^–1^. Furthermore, the reaction involving *i*-propyl BCB ketone **5** must surmount high energy **TS III** leading to the formation of a less stable **Int IV** and final product than in the case of dimethylcyclopropyl ketone **4**. This is because the ring-closing reaction of *i*-propyl BCB ketone **5** involves the formation of the bicyclo[2.1.1]pentene, necessitating compression of the already strained **Int III** (see structural information relating to **Int III**, **TS III**, and **Int IV** in the middle row of Figure 2c). Similarly, analyses of these reactions utilizing the Energetic Span Model (Table 2) yielded consistent conclusions: (i) the kinetics of the SmI_2_-catalyzed coupling reaction of *i*-propyl BCB ketone **5** with phenylacetylene is dominated by the radical-trapping **TS II** and (ii) the overall TOF of *i*-propyl BCB ketone **5** exceeds that of *i*-propyl dimethylcyclopropyl ketone **4** by 8 orders of magnitude at a temperature of −10 °C.

**Table 2:** Degree of TOF Control (*X*_TOF_) for Intermediates and Transition States of the SmI_2_-Catalyzed Coupling Reactions of *i*-Propyl Dimethylcyclopropyl Ketone **4**, *i*-Propyl Bicyclo[1.1.0]butyl (BCB) Ketone **5**, and *i*-Propyl Bicyclo[2.1.0]pentyl (BCP) Ketone **6** with Phenylacetylene or Acrylonitrile^a^

	cyclopropyl **4** + phenylacetylene		BCB **5** + phenylacetylene		BCB **5** + acrylonitrile		BCP **6** + phenylacetylene		BCP **6** + acrylonitrile	
	*X*_TOF, I_	*X*_TOF, T_	*X*_TOF, I_	*X*_TOF, T_	*X*_TOF, I_	*X*_TOF, T_	*X*_TOF, I_	*X*_TOF, T_	*X*_TOF, I_	*X*_TOF, T_
Step **Reactant**–**Int I**	**1.00**	0.00	**0.99**	0.02	**1.00**	**0.27**	**1.00**	**0.32**	**1.00**	**0.32**
Step **Int I**–**TS I**	0.00	**1.00**	0.00	0.05	0.00	**0.71**	0.00	**0.68**	0.00	**0.68**
Step **Int II**–**PreTS II**	0.00	0.00	0.00	0.00	0.00	0.00	0.00	0.00	0.00	0.00
Step **PreTS II–TSII**	0.00	0.00	0.00	**0.92**	0.00	0.02	0.00	0.00	0.00	0.00
Step **Int III–TS III**	0.00	0.00	0.01	0.01	0.00	0.00	0.00	0.00	0.00	0.00
Step **Int IV**–**Product**	0.00	0.00	0.00	0.00	0.00	0.00	0.00	0.00	0.00	0.00
TOF (−10 °C)	4.4 × 10^–8^		1		13.6		0.7		0.7	

a Relative overall TOF computed at −10 °C.

Given the pivotal role of the radical-trapping process in determining the kinetics of BCB ketone couplings, the selection of an appropriate coupling partner is essential for ensuring the reaction efficiency. Hence, we have assessed the SmI_2_-catalyzed coupling of *i*-propyl BCB ketone **5** with a different coupling partner, acrylonitrile, the substrate used in actual experiments (Scheme 1c).^4c^ The PESs in Figure 2b illustrate that acrylonitrile exhibits a more favorable coupling with the radical intermediate **Int II** compared to phenylacetylene. Computed overall TOFs (Table 2) corroborate this finding, indicating that the SmI_2_-catalyzed coupling reaction of *i*-propyl BCB ketone **5** with acrylonitrile is 13.6 times faster than that with phenylacetylene at −10 °C. This significant difference highlights the crucial role of the coupling partner in determining the reaction efficiency. The stabilized **TS II** associated with acrylonitrile arises from an enhanced electronic interaction between **Int II** and acrylonitrile, resulting in an interaction energy of −10.9 kcal mol^–1^, more stable than that computed for phenylacetylene at −6.7 kcal mol^–1^ (Figure 2d). Energy decomposition analyses^6^ of the interaction energy at **TS II** further reveals that the more favored interaction between the enolate radical and acrylonitrile is facilitated by reduced Pauli repulsion (Figure 2d). This finding resembles prior studies on acid-catalyzed Diels–Alder reactions, where the complexation of an acid to the dienophile promotes the reaction by reducing Pauli repulsion between the π-systems of reactants, rather than through enhanced donor–acceptor interactions.^8^ Similarly, the CN group in acrylonitrile can deplete electron density from the double bond, thereby diminishing Pauli repulsion toward another reactant. Overall, selection of suitable unsaturated partners for the coupling step is crucial for achieving efficient SmI_2_-catalyzed reactions of BCB ketones at low temperatures, given the fragmentation step of BCB ketones is already highly favored. Interestingly, previous experiments^4c^ have found that the SmI_2_-catalyzed couplings of BCB ketones proceeded smoothly with acrylonitrile and various acrylates but were much less effective with phenylacetylene. This observation aligns with and further supports our computational conclusions.

We next evaluated the reactivity of *i*-propyl bicyclo[2.1.0]pentyl (BCP) ketone **6** in the SmI_2_-catalyzed coupling with phenylacetylene (in orange, Figure 2a). The bicyclo[2.1.0]pentyl scaffold has been confirmed to possess less ring strain, with a total strain energy of 54.7 kcal mol^–1^, compared to the bicyclo[1.1.0]butyl motif (63.9 kcal mol^–1^); crucially, once the three-membered ring undergoes ring-opening, a five-membered ring cyclopentyl radical possesses a near-negligible strain compared to the analogous four-membered ring cyclobutyl radical, which retains strain.^9^ This difference accounts for the substantial energy release in transition **Int I** to **Int II** for *i*-propyl BCP ketone **6**, which is absent for *i*-propyl BCB ketone **5** (Figure 2a). Subsequently, the SmI_2_-catalyzed coupling reaction of *i*-propyl BCP ketone **6** proceeds with a facile radical-trapping **TS II**, making the previous fragmentation **TS I** the only rate-determining state, with an overall activation energy of 17.3 kcal mol^–1^. Thus, the choice of coupling partner is predicted to have no influence on the reaction rate (see Figure S5 and Table 2 for an assessment of the coupling of *i*-propyl BCP ketone **6** with acrylonitrile). Additionally, the reaction of *i*-propyl BCP ketone **6** leads to a near-strainless **Int III**, thus facilitating the ring-closing **TS III** and yielding a much more stable product than in the case for *i*-propyl BCB ketone **5** (Figure 2a).

In summary, our computational results indicate that alkyl bicyclo[2.1.0]pentyl (BCP) ketones possess lower overall strain than alkyl bicyclo[1.1.0]butyl (BCB) ketones, yet they are predicted to undergo efficient SmI_2_-catalyzed coupling reactions. Furthermore, the heightened stability of these less strained substrates may allow for elevated reaction temperatures to be employed, while the facile radical-trapping reaction enhances compatibility with various coupling partners. Although this has not yet undergone experimental scrutiny, we predict that these substrates may hold promise for enriching the scope of SmI_2_-catalyzed coupling reactions.

## Conclusions

We have conducted a systematic computational study of SmI_2_-catalyzed intermolecular couplings of cyclopropyl ketones, revealing fundamental relationships between the structure and reactivity of these systems. The reactivity of phenyl cyclopropyl ketones is enhanced by a stabilization of the ketyl radical and promotion of the subsequent cyclopropyl-fragmentation step, through a conjugation between the aromatic ring and radical center. However, an obstacle emerges during the formation of a *gauche* styrene intermediate, resulting in an increased energy for the subsequent radical-trapping step. By contrast, alkyl ketones lack the stabilization of the ketyl radical and therefore exhibit a high energy barrier for reduction and cyclopropyl fragmentation but, however, undergo a facile radical trapping due to the absence of steric hindrance of the pretwisted alkyl. Interestingly, *ortho*-substituted phenyl cyclopropyl ketones exhibit superior reactivity in SmI_2_-catalyzed coupling reactions, due to the balance between (i) the moderate conjugation of the substituted phenyl ring with the ketyl radical and (ii) the pretwisted conformation of the same ring, favoring formation of the required *gauche* styrene intermediate prior to the radical-trapping event. Our analyses rationalize previously observed experimental phenomena and establish the links between the structure and reactivity of aryl and alkyl cyclopropyl ketones.

We also compared the reactivity of cycloalkyl and bicycloalkyl ketones in the SmI_2_-catalyzed intermolecular couplings. Bicyclo[1.1.0]butyl (BCB) ketones exhibit extremely high reactivity compared to cyclopropyl ketones, attributed to the facile fragmentation of the highly strained BCB scaffold. Interestingly, despite being less strained than BCB ketones, bicyclo[2.1.0]pentyl (BCP) ketones also exhibit great potential for undergoing efficient SmI_2_-catalyzed coupling reactions with various coupling partners owing to the reduced energy of the radical-trapping step. While the experimental validation of the SmI_2_-catalyzed coupling reactions of BCP ketones remains unexplored, we predict that these reactive compounds may well enrich the scope of SmI_2_-catalyzed coupling reactions.

In summary, our study has revealed the critical relationships between the structure and reactivity in SmI_2_-catalyzed intermolecular coupling reactions of cyclopropyl ketones and identified the pivotal factors dictating the reactivity. These findings offer valuable insights into the understanding of this type of reaction and provide design principles for future advancement.

## Computational Details

Geometry optimizations were performed by using Gaussian 16, Revision C.01,^10^ employing the PBE0 functional^11^ and Dunning’s Correlation-Consistent Double-Zeta + polarization (cc-pVDZ)^12^ basis sets for C, H, and O. For Sm and I, Stuttgart-Köln Effective Core Potentials (ECPs) and associated valence basis sets were used.^13^ The dispersion corrections from Grimme’s D3 model^14^ with Becke-Johnson damping factors^15^ were incorporated during geometry optimizations. Harmonic vibrational frequency calculations were performed at 328.15 K (55 °C) for ketones **1**–**3** and at 263.15 K (−10 °C) for ketones **4**–**6**, the temperatures used in experiments,^4^ using the same level of theory to confirm the nature of stationary points as either true minima or transition states and to provide thermodynamic corrections.

Subsequent single-point calculations were carried out on the optimized geometries, including the Douglas-Kroll-Hess second order scalar relativistic Hamiltonian,^16^ and utilizing the SARC basis set for Sm, Jorge for I,^17^ and Dunning’s Correlation-Consistent Triple-Zeta + polarization (cc-pVTZ)^12^ for the remaining elements. These calculations were performed with the inclusion of solvent effects using PCM^18^ in THF. This methodology has been proven to produce reliable results.^4b^ The energies obtained from single-point calculations with solvent effects and the enthalpic corrections derived from frequency calculations collectively yielded the solvation enthalpies, *H*_sol_. The entropic contributions were determined from frequency calculations and adjusted by using the quasi-harmonic approximation proposed by Grimme.^19^ Specifically, the −*TS* terms were corrected directly using GoodVibes,^20^ with a cutoff frequency set to 100 cm^–1^. Previous calculations on systems of this type suggest that reactivity takes place via the quintet spin state, with antiparallel coupling of the ligand radical anion to the Sm(III) 4f^5^ metal center. Hence, the calculations were performed with a spin multiplicity of 7 for the initial reactants and final products and 5 for all intermediates and transition states.

Energetic Span Model analyses^5^ have been conducted to understand the chemical kinetics of serial catalytic cycles, utilizing the AUTOF program developed by Kozuch and Shaik.^21^ Energy decomposition analyses (EDA)^6^ were performed on key structures using AMS2023.1,^22^ employing the PBE0 functional and TZ2P basis sets^23^ and incorporating the zeroth order approximation (ZORA) for scalar relativistic effects.^24^ Note that these single-point calculations were performed based on geometries obtained from Gaussian 16. EDA serves as a valuable tool to explore the nature of electronic interactions between fragments in a chemical system. It decomposes the interaction energy between fragments into four physically meaningful terms (eq 1).
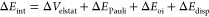
1

The electrostatic term Δ*V*_elstat_ corresponds to the classical electrostatic interaction between the unperturbed charge distributions of the interacting fragments. The Pauli term Δ*E*_Pauli_ comprises the Pauli-repulsive orbital interactions between closed-shell orbitals. Δ*E*_oi_ represents the orbital interactions, such as charge transfer, namely, the interactions between the occupied orbitals of one fragment and the unoccupied orbitals of the other, and polarization, that is, the occupied–unoccupied orbital mixing within one fragment due to the presence of the other. Additionally, Δ*E*_disp_ is added to account for the dispersion interactions.

## Data Availability

The data underlying this study are available in the published article and its Supporting Information.

## Supporting Information Available

Additional figures, analyses, and *xyz* coordinates and energies of all stationary points coordinates (PDF)
