# Supplementary material for: Computational Study of SmI2-Catalyzed Intermolecular Couplings of Cyclopropyl Ketones: Links between the Structure and Reactivity
Source: J Org Chem. 2024 Oct 22;89(21):15842–50. doi: 10.1021/acs.joc.4c01996 (PMC11536359; doi:10.1021/acs.joc.4c01996)
Supplement: Supplementary file 1 — jo4c01996_si_001.pdf [file jo4c01996_si_001.pdf]

Supporting Information

Computational Study of SmI_2-Catalyzed Intermolecular Couplings of Cyclopropyl Ketones: Links between Structure and Reactivity

Song Yu, Ciro Romano, David J. Procter and Nikolas Kaltsoyannis*

Department of Chemistry, School of Natural Sciences, The University of Manchester, Manchester, M13 9PL, UK

*Nikolas Kaltsoyannis: nikolas.kaltsoyannis@manchester.ac.uk

Contents

Page

1. **Multiple Reaction Pathways** S3

Figure S1. Gibbs energy profiles for two ring-closing pathways of the SmI_2-catalyzed coupling reactions of cyclohexyl, phenyl and 2,6-dimethylphenyl cyclopropyl ketones with phenylacetylene.

Figure S2. Gibbs energy profiles for two radical-trappings and subsequent processes associated with the SmI_2-catalyzed coupling reactions of *i*-propyl bicyclo[1.1.0]butyl and bicyclo[2.1.0]pentyl ketones with phenylacetylene.

2. ***Ortho*-Monosubstituted Phenyl Cyclopropyl Ketone** S5

Figure S3. Gibbs energy profiles and electronic (SCF) energies of critical reaction steps of SmI_2-catalyzed coupling reactions of cyclohexyl, phenyl, 2,6-dimethylphenyl and 2-methylphenyl cyclopropyl ketones with phenylacetylene.

3. **Influence of *Gem*-Dimethyl Substitution on Cyclopropyl Moiety** S6

Figure S4. Gibbs energy profiles for SmI_2-catalyzed couplings of 2,6-dimethylphenyl cyclopropyl and dimethylcyclopropyl ketones with phenylacetylene.

4. **SmI_2-Catalyzed Intermolecular Couplings of Bicycloalkyl Ketones with Acrylonitrile** S7

Figure S5. Gibbs energy profiles for SmI_2-catalyzed coupling reactions of *i*-propyl dimethylcyclopropyl, bicyclo[1.1.0]butyl and bicyclo[2.1.0]pentyl ketones with phenylacetylene. Comparison of Gibbs energy profiles for radical-trapping and subsequent reactions in the SmI_2-catalyzed couplings of *i*-propyl bicyclo[1.1.0]butyl ketone with phenylacetylene and acrylonitrile.

5. **Coordinates and Energies** S8

Table S1. Cartesian coordinates of optimized geometries and energies

1. Multiple Reaction Pathways

In this work, we have investigated all possible reaction pathways for each SmI_2-catalyzed coupling reaction to identify the most plausible mechanism. These findings have been then utilized to discuss the chemical reactivity and kinetics in the main text. The divergent pathways associated with the SmI_2-catalyzed couplings of aryl or alkyl cyclopropyl ketones with phenylacetylene originate from their ring-closing reactions. Figure S1a and 1b demonstrates that cyclohexyl **1** (red) and phenyl **2** (blue) ketones undergo two different ring-closing transition states **TS III** with a Gibb energy gap of 1.1–1.5 kcal/mol. By contrast, the 2,6-dimethylphenyl **3** (orange) counterpart experiences two different **TS III** with a more pronounced Gibbs energy gap. This difference can be attributed to the orientation of the cyclohexyl and phenyl moieties, which are positioned either perpendicular or parallel to the plane of the carbonyl group, resulting in nearly identical steric hindrance for two distinct **TS III**. Conversely, the 2,6-dimethylphenyl ketone features a twisted phenyl moiety, which induces significant steric hindrance for one of the two ring-closing transition states, **TS III'** (Figure S1c).

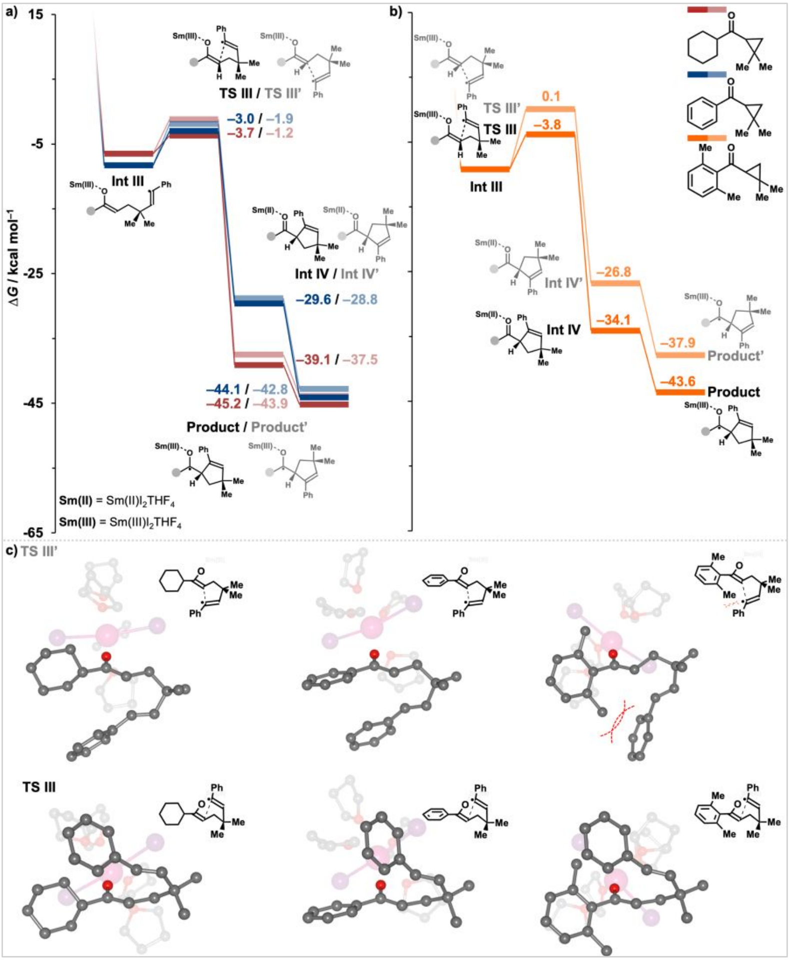

Figure S1. Gibbs energy profiles for two ring-closing pathways of the SmI_2-catalyzed coupling reactions of a) cyclohexyl cyclopropyl ketone (red), b) phenyl cyclopropyl ketone (blue) and b) 2,6-dimethylphenyl cyclopropyl ketone(orange) with phenylacetylene. c) Schematic diagrams with optimized geometries for two **TS III**.

On the other hand, the differing reaction pathways involved in the SmI_2-catalyzed couplings of bicycloalkyl ketones is attributed to the radical-trapping rather than the ring-closing. Figure S2 illustrates that the interaction of the radical intermediate **Int II** with phenylacetylene proceeds via two distinct pathways. Using *i*-propyl bicyclo[1.1.0]butyl ketone as an example: the bottom-side attack of phenylacetylene on the substrate (**TS II'**) encounters additional steric impediments due to the presence of SmI_2 additives. This interaction results in a tilted orientation of phenylacetylene and thus an increase in system energy of **TS II'**, as compared to **TS II** (Figure S2b).

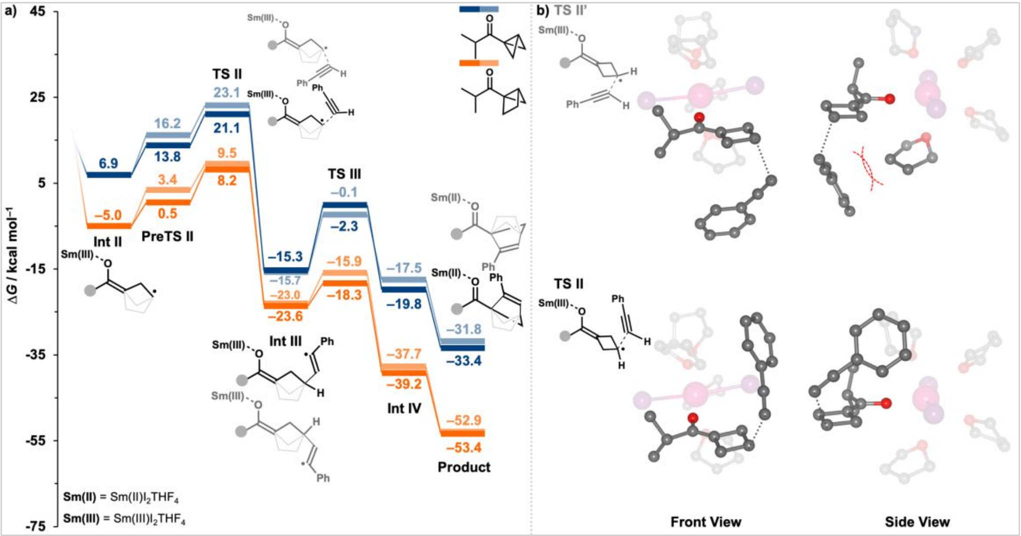

Figure S2. a) Gibbs energy profiles for two radical-trappings and subsequent processes associated with the SmI_2-catalyzed coupling reactions of *i*-propyl bicyclo[1.1.0]butyl (blue) and bicyclo[2.1.0]pentyl (orange) ketones with phenylacetylene. b) Front and side views of two different **TS II** associated with the SmI_2-catalyzed couplings of *i*-propyl bicyclo[1.1.0]butyl ketone with phenylacetylene.

2. *Ortho*-Monosubstituted Phenyl Cyclopropyl Ketone

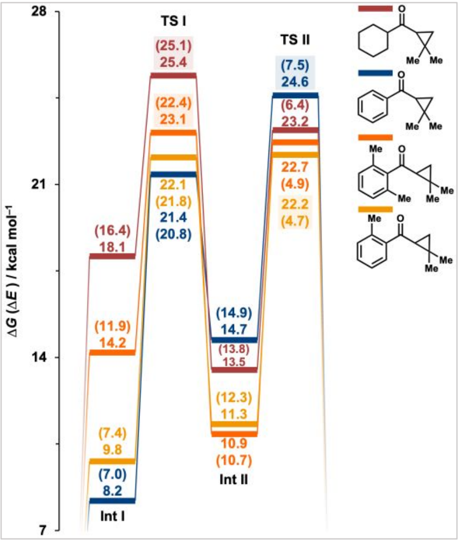

Figure S3. Gibbs energy profiles, ΔG, and, in parenthesis, electronic (SCF) energies, ΔE, of critical reaction steps of SmI_2-catalyzed coupling reactions of cyclohexyl (red), phenyl (blue), 2,6-dimethylphenyl (orange) and 2-methylphenyl (yellow) dimethylcyclopropyl ketones with phenylacetylene. All were computed at 328.15 K in THF using PCM.

3. Influence of Gem-Dimethyl Substitution on Cyclopropyl Moiety

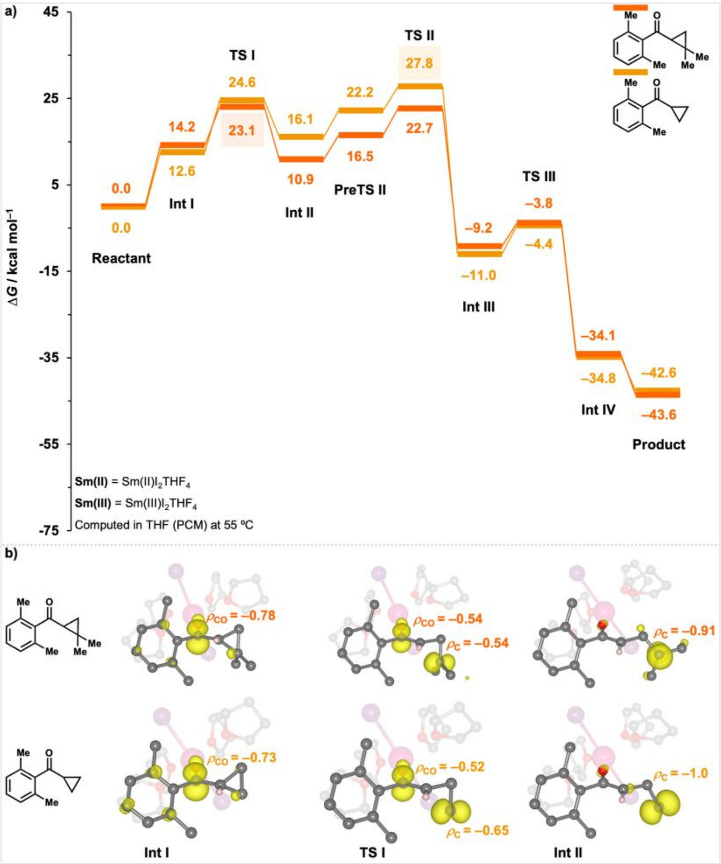

Figure S4. a) Gibbs energy profiles, ΔG for SmI_2-catalyzed coupling reactions of 2,6-dimethylphenyl dimethylcyclopropyl (orange) and cyclopropyl (yellow) ketones with phenylacetylene. b) Spin distributions (isovalue = 0.01 e^{-}/au^{3}) with numerical values (e^{-}) of key atoms at **Int I**, **TS I** and **Int II** of the SmI_2-catalyzed coupling reactions of 2,6-dimethylphenyl dimethylcyclopropyl (top) and cyclopropyl (bottom) ketones with phenylacetylene.

4. SmI_2-Catalyzed Intermolecular Couplings of Bicycloalkyl Ketones with Acrylonitrile

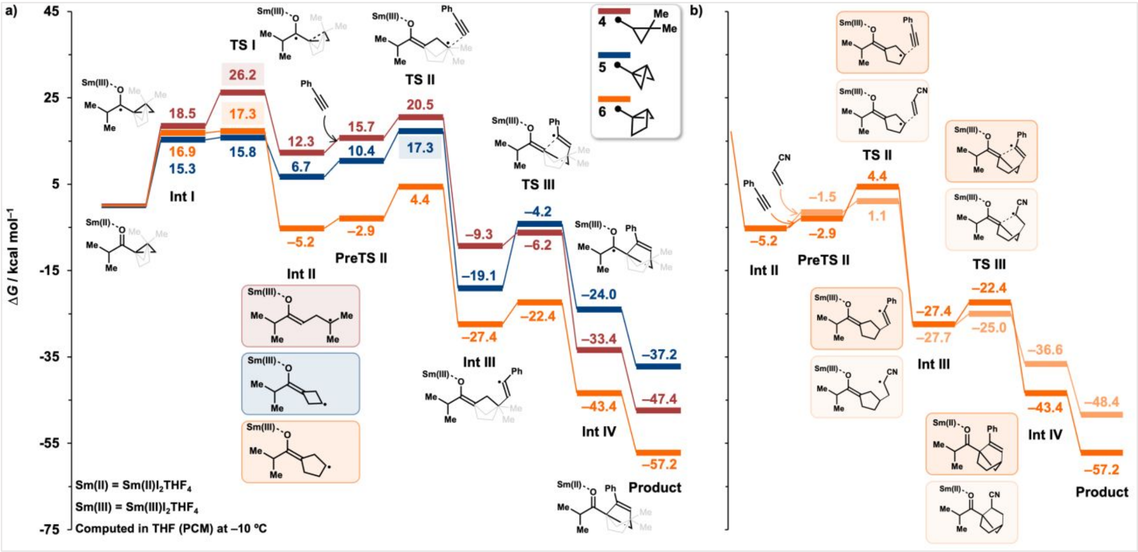

Figure S5. a) Gibbs energy profiles, ΔG, for SmI_2-catalyzed coupling reactions of *i*-propyl dimethylcyclopropyl ketone **4** (red), *i*-propyl bicyclo[1.1.0]butyl ketone **5** (blue) and *i*-propyl bicyclo[2.1.0]pentyl ketone **6** (orange) with phenylacetylene. b) Gibbs energy profiles, ΔG, for radical-trapping and subsequent reactions of the SmI_2-catalyzed coupling reactions of *i*-propyl bicyclo[1.1.0]butyl ketone **6** with phenylacetylene and acrylonitrile.

5. Coordinates and Energies

Table S1. Cartesian coordinates (in Å) of geometries optimized at PBE0 functional with cc-pVDZ basis sets for C, H and O, and ECPs for Sm and I, single point energies computed in vacuum E_{vacuum} and THF (PCM) E_{sol}, $-TS$ terms corrected by Grimme's method, and Gibbs energies G_{sol} (in Hartree).

Cyclohexyl Dimethylcyclopropyl Ketone Reactant

E_{vacuum} = -26109.9349
E_{sol} = -26109.9579
$-TS$ = -0.1460
G_{sol} = -26109.2730

H	-4.65530300	1.58807700	-1.40017200
H	-3.15284000	3.16666300	-2.66059900
H	-1.69145400	2.57344100	-1.68719700
H	-5.00653000	-4.07233700	1.52160100
H	-1.71138000	2.67299500	0.79766400
H	-5.28402800	3.92847400	0.34872900
C	-3.05474900	0.59318700	-0.33504300
C	-3.64655500	1.75097900	-1.01555900
C	-2.76825800	2.76404300	-1.72126200
C	-3.41607000	3.17901400	-0.45138500
C	-4.62210200	4.08295200	-0.51841600
O	-1.84302400	0.51562700	-0.13075300
Sm	0.59922400	-0.00743000	-0.06587500
O	-0.15383800	-0.83390700	-2.41344300
C	-0.57306100	-0.05298600	-3.52896500
C	-1.84899200	-0.73684600	-3.98713800
C	-1.55237800	-2.22428000	-3.72819300
C	-0.34769000	-2.20334100	-2.77203900
I	1.39148500	2.79700800	-1.43280100
I	-0.03806000	-2.85661200	1.31736100
O	2.75665800	0.12733000	1.39581000
C	3.96106300	0.79757900	0.99182100
C	4.81795300	0.87034900	2.24358800
C	4.41711300	-0.41248400	2.96711400
C	2.92456400	-0.46636300	2.69169700
O	0.09787300	1.31675700	2.11728600
C	0.77131200	2.52646800	2.50478600
C	0.05495200	3.00201600	3.75671100
C	-0.37833100	1.68168200	4.38826100
C	-0.78152900	0.87878200	3.16459300
O	2.59079400	-1.16175200	-1.28225600
C	3.08768600	-0.75964800	-2.56764900
C	4.39349400	-1.51363300	-2.74373000
C	4.09458900	-2.81647700	-2.00707000
C	3.29947700	-2.31913100	-0.81228900
H	4.44783200	0.19832600	0.20236600
H	3.68357200	1.77086400	0.56185100
H	2.35806700	0.13095700	3.42881100
H	2.49502500	-1.47819400	2.66336900
H	4.54446200	1.75176300	2.84657000
H	5.89096200	0.93462100	2.01574200
H	4.64731500	-0.40669500	4.04154000
H	4.92226700	-1.28162900	2.51491700
H	1.82893100	2.28463400	2.70816500
H	0.73610700	3.22431200	1.65622100
H	-1.81995400	1.09865400	2.86508800
H	-0.67035800	-0.21061700	3.27016500
H	-0.82764000	3.60460400	3.48812000
H	0.70094200	3.61161800	4.40360300
H	-1.19856000	1.78439900	5.11224000
H	0.47184800	1.20285700	4.90118900
H	2.35010400	-1.04807200	-3.33696900
H	3.17938000	0.33619400	-2.57078900
H	3.96385400	-2.01085000	0.01466200
H	2.56498800	-3.03404500	-0.41470000
H	5.21882500	-0.97416700	-2.25068200
H	4.66077600	-1.65212200	-3.80054400
H	4.99288600	-3.37594900	-1.71130100
H	3.47267500	-3.47604300	-2.63404400
H	-0.68156500	0.98461700	-3.18961200
H	0.20881200	-0.08176900	-4.31220700
H	0.56593100	-2.57667000	-3.26997600
H	-0.49083500	-2.76715700	-1.83931800
H	-2.09065900	-0.52088000	-5.03708900
H	-2.69043500	-0.39216600	-3.36726000
H	-1.29948600	-2.75243800	-4.65874300
H	-2.41446000	-2.73879600	-3.28236900
H	-2.15452800	4.39059300	0.78124000
H	-4.30270200	5.13769300	-0.51297500
H	-3.15562800	3.23251900	1.69422000
H	-5.20959100	3.91457400	-1.43308600
C	-2.56050800	3.36665400	0.77563600
C	-3.96972200	-0.51580300	0.12621100
C	-4.60323800	-2.00889300	2.05942600
C	-3.73573900	-0.84139900	1.60517100
C	-3.72785000	-1.76421600	-0.73769900
C	-4.58780600	-2.93465300	-0.27623200
C	-4.35701500	-3.24296500	1.19869100
H	-5.01527400	-0.18873100	-0.01255000
H	-5.67062100	-1.72344400	2.00368100
H	-4.39328600	-2.23327900	3.11729900
H	-2.67295100	-1.10305700	1.73440000
H	-3.93686900	0.05268800	2.21907500
H	-2.66104500	-2.03191800	-0.65146700
H	-3.93000200	-1.53235100	-1.79661300
H	-4.36473100	-3.82003700	-0.89337700
H	-5.65453300	-2.69528300	-0.44403100
H	-3.31300900	-3.57371700	1.33836400

Int I

E_{vacuum} = -26109.9185
E_{sol} = -26109.9318
$-TS$ = -0.1417
G_{sol} = -26109.2441

H	-4.53219100	1.29383500	-1.54701800
H	-3.10422800	2.53558800	-3.17527400
H	-1.62023100	2.26160400	-2.11475600
H	-4.84410700	-4.08534200	1.78105000
H	-1.63599900	2.91631900	0.25075600
H	-5.21845700	4.03623000	-0.42418700
C	-2.95886900	0.56068400	-0.28314000
C	-3.53236600	1.56800600	-1.19362900
C	-2.70456900	2.38081600	-2.16947400
C	-3.36731200	3.08500800	-1.02385400
C	-4.57434600	3.93934900	-1.31451900
O	-1.63032700	0.44172700	-0.15564800

Sm	0.42623200	0.02777600	-0.01402800
O	-0.18799000	-1.31542200	-2.00651700
C	-1.00358600	-0.75208500	-3.06338800
C	-1.50348300	-1.94097400	-3.86805700
C	-0.42062800	-2.98798300	-3.62530500
C	-0.07948500	-2.74036100	-2.16957100
I	1.33326000	2.40401100	-1.77220100
I	0.11993100	-2.50248000	1.73184900
O	2.62545900	0.43257900	1.32139500
C	3.78657700	1.13848600	0.84193300
C	4.61573000	1.43683800	2.07736400
C	4.29968600	0.23685900	2.96388600
C	2.82153200	0.02910800	2.69161300
O	0.12096200	1.55549200	1.92249400
C	0.74624000	2.83747100	2.13597200
C	-0.19461000	3.59762000	3.05430800
C	-0.83920000	2.47129900	3.85755500
C	-1.01762500	1.40021900	2.79998700
O	2.49290000	-1.17895800	-1.04272100
C	2.95332400	-1.00080600	-2.39826800
C	4.29659200	-1.70341700	-2.46216800
C	4.09880300	-2.84366200	-1.46895100
C	3.30852600	-2.15863500	-0.37021500
H	4.32661800	0.48273400	0.13729900
H	3.44425400	2.02336700	0.28908000
H	2.19157000	0.66686400	3.33493800
H	2.47536700	-1.00830200	2.79273200
H	4.27286500	2.36874600	2.55558400
H	5.68396300	1.54852700	1.84578900
H	4.50808800	0.40517900	4.02942500
H	4.87822800	-0.64346200	2.63946500
H	1.72711300	2.66554200	2.61044000
H	0.90321500	3.30952500	1.15576300
H	-1.93128500	1.54610600	2.20498400
H	-1.00125700	0.37062300	3.18304800
H	-0.95553800	4.13040700	2.46475600
H	0.33735000	4.33225400	3.67446300
H	-1.79284400	2.75558400	4.32292800
H	-0.15941800	2.12320900	4.65231800
H	2.21882800	-1.46434700	-3.07789600
H	2.98539200	0.07747100	-2.60457900
H	3.97368200	-1.63480000	0.33705800
H	2.64244500	-2.81761700	0.20346600
H	5.09961300	-1.03092900	-2.11905200
H	4.54476000	-2.03784300	-3.47887600
H	5.03735200	-3.27903000	-1.09904300
H	3.50509600	-3.65278200	-1.92502300
H	-1.80221700	-0.16042300	-2.59835300
H	-0.35838900	-0.08114800	-3.65237700
H	0.93790500	-3.03350000	-1.87993100
H	-0.78984200	-3.23292200	-1.48600500
H	-1.64334700	-1.69201300	-4.92873200
H	-2.46591500	-2.29540500	-3.46847000
H	0.45596500	-2.79579600	-4.26575000
H	-0.75802200	-4.01806300	-3.80496100
H	-2.11490400	4.59147000	-0.11441100
H	-4.27504900	4.95580800	-1.62299700
H	-3.06925200	3.62548100	1.04415100
H	-5.18450400	3.51208000	-2.12532500
C	-2.49733000	3.57970600	0.10104000
C	-3.81620000	-0.56603800	0.21984700
C	-4.50674900	-1.98347400	2.21250900
C	-3.65279300	-0.81882400	1.72320500
C	-3.55486500	-1.86544400	-0.56208500
C	-4.39992500	-3.03267200	-0.06458000
C	-4.20620800	-3.25725900	1.43050200
H	-4.86833600	-0.27438700	0.04220700
H	-5.57761400	-1.72961200	2.09763700
H	-4.33885000	-2.14802800	3.28960300
H	-2.59181900	-1.04089300	1.92466900
H	-3.90472100	0.10249000	2.27477100
H	-2.48481900	-2.11337700	-0.45524600
H	-3.74224100	-1.68541400	-1.63463500
H	-4.15219700	-3.94902100	-0.62692000
H	-5.46720800	-2.82261600	-0.26630200
H	-3.15949500	-3.55560800	1.61901600

TS I

E_{vacuum} = -26109.9045
E_{sol} = -26109.9179
$-TS$ = -0.1419
G_{sol} = -26109.2325

H	-4.50351300	1.09394300	-1.85194700
H	-3.02323500	2.66158700	-3.15673500
H	-1.65824200	2.37637600	-1.99891700
H	-4.85882400	-3.97717200	2.04700400
H	-2.00218100	2.70045900	0.42232500
H	-5.45431000	3.85653400	-0.51799300
C	-2.93343900	0.30302500	-0.66465100
C	-3.47931900	1.26562800	-1.51436700
C	-2.74639800	2.41273000	-2.12417600
C	-3.49051200	3.12448400	-1.05332600
C	-4.76089300	3.83683600	-1.37589700
O	-1.63817600	0.32611500	-0.31214500
Sm	0.42280500	0.02900800	-0.03860400
O	-0.03665000	-1.34458000	-2.04875700
C	-0.77749800	-0.80505700	-3.17191700
C	-1.24135200	-2.01421500	-3.96750100
C	-0.18676500	-3.06348000	-3.62841600
C	0.07077300	-2.77382100	-2.16291200
I	1.32379700	2.38013200	-1.82987400
I	0.12580300	-2.43107400	1.80944700
O	2.55757300	0.57154800	1.36296200
C	3.73095400	1.26133700	0.88762700
C	4.52439800	1.61576900	2.13225200
C	4.17480100	0.46268900	3.06691700
C	2.70370700	0.25565700	2.76147000
O	-0.02454800	1.59443800	1.84840200
C	0.49635800	2.93157300	2.00136100
C	-0.37505900	3.58869000	3.05917100
C	-0.82780700	2.39606100	3.89708300
C	-1.05031500	1.34118400	2.83251600
O	2.55765300	-1.16817400	-0.92667900
C	3.08457900	-1.00306500	-2.25911800
C	4.46870900	-1.62162000	-2.22016000
C	4.27129100	-2.75537100	-1.21939900
C	3.36878400	-2.10356100	-0.18815100
H	4.29157600	0.57731500	0.22791700
H	3.40339200	2.12146600	0.28860500
H	2.06804600	0.94444600	3.34417700
H	2.33852300	-0.76852800	2.91706700
H	4.17247900	2.57113300	2.55454300
H	5.59963700	1.71212600	1.92784000
H	4.35440300	0.68167100	4.12851100

H	4.75359400	-0.43732700	2.80242100
H	1.54737700	2.84943100	2.32443000
H	0.46967300	3.42631800	1.02051700
H	-2.03167900	1.44493000	2.34585600
H	-0.92846000	0.30442000	3.17571300
H	-1.24314000	4.07554500	2.58983100
H	0.17436100	4.34718400	3.63361600
H	-1.73600700	2.59215600	4.48318000
H	-0.03173700	2.08258000	4.59218900
H	2.42353700	-1.53379900	-2.96465600
H	3.06179200	0.06863900	-2.49874600
H	3.95066500	-1.54245400	0.56226000
H	2.69676700	-2.79214900	0.34190500
H	5.20492700	-0.89527100	-1.83888100
H	4.80425900	-1.95742700	-3.21100200
H	5.20639700	-3.12821200	-0.77919400
H	3.75876400	-3.60519000	-1.69908800
H	-1.60169600	-0.19559700	-2.77776700
H	-0.08749600	-0.16082500	-3.73918500
H	1.06779500	-3.06334800	-1.80541900
H	-0.68299800	-3.24194500	-1.50981100
H	-1.31499800	-1.79782100	-5.04193600
H	-2.23060600	-2.34563700	-3.61611200
H	0.72808300	-2.89947200	-4.22145000
H	-0.52416700	-4.09557500	-3.79610300
H	-2.31949900	4.43780900	0.19794500
H	-4.57245900	4.89305000	-1.66048400
H	-3.49196300	3.43241200	1.08397100
H	-5.28670300	3.37062500	-2.22434400
C	-2.79494100	3.43558100	0.22750600
C	-3.78708900	-0.77513300	-0.05441600
C	-4.65636300	-1.81503700	2.09136000
C	-3.79233900	-0.71864300	1.47836200
C	-3.36271600	-2.17386400	-0.52168700
C	-4.21806900	-3.27503200	0.09522000
C	-4.21175500	-3.19448600	1.61780700
H	-4.82180200	-0.60126200	-0.40298400
H	-5.71306600	-1.65340400	1.80636600
H	-4.61815900	-1.75757100	3.19188000
H	-2.75392600	-0.83954200	1.82895900
H	-4.13691000	0.27712100	1.80515400
H	-2.30872000	-2.32768500	-0.23411300
H	-3.41285700	-2.21820200	-1.62283800
H	-3.86111800	-4.26404600	-0.23746300
H	-5.25776600	-3.17874500	-0.27053000
H	-3.18768900	-3.38759600	1.98399200

Int II

E_{vacuum} = -26109.9229

E_{sol} = -26109.9360

$-TS$ = -0.1443

G_{sol} = -26109.2515

H	-4.30246000	2.29214500	-0.91577200
H	-2.49751600	3.54925700	-2.18263200
H	-1.25113100	2.73686500	-1.22433200
H	-5.64322400	-3.59204900	1.09327500
H	-2.72845500	3.18145500	1.53165600
H	-1.96363800	6.46132700	-0.05133900
C	-2.89159700	0.85696200	-0.34210400
C	-3.23500200	2.07811500	-0.81267000
C	-2.27296100	3.16601000	-1.17014800
C	-2.23071900	4.32550700	-0.21728600
C	-1.71406700	5.62624100	-0.72611700
O	-1.62184100	0.49162200	-0.14862200
Sm	0.40445300	-0.08937700	-0.07532800
O	-0.11006100	-0.38488700	-2.50638500
C	-0.53861100	0.64515300	-3.41087000
C	-1.88909300	0.16931500	-3.89633400
C	-1.66037300	-1.33586000	-4.04252900
C	-0.62527100	-1.65310600	-2.95990300
I	1.78783800	2.55422800	-0.94410200
I	-0.40567900	-2.90218400	0.95321200
O	2.54012700	-0.48572300	1.35574500
C	3.85113200	0.03310500	1.06031900
C	4.63531300	-0.10690600	2.35058500
C	4.04198000	-1.38519400	2.93365600
C	2.57138800	-1.23654200	2.58742400
O	0.21350600	0.93412400	2.17559000
C	1.03575700	1.95328500	2.78374500
C	0.24484700	2.45543400	3.98072600
C	-0.59713500	1.23835000	4.35160400
C	-0.95578300	0.68929100	2.98650100
O	2.22355400	-1.37580500	-1.37350600
C	2.84848300	-0.86691500	-2.56922700
C	4.12371600	-1.67168200	-2.71824500
C	3.68320900	-3.03571700	-2.19730800
C	2.79168500	-2.65602900	-1.02751400
H	4.29515900	-0.57380700	0.25222000
H	3.73312200	1.06269300	0.69673100
H	2.02493400	-0.66237300	3.35459500
H	2.03865000	-2.18242600	2.42095000
H	4.43789500	0.74955600	3.01567600
H	5.71907600	-0.15952400	2.17758700
H	4.20608600	-1.49841800	4.01408900
H	4.46835900	-2.26870600	2.43121600
H	1.98610600	1.48407200	3.08593900
H	1.24897700	2.71871600	2.02504700
H	-1.80748300	1.21925500	2.53497000
H	-1.15198200	-0.39121700	2.96453200
H	-0.40683100	3.29159300	3.68485100
H	0.89992100	2.80218400	4.79180800
H	-1.48673500	1.48526400	4.94692000
H	0.00223200	0.50568600	4.91641900
H	2.16676000	-1.02998300	-3.42066400
H	2.99427000	0.21422400	-2.43783700
H	3.36706700	-2.53398600	-0.09495500
H	1.96858400	-3.35538700	-0.83069600
H	4.92129100	-1.25233200	-2.08343600
H	4.48783000	-1.68979300	-3.75471600
H	4.51520400	-3.68525300	-1.89226900
H	3.10191900	-3.56758900	-2.96764900
H	-0.54989400	1.59011100	-2.85619000
H	0.19770500	0.72437600	-4.23179300
H	0.21375100	-2.25962600	-3.33926500
H	-1.05247800	-2.16583800	-2.08610500
H	-2.19356800	0.65073000	-4.83593600
H	-2.64581200	0.38850900	-3.12785100
H	-1.25661600	-1.56839500	-5.03978500
H	-2.57955400	-1.92220500	-3.90825300
H	-1.04339300	3.74473100	1.47319000
H	-0.60688400	5.60917900	-0.82023400
H	-2.34119800	4.90035100	1.86133800
H	-2.11040600	5.85987700	-1.72769200
C	-2.09101000	4.02909500	1.23442400

C	-3.94390600	-0.17974700	-0.01784500
C	-4.97938400	-1.63911400	1.77329600
C	-3.90772600	-0.60292800	1.45372800
C	-3.82773100	-1.41404600	-0.91881800
C	-4.89213300	-2.45782400	-0.59859900
C	-4.84852300	-2.86109100	0.87096000
H	-4.92365400	0.29214300	-0.21329600
H	-5.98034400	-1.18841900	1.63665800
H	-4.91302100	-1.93635100	2.83282400
H	-2.91607800	-1.03471900	1.66579400
H	-4.02681000	0.28647100	2.09517900
H	-2.82918000	-1.85825700	-0.77076400
H	-3.90261900	-1.10598400	-1.97466500
H	-4.75887900	-3.34239100	-1.24316200
H	-5.89137900	-2.04613800	-0.83474400
H	-3.88535700	-3.36024500	1.07876000

PreTS II

E_{vacuum} = -26418.1850

E_{sol} = -26418.1996

$-TS$ = -0.1636

G_{sol} = -26417.4131

H	1.22068200	4.78772700	-0.51852600
H	-0.89209200	2.71933300	0.43420900
H	-1.30279300	4.15666000	-0.53274800
H	6.57072000	1.76226100	-2.34964300
H	-0.41803100	2.95620000	2.86208100
H	-1.36295500	6.49153000	2.30214100
C	1.61018400	2.73557900	-0.42378100
C	0.80015500	3.80793800	-0.27419400
C	-0.61181800	3.76700000	0.23947700
C	-0.77435000	4.57707700	1.49470800
C	-0.96812600	6.05018900	1.37251700
O	1.22322600	1.48686900	-0.16092200
Sm	0.50980400	-0.49136200	0.03910300
O	2.26100400	-0.46115300	1.79277800
C	2.30810100	0.59593100	2.78154600
C	3.71932700	0.56003300	3.34435700
C	4.11645300	-0.90039700	3.14759800
C	3.49128600	-1.20732600	1.80141200
I	-1.52967300	0.10194300	2.29642300
I	2.50269200	-1.69996400	-1.97808000
O	-1.21355200	-2.19418000	-0.84423500
C	-2.33727800	-2.73158800	-0.11785100
C	-3.24841000	-3.33479800	-1.17109900
C	-2.25067600	-3.78271900	-2.23386100
C	-1.24661500	-2.64621800	-2.21416800
O	-0.93583200	0.42527400	-1.75045900
C	-2.35645900	0.64851800	-1.59792200
C	-2.69434300	1.78273500	-2.54969800
C	-1.62783600	1.63255000	-3.63044800
C	-0.41251300	1.26101900	-2.80635000
O	0.74655800	-2.80287500	1.21462700
C	0.64091600	-3.01404000	2.63681900
C	0.35083500	-4.49279000	2.79553400
C	1.16034100	-5.08492300	1.64681900
C	0.92876400	-4.06739700	0.54367100
H	-1.96058900	-3.49316800	0.58680400
H	-2.78870500	-1.91808200	0.46512800
H	-1.56607300	-1.80321400	-2.84894700
H	-0.22407600	-2.92779100	-2.50046000
H	-3.92648500	-2.56589800	-1.57472300
H	-3.86410400	-4.15168700	-0.76932000
H	-2.69739800	-3.92577400	-3.22735600
H	-1.76877400	-4.72879300	-1.93759400
H	-2.87936000	-0.28402300	-1.86665600
H	-2.56089800	0.88037500	-0.54448100
H	0.05947600	2.14648300	-2.35325300
H	0.34956400	0.67593500	-3.33949500
H	-2.59008500	2.74913200	-2.03385600
H	-3.72251600	1.70522000	-2.92689600
H	-1.46639400	2.54772500	-4.21648100
H	-1.88960700	0.81885600	-4.32672500
H	1.60131900	-2.74257000	3.10790800
H	-0.14164200	-2.34388000	3.01682800
H	0.01179400	-4.28777200	-0.02651800
H	1.75971700	-3.96001900	-0.16673200
H	-0.72450900	-4.68971000	2.65415900
H	0.64029100	-4.87303400	3.78491800
H	0.84387000	-6.09543800	1.35348700
H	2.22834900	-5.12810100	1.91522500
H	2.04478300	1.53773100	2.28111000
H	1.54175100	0.37168400	3.53962600
H	3.24091400	-2.26339400	1.63591600
H	4.12165500	-0.86730500	0.96360200
H	3.75292400	0.88601800	4.39278300
H	4.38373700	1.21389400	2.75933400
H	3.67082200	-1.53227700	3.93337500
H	5.20287500	-1.06385500	3.14950000
H	0.91628300	4.12308600	2.75044000
H	-0.01129600	6.57503800	1.16080300
H	-0.55826800	4.56202900	3.64219900
H	-1.65239700	6.30672600	0.54664300
C	-0.19070800	4.02867200	2.75106500
C	3.01995200	2.88098200	-0.95134600
C	4.56663400	2.39166500	-2.90327500
C	3.13938800	2.31896100	-2.37308800
C	4.03190800	2.20890600	-0.02062700
C	5.45537700	2.27838600	-0.55979200
C	5.54074400	1.68987700	-1.96353300
H	3.24748100	3.96138800	-0.98803100
H	4.86772000	3.45050800	-3.01406200
H	4.61610500	1.94461300	-3.90939100
H	2.81107500	1.26618200	-2.36481400
H	2.45317600	2.86900000	-3.03844200
H	3.73546800	1.15347800	0.09585500
H	3.97137900	2.67454700	0.97785700
H	6.14431600	1.75177700	0.12227100
H	5.78880900	3.33257700	-0.58503100
H	5.28787400	0.61491000	-1.92334400
C	-6.13680600	1.42085400	-1.18183800
C	-5.34227000	1.26184100	-0.03514300
C	-5.25459300	-0.00400500	0.56909000
C	-5.94526500	-1.08364000	0.02854600
C	-6.72619900	-0.92137000	-1.11649100
C	-6.82029200	0.33385800	-1.71771000
C	-4.59215400	2.35255700	0.49880100
C	-3.89500500	3.23462600	0.95716000
H	-3.22839000	3.97328400	1.36951000
H	-4.62658200	-0.12304100	1.45366200
H	-5.87385800	-2.06231600	0.50793800
H	-7.26676800	-1.77168900	-1.53708900
H	-7.43440800	0.46817900	-2.61036900
H	-6.20682000	2.40552600	-1.64661600

TS II

E_{vacuum} = -26418.1753
E_{sol} = -26418.1935
$-TS$ = -0.1609
G_{sol} = -26417.4059

H	-1.06399700	4.56354600	-0.55156500
H	-2.01656000	1.77372700	0.44069300
H	-3.04905500	2.88390100	-0.49410000
H	5.16315200	4.34959100	-2.10167400
H	-1.59941900	2.19024400	2.83712900
H	-3.90748200	5.07838500	2.33180700
C	0.22533500	2.92695900	-0.40480200
C	-0.99257100	3.50368300	-0.29038200
C	-2.22719600	2.83586800	0.24419300
C	-2.68221700	3.49660100	1.51368600
C	-3.38318900	4.80952900	1.39957800
O	0.45500500	1.64431100	-0.12202800
Sm	0.69517400	-0.44864800	0.01987600
O	2.25477600	0.30458400	1.79281700
C	1.83510800	1.24614700	2.80940900
C	3.11993300	1.82626700	3.37622100
C	4.11907100	0.69446300	3.15220100
C	3.68776800	0.17025400	1.79694300
I	-1.43256300	-0.82514500	2.24224600
I	3.05184500	-0.65820000	-1.95939800
O	-0.16327900	-2.70004300	-0.88914000
C	-0.96038300	-3.67289900	-0.18410500
C	-1.54842000	-4.56545600	-1.26049700
C	-0.44404800	-4.55508100	-2.31202800
C	0.01889400	-3.11123600	-2.26175100
O	-0.89418900	-0.20166400	-1.86571100
C	-2.30307500	-0.48981500	-1.71497000
C	-3.01460400	0.40687700	-2.71286600
C	-1.94747500	0.61951800	-3.78200500
C	-0.69939400	0.74916900	-2.93496300
O	1.87351800	-2.45387200	1.18019800
C	1.87095800	-2.69684200	2.60082000
C	2.20995500	-4.16601600	2.74852400
C	3.19258100	-4.36590400	1.59961800
C	2.56952900	-3.52081000	0.50194500
H	-0.29970600	-4.23605800	0.49797900
H	-1.69920100	-3.13407000	0.42347400
H	-0.59742500	-2.45785200	-2.90001400
H	1.07429900	-2.95619300	-2.52516800
H	-2.47064600	-4.11866100	-1.66691500
H	-1.79314200	-5.56800000	-0.88342000
H	-0.78359500	-4.84784600	-3.31504400
H	0.37234800	-5.23452600	-2.01732200
H	-2.45504500	-1.55916100	-1.93713000
H	-2.59353700	-0.30674400	-0.67215900
H	-0.61256200	1.75541600	-2.49761000
H	0.24069000	0.48534300	-3.43821800
H	-3.27635600	1.36424100	-2.23820500
H	-3.94132900	-0.04985900	-3.08265900
H	-2.12162200	1.50922500	-4.40261800
H	-1.87762500	-0.25707900	-4.44715600
H	2.63991200	-2.06029000	3.07172100
H	0.88423400	-2.40776800	2.98658700
H	1.83065500	-4.09284700	-0.08222300
H	3.28899300	-3.07080800	-0.19584800
H	1.30858300	-4.78304800	2.60083100
H	2.62836700	-4.40303500	3.73644300
H	3.31619000	-5.41488300	1.29682800
H	4.18489300	-3.97282000	1.87391200
H	1.17707200	1.98506900	2.33261600
H	1.25424400	0.68554900	3.55831200
H	3.92330400	-0.88602400	1.61100700
H	4.10395900	0.76621100	0.96839300
H	3.01231000	2.11376500	4.43091800
H	3.42369200	2.71736000	2.80596400
H	4.00101200	-0.08436700	3.92341000
H	5.16639400	1.02628200	3.15610100
H	-0.91405600	3.82761600	2.69013300
H	-2.66589100	5.63201200	1.19813200
H	-2.38493200	3.57927500	3.65267000
H	-4.11727200	4.80785400	0.57859700
C	-1.86303200	3.25446100	2.73691600
C	1.42845300	3.70641700	-0.88943400
C	3.12533600	3.98175400	-2.75774700
C	1.86644600	3.26080700	-2.28934700
C	2.58953400	3.58135400	0.10104100
C	3.84550100	4.29953900	-0.37649400
C	4.26414700	3.81176400	-1.75867500
H	1.13201400	4.76920700	-0.94299900
H	2.91045900	5.05980800	-2.88270400
H	3.42574700	3.60484300	-3.74877900
H	2.05838100	2.17484100	-2.27200600
H	1.04118800	3.43394600	-2.99993000
H	2.80791600	2.50830500	0.23009000
H	2.27074000	3.96631100	1.08484800
H	4.66369500	4.15388100	0.34918000
H	3.65552100	5.38827800	-0.41575300
H	4.53358900	2.74147000	-1.70129900
C	-6.16508400	0.80120800	-0.86717900
C	-5.38653300	0.41156900	0.24047400
C	-4.99187600	-0.93738400	0.34976200
C	-5.36512100	-1.85821000	-0.62225600
C	-6.13876200	-1.46334300	-1.71560300
C	-6.53646900	-0.12979600	-1.83014300
C	-5.02866900	1.34750700	1.24431600
C	-4.66600700	2.16621700	2.08135200
H	-4.63787000	2.74368100	2.98711600
H	-4.38061700	-1.23967400	1.20135000
H	-5.05332400	-2.90051100	-0.52050400
H	-6.43632300	-2.19213900	-2.47216200
H	-7.14446900	0.18784300	-2.67993200
H	-6.47639200	1.84329300	-0.95395500

Int III

E_{vacuum} = -26418.2366
E_{sol} = -26418.2503
$-TS$ = -0.1576
G_{sol} = -26417.4561

H	-1.35215700	4.48406600	-0.42723600
H	-2.16390700	1.58389900	0.33306600
H	-3.20416800	2.70049100	-0.57097700
H	4.82264500	4.70153900	-2.07803600
H	-1.81256600	1.91039700	2.81604200
H	-4.08690400	4.88691100	2.32999000
C	0.02074700	2.91974400	-0.33451500
C	-1.22728600	3.42278600	-0.19613400
C	-2.43096000	2.64551800	0.21919000
C	-3.09035900	3.11757100	1.52961000

C	-3.53987900	4.57897900	1.42458200
O	0.32184600	1.64076500	-0.10980500
Sm	0.72758800	-0.42849100	0.00158600
O	2.19533000	0.41915200	1.81018200
C	1.68436200	1.30758300	2.83325500
C	2.91013500	1.96129700	3.44921000
C	3.99393500	0.91078500	3.22341200
C	3.63347800	0.39091100	1.84594800
I	-1.36792600	-1.05279800	2.20022100
I	3.08695200	-0.38039500	-1.98006400
O	0.12064000	-2.73875700	-0.96784300
C	-0.55378700	-3.81089000	-0.28063000
C	-1.03418600	-4.74768600	-1.37322200
C	0.05458700	-4.58165500	-2.42857700
C	0.33923200	-3.09370700	-2.34956000
O	-0.93922100	-0.31080800	-1.82890300
C	-2.28690100	-0.82119700	-1.70727500
C	-3.13529000	0.04955600	-2.61444900
C	-2.13385400	0.47169600	-3.68474600
C	-0.88810200	0.70667300	-2.85502800
O	2.08264100	-2.34960000	1.13454900
C	2.06908300	-2.64211200	2.54626900
C	2.59806900	-4.05741000	2.66225700
C	3.62326700	-4.09130000	1.53411200
C	2.91597800	-3.30482600	0.44468500
H	0.17051400	-4.30420800	0.39065100
H	-1.34825400	-3.37221200	0.33733800
H	-0.35757300	-2.50706900	-2.97038700
H	1.36546700	-2.80296800	-2.61259400
H	-2.00649600	-4.41067800	-1.76791800
H	-1.15178300	-5.77994000	-1.01579000
H	-0.25360900	-4.89349300	-3.43594500
H	0.94975100	-5.16209200	-2.15157800
H	-2.28282700	-1.87495400	-2.03425200
H	-2.58298300	-0.78187300	-0.65105600
H	-0.90422300	1.69406000	-2.36885500
H	0.06133100	0.58159000	-3.39374500
H	-3.49764000	0.92742500	-2.05998200
H	-4.01142700	-0.48870300	-2.99914000
H	-2.43734700	1.37126400	-4.23763300
H	-1.97084600	-0.34109200	-4.41166800
H	2.72925400	-1.92440800	3.06298800
H	1.04182200	-2.50041700	2.90743900
H	2.26421600	-3.95139200	-0.16533900
H	3.58352500	-2.75259300	-0.23058400
H	1.79130000	-4.78481200	2.47499600
H	3.02223800	-4.26630400	3.65405200
H	3.89247000	-5.10512700	1.20702200
H	4.54802500	-3.57748600	1.84304900
H	0.99120700	2.01106400	2.35283200
H	1.12181800	0.69245000	3.55274600
H	3.95139800	-0.64010200	1.64244600
H	4.02283900	1.03696500	1.04230700
H	2.75571500	2.21101200	4.50768700
H	3.16369400	2.88728400	2.91097000
H	3.91524000	0.10598700	3.97265800
H	5.01387400	1.31735400	3.26144100
H	-1.22012000	3.57049000	2.52515600
H	-2.67264300	5.24785600	1.31569700
H	-2.58289300	3.29320400	3.64582000
H	-4.20536000	4.72782000	0.55954500
C	-2.11999700	2.96066700	2.70269900
C	1.17050000	3.78404200	-0.80141500
C	2.79353400	4.23675100	-2.69878800
C	1.59428600	3.42105100	-2.22973800
C	2.36194100	3.68575600	0.15399300
C	3.55981000	4.49721100	-0.32390100
C	3.96775000	4.09510200	-1.73667800
H	0.81457300	4.82984800	-0.80398300
H	2.51040200	5.30376900	-2.77183900
H	3.08880900	3.92051100	-3.71234000
H	1.85297600	2.34934800	-2.26050900
H	0.73898100	3.56933500	-2.90985800
H	2.64680000	2.62360700	0.23273700
H	2.04844200	4.01218700	1.16034100
H	4.40536600	4.37002800	0.37324200
H	3.30497300	5.57329400	-0.30988600
H	4.30267800	3.04201900	-1.73309900
C	-6.27408400	0.68302600	-0.80380300
C	-5.36060700	0.36387200	0.24912500
C	-5.00544500	-1.00978700	0.42842100
C	-5.52312700	-1.98304700	-0.40729800
C	-6.40862000	-1.64928400	-1.44178000
C	-6.77830900	-0.30933000	-1.62476700
C	-4.83527100	1.34657000	1.05587600
C	-4.33334900	2.28300700	1.81212500
H	-4.84872800	2.53276800	2.75509200
H	-4.30856500	-1.26754600	1.22735200
H	-5.23696600	-3.02616000	-0.25203900
H	-6.81434300	-2.42555300	-2.09285900
H	-7.47356100	-0.04235100	-2.42390000
H	-6.56087300	1.72579500	-0.94850300

TS III

E_{vacuum} = -26418.2267

E_{sol} = -26418.2422

$-TS$ = -0.1561

G_{sol} = -26417.4477

C	-2.11303500	2.79875800	0.72230300
C	-2.75799900	1.54782600	1.22797100
C	-4.18213500	1.65776100	-0.62352600
C	-4.07027300	2.96117700	-0.76065300
C	-3.10066800	3.77709400	0.06668800
C	-2.14408800	0.32084900	1.22992300
H	-6.05719600	0.30987000	0.73087500
H	-1.59563800	3.33501800	1.54099400
C	-3.89794200	4.54566400	1.12827300
C	-2.34194200	4.76762700	-0.81598200
H	-3.02922400	5.48652900	-1.29071200
H	-1.61787800	5.34421800	-0.21655500
H	-4.73150500	3.51661300	-1.44630500
H	-1.34542600	2.51892300	-0.01468400
O	-0.97850200	0.09979500	0.63899100
Sm	1.02305300	-0.04799900	-0.03281700
O	-0.02422200	-1.72173300	-1.49086100
C	-1.20537700	-1.38281100	-2.26251900
C	-1.82338000	-2.71311600	-2.65794300
C	-0.62099400	-3.65224100	-2.66947600
C	0.17725600	-3.14747100	-1.48466000
I	0.77040400	2.10505300	-2.22294300
I	1.80469300	-2.32085300	1.90997800
O	3.43264800	0.85702300	0.32766200
C	4.20280900	1.60698600	-0.63283100
C	5.38317800	2.16427000	0.14259800

C	5.60259400	1.08784300	1.20073300
C	4.17721500	0.70734000	1.55224700
O	1.15770000	1.66174900	1.75103500
C	1.51432200	3.05145300	1.59824600
C	1.08451400	3.73299700	2.89068900
C	1.06845500	2.58083400	3.89235800
C	0.54446100	1.44468900	3.04002000
O	2.75434200	-1.15920400	-1.60031700
C	2.63532400	-1.16545500	-3.03899300
C	3.98265400	-1.63887700	-3.54822600
C	4.40128300	-2.61110900	-2.45060900
C	3.93472200	-1.88660200	-1.20192500
H	4.52593100	0.91755300	-1.43137600
H	3.54568300	2.36340800	-1.08247800
H	3.74886000	1.38823300	2.30784500
H	4.04493100	-0.32549600	1.90232700
H	5.11413200	3.12073800	0.61973100
H	6.25734600	2.34108600	-0.49900500
H	6.17032900	1.43495600	2.07495600
H	6.13633800	0.22552500	0.76897600
H	2.60414600	3.09838600	1.44490400
H	1.01730600	3.44365100	0.69959000
H	-0.55067800	1.48209600	2.92365600
H	0.83758700	0.44023000	3.37504600
H	0.07329900	4.15419700	2.78289500
H	1.76322300	4.55006600	3.17151000
H	0.43319800	2.77364300	4.76758400
H	2.08706600	2.35658500	4.24878900
H	1.82892800	-1.86348400	-3.31899400
H	2.34957700	-0.15377000	-3.35709400
H	4.68758600	-1.16108000	-0.85124500
H	3.66335900	-2.53968800	-0.36156500
H	4.69033300	-0.79588200	-3.60691800
H	3.91473900	-2.09635300	-4.54480400
H	5.47921900	-2.82290500	-2.43125700
H	3.86813300	-3.56924400	-2.56280000
H	-1.85300800	-0.75998600	-1.63289800
H	-0.87077900	-0.79104100	-3.12893300
H	1.25836900	-3.33135400	-1.54348200
H	-0.19112900	-3.55351500	-0.52889700
H	-2.33839900	-2.65387500	-3.62564300
H	-2.55783200	-3.03144700	-1.90329100
H	-0.04435800	-3.53486800	-3.60185200
H	-0.89218800	-4.71181400	-2.56558800
H	-1.78458000	4.24264900	-1.60617500
H	-7.08302700	-1.84719000	0.07440400
H	-4.66025000	5.18878800	0.66025900
H	-4.41788200	3.85951200	1.81414300
H	-3.23153200	5.19223600	1.72332000
H	-3.64424600	1.63779800	1.86205400
C	-4.84009800	0.48417400	-1.05024700
C	-4.50089500	-0.14514000	-2.27254500
C	-5.08520000	-1.35178400	-2.63588300
C	-6.00875300	-1.97980400	-1.79614500
C	-6.35695700	-1.36916600	-0.58715000
C	-5.78718800	-0.16018400	-0.21654200
H	-3.77833900	0.34439800	-2.92844700
H	-4.82162000	-1.81047900	-3.59193100
H	-6.45764900	-2.93236700	-2.08282300
H	-3.19836600	-4.59188500	3.23019600
C	-2.79180000	-0.88607000	1.86077200
C	-2.67059400	-2.55477100	3.76580600
C	-2.05505100	-1.30898300	3.13850300
C	-2.86316800	-2.05709900	0.87596000
C	-3.48113700	-3.29704000	1.50794600
C	-2.72911700	-3.70730600	2.76894100
H	-3.82391200	-0.60180300	2.13248700
H	-3.69539000	-2.32354700	4.11236300
H	-2.09644100	-2.84911100	4.65943000
H	-1.00006200	-1.51011600	2.88443400
H	-2.06144100	-0.47396800	3.85932000
H	-3.49555000	-4.12472700	0.77877000
H	-4.53798800	-3.08940300	1.75834800
H	-1.69769300	-3.99844300	2.49806000
H	-1.83478300	-2.28485700	0.54867900
H	-3.43477400	-1.75477200	-0.01522800

TS III'

E_{vacuum} = -26418.2248

E_{sol} = -26418.2400

$-TS$ = -0.1564

G_{sol} = -26417.4459

C	6.56588000	1.29564200	0.68617900
C	6.41836400	1.30749800	2.07657500
C	5.64424600	0.31894800	2.68865800
C	5.00999000	-0.65311900	1.92637600
H	6.04257600	0.33446300	-1.17076700
H	7.17807300	2.05750700	0.19834600
C	2.08860800	0.32285400	-1.21579200
C	2.76306400	-0.74959900	-1.74617000
C	2.17944000	-2.12628100	-1.78075100
C	3.22814300	-3.24435500	-1.69428400
C	4.29328600	-2.81011000	-0.71061000
C	4.38554500	-1.57046200	-0.27597900
C	5.12987600	-0.66743000	0.51696700
O	0.90922500	0.17965500	-0.62546700
Sm	-1.08906200	-0.00810200	0.05347700
O	-1.43320800	0.94973300	-2.19897400
C	-0.80639800	0.36618900	-3.36853000
C	-0.92923600	1.41697100	-4.46077400
C	-2.16197200	2.20653200	-4.02979700
C	-1.99417600	2.23381700	-2.52400500
I	-1.65938100	-2.80134100	-1.14798600
I	-1.12464200	2.79331700	1.32709500
O	-2.57312600	-0.75394500	2.02096200
C	-3.57410600	-1.79192100	1.99548700
C	-3.86264500	-2.10787200	3.45156800
C	-3.63393400	-0.75500300	4.11787600
C	-2.42475400	-0.24119700	3.36085800
O	0.22209900	-1.01261200	1.88816900
C	0.24756000	-2.40376600	2.27287600
C	1.55784400	-2.59135300	3.02121500
C	1.80187800	-1.19751500	3.59212600
C	1.37917600	-0.33197500	2.42568200
O	-3.60391300	0.44420900	-0.39932800
C	-4.35443600	-0.12247600	-1.49358200
C	-5.81036900	0.08171800	-1.12434700
C	-5.75381600	1.40969000	-0.37686200
C	-4.46572200	1.26875900	0.41351900
C	2.56615600	-4.54280600	-1.23242300
H	-4.46714000	-1.39913100	1.47995600
H	-3.17952400	-2.63158600	1.40789000
H	-1.48331300	-0.63338200	3.78064700
H	-2.34842700	0.85273000	3.29402300

H	-3.14334600	-2.85003800	3.83431400
H	-4.87457900	-2.50989200	3.59810700
H	-3.45321600	-0.81851300	5.19969100
H	-4.50054100	-0.09364500	3.95503300
H	-0.62833800	-2.58933300	2.91759100
H	0.15141200	-3.01897100	1.36721400
H	2.15464600	-0.28459000	1.64625000
H	1.06725000	0.68839300	2.68690600
H	2.36148700	-2.85607200	2.31509600
H	1.49153200	-3.38067500	3.78242800
H	2.84226700	-1.01435600	3.89019900
H	1.15613200	-1.01423000	4.46676600
H	-4.09328200	0.42115300	-2.41747500
H	-4.04599400	-1.17066500	-1.60435600
H	-4.63076300	0.75273900	1.37382700
H	-3.94115200	2.21196100	0.61688500
H	-6.15387600	-0.72419900	-0.45543300
H	-6.46693700	0.09573200	-2.00517700
H	-6.62185500	1.59262900	0.27137500
H	-5.67982100	2.24709800	-1.08964300
H	0.23102900	0.11188700	-3.11003100
H	-1.35298500	-0.56007400	-3.60323200
H	-2.92711600	2.34161200	-1.95588500
H	-1.29643400	3.02266100	-2.19938200
H	-1.02473900	0.96525400	-5.45742600
H	-0.04518900	2.07260600	-4.46470700
H	-3.08365800	1.66843300	-4.30588800
H	-2.20963600	3.21515700	-4.46273400
C	5.93532100	0.33004600	-0.08459200
H	6.90645600	2.07721800	2.67660500
H	5.53793900	0.30632500	3.77621500
H	4.41535300	-1.43243600	2.40469300
H	5.04851700	-3.56384000	-0.43037700
H	3.61824600	-0.51610400	-2.38556900
C	3.89783500	-3.47129000	-3.05584700
H	1.56495000	-2.28569600	-2.68607700
H	1.48278500	-2.21943400	-0.93511300
H	4.70779500	-4.21443600	-2.98062100
H	3.16485200	-3.84196400	-3.79073400
H	4.33602400	-2.53926500	-3.44452200
H	3.29336900	-5.36979700	-1.18873200
H	2.12311300	-4.42170100	-0.23152900
H	1.75764200	-4.83418000	-1.92134500
H	3.02851600	5.61198900	-0.56051200
C	2.67171000	1.71187800	-1.21070500
C	3.46608100	3.63418900	0.22847900
C	2.88340600	2.22677300	0.21923100
C	1.79953500	2.69551500	-1.99855500
C	2.37050400	4.10893200	-1.98046500
C	2.58584600	4.60227600	-0.55380300
H	3.65908300	1.65236400	-1.70261000
H	4.47901300	3.61290300	-0.21533400
H	3.59071700	3.98002600	1.26744800
H	1.90749500	2.23494900	0.73255600
H	3.54349500	1.53883900	0.76792500
H	0.79371900	2.69915200	-1.54496500
H	1.69098500	2.33856300	-3.03686200
H	1.70242500	4.79421200	-2.52884800
H	3.33659400	4.11789100	-2.51902200
H	1.60647300	4.68485000	-0.04908200

Int IV

E_{vacuum} = -26418.2749
E_{sol} = -26418.2892
$-TS$ = -0.1545
G_{sol} = -26417.4900

C	-2.66557500	2.27871800	1.56177200
C	-3.26079100	0.92920400	1.10342700
C	-3.69799000	1.26967900	-0.32233000
C	-3.72633500	2.59652900	-0.51268400
C	-3.33470400	3.37991400	0.71603600
C	-2.32390400	-0.23498900	1.23137000
C	-4.59331300	3.92402600	1.40369400
H	-2.87964600	5.28607700	-0.24018300
H	-2.04412500	5.03431000	1.30724200
H	-1.50412700	4.15955300	-0.16233400
C	-2.38342600	4.52818500	0.38782400
H	-5.51647300	-2.84829900	-1.47511200
H	-4.06427100	3.08777600	-1.42897900
H	-1.59405200	2.24835000	1.31509600
O	-1.09142700	-0.10225600	0.73863900
Sm	0.85280300	0.01254400	-0.04393600
O	0.00213500	-1.88881500	-1.36840600
C	-1.28277800	-1.80367600	-2.03711700
C	-1.64828200	-3.23312200	-2.39447300
C	-0.28522100	-3.90586200	-2.52124200
C	0.49158500	-3.24078300	-1.40233300
I	0.06701400	2.08585600	-2.18153100
I	2.09697800	-2.06821700	1.88127900
O	3.15179800	1.24407600	0.06179700
C	3.69395300	2.09157800	-0.96865400
C	4.85012200	2.82611000	-0.31587400
C	5.35719800	1.78734500	0.67939200
C	4.06138500	1.17347400	1.17721900
O	0.99809000	1.71781500	1.77088800
C	1.17564600	3.13720900	1.59275100
C	0.63801400	3.77368800	2.86244700
C	0.90678100	2.69030700	3.90300300
C	0.58548300	1.42967800	3.12491400
O	2.58396600	-0.81781400	-1.79995700
C	2.33675900	-0.86053600	-3.22025000
C	3.66500000	-1.24148300	-3.84779600
C	4.27580000	-2.13018300	-2.76899900
C	3.86810800	-1.39802900	-1.50443100
H	4.03470400	1.45383300	-1.80257100
H	2.88477400	2.73383800	-1.34114700
H	3.63063100	1.74934900	2.01395900
H	4.13703100	0.12292100	1.48941300
H	4.48765000	3.72182400	0.21437600
H	5.60616000	3.14749800	-1.04546900
H	5.95846400	2.21119000	1.49546100
H	5.97044000	1.02996100	0.16440900
H	2.25270800	3.33482200	1.46181500
H	0.64919300	3.43783800	0.67587800
H	-0.49254500	1.21072800	3.11700000
H	1.12997900	0.53323000	3.45188400
H	-0.44351600	3.95254600	2.76983400
H	1.12756800	4.73191400	3.08449400
H	0.28965900	2.78821400	4.80654500
H	1.96585100	2.69803800	4.20840300
H	1.56143600	-1.62003500	-3.41351300
H	1.94584800	0.12024700	-3.52393700
H	4.57552100	-0.58501300	-1.26723000
H	3.75595300	-2.03336200	-0.61468600

H	4.28847800	-0.34628500	-4.00499700
H	3.53987500	-1.74279700	-4.81738900
H	5.36523300	-2.24580800	-2.85259600
H	3.82355600	-3.13502500	-2.79646900
H	-1.99059700	-1.32068200	-1.35390200
H	-1.15373300	-1.16373100	-2.92358300
H	1.57765900	-3.20187700	-1.55880500
H	0.30085800	-3.71207800	-0.42471800
H	-2.25514600	-3.28096100	-3.30770100
H	-2.22759900	-3.69310200	-1.57933100
H	0.17137200	-3.67664900	-3.49844000
H	-0.32128400	-4.99831900	-2.40884700
H	-5.15024600	4.59895900	0.73424000
H	-4.85633500	-1.12726100	0.17367600
H	-5.27146500	3.10670700	1.69448800
H	-2.77255700	2.44465700	2.64549200
H	-4.32896600	4.49201400	2.31115900
H	-4.16275000	0.68746100	1.69632100
C	-4.08408500	0.25019100	-1.30119500
C	-3.85884000	0.44513500	-2.67259100
C	-4.23229800	-0.52243700	-3.60055200
C	-4.83072800	-1.71040000	-3.17819800
C	-5.04991100	-1.92192000	-1.81738200
C	-4.67525100	-0.95449000	-0.88823800
H	-3.34825800	1.35253500	-3.00023000
H	-4.04352100	-0.35260000	-4.66295200
H	-5.12159600	-2.47026500	-3.90677400
H	-1.94139400	-4.74572000	4.20934300
C	-2.60125200	-1.42740900	2.09884300
C	-2.10156100	-2.58234900	4.31512000
C	-1.85238100	-1.35712600	3.44258500
C	-2.25908900	-2.74216600	1.38543500
C	-2.50021500	-3.96604000	2.26095800
C	-1.73802300	-3.86576100	3.57710300
H	-3.68607100	-1.43075100	2.32064700
H	-3.16902400	-2.61765900	4.60409600
H	-1.52685700	-2.50271100	5.25271000
H	-0.77343600	-1.28061400	3.22480700
H	-2.14464300	-0.43716400	3.97742200
H	-1.19557600	-2.70242300	1.09484400
H	-2.84537000	-2.80957300	0.45458300
H	-2.21111500	-4.88205000	1.71853100
H	-3.58218000	-4.05886500	2.47289700
H	-0.65330300	-3.86465500	3.36724700

Int IV’

E_{vacuum} = -26418.2696
E_{sol} = -26418.2835
$-TS$ = -0.1512
G_{sol} = -26417.4887

C	5.52128300	0.74254600	2.36654600
C	6.03102100	-0.39424800	2.98965500
C	5.87789500	-1.63633500	2.37031200
C	5.21823200	-1.73611200	1.15064500
H	4.48813900	1.54202300	0.66020700
H	5.63719400	1.72285100	2.83376500
C	2.19994100	1.14401000	-0.58215200
C	3.21761000	0.43231300	-1.43630900
C	2.62741500	-0.27423600	-2.67309300
C	3.59140800	-1.41935800	-3.03244500
C	4.22460700	-1.68994100	-1.69143200
C	4.04646900	-0.69414200	-0.80613400
C	4.69886600	-0.59939600	0.50639600
O	1.00756100	0.57350700	-0.37697700
Sm	-0.92733600	-0.14212500	0.09290100
O	-1.51318300	1.30955100	-1.83920900
C	-0.81123700	1.20653600	-3.09982200
C	-1.17018400	2.46746600	-3.86742600
C	-2.54927900	2.80980700	-3.31139600
C	-2.38833700	2.45324900	-1.84665800
I	-0.76050600	-2.60486000	-1.76048100
I	-1.80175000	2.18171400	1.93365200
O	-2.31064200	-1.66703700	1.68310300
C	-2.95178800	-2.90715000	1.32975300
C	-3.24917400	-3.59532400	2.64859900
C	-3.51703800	-2.40703400	3.56613800
C	-2.48143100	-1.40407800	3.09175800
O	0.40443100	-1.15986700	1.89274800
C	0.62531500	-2.57976300	2.01266600
C	2.07992500	-2.71346100	2.40988600
C	2.28215200	-1.47939600	3.28308600
C	1.49042700	-0.43212500	2.52532800
O	-3.48790800	-0.26257900	-0.50473400
C	-4.01209000	-0.64585100	-1.79254300
C	-5.51237900	-0.77260100	-1.59998400
C	-5.77719800	0.26989100	-0.51911200
C	-4.57004100	0.08624000	0.37983300
C	2.87791200	-2.63741300	-3.61091600
H	-3.87615600	-2.67731400	0.77174700
H	-2.28077500	-3.46056000	0.65982600
H	-1.50771300	-1.54642200	3.58867500
H	-2.77412000	-0.35186400	3.20811600
H	-2.36744800	-4.15800200	2.99543200
H	-4.09174900	-4.29646500	2.57313300
H	-3.41212600	-2.63941900	4.63482800
H	-4.53506900	-2.01774900	3.40181700
H	-0.04892100	-2.97369000	2.79374300
H	0.37616500	-3.04774900	1.04966200
H	2.08747300	0.03697200	1.73127800
H	1.04144500	0.35035500	3.15294600
H	2.71821400	-2.65206000	1.51620600
H	2.28861100	-3.66082500	2.92603400
H	3.33660000	-1.19238200	3.38835600
H	1.85923700	-1.64424200	4.28773400
H	-3.76187100	0.14640200	-2.51748900
H	-3.50837000	-1.57135400	-2.10236400
H	-4.72526600	-0.74193800	1.09224400
H	-4.26961400	0.98003200	0.94333400
H	-5.76988300	-1.77907200	-1.23192700
H	-6.06866300	-0.59932800	-2.53153200
H	-6.72480700	0.12342600	0.01746800
H	-5.78462200	1.28311200	-0.95341500
H	0.25871400	1.10613300	-2.88068400
H	-1.15837200	0.28882700	-3.60099500
H	-3.31665500	2.16599300	-1.33662500
H	-1.91511300	3.26345500	-1.26883200
H	-1.16297100	2.30560800	-4.95388800
H	-0.45747300	3.27340100	-3.63457000
H	-3.32327000	2.18107500	-3.78137700
H	-2.83041000	3.86229200	-3.45383300
C	4.86488200	0.64095800	1.14150500
H	6.54443200	-0.31568500	3.94994000
H	6.27092700	-2.53728700	2.84652600
H	5.09451300	-2.71689100	0.68722900

H	4.86091300	-2.56456900	-1.52932900
H	3.93993500	1.20710600	-1.75831300
C	4.68023600	-0.94418300	-4.00531200
H	2.46207600	0.41820400	-3.51352800
H	1.65785900	-0.70546800	-2.38160600
H	5.45277200	-1.71732500	-4.14511500
H	4.24469000	-0.72134800	-4.99323200
H	5.17890100	-0.03484600	-3.63495400
H	3.59334900	-3.44806000	-3.82725400
H	2.11589200	-3.01736600	-2.91537000
H	2.37018700	-2.37992400	-4.55460700
H	1.13543800	6.32839400	0.59490800
C	2.29513700	2.62735600	-0.33884400
C	2.05606500	4.51221100	1.35335900
C	2.01923200	3.00619000	1.12134400
C	1.34931300	3.40496100	-1.26971700
C	1.38219500	4.90944800	-1.02940900
C	1.08674400	5.23967100	0.42872800
H	3.32909800	2.93845300	-0.58493700
H	3.08064000	4.89110300	1.17570400
H	1.81811200	4.73438600	2.40628000
H	1.02331800	2.62374600	1.39685900
H	2.74444600	2.49888000	1.77824500
H	0.32932400	3.02537500	-1.09145500
H	1.60118700	3.17536700	-2.31951100
H	0.66207600	5.41643000	-1.69473600
H	2.38096200	5.30177000	-1.29766200
H	0.05630700	4.92371900	0.67253600

Product

E_{vacuum} = -26418.2891
E_{sol} = -26418.3094
$-TS$ = -0.1587
G_{sol} = -26417.5130

C	-2.88428600	2.29846600	1.49004100
C	-3.48240500	0.94578400	1.05239900
C	-3.81424600	1.20895800	-0.41225800
C	-3.80690100	2.52537800	-0.66270900
C	-3.47935900	3.36439500	0.54385800
C	-2.48278000	-0.18425100	1.20063500
C	-4.76196900	3.97296300	1.12347900
H	-2.92435400	5.22182000	-0.44867900
H	-2.14211600	4.98129100	1.12880800
H	-1.59706500	4.04618300	-0.30425800
C	-2.47393700	4.46496200	0.21293700
H	-5.42975800	-3.00411300	-1.55012100
H	-4.05655500	2.96859800	-1.62922800
H	-1.79850100	2.24512800	1.32258700
O	-1.39036700	-0.08443100	0.65745700
Sm	0.96218900	0.04030700	-0.10999500
O	0.01860800	-2.05734100	-1.30451300
C	-1.15055900	-2.05098600	-2.14472200
C	-1.46551300	-3.51225500	-2.42109200
C	-0.08957600	-4.16440500	-2.32473100
C	0.53523500	-3.38827300	-1.17974900
I	0.07794700	2.06433500	-2.43293200
I	1.96226200	-1.96137900	2.22310700
O	3.22772900	1.33082600	0.13264100
C	3.83461300	2.07629100	-0.93509700
C	5.00996600	2.79970000	-0.30086500
C	5.43266600	1.81280300	0.78425200
C	4.08912700	1.30813000	1.28063100
O	0.73236500	1.90468800	1.70810500
C	1.02826000	3.28501300	1.44129000
C	0.66736400	4.02565600	2.71594500
C	1.00230400	2.98465500	3.78069400
C	0.52922200	1.70307900	3.11718100
O	2.77383000	-0.93526400	-1.71618200
C	2.58296000	-1.08196500	-3.13291400
C	3.92070200	-1.55996700	-3.67029200
C	4.44824000	-2.38324300	-2.49809200
C	4.01080600	-1.54055200	-1.31290900
H	4.16439700	1.36625800	-1.71341000
H	3.06790800	2.72949700	-1.37587800
H	3.66861200	1.97980800	2.05116200
H	4.09501900	0.28569800	1.68498100
H	4.67929700	3.74844900	0.15281800
H	5.80333500	3.02886900	-1.02573100
H	6.03455400	2.26449100	1.58494400
H	6.01557200	0.98646600	0.34549400
H	2.10350800	3.37523000	1.21140000
H	0.46074400	3.58986900	0.55120800
H	-0.54765000	1.53596400	3.29310500
H	1.07927300	0.79845500	3.41503100
H	-0.40963900	4.25735000	2.73292200
H	1.22221500	4.96771800	2.82596600
H	0.50925600	3.16379800	4.74618300
H	2.09008700	2.94902700	3.95496700
H	1.78767300	-1.82827500	-3.30158500
H	2.23991500	-0.11645200	-3.53190100
H	4.74307100	-0.74153100	-1.09859400
H	3.83057400	-2.10364900	-0.38511500
H	4.58579000	-0.70306100	-3.86601000
H	3.81916400	-2.13153500	-4.60321300
H	5.53479500	-2.54503700	-2.52210500
H	3.95708400	-3.36979300	-2.47059900
H	-1.95504900	-1.51933200	-1.62163400
H	-0.91157200	-1.49069100	-3.06351100
H	1.63185900	-3.33035400	-1.21292600
H	0.24897400	-3.79651000	-0.19579500
H	-1.95824500	-3.65327300	-3.39232600
H	-2.13337800	-3.91385200	-1.64242700
H	0.47844600	-4.00331400	-3.25602400
H	-0.12569700	-5.24522900	-2.12947000
H	-5.25868000	4.62229300	0.38562000
H	-4.98034100	-1.20250300	0.07822900
H	-5.47898600	3.19011800	1.41666300
H	-3.06785500	2.51990300	2.55251500
H	-4.53570500	4.58474100	2.01207700
H	-4.39496300	0.69617600	1.62064000
C	-4.09865800	0.13850200	-1.37330100
C	-3.75077100	0.28971900	-2.72508700
C	-4.01913100	-0.72096300	-3.64193200
C	-4.62418100	-1.90867400	-3.22831300
C	-4.95864600	-2.07793500	-1.88585300
C	-4.69722900	-1.06375100	-0.96739400
H	-3.21703700	1.18862300	-3.03880000
H	-3.73221300	-0.58771300	-4.68708200
H	-4.82649000	-2.70375900	-3.94881600
H	-2.06953700	-4.48782600	4.39377500
C	-2.79757400	-1.38640100	2.04838400
C	-2.41091300	-2.34691900	4.35430100
C	-2.17409800	-1.15077300	3.43968500
C	-2.25598100	-2.67777900	1.43464100

C	-2.48656300	-3.86912300	2.35536500
C	-1.87311000	-3.63016600	3.73063500
H	-3.89296300	-1.45839200	2.17175500
H	-3.49415200	-2.45243700	4.55057700
H	-1.93055000	-2.16264100	5.32850200
H	-1.08889700	-1.00523900	3.30211300
H	-2.58137000	-0.22930100	3.88958900
H	-1.17589100	-2.54753200	1.25985100
H	-2.72934200	-2.84301500	0.45323500
H	-2.05953800	-4.77677300	1.89910800
H	-3.57243000	-4.05326900	2.46063100
H	-0.77723500	-3.54541100	3.62490900

Product’

E_{vacuum} = -26418.2855

E_{sol} = -26418.3064

$-TS$ = -0.1599

G_{sol} = -26417.5111

C	-5.10304800	-0.46454700	-2.78622700
C	-5.19698400	-1.82595200	-3.06381700
C	-4.95558800	-2.74953300	-2.04524600
C	-4.61259300	-2.31653000	-0.76966600
H	-4.71970200	1.03910300	-1.30238700
H	-5.29325200	0.27021900	-3.57103000
C	-2.43890200	1.27405400	0.19534100
C	-3.55735800	0.89075600	1.14656700
C	-3.04822500	0.73369400	2.59296100
C	-3.94368600	-0.34247700	3.24139300
C	-4.34518300	-1.14038500	2.02765000
C	-4.14987900	-0.48690300	0.87101400
C	-4.51399500	-0.94694300	-0.47330100
O	-1.42912300	0.58555800	0.13855700
Sm	1.00984100	-0.14862100	-0.01102200
O	1.19079400	2.10956600	1.28093100
C	0.69570300	2.36332500	2.60692100
C	1.01215800	3.82285600	2.88013100
C	2.31679100	4.00689500	2.11044700
C	2.06972300	3.17125200	0.86531100
I	0.62731000	-1.64598100	2.80726300
I	1.83203700	1.38536800	-2.72581300
O	2.36742400	-2.25462200	-0.73457800
C	3.07800100	-3.16489900	0.12012500
C	3.46616800	-4.33332300	-0.76963100
C	3.65652600	-3.64575800	-2.11882400
C	2.51775200	-2.64274100	-2.11052200
O	-0.44759800	-1.70007100	-1.44913800
C	-0.97082300	-2.91222600	-0.90833000
C	-1.38913100	-3.75008100	-2.12294500
C	-1.44100100	-2.73229700	-3.28070800
C	-1.25590400	-1.39043300	-2.58348500
O	3.52969600	0.21222500	0.63711700
C	4.00854100	0.30193800	1.98741700
C	5.46665300	-0.11138100	1.92047000
C	5.87423000	0.43776500	0.55611300
C	4.63668700	0.15416400	-0.27978200
C	-3.18977500	-1.17029600	4.27760300
H	3.96865300	-2.64753600	0.51852900
H	2.42560800	-3.42051700	0.96698700
H	1.57338000	-3.09948100	-2.45173700
H	2.69616200	-1.73306600	-2.70258100
H	2.64222200	-5.06331800	-0.82637400
H	4.36120200	-4.85806800	-0.40766800
H	3.60757200	-4.33049700	-2.97688000
H	4.62828600	-3.12614600	-2.15195300
H	-0.18407000	-3.36029300	-0.28722500
H	-1.83096500	-2.67271400	-0.26016500
H	-2.22289100	-0.98042500	-2.24392500
H	-0.71853700	-0.63166100	-3.16803400
H	-2.36325800	-4.22800300	-1.94911600
H	-0.66202100	-4.54903200	-2.32818700
H	-2.38633500	-2.77080200	-3.83883500
H	-0.61962600	-2.90688000	-3.99123400
H	3.90967000	1.34735300	2.33284200
H	3.37278200	-0.33941100	2.61535700
H	4.66511800	-0.85703600	-0.71996800
H	4.45053200	0.87338400	-1.09000900
H	5.55278200	-1.21018300	1.93739800
H	6.05833600	0.28965700	2.75510400
H	6.77659200	-0.03155600	0.14014400
H	6.05539600	1.52297000	0.62124500
H	-0.37764800	2.12775400	2.62178600
H	1.20138400	1.68194600	3.31095200
H	2.97202400	2.71633600	0.43513200
H	1.56819800	3.74787800	0.07033000
H	1.09947100	4.03857000	3.95394700
H	0.22669500	4.47281500	2.46134600
H	3.16057900	3.59967300	2.69066600
H	2.54367900	5.05494900	1.87104700
C	-4.76807600	-0.03183500	-1.50497000
H	-5.45626100	-2.16756300	-4.06757500
H	-5.02538100	-3.81974300	-2.25139400
H	-4.39740100	-3.04826000	0.01133100
H	-4.82823300	-2.11714700	2.11152800
H	-4.35046100	1.65592100	1.07864200
C	-5.19842100	0.27748200	3.87178700
H	-3.04857100	1.68319000	3.14950400
H	-2.01875700	0.34469900	2.54858000
H	-5.89763100	-0.50436800	4.20769800
H	-4.92772000	0.88543700	4.75006700
H	-5.73483900	0.92112300	3.15682300
H	-3.83592700	-1.95978000	4.69422200
H	-2.29382700	-1.63618200	3.84115300
H	-2.86090900	-0.53334600	5.11430800
H	-1.15867300	5.71318800	-2.54973800
C	-2.53958300	2.54895300	-0.60448900
C	-1.96906600	3.71099700	-2.77355500
C	-2.00129100	2.38528100	-2.02531000
C	-1.74221100	3.62307600	0.16170600
C	-1.70823900	4.94270900	-0.59934200
C	-1.16091400	4.75316000	-2.00933900
H	-3.59658000	2.86882900	-0.63565000
H	-2.99946300	4.08359200	-2.92763600
H	-1.53471600	3.55240800	-3.77258900
H	-0.97825700	1.97760100	-1.97790500
H	-2.61015400	1.64560600	-2.56943800
H	-0.71735400	3.24031900	0.29252300
H	-2.16738200	3.76570900	1.17010800
H	-1.09896800	5.67217900	-0.04015900
H	-2.72908900	5.36381500	-0.65149800
H	-0.11091300	4.41360300	-1.95582200

Phenyl Dimethylcyclopropyl Ketone Reactant

E_{vacuum} = -26106.3197
E_{sol} = -26106.3382
$-TS$ = -0.1430
G_{sol} = -26105.7216

C	-5.32303800	-3.03324600	0.99265300
C	-5.96618500	-1.86895300	0.56986400
C	-5.22772800	-0.81868600	0.03579000
C	-3.83125000	-0.91961200	-0.07387400
C	-3.19261900	-2.09018300	0.36507400
C	-3.93521700	-3.14017000	0.88924600
C	-3.00445100	0.18251700	-0.60756500
C	-3.67057800	1.31295000	-1.27837800
C	-2.88027300	2.48831900	-1.79884300
C	-3.71491500	2.69137000	-0.58084000
C	-5.01926500	3.43720900	-0.70692600
O	-1.77310500	0.15415200	-0.46250000
Sm	0.65452500	0.05548800	-0.09750500
O	0.28262200	-0.79057600	-2.54653200
C	-0.70038500	-0.23570000	-3.42299900
C	-1.75144600	-1.33576300	-3.61805400
C	-1.04912700	-2.61169700	-3.11337000
C	0.39182200	-2.16632600	-2.91421400
I	1.02063900	3.10532800	-0.92117600
I	0.58469700	-3.04539000	0.88200200
O	2.53933800	0.39245200	1.66360500
C	3.62398000	1.32285800	1.48257000
C	4.47874600	1.20201800	2.73421200
C	4.23589400	-0.24657400	3.14790400
C	2.76014300	-0.40497000	2.83651900
O	-0.39953900	0.81178500	2.12796200
C	-0.03547200	1.99100000	2.86639200
C	-1.01762600	2.06183800	4.02401300
C	-1.32221300	0.58640600	4.27094500
C	-1.37581700	0.04688100	2.85406900
O	2.95865400	-0.40744500	-1.20361200
C	3.35568100	0.18272200	-2.45797100
C	4.62175100	-0.55186300	-2.87691900
C	4.45388800	-1.90584300	-2.19234700
C	3.83138600	-1.49547600	-0.87263900
H	4.17422400	1.02889800	0.57380700
H	3.19844400	2.32199200	1.31257400
H	2.13374600	-0.01357600	3.65979400
H	2.44057300	-1.43242500	2.61219800
H	4.11564000	1.88673200	3.51785400
H	5.53474800	1.43779500	2.54258300
H	4.47021800	-0.45195000	4.20165300
H	4.83141900	-0.93269000	2.52381600
H	1.00336300	1.87050600	3.21628500
H	-0.07023900	2.84974000	2.18043600
H	-2.36703200	0.20903700	2.39683500
H	-1.11184300	-1.01647800	2.75738600
H	-1.93284900	2.59398600	3.71928900
H	-0.59724800	2.57906100	4.89762600
H	-2.25700200	0.41404700	4.82202800
H	-0.50035300	0.11081400	4.83059100
H	2.52864700	0.03144700	-3.16890600
H	3.48637900	1.26378500	-2.30644100
H	4.60195100	-1.14027000	-0.16244100
H	3.22893700	-2.27407700	-0.38253200
H	5.51560100	-0.03916700	-2.48654600
H	4.72167100	-0.61830200	-3.96915400
H	5.39581600	-2.45720400	-2.06513700
H	3.75395900	-2.54302500	-2.75690800
H	-1.07849900	0.68206400	-2.95784700
H	-0.21000300	0.02745200	-4.37751000
H	0.98004000	-2.24185200	-3.84926300
H	0.90973500	-2.69267400	-2.10133700
H	-2.04577000	-1.41151300	-4.67452800
H	-2.66127500	-1.13081400	-3.03695500
H	-1.13099100	-3.45537500	-3.81242500
H	-1.46334600	-2.93101600	-2.14594700
H	-5.73817100	0.09337200	-0.27643000
H	-2.10519600	-2.17318000	0.30051600
H	-7.05033300	-1.77895500	0.66020800
H	-3.42058400	-4.04363100	1.22190500
H	-5.90675500	-3.85757200	1.40776300
H	-4.59124500	1.05646300	-1.80478000
C	-3.01906100	2.85707100	0.74619000
H	-3.22291100	2.92394000	-2.73952100
H	-1.79614800	2.46462200	-1.65512000
H	-5.73943200	3.11324700	0.06239800
H	-5.48116700	3.28808000	-1.69433200
H	-4.85317500	4.51853500	-0.57335700
H	-2.75642700	3.91600000	0.89659500
H	-2.08768200	2.27897400	0.79478800
H	-3.67630700	2.54662200	1.57559700

Int I

E_{vacuum} = -26106.3116
E_{sol} = -26106.3271
$-TS$ = -0.1398
G_{sol} = -26105.7086

C	-5.15326400	-3.03359500	1.16958700
C	-5.71800100	-2.05390400	0.34374800
C	-4.97690900	-0.95835400	-0.07142300
C	-3.61580700	-0.80663100	0.31511700
C	-3.06975800	-1.79531000	1.17857000
C	-3.82547900	-2.88162800	1.58861600
C	-2.82076400	0.27611600	-0.15711500
C	-3.39555200	1.31019900	-1.05223600
C	-2.72600100	2.64566700	-1.20553600
C	-4.04315900	2.57444100	-0.48721400
C	-5.26870200	3.09512900	-1.19420500
O	-1.54222200	0.36304800	0.17935700
Sm	0.56117000	0.01343000	0.02680100
O	-0.39956400	-1.75364800	-1.41231900
C	-1.28735800	-1.47939000	-2.51913200
C	-2.26858500	-2.63330500	-2.52628100
C	-1.38811500	-3.79219400	-2.06802500
C	-0.52747300	-3.13560300	-1.00337300
I	0.87572400	1.78120300	-2.47870300
I	0.79119500	-1.84951500	2.46631300
O	2.90695300	0.88869500	0.64660400
C	3.90727500	1.39282000	-0.26169300
C	4.92215100	2.09989400	0.61549400
C	4.87689200	1.25500000	1.88413900
C	3.39456000	0.95612900	2.00492600
O	0.46280400	1.90696100	1.62991300
C	0.75995600	3.27011500	1.27583900
C	-0.37693200	4.07864500	1.86637200
C	-0.65899800	3.32081600	3.16086700
C	-0.45480400	1.86985400	2.75202900
O	2.37483500	-1.48495600	-0.96932400
C	2.48554600	-1.77464800	-2.37749700

C	3.90425600	-2.27240700	-2.55984700
C	4.12039200	-3.04687800	-1.26377200
C	3.42290000	-2.16317100	-0.24330600
H	4.35721800	0.53743900	-0.79525000
H	3.40444800	2.03155100	-0.99934900
H	2.85347000	1.76330400	2.52455500
H	3.15283300	0.00469900	2.49794300
H	4.59294600	3.13000800	0.82772500
H	5.91569100	2.14860300	0.14899300
H	5.27180500	1.76633400	2.77277600
H	5.44945200	0.32333500	1.74550400
H	1.73202900	3.54883700	1.72227500
H	0.83925800	3.32577700	0.18059600
H	-1.37738100	1.38938900	2.39519500
H	-0.00834000	1.23892500	3.53281900
H	-1.25142600	4.04469500	1.19896900
H	-0.10427100	5.13107200	2.02486300
H	-1.66998100	3.49561400	3.55378700
H	0.06358100	3.61174700	3.93974500
H	1.74856500	-2.55290500	-2.63854600
H	2.24319300	-0.85419600	-2.92604500
H	4.10062400	-1.39767800	0.16774400
H	2.96346000	-2.70394300	0.59507700
H	4.60254200	-1.42227100	-2.62697900
H	4.02101300	-2.88369300	-3.46527900
H	5.17797700	-3.21172600	-1.01581800
H	3.62830200	-4.03097200	-1.32210400
H	-1.74107800	-0.49491200	-2.35167100
H	-0.68070200	-1.43651700	-3.43977800
H	0.48458500	-3.55626500	-0.91289800
H	-1.00974400	-3.15745200	-0.01547700
H	-2.71714700	-2.79074700	-3.51679800
H	-3.07285800	-2.45084300	-1.79760000
H	-0.76744700	-4.16024200	-2.90151900
H	-1.95920800	-4.63851100	-1.66311600
H	-5.44342600	-0.20277000	-0.70544200
H	-2.04352600	-1.68167300	1.53049300
H	-6.75874500	-2.14795100	0.02384500
H	-3.37447200	-3.62191500	2.25440800
H	-5.74366000	-3.89255600	1.49336900
H	-3.89995500	0.91946200	-1.94438900
C	-4.05994000	2.74314400	1.01048900
H	-2.69017800	3.09066500	-2.20123400
H	-1.81964200	2.78974000	-0.61643300
H	-4.11426500	3.81008100	1.28523800
H	-4.92918300	2.23234000	1.45569300
H	-3.15864800	2.31379700	1.46976000
H	-6.18151500	2.60115000	-0.82115900
H	-5.38777900	4.17999300	-1.03446500
H	-5.21006000	2.92253300	-2.27965300

TS I

E_{vacuum} = -26106.2902

E_{sol} = -26106.3051

$-TS$ = -0.1389

G_{sol} = -26105.6876

C	-5.22568000	-3.04736700	1.15798800
C	-5.84975600	-1.87015400	0.73745300
C	-5.11108000	-0.84129900	0.16709500
C	-3.71672600	-0.96169200	-0.01440900
C	-3.10154500	-2.14847000	0.42806800
C	-3.84498200	-3.17338500	1.00183500
C	-2.91862500	0.09936400	-0.62740400
C	-3.50844400	1.08175400	-1.41724400
C	-2.83763800	2.32183400	-1.90890500
C	-3.55995500	2.99118900	-0.79745700
C	-4.86601500	3.66288200	-1.04804900
O	-1.59550400	0.07853100	-0.41483800
Sm	0.49790600	0.00465600	-0.10782400
O	0.26921600	-0.93620500	-2.42398100
C	-0.83819100	-0.59100400	-3.27416600
C	-1.71191600	-1.84112600	-3.31319500
C	-0.74361000	-2.98267000	-2.93950000
C	0.60171200	-2.28449300	-2.78351300
I	1.03970200	2.86645300	-1.09052000
I	0.69897100	-2.88744800	0.99833700
O	2.45162000	0.43233600	1.55602400
C	3.53902700	1.35300900	1.34207700
C	4.13044800	1.58954500	2.71688900
C	3.95859900	0.21805700	3.36123500
C	2.59732200	-0.21127200	2.84118400
O	-0.31759800	0.84806100	2.06903900
C	-0.03545400	2.12035000	2.69053800
C	-1.12601300	2.31601500	3.73272000
C	-1.52134800	0.88122600	4.07244100
C	-1.44917500	0.21694400	2.71419900
O	2.76694400	-0.50459200	-1.17139900
C	3.22395300	0.10068400	-2.40060600
C	4.34517300	-0.79555000	-2.88933800
C	4.95804900	-1.26524500	-1.57428100
C	3.72323200	-1.48433100	-0.71980600
H	4.27407700	0.88377700	0.66474900
H	3.12958500	2.24446200	0.84906800
H	1.77757600	0.13575000	3.49093800
H	2.48218200	-1.29367000	2.69482500
H	3.54605100	2.35010900	3.25989100
H	5.17408100	1.93014200	2.67147600
H	3.99705400	0.23488800	4.45911200
H	4.74229700	-0.47052800	3.00578800
H	0.96560400	2.05711200	3.14686100
H	-0.01475500	2.89188900	1.90786200
H	-2.34981200	0.40473500	2.10979400
H	-1.25518300	-0.86392500	2.73542300
H	-1.98187800	2.85057100	3.29405600
H	-0.77034200	2.89207800	4.59808400
H	-2.52008500	0.79751200	4.52184600
H	-0.79257100	0.42760900	4.76393000
H	2.36269000	0.16962500	-3.07689200
H	3.57045100	1.12153900	-2.17412700
H	3.87978600	-1.33716300	0.35691300
H	3.28984400	-2.48707400	-0.86106300
H	5.05081200	-0.26228300	-3.54085600
H	3.93936400	-1.65346100	-3.44945600
H	5.59298900	-0.47254000	-1.14586800
H	5.56486100	-2.17632800	-1.66736600
H	-1.32782400	0.28834500	-2.84480200
H	-0.42986700	-0.33921900	-4.26907800
H	1.16880600	-2.25895400	-3.73260900
H	1.22805900	-2.70090400	-1.98549800
H	-2.16413100	-1.97561300	-4.30565600
H	-2.52451400	-1.76055500	-2.57874900
H	-0.69879100	-3.77653800	-3.69794400
H	-1.03442900	-3.44054800	-1.98346800
H	-5.61304800	0.08526100	-0.11639800

H	-2.02336000	-2.26380800	0.31929400
H	-6.92711100	-1.74643500	0.87125000
H	-3.33309700	-4.08073600	1.33121300
H	-5.80940200	-3.85082400	1.61148900
H	-4.54528200	0.93372800	-1.71955300
C	-2.88188200	3.17373900	0.51481100
H	-3.13104000	2.63840500	-2.91855000
H	-1.74681800	2.31875100	-1.80001400
H	-2.38515300	4.16249700	0.57844900
H	-3.59657500	3.11062100	1.35338700
H	-2.10204400	2.41315500	0.65828900
H	-5.54780400	3.58376800	-0.18450300
H	-4.72579400	4.74587100	-1.24458800
H	-5.37964100	3.24677000	-1.92889100

Int II

E_{vacuum} = -26106.2995

E_{sol} = -26106.3145

$-TS$ = -0.1409

G_{sol} = -26105.6981

C	-4.55190100	-3.86598000	0.92887900
C	-5.16872800	-3.09472500	-0.05718100
C	-4.66281800	-1.84063100	-0.38801600
C	-3.53171400	-1.32762700	0.26385500
C	-2.91907800	-2.11143000	1.25091400
C	-3.42644700	-3.36548000	1.58240100
C	-2.94092500	-0.01143800	-0.09356100
C	-3.67923100	0.99291900	-0.62267400
C	-3.13624300	2.32656100	-1.02326000
C	-3.88097200	3.49733100	-0.45440300
C	-3.82042300	4.80672600	-1.16131500
O	-1.63600400	0.10599300	0.14484700
Sm	0.47052000	-0.02044200	0.02484400
O	-0.22361600	-2.12457100	-1.09009400
C	-1.17595900	-2.16759000	-2.17805800
C	-1.82102400	-3.53700000	-2.08521800
C	-0.68477900	-4.38747900	-1.52612200
C	-0.05410300	-3.44028300	-0.52314400
I	0.55178000	1.36122900	-2.72929500
I	0.83766500	-1.43969400	2.74024100
O	2.70802100	1.16058400	0.51157100
C	3.62939300	1.65989100	-0.47623900
C	4.48880900	2.66634600	0.26195700
C	4.58993500	2.02753600	1.64333100
C	3.19412000	1.45966000	1.83882600
O	0.14682100	2.13375100	1.21608500
C	0.48309700	3.44115900	0.70798200
C	-0.53752100	4.38347100	1.31975800
C	-0.83776400	3.70527100	2.65310700
C	-0.85672400	2.24379300	2.25402300
O	2.48317800	-1.41379800	-0.76282700
C	2.67495500	-1.86015900	-2.12082500
C	4.12599100	-2.29260900	-2.19097900
C	4.34309700	-2.85888600	-0.79143600
C	3.56901400	-1.87459300	0.06700900
H	4.23264800	0.81648400	-0.85710600
H	3.04063800	2.06770000	-1.30863400
H	2.50492700	2.18897600	2.29400400
H	3.15668200	0.53791200	2.43541300
H	3.97412800	3.63903800	0.32047900
H	5.46116800	2.82412000	-0.22462000
H	4.86590500	2.73284100	2.43921100
H	5.33812300	1.21835100	1.63447100
H	1.50846500	3.68362900	1.03630700
H	0.46002600	3.40013400	-0.39055200
H	-1.82374800	1.93993200	1.82547300
H	-0.58053300	1.54124400	3.05205500
H	-1.44490500	4.41546000	0.69798600
H	-0.15050100	5.40682800	1.42003600
H	-1.78938700	4.02247800	3.10119100
H	-0.03255800	3.90142300	3.37972900
H	1.99165000	-2.70442200	-2.31270400
H	2.40491900	-1.02955500	-2.78732000
H	4.18617400	-1.00342100	0.34274500
H	3.13997500	-2.29951500	0.98483800
H	4.77931700	-1.42135700	-2.36117800
H	4.30783800	-3.01805200	-2.99578100
H	5.39967800	-2.92632300	-0.49781800
H	3.90362300	-3.86659600	-0.71448600
H	-1.87524700	-1.33085000	-2.05454600
H	-0.61794600	-2.02548000	-3.11825100
H	1.02096300	-3.59822500	-0.35745100
H	-0.56689400	-3.46876100	0.44994400
H	-2.19120600	-3.88568600	-3.05908800
H	-2.66405600	-3.51342100	-1.37884800
H	0.03372100	-4.64939100	-2.32051100
H	-1.02828500	-5.31711400	-1.05221500
H	-5.13160700	-1.26271400	-1.18652600
H	-2.03274500	-1.72826800	1.75994500
H	-6.04382400	-3.48006100	-0.58520900
H	-2.93401800	-3.95434700	2.35980600
H	-4.94568100	-4.85242400	1.18246900
H	-4.75145300	0.82905700	-0.75330500
C	-4.36010400	3.48146000	0.95607000
H	-3.13635800	2.41564500	-2.12648600
H	-2.06161200	2.35897800	-0.75027700
H	-3.69763300	4.06754300	1.62858900
H	-5.35900600	3.94346900	1.05227500
H	-4.41409000	2.45758400	1.35523500
H	-4.71055400	5.42459700	-0.95354800
H	-2.94541600	5.41589100	-0.84517000
H	-3.73302600	4.67975800	-2.25175600

PreTS II

E_{vacuum} = -26414.5600

E_{sol} = -26414.5754

$-TS$ = -0.1613

G_{sol} = -26413.8578

C	-6.22544300	1.72482900	-0.99320900
C	-5.32065400	1.63929300	0.07738100
C	-5.32086600	0.49109400	0.88735700
C	-6.21180000	-0.54501600	0.62573100
C	-7.10421000	-0.45693500	-0.44294700
C	-7.10757100	0.68043600	-1.25049600
C	-4.38576300	2.68724500	0.33210800
C	-3.54919800	3.53959800	0.55132900
C	-0.40925800	4.73556300	0.71981100
C	-0.01599200	4.26938900	2.07928500
C	-0.21171600	3.78756000	-0.43046500
C	1.24866000	3.61888200	-0.73673800
C	1.91028300	2.43628100	-0.72351500
C	3.38135000	2.36457100	-0.92007900
C	4.23708900	3.35070900	-0.40881600
C	5.61600800	3.25377400	-0.57958100

C	6.16452000	2.16681000	-1.26071400
C	5.32090800	1.17869700	-1.76847900
C	3.94274400	1.27329500	-1.59567200
O	1.29689900	1.26945200	-0.54714700
Sm	0.52817000	-0.62438800	-0.02270900
O	-1.25803200	-2.40991900	-0.41699900
C	-2.13095800	-3.00388000	0.54929700
C	-3.47068200	-3.03408600	-0.15560500
C	-3.08006600	-3.38855700	-1.59586700
C	-1.61171200	-2.94905900	-1.70507800
O	1.02268800	-2.68248200	1.39590100
C	1.25309200	-4.02372700	0.90980800
C	1.39453200	-4.89776200	2.14983800
C	0.65139300	-4.09576100	3.21415800
C	1.02652400	-2.67929700	2.83552600
O	-1.06237100	0.16655500	-1.77570700
C	-2.47193100	0.43574800	-1.60190300
C	-2.80423100	1.51552000	-2.61457100
C	-1.80624300	1.22645000	-3.73135700
C	-0.56451400	0.85952600	-2.94431900
O	2.38655200	-0.25705600	1.62505700
C	2.51020500	0.94303800	2.41807500
C	3.91908600	0.89934400	2.97761600
C	4.68455400	0.22030700	1.84582600
C	3.68969100	-0.82177900	1.36851800
I	-1.38437500	0.31786600	2.24734500
I	2.05232300	-2.15911000	-2.23749600
C	-0.44699200	6.19899400	0.44011300
H	-1.77349800	-4.02370000	0.78857200
H	-2.10109100	-2.37596600	1.44902700
H	-1.42647500	-2.15556400	-2.43846200
H	-0.93463400	-3.78727200	-1.93358500
H	-3.92963500	-2.03581000	-0.10502400
H	-4.16945900	-3.75467600	0.29195100
H	-3.71878800	-2.88113300	-2.33238300
H	-3.16974400	-4.46999000	-1.77473900
H	-3.02734900	-0.49495300	-1.80377600
H	-2.63884100	0.73311300	-0.55855100
H	-0.00959200	1.74897000	-2.60852300
H	0.12801200	0.17897200	-3.45895500
H	-2.62329900	2.50883200	-2.17697000
H	-3.85430800	1.46851600	-2.93254100
H	-1.63418000	2.08207100	-4.39870400
H	-2.14376300	0.37470300	-4.34440800
H	2.03749400	-2.41811400	3.19369500
H	0.31890100	-1.90732700	3.16647100
H	0.38787500	-4.30225300	0.28986200
H	2.14065500	-4.01343500	0.26301200
H	-0.43771200	-4.23555900	3.12296900
H	0.98837900	-5.90651200	1.99284300
H	2.45509300	-5.00208100	2.42819200
H	2.35614900	1.81345300	1.76165000
H	1.71172400	0.92332800	3.17197800
H	3.76952300	-1.76490500	1.93728300
H	3.76790400	-1.05031800	0.29527400
H	3.95334800	0.28955400	3.89524200
H	4.29852800	1.90231100	3.21630500
H	5.63424000	-0.23050700	2.16524500
H	4.89482100	0.93926500	1.04072500
H	3.81391900	4.18731600	0.15102500
H	3.29038200	0.48936000	-1.98480600
H	6.26823200	4.02586900	-0.16546100
H	5.73588600	0.32115200	-2.30270600
H	7.24610000	2.08791300	-1.38886400
H	1.82916200	4.52152500	-0.94230900
H	-0.63943700	2.80146400	-0.18692500
H	-0.74688700	4.17510100	-1.31808700
H	-0.46649600	4.89000500	2.87049600
H	-0.30641000	3.21972900	2.24543500
H	1.08481200	4.32217500	2.21974100
H	-0.87218600	6.76671200	1.28346000
H	0.57080600	6.61191000	0.26891500
H	-1.03192900	6.42869400	-0.46569700
H	-2.76556200	4.25276800	0.74992500
H	-4.60490800	0.42326700	1.70823400
H	-6.20864000	-1.43101000	1.26402000
H	-7.80069700	-1.27320700	-0.64446700
H	-7.80630400	0.75628700	-2.08597600
H	-6.22485800	2.61908700	-1.61835800
H	0.94601500	-4.35010700	4.24163400

TS II

E_{vacuum} = -26414.5548
E_{sol} = -26414.5721
$-TS$ = -0.1579
G_{sol} = -26413.8522

C	5.98340100	0.83360800	1.01492800
C	5.26993300	0.31784200	-0.08498700
C	4.86878500	-1.03322400	-0.05963200
C	5.17887500	-1.83484400	1.03293200
C	5.88765500	-1.31576900	2.11819900
C	6.28699700	0.02201900	2.10167100
C	4.98197700	1.13291100	-1.20957800
C	4.66285800	1.86493300	-2.13944900
C	2.71945500	3.27751500	-1.67116900
C	1.91386500	3.03550000	-2.90314600
C	2.24205200	2.63765300	-0.39851400
C	1.05303500	3.36786400	0.14846300
C	-0.19643600	2.85584800	0.27336100
C	-1.33345000	3.69855700	0.73090000
C	-1.38785100	5.07496400	0.46278400
C	-2.46478800	5.84327200	0.89406300
C	-3.51465200	5.25241400	1.59798400
C	-3.47588000	3.88456400	1.86137400
C	-2.40008600	3.11428300	1.42672300
O	-0.49065800	1.57917300	0.03467100
Sm	-0.81979000	-0.51090900	-0.07633200
O	-0.06436600	-2.78310100	0.84189700
C	0.52243900	-3.87148000	0.12119200
C	1.67693300	-4.29684600	1.00708300
C	1.11165700	-4.10352800	2.42406700
C	-0.11184100	-3.19710100	2.21676000
O	-2.03521100	-2.39977000	-1.29357300
C	-2.81707700	-3.45424800	-0.71337300
C	-2.72363600	-4.63777700	-1.68958300
C	-1.93535700	-4.07666700	-2.88264900
C	-2.09207900	-2.57881900	-2.71304500
O	0.62043300	-0.23352100	1.93199100
C	2.05269800	-0.41427400	1.83113500
C	2.65918900	0.60727100	2.77376500
C	1.57025200	0.75425500	3.83150100
C	0.31703100	0.71488200	2.98162400
O	-2.29745900	0.34404800	-1.88250000
C	-1.90935500	1.47532500	-2.68090700

C	-2.92107200	2.57666100	-2.35417800
C	-4.07127200	1.83589600	-1.64480400
C	-3.72985600	0.36798400	-1.84112100
I	1.37439500	-1.01820600	-2.19820700
I	-3.16854900	-0.78518100	1.90210800
C	3.45409900	4.57243900	-1.56478500
H	-0.22569300	-4.67930500	0.01333100
H	0.81514500	-3.49532500	-0.86674200
H	-0.11766400	-2.28623700	2.82396800
H	-1.05912900	-3.73579500	2.38707800
H	2.52949800	-3.62434400	0.82925900
H	2.00548400	-5.32601700	0.80754400
H	1.85034500	-3.64593300	3.09709000
H	0.81023200	-5.06046400	2.87346300
H	2.28697600	-1.44657500	2.13723100
H	2.35361800	-0.28650200	0.78254200
H	0.11300800	1.69342800	2.52076800
H	-0.58688600	0.35772300	3.49284600
H	2.80868500	1.56229000	2.24891900
H	3.62976700	0.27550400	3.16404700
H	1.64570700	1.68432100	4.41110800
H	1.59086200	-0.09422000	4.53516000
H	-3.07088600	-2.22456300	-3.08544800
H	-1.28613200	-1.98082500	-3.15498900
H	-2.40678600	-3.64689700	0.28458900
H	-3.85230500	-3.09784200	-0.58761200
H	-0.86889900	-4.33754000	-2.81013800
H	-2.22176900	-5.50570200	-1.23922700
H	-3.72908700	-4.96428400	-1.99179200
H	-0.87314600	1.71793000	-2.42110100
H	-1.95519100	1.16930400	-3.74029400
H	-4.11384900	-0.02092900	-2.80270100
H	-4.05098300	-0.28583300	-1.01981200
H	-3.25368000	3.08251700	-3.27160900
H	-2.48316300	3.33248900	-1.68944600
H	-5.06111400	2.08384100	-2.05221500
H	-4.07313600	2.07406100	-0.57193400
H	-0.58780700	5.54024000	-0.11608800
H	-2.38907200	2.04214300	1.62900500
H	-2.49185100	6.91109500	0.66583600
H	-4.29092100	3.40397400	2.40715700
H	-4.36191600	5.85550400	1.93086800
H	1.19417400	4.41460100	0.42762900
H	1.98004800	1.58761800	-0.59417200
H	3.07343500	2.64562400	0.32980800
H	2.44972400	3.35368200	-3.81285900
H	1.64707200	1.97188200	-3.00347400
H	0.96697400	3.61326400	-2.87387300
H	4.00620900	4.80739600	-2.48979400
H	2.75769500	5.41966900	-1.39452800
H	4.17008400	4.56477800	-0.72809500
H	4.66487800	2.34413500	-3.10133300
H	4.30585600	-1.43208400	-0.90489500
H	4.87082900	-2.88303800	1.03380300
H	6.13412200	-1.95233200	2.97023900
H	6.84424600	0.43651100	2.94446800
H	6.29822300	1.87809600	0.99768300
H	-2.30765600	-4.43310400	-3.85291200

Int III

E_{vacuum} = -26414.6125

E_{sol} = -26414.6267

$-TS$ = -0.1557

G_{sol} = -26413.9019

C	-6.24123900	0.69747500	-0.93262200
C	-5.30725400	0.58052700	0.14292000
C	-5.00888100	-0.73095700	0.62756100
C	-5.60157900	-1.84180000	0.05418000
C	-6.50827700	-1.70810900	-1.00659500
C	-6.82233700	-0.42893000	-1.48657500
C	-4.69412100	1.69023000	0.67911900
C	-4.20682300	2.74146400	1.27761100
C	-2.92511800	3.48889000	0.93875500
C	-2.02606300	3.47462600	2.17849400
C	-2.21567100	2.81139300	-0.25092900
C	-0.92101600	3.44691000	-0.63106800
C	0.26374000	2.79462000	-0.70795400
C	1.53059000	3.50971600	-1.01404700
C	1.77647400	4.80470500	-0.53631100
C	2.98537800	5.44356100	-0.80222400
C	3.97212200	4.79798900	-1.54772400
C	3.73802700	3.50785500	-2.02373900
C	2.53149500	2.86774900	-1.75453300
O	0.38479100	1.48247900	-0.52507600
Sm	0.76223900	-0.53207500	-0.00947500
O	0.09389500	-2.99107500	-0.38314800
C	-0.48962300	-3.87376100	0.59228200
C	-1.25985600	-4.89257400	-0.22087500
C	-0.34234400	-5.07156300	-1.42625000
C	0.16667200	-3.65800300	-1.66551700
O	2.21064500	-2.08189200	1.41432200
C	3.03090800	-3.16478100	0.92591500
C	3.81725500	-3.65357600	2.12965500
C	2.86216300	-3.34720900	3.27839000
C	2.29890400	-2.00696700	2.85229100
O	-0.96411100	-0.73499200	-1.77551200
C	-2.32952900	-1.13510400	-1.51616300
C	-3.17528100	-0.33773900	-2.48906800
C	-2.22472800	-0.16186700	-3.66819600
C	-0.91019500	0.09593600	-2.96046800
O	2.26054900	0.74092000	1.52700600
C	1.77103400	1.84213100	2.32885500
C	3.01209800	2.59092400	2.77813900
C	3.96531300	2.36070800	1.60986300
C	3.66300600	0.92277300	1.23620900
I	-1.27903900	-0.59491100	2.32454700
I	2.88066800	-1.04313500	-2.20930300
C	-3.31457200	4.93152000	0.59816000
H	0.31952300	-4.35274400	1.17342300
H	-1.09807400	-3.26361100	1.27238400
H	-0.46579100	-3.09337800	-2.36687100
H	1.20398200	-3.60440700	-2.02330800
H	-2.23273700	-4.47513400	-0.52742100
H	-1.44489800	-5.82216000	0.33492400
H	-0.84819100	-5.48312100	-2.31044600
H	0.49109100	-5.74531600	-1.17031400
H	-2.40866800	-2.22090300	-1.69823400
H	-2.55595800	-0.93664900	-0.46055500
H	-0.81396800	1.14464900	-2.63959400
H	-0.01217300	-0.19445300	-3.52245600
H	-3.42684700	0.63932900	-2.05155900
H	-4.11554000	-0.84897800	-2.73367600
H	-2.50279900	0.66582400	-4.33492400
H	-2.17004800	-1.08467500	-4.26890500

H	2.97450700	-1.17797600	3.12076900
H	1.29435700	-1.78483400	3.23703400
H	2.36268800	-3.94564600	0.52754600
H	3.64524800	-2.78241600	0.10003000
H	2.06022000	-4.10172000	3.32808100
H	4.08670500	-4.71549500	2.04650900
H	4.74749300	-3.07401100	2.24267300
H	1.11848600	2.46204700	1.69657500
H	1.17131400	1.41792300	3.14546100
H	4.23674900	0.20547000	1.85038900
H	3.82824800	0.69067900	0.17441300
H	3.41735300	2.15271700	3.70479000
H	2.80326400	3.65341300	2.96330100
H	5.02273000	2.50740200	1.86980500
H	3.71590900	3.02903200	0.77282900
H	1.01900500	5.30219300	0.07295300
H	2.36256400	1.85183000	-2.11504700
H	3.16372500	6.44821500	-0.41240000
H	4.50189500	2.98805100	-2.60624000
H	4.92227200	5.29677500	-1.75004400
H	-0.91537600	4.51194200	-0.87314300
H	-2.04056400	1.75461600	0.00181700
H	-2.92011700	2.82733100	-1.10478900
H	-2.53727000	3.93196700	3.04077600
H	-1.74746700	2.44410100	2.44495000
H	-1.10331500	4.04352600	1.98395800
H	-3.88563600	5.38774500	1.42241000
H	-2.42182000	5.55343300	0.43139800
H	-3.93847400	4.96984800	-0.30860500
H	-4.76600300	3.16283700	2.13121100
H	-4.29506200	-0.83419500	1.44647300
H	-5.35857900	-2.83475400	0.44005600
H	-6.97269200	-2.59076500	-1.44963000
H	-7.53460800	-0.31677100	-2.30711200
H	-6.48320500	1.69247800	-1.30934000
H	3.35121100	-3.30225800	4.26123300

TS III

E_{vacuum} = -26414.6081

E_{sol} = -26414.6240

$-TS$ = -0.1529

G_{sol} = -26413.8974

C	2.20542900	-2.68607100	1.23873500
C	2.74366400	-1.34558200	1.61779600
C	3.98932500	-1.47317600	-0.48043400
C	3.98961500	-2.78923000	-0.45502300
C	3.22647400	-3.60795600	0.55876800
C	1.95294100	-0.22395600	1.66549400
C	2.43809200	1.06274300	2.20218100
C	3.80294300	1.38895300	2.23664000
C	4.22820000	2.62618500	2.70877900
C	3.30084200	3.56480700	3.16457200
C	1.94270500	3.24714200	3.14518600
C	1.51468800	2.01288200	2.66467500
H	4.59933400	-3.34945200	-1.18340900
H	1.35222800	-2.53542200	0.56089300
O	0.70529400	-0.23089700	1.21318600
Sm	-1.06581300	0.05905700	0.08040000
O	0.27459800	2.01170400	-0.59600700
C	1.62754200	1.85280500	-1.09005500
C	2.32938700	3.14909800	-0.74728400
C	1.19371800	4.16160900	-0.86297600
C	0.01994300	3.39530300	-0.27816000
I	0.01070900	-1.58693000	-2.29682400
I	-2.53049200	1.72577600	2.21630100
O	-3.38444600	-0.89595300	-0.52193900
C	-3.77432300	-1.40385800	-1.81230500
C	-5.00530100	-2.24772500	-1.54820800
C	-5.66837700	-1.47607000	-0.41183700
C	-4.47309000	-1.03813100	0.41664000
O	-1.59774300	-2.00185200	1.36730700
C	-1.78729700	-3.31774400	0.80839500
C	-1.28180800	-4.28061200	1.86676400
C	-1.58499900	-3.52430100	3.15704500
C	-1.24916900	-2.09744400	2.76895200
O	-2.27995600	1.49440300	-1.66031600
C	-1.72573700	1.83937700	-2.94668200
C	-2.88477600	2.42288100	-3.73001100
C	-3.67218200	3.12784200	-2.63022500
C	-3.55671000	2.14164600	-1.48187800
H	-4.00337500	-0.54971300	-2.47380500
H	-2.91726500	-1.94608900	-2.23318400
H	-4.18193600	-1.79608400	1.16174500
H	-4.60274900	-0.08014100	0.93814500
H	-4.71362100	-3.25625000	-1.21305200
H	-5.63875100	-2.35492300	-2.43947100
H	-6.38220500	-2.07276900	0.17248800
H	-6.20519900	-0.59912600	-0.80867300
H	-2.86477600	-3.45713000	0.61341300
H	-1.24229300	-3.36751600	-0.14510000
H	-0.17589000	-1.87655900	2.86567900
H	-1.82193900	-1.32758100	3.30408600
H	-0.19737700	-4.42899900	1.75524500
H	-1.77301200	-5.26137900	1.80389900
H	-0.99220700	-3.86859200	4.01552100
H	-2.65177500	-3.61622000	3.41818500
H	-0.92380400	2.58087300	-2.79529600
H	-1.28667500	0.92842000	-3.37589600
H	-4.34390800	1.37064600	-1.52772600
H	-3.57287600	2.59540000	-0.48139700
H	-3.48779600	1.61872000	-4.18224400
H	-2.55058600	3.09260000	-4.53422800
H	-4.71754900	3.33687800	-2.89594100
H	-3.19026000	4.08347600	-2.36751000
H	2.06242800	0.96503300	-0.61728400
H	1.57234400	1.67947800	-2.17662900
H	-0.95698700	3.66705700	-0.70415400
H	-0.03691600	3.49256500	0.81608300
H	3.17248300	3.34335100	-1.42336100
H	2.71125800	3.11404300	0.28326100
H	1.00485500	4.41426800	-1.91966800
H	1.38364900	5.09538000	-0.31609800
H	4.53332000	0.67784600	1.85180300
H	0.45047800	1.77164000	2.65022300
H	5.29378000	2.86602100	2.70735400
H	1.20432000	3.96591100	3.50835500
H	3.63628900	4.53706200	3.53133800
H	3.69570300	-1.30306800	2.14942500
C	4.48063600	-0.37934600	-1.23885700
C	3.88236400	-0.04815900	-2.47813500
C	4.33824000	1.03560400	-3.21705500
C	5.38688800	1.82957200	-2.74440500
C	5.98629800	1.51618700	-1.52119200
C	5.54469900	0.43012700	-0.77831700

H	3.04987200	-0.65801800	-2.83412500
H	3.86978100	1.26500300	-4.17727100
H	5.73994800	2.68232400	-3.32766300
H	6.81323100	2.12420800	-1.14765400
H	6.02858400	0.17792900	0.16698000
H	1.82259000	-3.20675400	2.13850800
C	4.21599900	-4.18648100	1.57739300
C	2.49044400	-4.74985000	-0.14428900
H	3.19655600	-5.43599900	-0.63885600
H	1.90750100	-5.34087400	0.58186800
H	1.80039900	-4.35610600	-0.90652200
H	4.98711700	-4.79287900	1.07629300
H	4.73098200	-3.38866100	2.13362500
H	3.69578500	-4.83380800	2.30277800

TS III’

E_{vacuum} = -26414.6024

E_{sol} = -26414.6181

$-TS$ = -0.1547

G_{sol} = -26413.8934

C	3.33977000	4.64679300	-0.51772000
C	3.77804400	3.93163100	-1.63405600
C	3.38419700	2.61073900	-1.82278800
C	2.54648100	1.96786600	-0.89499900
C	2.10817400	2.70016800	0.21841300
C	2.50214500	4.02188300	0.40542700
C	2.10838800	0.56547200	-1.06019500
C	2.82630200	-0.33627600	-1.79618900
C	2.38495400	-1.73578000	-2.06903700
C	3.54775400	-2.73532300	-2.16319700
C	4.50721300	-2.46254600	-1.02209900
C	4.37241600	-1.43140900	-0.21617600
C	4.79384000	-0.73234300	0.93456700
O	1.01010500	0.21661600	-0.40239900
Sm	-1.05838900	0.01259800	0.04971100
O	-1.03622000	1.88817100	-1.57569300
C	-0.43521000	1.80263900	-2.88830800
C	-0.03776900	3.22514800	-3.23643800
C	-1.10137600	4.04124400	-2.50901000
C	-1.24158700	3.27026100	-1.21148400
I	-1.60652600	-1.92750600	-2.28731700
I	-1.19119200	1.95109900	2.43993000
O	-2.80113300	-1.37990300	1.33938500
C	-3.88957800	-2.13313700	0.76757600
C	-4.28459800	-3.12908400	1.83873700
C	-4.05180900	-2.31532600	3.10733500
C	-2.76970400	-1.57529400	2.77215300
O	0.01768000	-1.65177400	1.52725200
C	0.12995000	-3.05098300	1.18804300
C	1.42707700	-3.51774400	1.82799800
C	1.54123300	-2.58495400	3.02951800
C	1.09235800	-1.27424900	2.42247100
O	-3.40840700	0.88418700	-0.42274800
C	-3.96406100	1.08805400	-1.72598300
C	-5.41298200	0.67950000	-1.55751000
C	-5.74863600	1.20875000	-0.15684400
C	-4.38203700	1.32802500	0.53621000
C	3.00823400	-4.16363300	-2.06872200
H	-4.71862800	-1.44017000	0.54365800
H	-3.53486200	-2.57310600	-0.17385400
H	-1.87596600	-2.17107300	3.01368200
H	-2.66809400	-0.58901000	3.24594100
H	-3.61622800	-4.00503200	1.81241300
H	-5.31809100	-3.48296600	1.72114300
H	-3.95272000	-2.92569200	4.01547400
H	-4.88068600	-1.60576200	3.26307200
H	-0.75001700	-3.56965400	1.60660900
H	0.10331700	-3.15182600	0.09319800
H	1.88972300	-0.79956000	1.82905600
H	0.68303100	-0.54319600	3.13193400
H	2.27185800	-3.34707400	1.14134900
H	1.39968500	-4.58453200	2.08939600
H	2.55466000	-2.51910700	3.44409100
H	0.85468800	-2.89651000	3.83415900
H	-3.86778700	2.15383300	-2.00530600
H	-3.39365400	0.46590700	-2.42713600
H	-4.26361600	0.69541300	1.42423100
H	-4.14590000	2.36310300	0.82972000
H	-5.48412200	-0.41811200	-1.59566000
H	-6.06353900	1.09038100	-2.34172700
H	-6.43062800	0.53969600	0.38668200
H	-6.23517600	2.19323300	-0.21365700
H	0.40861800	1.10190200	-2.83653800
H	-1.19232700	1.39482700	-3.57815700
H	-2.22793300	3.34823100	-0.73390700
H	-0.47162600	3.55657500	-0.47865800
H	-0.01878100	3.39501500	-4.32172300
H	0.95916100	3.45099900	-2.82834600
H	-2.04996600	4.03406500	-3.07091100
H	-0.80909500	5.08597300	-2.33659900
H	3.71184300	2.07720100	-2.71691100
H	1.45648600	2.21933600	0.94867000
H	4.42388400	4.41218000	-2.37240200
H	2.14951600	4.56413800	1.28567000
H	3.64621000	5.68488000	-0.37374000
H	3.69536800	0.03992800	-2.33822700
C	4.30527000	-2.57231100	-3.48696500
H	1.79715500	-1.78756900	-3.00396000
H	1.69470300	-2.04286900	-1.27001600
H	5.17105100	-3.25212400	-3.53126200
H	3.64782500	-2.80442200	-4.34016800
H	4.68311100	-1.54636600	-3.61209600
H	3.81987400	-4.90455800	-2.14823300
H	2.49078600	-4.32634300	-1.11066100
H	2.28817400	-4.35966100	-2.87916400
C	5.14557400	0.63666800	0.87269600
C	5.46957700	1.33621300	2.02597600
C	5.45573700	0.70298200	3.27142400
C	5.12190900	-0.65141500	3.34963800
C	4.79142900	-1.36122100	2.20326800
H	5.13720800	1.14327700	-0.09228100
H	5.72349700	2.39571500	1.95391900
H	5.70193000	1.26169400	4.17612300
H	5.12200300	-1.15851300	4.31749900
H	4.53837600	-2.42166200	2.25920400
H	5.37660900	-3.13694500	-0.93496000

Int IV

E_{vacuum} = -26414.6681

E_{sol} = -26414.6844

$-TS$ = -0.1521

G_{sol} = -26413.9538

C	-2.47918900	2.50955700	1.37559900
C	-3.16395800	1.18281800	0.99752200

C	-3.51192100	1.43022300	-0.47081100
C	-3.44093800	2.73915400	-0.75512200
C	-3.04630700	3.58531200	0.42948300
C	-2.30812000	-0.02494900	1.27188100
C	-2.75400900	-1.17471700	1.99007100
C	-4.06400400	-1.30502300	2.53282100
C	-4.46131000	-2.46050300	3.18708700
C	-3.58240100	-3.53777800	3.34615200
C	-2.28148300	-3.42247400	2.84248300
C	-1.87284000	-2.27553300	2.18293700
H	-3.71206300	3.17947300	-1.71798000
H	-1.40709200	2.38687100	1.16563100
O	-1.07459200	0.00282700	0.79423400
Sm	0.91337700	-0.06778800	0.02683400
O	-0.05406100	-2.07636900	-1.02760600
C	-1.35809600	-2.01780900	-1.65719300
C	-1.94651800	-3.39608300	-1.45397500
C	-0.70772500	-4.27946500	-1.55896200
C	0.33641000	-3.45331700	-0.82891700
I	0.21066000	1.85727700	-2.25581500
I	2.16917300	-1.98260400	2.11203300
O	3.23552500	1.09445400	0.01124700
C	3.78121900	1.85564600	-1.08438600
C	4.95018900	2.61898100	-0.49391300
C	5.47037200	1.63159900	0.54509100
C	4.18373600	1.04600400	1.09943500
O	1.10245900	1.68653700	1.77093500
C	1.35086600	3.08953800	1.54977900
C	0.79836900	3.79661300	2.77526400
C	0.96761300	2.73864200	3.86219200
C	0.61827800	1.46592900	3.11714100
O	2.52808300	-1.12691000	-1.66577400
C	2.19186500	-1.35721300	-3.05030900
C	3.48531900	-1.80390600	-3.70347800
C	4.15723000	-2.56646900	-2.56634500
C	3.81882200	-1.69715400	-1.36972800
H	4.10768600	1.15421100	-1.87203500
H	2.97658100	2.47800500	-1.49636600
H	3.78060900	1.64591900	1.93217600
H	4.26239700	0.00508300	1.43999600
H	4.59862600	3.54320300	-0.00699100
H	5.69423200	2.89696400	-1.25303900
H	6.08715700	2.09398800	1.32794400
H	6.07308600	0.84743900	0.05869200
H	2.43944800	3.23392900	1.45133000
H	0.87069700	3.38092300	0.60471700
H	-0.46783100	1.29266100	3.06888200
H	1.10837200	0.55926600	3.49602600
H	-0.26707700	4.02990100	2.63284200
H	1.33033100	4.73472200	2.98541200
H	0.31812600	2.89978900	4.73327400
H	2.01159300	2.70572300	4.21390800
H	1.42040700	-2.14361800	-3.09380400
H	1.76856100	-0.42578100	-3.45035900
H	4.54432200	-0.87447100	-1.25326700
H	3.74164000	-2.23590100	-0.41513800
H	4.09238400	-0.93077100	-3.99294900
H	3.31079500	-2.41122400	-4.60219700
H	5.24082400	-2.69405800	-2.69572500
H	3.70742200	-3.56656300	-2.45596700
H	-1.92299000	-1.21045500	-1.18183200
H	-1.21533700	-1.77525300	-2.72335200
H	1.36006800	-3.57801800	-1.21115200
H	0.34721800	-3.65200400	0.25289800
H	-2.71966700	-3.62439000	-2.19842000
H	-2.39381700	-3.46739700	-0.45015300
H	-0.42557400	-4.42095900	-2.61532700
H	-0.83338900	-5.27123500	-1.10320900
H	-4.77938200	-0.48586200	2.44354800
H	-0.84889100	-2.19834500	1.82025500
H	-5.47659800	-2.52358400	3.58600800
H	-1.56856600	-4.24007600	2.97595000
H	-3.90252600	-4.44383600	3.86330500
H	-4.10539000	1.07779700	1.55982300
C	-3.93692400	0.37718400	-1.39943200
C	-3.63465100	0.47152900	-2.76764000
C	-4.04876800	-0.51331100	-3.65841800
C	-4.77035100	-1.61743900	-3.20224800
C	-5.06653100	-1.72842500	-1.84449100
C	-4.64840000	-0.74615000	-0.95064800
H	-3.03293000	1.31118700	-3.11909800
H	-3.79660700	-0.42321700	-4.71736500
H	-5.09473200	-2.39085400	-3.90192400
H	-5.62383600	-2.59078000	-1.47234700
H	-4.88129300	-0.85644700	0.10852900
H	-2.60483100	2.76274400	2.44003100
C	-4.29305100	4.25146400	1.02572700
C	-2.01269100	4.64851500	0.06620900
H	-2.43854600	5.39371500	-0.62511100
H	-1.67204400	5.19007600	0.96463100
H	-1.14163300	4.19165900	-0.42771100
H	-4.78009200	4.90972800	0.28884800
H	-5.03286900	3.50009100	1.34283600
H	-4.02744500	4.86498400	1.90255600

Int IV’

E_{vacuum} = -26414.6669

E_{sol} = -26414.6818

$–TS$ = -0.1521

G_{sol} = -26413.9512

C	-2.36697000	-5.29954300	0.63711200
C	-3.22780000	-4.79860300	-0.34670400
C	-3.20494300	-3.45799000	-0.70119800
C	-2.29199600	-2.55125100	-0.09396200
C	-1.44061800	-3.07763600	0.91476700
C	-1.48427700	-4.41625800	1.27006200
C	-2.20278900	-1.16991900	-0.45664100
C	-3.27739000	-0.46238100	-1.23681900
C	-2.77534100	0.08195800	-2.58855800
C	-3.61948900	1.33124200	-2.90291000
C	-4.06252700	1.73780500	-1.52128200
C	-3.89629000	0.77922600	-0.59218200
C	-4.37497600	0.83312800	0.79808400
O	-1.14704000	-0.46113300	-0.09471100
Sm	0.92650600	0.09680500	0.06895400
O	1.12424700	-2.11915800	-0.99937400
C	0.42225400	-2.47521100	-2.21356600
C	0.30313600	-3.98513900	-2.16998400
C	1.59030100	-4.38287200	-1.45382800
C	1.71937700	-3.29414600	-0.40436500
I	1.07621000	1.37859600	-2.73755200
I	1.43479300	-1.09390900	2.86199800
O	2.56504600	1.99044100	0.72421200
C	3.44258700	2.72419300	-0.15403800

C	3.94952400	3.89535300	0.66726700
C	3.97084000	3.30690800	2.07380000
C	2.70275900	2.47588100	2.07681400
O	-0.17958500	1.98833100	1.17201700
C	-0.38710100	3.25100100	0.50482200
C	-1.73373100	3.74157000	0.99904500
C	-1.77175400	3.18406600	2.41817000
C	-1.16468600	1.81008700	2.22296300
O	3.39401200	-0.54958800	-0.37815100
C	3.91948100	-0.92015400	-1.66945700
C	5.42511700	-0.82751600	-1.52449300
C	5.61953400	-1.26977800	-0.07819300
C	4.44136700	-0.60765200	0.61342000
C	-2.81677100	2.42109000	-3.60829900
H	4.26377300	2.05439700	-0.46139800
H	2.87390600	3.00105000	-1.05111600
H	1.81635700	3.08457800	2.32009000
H	2.72158000	1.60572800	2.74702500
H	3.23959800	4.73666900	0.61535700
H	4.92817100	4.25484700	0.32058500
H	3.97544400	4.06285000	2.87109100
H	4.85545100	2.66337100	2.20967600
H	0.43315100	3.93128200	0.79511500
H	-0.33752800	3.08166100	-0.58035700
H	-1.90496600	1.07732000	1.87153200
H	-0.64023400	1.40605000	3.09889700
H	-2.53514400	3.29187700	0.39367200
H	-1.82237700	4.83560900	0.94798500
H	-2.78505000	3.12109900	2.83502800
H	-1.14881300	3.79530600	3.09202800
H	3.60017100	-1.95109700	-1.89796700
H	3.48253700	-0.24206000	-2.41448700
H	4.67792100	0.42344000	0.92365900
H	4.05468500	-1.15075100	1.48616800
H	5.76108000	0.21352500	-1.65987700
H	5.95474100	-1.45444000	-2.25496600
H	6.58164700	-0.96384200	0.35531700
H	5.54557300	-2.36664400	-0.00091300
H	-0.54216700	-1.95200300	-2.21101100
H	1.02178200	-2.11913400	-3.06761200
H	2.75362300	-3.04491000	-0.12995000
H	1.15939200	-3.53595200	0.50970400
H	0.21034500	-4.42052300	-3.17442600
H	-0.57451100	-4.27991600	-1.57548500
H	2.44531000	-4.35911000	-2.14970700
H	1.54371800	-5.38210000	-0.99984200
H	-3.87683300	-3.11123800	-1.48787000
H	-0.76304400	-2.40098800	1.43669100
H	-3.92753300	-5.47031000	-0.84980700
H	-0.82407600	-4.77626200	2.06361400
H	-2.39484300	-6.35481400	0.91378300
H	-4.10443100	-1.17434400	-1.39242100
C	-4.85734300	0.97343200	-3.73707100
H	-2.81935100	-0.67435400	-3.38718100
H	-1.72454500	0.38321500	-2.46694600
H	-5.53359700	1.83772200	-3.83693900
H	-4.56188100	0.65761400	-4.75088300
H	-5.42742700	0.15373800	-3.27246600
H	-3.43645600	3.31539800	-3.78696200
H	-1.93920300	2.71459100	-3.01363600
H	-2.44869900	2.06646300	-4.58414300
C	-4.31476000	-0.29194900	1.63577400
C	-4.76340900	-0.23403600	2.95388100
C	-5.29433100	0.94480300	3.46886200
C	-5.38136200	2.06805500	2.64389500
C	-4.92769200	2.01214000	1.33203200
H	-3.90212000	-1.22682200	1.25856800
H	-4.69323600	-1.12547100	3.58024900
H	-5.64400300	0.99029100	4.50206400
H	-5.80642100	2.99813800	3.02811500
H	-5.00293200	2.90470100	0.70873700
H	-4.56605700	2.69368300	-1.35241400

Product

E_{vacuum} = -26414.6737

E_{sol} = -26414.6928

$-TS$ = -0.1545

G_{sol} = -26413.9635

C	-2.87153300	1.75920600	1.86157100
C	-3.42322800	0.64669300	0.94454400
C	-3.55112400	1.37527900	-0.38432800
C	-3.51570600	2.70203400	-0.19269000
C	-3.36152100	3.09030500	1.25609100
C	-2.49082700	-0.54218800	0.92146000
C	-2.86986400	-1.83749500	1.49299300
C	-4.17635400	-2.13957400	1.92505500
C	-4.47914200	-3.39523800	2.43798100
C	-3.48778300	-4.37240800	2.54186500
C	-2.18585300	-4.08258500	2.12717700
C	-1.87980300	-2.83451400	1.60547500
H	-3.65259800	3.44433600	-0.98191200
H	-1.77481100	1.72495700	1.79309900
O	-1.34500500	-0.38866700	0.44436500
Sm	0.92423100	0.01653700	-0.08975200
O	0.23039200	-1.56430900	-2.00464300
C	-1.09466700	-1.60906000	-2.54532200
C	-1.63192500	-2.98627700	-2.16365400
C	-0.35893100	-3.82473600	-1.92695900
C	0.77619400	-2.86087800	-2.25528900
I	0.28807500	2.79631200	-1.44866400
I	2.05391900	-2.66680500	1.31826500
O	3.13486300	1.11408300	0.78586900
C	3.77042700	2.23223800	0.13667900
C	4.93702400	2.61213000	1.03224900
C	5.31599600	1.26697200	1.64381200
C	3.95322800	0.63958800	1.86826400
O	0.59609000	0.94037400	2.28744500
C	0.86325000	2.29598400	2.68665200
C	0.28839900	2.42430900	4.08614600
C	0.47653900	1.01105300	4.63153000
C	0.14850600	0.16495000	3.41526900
O	2.82182400	-0.19587600	-1.83456500
C	2.64736100	0.15131500	-3.22390100
C	3.93444300	-0.27039000	-3.91683400
C	4.41747400	-1.41678800	-3.03269400
C	4.05792900	-0.90079800	-1.65336000
H	4.10572900	1.90471600	-0.86164300
H	3.01925700	3.02175500	-0.00287200
H	3.50938700	0.97537700	2.82330200
H	3.93776600	-0.45869400	1.85045600
H	4.60793800	3.30984200	1.81964600
H	5.75268800	3.09236800	0.47416300
H	5.89872900	1.34937100	2.57174800
H	5.89981800	0.66770400	0.92621800

H	1.95446900	2.45331900	2.67836400
H	0.41213300	2.96824500	1.94332800
H	-0.93803100	-0.00120700	3.31943600
H	0.66039200	-0.80723900	3.38003400
H	-0.78263400	2.67705800	4.03931700
H	0.79726000	3.19842700	4.67706000
H	-0.16967300	0.77917600	5.48922500
H	1.52321700	0.85216100	4.93794700
H	1.77117400	-0.40241700	-3.59637900
H	2.42847700	1.22689100	-3.28347300
H	4.82408400	-0.19646900	-1.27986300
H	3.89539200	-1.68040500	-0.89520500
H	4.66795500	0.55187400	-3.90591200
H	3.76732200	-0.55975500	-4.96352700
H	5.48937900	-1.63483900	-3.13698400
H	3.85478100	-2.33870600	-3.25079800
H	-1.65587000	-0.76534100	-2.13201900
H	-1.02821000	-1.48630800	-3.64106300
H	1.06854600	-2.91955800	-3.32134700
H	1.65924400	-2.98497300	-1.61603100
H	-2.27282700	-3.39284900	-2.95792700
H	-2.23645300	-2.92441900	-1.24834400
H	-0.31607900	-4.72888100	-2.55018700
H	-0.28318400	-4.13352600	-0.87448000
H	-4.96996200	-1.39505000	1.85512000
H	-0.85682900	-2.61412600	1.29555400
H	-5.49874100	-3.61392400	2.76112700
H	-1.39620400	-4.83167100	2.21386100
H	-3.72993300	-5.35613800	2.94893800
H	-4.42167100	0.33459800	1.28596800
C	-3.75804000	0.68155500	-1.65786100
C	-3.30609900	1.24840000	-2.86085200
C	-3.51202200	0.59676500	-4.07185400
C	-4.16378900	-0.63704500	-4.10841000
C	-4.60447500	-1.21592300	-2.91963700
C	-4.39934400	-0.56554200	-1.70528100
H	-2.74701500	2.18514500	-2.82837700
H	-3.14377900	1.04875400	-4.99519100
H	-4.31941400	-1.14957900	-5.05992600
H	-5.10920400	-2.18414100	-2.93481300
H	-4.74890700	-1.03410000	-0.78317200
H	-3.16617300	1.62879400	2.91416100
C	-4.72554900	3.49371100	1.83072700
C	-2.35428100	4.21949700	1.45663700
H	-2.73395800	5.16388700	1.03514700
H	-2.16430500	4.38819200	2.52977100
H	-1.40421900	3.98672600	0.95244200
H	-5.13834300	4.36080900	1.29170000
H	-5.45464300	2.67231200	1.74885900
H	-4.63421400	3.77018300	2.89395400

Product’

E_{vacuum} = -26414.6706
E_{sol} = -26414.6893
$-TS$ = -0.1555
G_{sol} = -26413.9615

C	-2.16847400	-5.27761400	0.91996700
C	-3.02285900	-4.89341300	-0.11576700
C	-3.08860500	-3.56255600	-0.51288100
C	-2.27833200	-2.59191200	0.10703900
C	-1.41915000	-2.99211900	1.14705300
C	-1.37337300	-4.31896500	1.55272600
C	-2.27482200	-1.18317300	-0.30749200
C	-3.41132400	-0.61331700	-1.12625000
C	-2.93019700	-0.20053900	-2.53334100
C	-3.79855500	1.00514600	-2.94561400
C	-4.20560000	1.54396400	-1.59931800
C	-4.01324200	0.67998900	-0.58755500
C	-4.44331800	0.84965300	0.80707300
O	-1.31866100	-0.44238300	-0.00224300
Sm	1.01313800	0.14722700	0.08520300
O	1.03404800	-2.20088600	-0.95750600
C	0.39208400	-2.58282700	-2.18376700
C	0.31723200	-4.09609200	-2.13242000
C	1.61950000	-4.44058700	-1.41620500
C	1.71757000	-3.33056100	-0.38173000
I	0.92517700	1.37576200	-2.85987600
I	1.55292200	-1.09851100	2.95867900
O	2.68471600	2.03646000	0.67589400
C	3.48614300	2.78463200	-0.25275000
C	3.84794000	4.06366200	0.47808900
C	3.97495800	3.56776900	1.91559900
C	2.83096400	2.57229300	2.00517800
O	-0.18364000	2.10515600	1.16924200
C	-0.40015700	3.34354300	0.47185600
C	-1.75712400	3.83090100	0.94294100
C	-1.78331800	3.32610600	2.38249100
C	-1.14725500	1.95700700	2.23379200
O	3.45559500	-0.57742300	-0.49163200
C	3.92803800	-0.91961700	-1.80468900
C	5.43714500	-0.78857700	-1.72850700
C	5.70861500	-1.23840500	-0.29593600
C	4.54043000	-0.61976600	0.45358800
C	-3.02294500	2.01890400	-3.78188300
H	4.38774800	2.19497600	-0.49946200
H	2.90022900	2.92186600	-1.17219600
H	1.88143100	3.05796200	2.28369100
H	3.00288700	1.73401500	2.69532300
H	3.02880300	4.79640800	0.39407700
H	4.76340900	4.52820900	0.08627700
H	3.89535300	4.36405100	2.66857300
H	4.94243800	3.05954200	2.05946700
H	0.40451000	4.04807200	0.75194600
H	-0.33578700	3.14842100	-0.60893100
H	-1.88606600	1.19998100	1.92860900
H	-0.61193800	1.59598800	3.12298800
H	-2.55116800	3.34351500	0.35586200
H	-1.86857600	4.92022100	0.85017200
H	-2.79315300	3.26318200	2.80799400
H	-1.16862900	3.97439000	3.02868600
H	3.62833400	-1.95870300	-2.03179700
H	3.43900000	-0.24761400	-2.52398700
H	4.76016400	0.41312500	0.77194900
H	4.20605700	-1.18539200	1.33490000
H	5.73653900	0.26320000	-1.86679300
H	5.95189700	-1.39290400	-2.48828100
H	6.67988100	-0.90765800	0.09737200
H	5.67169800	-2.33795100	-0.22842500
H	-0.58677400	-2.08686000	-2.22679100
H	0.99726100	-2.21840600	-3.03150900
H	2.74648300	-3.02441800	-0.14651700
H	1.21300700	-3.59309100	0.55983900
H	0.23184800	-4.54656000	-3.13106200
H	-0.54767200	-4.41246400	-1.52891000

H	2.46684100	-4.39490200	-2.11985000
H	1.61440300	-5.43667700	-0.95261600
H	-3.75315300	-3.28416300	-1.33196400
H	-0.79409200	-2.24903400	1.64784300
H	-3.64406100	-5.63797400	-0.61739800
H	-0.70999700	-4.60261700	2.37261900
H	-2.12757400	-6.32196900	1.23585300
H	-4.22141300	-1.35769800	-1.18476100
C	-5.05664600	0.55424000	-3.70099900
H	-2.98065800	-1.02933200	-3.25570500
H	-1.88176900	0.12792000	-2.46061900
H	-5.74145700	1.40034300	-3.87038500
H	-4.78645300	0.13810400	-4.68499700
H	-5.60859000	-0.21548100	-3.13874400
H	-3.65329400	2.88911700	-4.02745100
H	-2.12435000	2.36771200	-3.25262700
H	-2.68956900	1.56744700	-4.72956000
C	-4.37305500	-0.20973700	1.72444500
C	-4.77600800	-0.04771600	3.04824100
C	-5.27191600	1.17590400	3.48772800
C	-5.36709100	2.23603700	2.58398200
C	-4.95683400	2.07569100	1.26677000
H	-3.99804500	-1.18365400	1.40862100
H	-4.69847500	-0.88987100	3.73839700
H	-5.58578900	1.30512000	4.52511900
H	-5.76161000	3.20039600	2.91151900
H	-5.03193400	2.92102700	0.58106400
H	-4.70094700	2.51361800	-1.50533200

2,6-Dimethylphenyl Dimethylcyclopropyl Ketone Reactant

E_{vacuum} = -26184.9030
E_{sol} = -26184.9242
$-TS$ = -0.1507
G_{sol} = -26184.2567

C	5.41705800	-2.71598200	0.69587700
C	5.49533100	-1.88444600	-0.41788500
C	4.70074400	-0.73907800	-0.51150700
C	3.81126500	-0.45642800	0.53951400
C	3.71047500	-1.28852700	1.66836300
C	4.53127600	-2.41927800	1.72709300
C	2.95623300	0.77080300	0.47461300
C	3.62681500	2.06540800	0.66713600
C	3.40265300	3.26635500	-0.28474600
C	2.77530500	3.25895500	1.06051300
H	3.17337600	3.91117000	1.84035100
O	1.74185500	0.68200600	0.30628800
Sm	-0.67660200	-0.03359800	0.01365200
O	0.70412900	-2.15300000	-0.50779100
C	1.57063400	-2.32338500	-1.64180300
C	2.33609700	-3.60689800	-1.37445000
C	1.32171700	-4.41842700	-0.57424200
C	0.68768000	-3.34435400	0.29204500
I	-0.40082200	0.82670600	-3.06659800
I	-1.27030000	-1.02722800	3.01070900
O	-3.12757000	0.89351800	-0.16385800
C	-3.92715200	0.89662800	-1.35869500
C	-5.23112500	1.57291400	-0.97053100
C	-5.36311200	1.17969300	0.49826200
C	-3.92274100	1.27546900	0.96804300
O	-0.78560500	2.42164600	0.95459600
C	-1.14122300	3.49320400	0.06802400
C	-0.99708600	4.75526400	0.89726600
C	-1.44104500	4.26618500	2.27364000
C	-0.85139000	2.86455200	2.32328100
O	-2.27915800	-1.97528000	-0.69502900
C	-2.14936200	-2.67162600	-1.94355600
C	-3.49065600	-3.34577000	-2.16157200
C	-3.88568600	-3.69750500	-0.72996200
C	-3.41290000	-2.47199200	0.03514100
H	-4.08799600	-0.14974900	-1.67088100
H	-3.35762800	1.40258200	-2.15059700
H	-3.66695200	2.31376600	1.24746200
H	-3.66321100	0.61308900	1.80721300
H	-5.14091400	2.66734700	-1.06587000
H	-6.07478800	1.24681600	-1.59449300
H	-6.03762700	1.82934100	1.07315000
H	-5.72904400	0.14391700	0.58901800
H	-2.18068800	3.34348900	-0.27286000
H	-0.48558800	3.43970400	-0.81227900
H	0.17323400	2.86626100	2.73290700
H	-1.44235500	2.13656800	2.89693200
H	0.05629700	5.07845600	0.92291000
H	-1.60107200	5.58669400	0.50819900
H	-1.08873700	4.89641800	3.10188600
H	-2.54128100	4.22516600	2.32546900
H	-1.33999100	-3.41753600	-1.85347100
H	-1.86754400	-1.93672700	-2.71211600
H	-4.18535600	-1.68384100	0.05189900
H	-3.09834400	-2.66592000	1.07047500
H	-4.21098800	-2.63468400	-2.59833800
H	-3.42246200	-4.21569900	-2.82946100
H	-4.96049500	-3.88480200	-0.59865300
H	-3.34072800	-4.59478600	-0.39443800
H	2.20696700	-1.43267200	-1.72241100
H	0.94968800	-2.38178700	-2.55114700
H	-0.35329100	-3.54372900	0.58332200
H	1.27291700	-3.16530300	1.20931900
H	2.66300100	-4.09914700	-2.30099700
H	3.22415200	-3.39679200	-0.76024800
H	0.57157700	-4.87066500	-1.24392500
H	1.77458900	-5.22215900	0.02280100
H	6.18140900	-2.12346100	-1.23369900
H	4.47432400	-3.06827500	2.60395400
H	6.05019500	-3.60344400	0.75890500
H	4.63587300	1.99230800	1.07707400
C	4.62466300	4.12900800	-0.48631100
C	2.53928100	3.11143300	-1.50873100
H	1.70785500	3.03426500	1.10665200
H	2.84539700	0.09116000	3.09345600
H	2.99015300	-1.58262900	3.67558600
C	4.78478400	0.15058400	-1.72018700
H	5.25407300	1.11728500	-1.47679000
H	3.78913700	0.37206300	-2.13489000
H	5.38280200	-0.32115600	-2.51140200
C	2.77261400	-0.96568500	2.79365000
H	1.71761600	-1.13569600	2.52375700
H	2.09469200	4.08536200	-1.77006400
H	3.13234300	2.77998400	-2.37460600
H	1.72064600	2.39396600	-1.37451500
H	4.32419600	5.13864600	-0.80980300
H	5.21498700	4.23070400	0.43647100
H	5.27822100	3.71136300	-1.26888700

Int I

E_{vacuum} = -26184.8910

E_{sol} = -26184.9053

$-TS$ = -0.1459

G_{sol} = -26184.2341

C	5.40896500	-2.66033700	0.85118500
C	5.60978700	-1.72075100	-0.15647900
C	4.76523500	-0.61705500	-0.29645000
C	3.68075100	-0.44498800	0.60273500
C	3.49970600	-1.38416400	1.65109100
C	4.35792500	-2.48145500	1.74813700
C	2.79912400	0.72414000	0.48520200
C	3.39154900	2.07171700	0.63089000
C	3.29463700	3.23594900	-0.36746700
C	2.51350900	3.26799700	0.91053000
H	2.80837100	3.96112400	1.70242000
O	1.47695200	0.56858700	0.47089200
Sm	-0.53523800	-0.01596600	0.06402700
O	0.70751600	-2.01568700	-0.65617500
C	1.69007800	-1.99463300	-1.72031300
C	2.53446600	-3.23632700	-1.50817700
C	1.54726100	-4.19731300	-0.85295300
C	0.77145200	-3.26630800	0.05816600
I	-0.50312100	0.92117200	-2.87190200
I	-1.39193100	-1.16279000	2.80829900
O	-3.05255100	0.75846500	-0.11261900
C	-3.85495400	0.81328100	-1.31103300
C	-5.17598800	1.43974700	-0.89610100
C	-5.28348600	1.02595800	0.56754200
C	-3.84390100	1.15751000	1.02171800
O	-0.94653100	2.26932500	0.99250900
C	-1.25949300	3.41454600	0.17226600
C	-1.03967300	4.62190000	1.06679100
C	-1.36498500	4.06392800	2.44986000
C	-0.78738900	2.66281400	2.37413500
O	-2.09144300	-1.92240100	-0.79860100
C	-1.91018600	-2.57215300	-2.07092000
C	-3.26257200	-3.16872100	-2.39937000
C	-3.73635700	-3.60949000	-1.01850200
C	-3.24415000	-2.47777100	-0.12956300
H	-3.98453200	-0.21699800	-1.68029500
H	-3.30010400	1.37745900	-2.07173200
H	-3.60268100	2.20494400	1.27476000
H	-3.56389300	0.51386200	1.86667500
H	-5.12467000	2.53755500	-0.97783700
H	-6.01299100	1.09433400	-1.51850700
H	-5.96785700	1.65236700	1.15627400
H	-5.61987800	-0.02014100	0.65369600
H	-2.30846400	3.32300500	-0.15576400
H	-0.61602100	3.38585500	-0.71812500
H	0.28719100	2.64084900	2.61622700
H	-1.30241100	1.91743200	2.99479900
H	0.01148100	4.94495300	1.02098600
H	-1.67254500	5.47163100	0.77621100
H	-0.92688100	4.64777300	3.27078100
H	-2.45552800	4.02412600	2.60471300
H	-1.14407000	-3.35931600	-1.96677400
H	-1.55372300	-1.81434600	-2.78169000
H	-3.99246200	-1.67629100	-0.02852200
H	-2.94225200	-2.78928600	0.87911100
H	-3.93065200	-2.39743200	-2.81602100
H	-3.19407600	-3.98989500	-3.12619500
H	-4.82360100	-3.75061900	-0.94566400
H	-3.25297500	-4.55888000	-0.73766100
H	2.25135000	-1.05515600	-1.64259500
H	1.14353900	-2.00593800	-2.67673900
H	-0.25962800	-3.58023700	0.27275700
H	1.30010300	-3.10139000	1.00943600
H	2.95160800	-3.61454500	-2.45154200
H	3.36545600	-3.01793900	-0.82302800
H	0.88115300	-4.65220400	-1.60480200
H	2.03609900	-5.00587900	-0.29243900
H	6.43026800	-1.85091500	-0.86710400
H	4.21818000	-3.18732500	2.57147600
H	6.07528900	-3.52030300	0.94501800
H	4.34805700	2.05256500	1.16334500
C	4.50243700	4.13423800	-0.46183800
C	2.54402600	3.04799700	-1.65647000
H	1.44715200	3.06040900	0.83156900
H	2.47288700	-0.10587700	3.04225300
H	2.66014300	-1.79608700	3.58852300
C	4.98967900	0.34100100	-1.43016100
H	5.40079100	1.30206100	-1.08362700
H	4.04390700	0.57881100	-1.94096500
H	5.69136700	-0.07870000	-2.16485800
C	2.47084300	-1.15691100	2.71468400
H	1.44447600	-1.35640700	2.37568700
H	2.08690800	3.99675100	-1.98534100
H	3.21391300	2.71130200	-2.46385300
H	1.74439100	2.30273100	-1.56226600
H	4.21173500	5.15001900	-0.77985200
H	5.02057600	4.22185900	0.50561100
H	5.22667900	3.75159600	-1.20009500

TS I

E_{vacuum} = -26184.8742

E_{sol} = -26184.8885

$-TS$ = -0.1465

G_{sol} = -26184.2198

C	5.31114300	-2.84504200	0.70917900
C	5.48461900	-1.89580700	-0.29272000
C	4.67317400	-0.75802900	-0.35996000
C	3.66625700	-0.56742600	0.61385900
C	3.50755600	-1.51766800	1.64858100
C	4.32535600	-2.65039300	1.67297000
C	2.80022600	0.63958200	0.59146800
C	3.36446600	1.90171900	0.78544900
C	3.43634400	3.32370300	-0.52871200
C	2.62180800	3.19192000	0.70290400
H	2.80390400	3.91978600	1.50627500
O	1.48167000	0.46433100	0.43150000
Sm	-0.54752600	-0.01496600	0.04627000
O	0.59220500	-2.00336100	-0.86096400
C	1.60880600	-1.92086900	-1.88750100
C	2.41005600	-3.20416300	-1.76354000
C	1.38435300	-4.17963100	-1.19553300
C	0.63798400	-3.29371800	-0.21973100
I	-0.59071800	1.26107400	-2.74613900
I	-1.36855100	-1.56790000	2.60630300
O	-3.02298700	0.87476500	0.14548000
C	-3.87256800	1.13765300	-0.99234800
C	-5.16276100	1.70372800	-0.42348800
C	-5.21957300	1.04992600	0.95256200
C	-3.76172900	1.09031700	1.36361400

O	-0.80215700	2.08876300	1.33939300
C	-1.17794300	3.37344800	0.80213300
C	-0.75341400	4.39519500	1.84745200
C	-0.76971200	3.57962200	3.13844700
C	-0.25192100	2.23623800	2.66585400
O	-2.19948700	-1.69414700	-1.02948300
C	-2.03158500	-2.29197400	-2.31675300
C	-3.44456600	-2.36660100	-2.86682400
C	-4.29504100	-2.63497200	-1.61277800
C	-3.34948000	-2.31667500	-0.44502300
H	-4.03551200	0.18514900	-1.51949000
H	-3.33707300	1.81196700	-1.67310800
H	-3.49067600	2.07763500	1.77718600
H	-3.46015600	0.31459000	2.08024000
H	-5.09284200	2.79877700	-0.32124700
H	-6.03033600	1.47903500	-1.05923300
H	-5.87142000	1.57255600	1.66614000
H	-5.56762000	0.00688800	0.87310000
H	-2.26808700	3.36495300	0.64361500
H	-0.68957000	3.50181100	-0.17516200
H	0.84739600	2.20971000	2.59432400
H	-0.59344400	1.37522900	3.25642100
H	0.26518300	4.75550400	1.63986300
H	-1.42467700	5.26466600	1.86948000
H	-0.14599500	4.01093500	3.93320800
H	-1.79711900	3.47920500	3.52435000
H	-1.58437900	-3.29641200	-2.19793100
H	-1.35698800	-1.64904000	-2.89508400
H	-3.75083600	-1.61993200	0.29890000
H	-3.02814700	-3.22707700	0.08788100
H	-3.70608900	-1.40024800	-3.32336600
H	-3.55341900	-3.14305000	-3.63641800
H	-5.19590100	-2.00497000	-1.59269100
H	-4.62960600	-3.68075300	-1.56295100
H	2.19791300	-1.01284800	-1.71000900
H	1.09408700	-1.82973200	-2.85751200
H	-0.39656300	-3.59606900	-0.00855200
H	1.17921300	-3.19581300	0.73488800
H	2.83173800	-3.52077700	-2.72734100
H	3.23661100	-3.06568100	-1.05201300
H	0.70623100	-4.54466000	-1.98460500
H	1.83852400	-5.04837100	-0.69973500
H	6.25576300	-2.04099100	-1.05369200
H	4.20312200	-3.37341600	2.48367100
H	5.94943900	-3.73056600	0.74487900
H	4.38906400	1.93278000	1.15989300
C	4.71257100	4.09484100	-0.46997600
C	2.81637900	3.04879000	-1.85302900
H	1.55351200	3.04184700	0.51967700
H	2.56569800	-0.23711400	3.09227800
H	2.73277700	-1.93917600	3.61262100
C	4.87223300	0.21509500	-1.48635400
H	5.32162000	1.15814200	-1.13980700
H	3.91509600	0.49219300	-1.95199100
H	5.52700400	-0.21320200	-2.25862700
C	2.52543900	-1.28449400	2.75467000
H	1.48602800	-1.46437800	2.44355200
H	2.26101300	3.93153800	-2.22890200
H	3.57146600	2.79717000	-2.61477200
H	2.09019400	2.22428000	-1.80285900
H	4.53853900	5.17082900	-0.67697100
H	5.18477400	4.03632600	0.52379700
H	5.44358500	3.74176800	-1.21622200

Int II

E_{vacuum} = -26184.8922
E_{sol} = -26184.9071
$-TS$ = -0.1483
G_{sol} = -26184.2392

C	5.45204500	-2.71554900	0.72128300
C	5.51671700	-1.81839600	-0.33767200
C	4.65741200	-0.71433100	-0.40315600
C	3.71728300	-0.51851500	0.62782000
C	3.65508300	-1.41973800	1.71187000
C	4.52561300	-2.51155200	1.74045400
C	2.75701800	0.62805300	0.58513200
C	3.19325000	1.90519100	0.64560800
C	2.69340800	4.17701800	-0.38209000
C	2.31317100	3.11204000	0.60309800
H	2.26995300	3.58095900	1.60756100
O	1.46817800	0.28767400	0.49657600
Sm	-0.55369000	-0.11907800	0.02965600
O	0.55035300	-2.02074900	-1.10699400
C	1.67493800	-1.83323200	-1.99545200
C	2.49631500	-3.09877900	-1.85913900
C	1.41739300	-4.14699900	-1.61079300
C	0.47354500	-3.40011400	-0.68782200
I	-0.34034700	1.67106300	-2.45491900
I	-1.25540500	-2.00290600	2.38879900
O	-3.01390000	0.70073800	0.20796000
C	-3.77495000	1.27728000	-0.87113100
C	-4.99129000	1.89987300	-0.21323300
C	-5.23872600	0.94465900	0.94963800
C	-3.82531100	0.61850000	1.39835200
O	-0.83086400	1.77517400	1.61139900
C	-1.34204500	3.08943800	1.30785700
C	-0.81601900	3.99855500	2.40699100
C	-0.63869700	3.03301000	3.57628100
C	-0.13585500	1.78546800	2.87828100
O	-2.20853000	-1.54058100	-1.36002200
C	-2.02926300	-1.83736700	-2.75983600
C	-3.32615600	-2.49039100	-3.19627600
C	-3.72694400	-3.24531600	-1.93313300
C	-3.35390300	-2.25067100	-0.84936100
H	-4.05612000	0.47154600	-1.57143100
H	-3.12471500	1.98132400	-1.40680200
H	-3.44315600	1.35272800	2.12728100
H	-3.69976800	-0.38324500	1.83044400
H	-4.75235800	2.90886100	0.16041700
H	-5.84128700	1.98675500	-0.90394400
H	-5.84231100	1.37777700	1.75905600
H	-5.75084000	0.03522000	0.59536700
H	-2.44245900	3.03424900	1.31332300
H	-1.00865100	3.36727400	0.29709300
H	0.94666800	1.82174500	2.68034900
H	-0.37767000	0.84344000	3.38905600
H	0.15329000	4.42786400	2.11536000
H	-1.50625400	4.82595000	2.62201300
H	0.06538000	3.39575000	4.33751100
H	-1.60464900	2.83465500	4.06850500
H	-1.17484400	-2.52630900	-2.86319200
H	-1.78752500	-0.89635400	-3.27294800
H	-4.16535300	-1.52392500	-0.67625400
H	-3.07484400	-2.70046100	0.11361400

H	-4.07991600	-1.72513300	-3.44337400
H	-3.19381900	-3.13547700	-4.07570900
H	-4.78972200	-3.52177500	-1.89789600
H	-3.13135200	-4.16718700	-1.83225300
H	2.19702000	-0.92231000	-1.68368700
H	1.28246800	-1.69124700	-3.01661800
H	-0.57744600	-3.71556200	-0.75464200
H	0.78634800	-3.45966700	0.36582000
H	3.10831800	-3.29560000	-2.74993000
H	3.16567200	-3.02033500	-0.98962800
H	0.91060700	-4.41674200	-2.55221100
H	1.79872200	-5.06827900	-1.14970600
H	6.24081200	-1.97450700	-1.14140500
H	4.48246500	-3.20175000	2.58668800
H	6.12775700	-3.57296600	0.75667600
H	4.27149300	2.05948000	0.74165600
C	1.99433700	5.48638400	-0.25086400
C	3.23771900	3.79360300	-1.71313200
H	1.28147600	2.76913000	0.38583900
H	2.68892100	-0.13469100	3.13991500
H	2.98071100	-1.79793800	3.72030400
C	4.76801500	0.21550000	-1.57997500
H	5.43815200	1.06203800	-1.35841100
H	3.79929600	0.65222400	-1.85819500
H	5.18553900	-0.31134600	-2.45066900
C	2.69956100	-1.19534700	2.84544800
H	1.66716600	-1.45831300	2.56987900
H	2.44356000	3.36896500	-2.36289200
H	3.66428900	4.66140700	-2.24093700
H	4.01367600	3.01868500	-1.62913600
H	0.95372500	5.43266500	-0.63982300
H	1.92190400	5.80982600	0.80160300
H	2.49943500	6.28338000	-0.81956400

PreTS II

E_{vacuum} = -26493.1546

E_{sol} = -26493.1696

$-TS$ = -0.1682

G_{sol} = -26492.4002

C	3.27432000	-2.10279000	-1.21027500
O	2.21561300	-1.16192800	-1.47239800
C	2.67565400	-0.15917000	-2.41162100
C	4.11602900	-0.52530000	-2.73989700
C	4.54105000	-1.33606900	-1.51971400
Sm	0.29793500	-0.70618200	0.04074300
I	1.48308300	-2.58115800	2.21895500
O	1.47927100	1.01463600	0.42090600
C	2.21487400	2.11832300	0.55727300
C	3.69578100	1.91446200	0.47254100
C	4.42080000	2.40268400	-0.63184300
C	5.79426500	2.13928600	-0.71044700
C	6.44502600	1.42750700	0.29013100
C	5.72786400	0.98043500	1.39702200
C	4.35543800	1.21535600	1.50440000
C	1.70552600	3.35340300	0.75704000
C	0.24569900	3.71624500	0.75319400
C	-0.02668600	4.89245500	-0.13998100
C	0.17062300	6.26415200	0.40891400
C	-3.24784100	4.01053600	-0.14541700
C	-4.10263400	3.14845700	-0.17388500
C	-5.06615200	2.09610600	-0.21355000
C	-5.96626700	1.90494300	0.84755600
C	-6.89059500	0.86587000	0.80419900
C	-6.93160100	0.00834400	-0.29533900
C	-6.03746800	0.19094300	-1.35067400
C	-5.10692500	1.22407000	-1.31447700
C	0.04087300	4.69614900	-1.61548300
O	-1.94936300	-1.89293600	0.52804100
C	-3.05897300	-1.98464800	-0.38794200
C	-4.24361000	-2.39941700	0.46211400
C	-3.57996600	-3.28259300	1.51259900
C	-2.28074800	-2.54201600	1.77400100
O	-0.02153700	-2.86103800	-1.34260300
C	-0.25823500	-4.17809600	-0.81055100
C	0.08534700	-5.12947400	-1.93973100
C	-0.35317300	-4.32402400	-3.15818600
C	0.09548700	-2.92467000	-2.77982900
O	-0.98191000	0.36101900	1.86167300
C	-2.29978300	0.93583900	1.71255700
C	-2.41949400	1.97527600	2.81337300
C	-1.46798500	1.43941700	3.87973000
C	-0.33334500	0.89202000	3.03738000
I	-1.15921700	0.71369200	-2.26724600
H	-2.81592400	-2.74055200	-1.15539100
H	-3.17208300	-1.01279200	-0.88468500
H	-2.39480400	-1.76389100	2.54664800
H	-1.43660200	-3.18284400	2.06107600
H	-4.70669500	-1.51470500	0.92685500
H	-5.01794900	-2.91256200	-0.12463200
H	-4.17636600	-3.40809100	2.42679600
H	-3.37662900	-4.28446700	1.10037900
H	-3.03881400	0.12785700	1.82968100
H	-2.38718600	1.35304700	0.70082100
H	0.37135400	1.67760100	2.72248500
H	0.22811100	0.06855800	3.49949600
H	-2.08096800	2.95492100	2.44686800
H	-3.45587500	2.08446600	3.16063300
H	-1.12659500	2.20809800	4.58664200
H	-1.94332800	0.62919000	4.45633200
H	1.14980400	-2.74875700	-3.04597800
H	-0.51377000	-2.11481400	-3.20438000
H	-1.32254600	-4.25863800	-0.52993400
H	0.35566500	-4.29180900	0.09318100
H	-1.44912900	-4.36290200	-3.26874500
H	-0.42287000	-6.09855900	-1.84049800
H	1.17171700	-5.31206300	-1.96910700
H	2.59399200	0.81016400	-1.90647200
H	2.00133700	-0.16546000	-3.28006400
H	3.14759300	-2.97503000	-1.87924000
H	3.17763100	-2.43331200	-0.16760900
H	4.16362600	-1.14350900	-3.65089600
H	4.73573500	0.36766300	-2.89642800
H	5.39775400	-1.99720900	-1.71023700
H	4.79109600	-0.66717700	-0.68238100
C	3.77490100	3.21997700	-1.71746100
C	3.60522000	0.76156300	2.71958600
H	6.35713700	2.50684700	-1.57252600
H	6.23905400	0.44422500	2.20025300
H	7.51659100	1.23017400	0.21448200
H	2.42620800	4.16701500	0.87947900
H	-0.33062500	2.83977700	0.41388600
H	-0.08930600	3.96310100	1.77924600
H	-0.56605600	5.44365600	-2.15379600
H	-0.29771600	3.69006100	-1.90761300

H	1.07882300	4.80900400	-1.99223300
H	-0.32686700	7.02930500	-0.20917600
H	1.24605400	6.54330600	0.44131000
H	-0.20438300	6.35185400	1.44196800
H	-2.43848000	4.72197500	-0.12683400
H	-4.39201300	1.36337100	-2.12635800
H	-6.06288600	-0.47910300	-2.21252000
H	-7.66154000	-0.80296900	-0.32928500
H	-7.58860700	0.72805700	1.63235200
H	-5.93409300	2.58513000	1.70019100
H	0.09629000	-4.66419900	-4.10129000
H	4.29535800	0.46549200	3.52154500
H	2.95809900	-0.10001500	2.49620700
H	2.95452400	1.56646300	3.09464900
H	4.33535300	3.13047800	-2.65996800
H	3.75895400	4.28837500	-1.44640400
H	2.72958900	2.93384900	-1.89419300

TS II

E_{vacuum} = -26493.1449

E_{sol} = -26493.1622

$-TS$ = -0.1645

G_{sol} = -26492.3903

C	3.77969600	-1.17922100	-1.00531400
O	2.49254600	-0.63398100	-1.35319200
C	2.66098300	0.43453300	-2.31804100
C	4.15772000	0.53396500	-2.57348500
C	4.75307200	-0.05973700	-1.30093200
Sm	0.42267600	-0.75406800	0.02388900
I	2.00478000	-2.19225400	2.28627800
O	0.98957800	1.25503100	0.39445400
C	1.33320000	2.53056900	0.56231600
C	2.80578300	2.80348600	0.51981900
C	3.36856000	3.48500300	-0.57636300
C	4.75503700	3.67863300	-0.61503300
C	5.56939600	3.22694500	0.41678100
C	5.00157900	2.58001200	1.51137500
C	3.62403200	2.35865800	1.57806700
C	0.46169900	3.54367400	0.76085800
C	-1.03974900	3.46097400	0.72923600
C	-1.61395000	4.47810300	-0.21991300
C	-1.49447300	5.91433900	0.17644600
C	-3.98044400	4.28860000	0.05455800
C	-4.50455800	3.17765000	-0.01426800
C	-4.98823700	1.84876100	-0.12691800
C	-5.74311500	1.26697000	0.91128300
C	-6.20150400	-0.04022500	0.80130800
C	-5.92537800	-0.79054500	-0.34412700
C	-5.18544900	-0.22008800	-1.38095500
C	-4.72123800	1.08598700	-1.27989800
C	-1.54829500	4.17444000	-1.67916400
O	-1.43027800	-2.49401500	0.44207400
C	-2.45256600	-2.86255800	-0.50624800
C	-3.48947700	-3.61963500	0.30004800
C	-2.62836900	-4.30950900	1.35212600
C	-1.59544200	-3.24222700	1.66591000
O	0.82079200	-2.92923600	-1.30330000
C	0.86472500	-4.25848700	-0.75075400
C	1.55184300	-5.10524300	-1.80389900
C	1.06113700	-4.44624800	-3.08846700
C	1.11159100	-2.97636800	-2.71682500
O	-1.13944900	-0.08381800	1.82780400
C	-2.56652900	0.10153800	1.69604800
C	-2.97399500	1.01239400	2.84505200
C	-1.87221100	0.77269000	3.87337600
C	-0.65189900	0.61815800	2.99163700
I	-1.18794400	0.10312900	-2.45231200
H	-1.99177200	-3.49203000	-1.28735900
H	-2.82411900	-1.94496900	-0.98004500
H	-1.93974500	-2.54730400	2.44930800
H	-0.61345300	-3.63013800	1.96718100
H	-4.18981000	-2.91282600	0.77313800
H	-4.07335800	-4.31438400	-0.31962900
H	-3.18589600	-4.62163900	2.24591700
H	-2.14353700	-5.20296600	0.92621000
H	-3.04418900	-0.88736000	1.76629800
H	-2.78047500	0.52118800	0.70387300
H	-0.24237700	1.58895300	2.67148600
H	0.15372400	0.00813600	3.42182700
H	-2.97826700	2.06180200	2.51536100
H	-3.97734800	0.76990800	3.21787200
H	-1.76334700	1.59472300	4.59409800
H	-2.05762200	-0.15641300	4.43651900
H	2.11542600	-2.55200100	-2.87539500
H	0.37513600	-2.34485500	-3.23243500
H	-0.16958500	-4.60155800	-0.57731300
H	1.39066400	-4.20625000	0.21234800
H	0.02603100	-4.75534500	-3.30708000
H	1.29084300	-6.16937500	-1.72132000
H	2.64622400	-5.01120800	-1.71610100
H	2.25193800	1.34235700	-1.85950000
H	2.06664400	0.18922500	-3.20959100
H	3.97105400	-2.06761000	-1.63661400
H	3.73702500	-1.49126800	0.04629900
H	4.44456800	-0.06650900	-3.45186200
H	4.47074400	1.57205000	-2.74796200
H	5.78371600	-0.41972100	-1.42578900
H	4.73422100	0.68088800	-0.48735500
C	2.52412600	4.03049600	-1.69563000
C	3.02586000	1.68518500	2.77578900
H	5.19541900	4.19834300	-1.47002100
H	5.63404200	2.24224900	2.33596900
H	6.64893800	3.38688600	0.37348000
H	0.90371700	4.53196000	0.91544600
H	-1.34385500	2.44866400	0.42347300
H	-1.45316900	3.64816800	1.73861600
H	-2.23870300	4.80981700	-2.25677500
H	-1.77826900	3.11885100	-1.88546600
H	-0.52919200	4.36238300	-2.07234800
H	-2.15680600	6.56019400	-0.42457200
H	-0.46631500	6.29216500	0.00971700
H	-1.72626800	6.06914700	1.24267400
H	-3.96846900	5.36400100	0.10297300
H	-4.12607000	1.52210800	-2.08171900
H	-4.95711800	-0.79710900	-2.27915000
H	-6.29559000	-1.81401300	-0.43178000
H	-6.78777400	-0.47657900	1.61294800
H	-5.96349300	1.85965300	1.80026600
H	1.67804600	-4.67404700	-3.96865200
H	3.75460700	1.61578000	3.59498200
H	2.68749100	0.66499700	2.53836100
H	2.14678600	2.24247600	3.13389700
H	3.11940200	4.14541800	-2.61353200
H	2.12224000	5.02388400	-1.43751400

H	1.65464100	3.39507000	-1.91245800

Int III

E_{vacuum} = -26493.2047

E_{sol} = -26493.2194

$-TS$ = -0.1614

G_{sol} = -26492.4412

C	-5.61369400	1.83572500	0.97498600
C	-4.76337200	1.87828700	-0.17092500
C	-4.80200200	0.76903300	-1.07146400
C	-5.62064800	-0.31595100	-0.81314000
C	-6.43388500	-0.34904100	0.32844000
C	-6.42601600	0.74007200	1.21023200
C	-3.88620100	2.92219300	-0.38022700
C	-3.23021600	3.99517100	-0.72669000
C	-1.77250700	4.34488200	-0.46579700
C	-1.08268800	4.41513100	-1.83244200
C	-1.10365100	3.28445700	0.43064700
C	0.36869900	3.47680000	0.61826200
C	1.29943300	2.49736700	0.57147900
C	2.76314900	2.80243700	0.68013800
C	3.42682400	3.50544500	-0.34491000
C	4.81010700	3.70444800	-0.24874100
C	5.52800700	3.23486000	0.84364000
C	4.86241700	2.56488800	1.86639500
C	3.48610200	2.33898500	1.80002700
O	1.01138100	1.20446100	0.41847000
Sm	0.43134600	-0.78594100	-0.00817800
O	-1.46229300	-2.51103500	0.30664000
C	-2.49931300	-2.79375600	-0.65434100
C	-3.57139200	-3.53135300	0.12319900
C	-2.74101400	-4.31218800	1.13665300
C	-1.64524000	-3.32057000	1.48802900
O	0.82738500	-2.90301100	-1.40614400
C	0.81599500	-4.25412700	-0.90804300
C	1.52253100	-5.07511000	-1.96869500
C	1.09787800	-4.35189100	-3.24230100
C	1.17159300	-2.90015900	-2.80867300
O	-1.14221400	-0.17130700	1.81168000
C	-2.56925400	0.01125500	1.67868200
C	-2.98981400	0.87916400	2.85528500
C	-1.88616000	0.62078700	3.87605500
C	-0.66781900	0.52153200	2.98482000
O	2.53962600	-0.63014400	-1.33350900
C	2.73497000	0.48000200	-2.24472400
C	4.24206500	0.64605400	-2.38661900
C	4.77285900	0.00092900	-1.11046700
C	3.82040300	-1.16203200	-0.94382200
I	-1.12238500	0.19946000	-2.47987200
I	1.94852200	-2.31024900	2.23809800
C	-1.74243600	5.72352200	0.20225800
H	-2.06952300	-3.41443400	-1.46013500
H	-2.82434200	-1.84142900	-1.09251300
H	-1.93260800	-2.65435100	2.31745600
H	-0.68056700	-3.77752400	1.74528500
H	-4.23572100	-2.81293900	0.63026600
H	-4.19068100	-4.16838700	-0.52347100
H	-3.30836600	-4.63239500	2.02129800
H	-2.31124000	-5.21161900	0.66653300
H	-3.04178800	-0.98143500	1.71320800
H	-2.77939300	0.46589500	0.70274900
H	-0.29505500	1.51256200	2.68102300
H	0.15998500	-0.07075700	3.39624000
H	-2.99926300	1.93890300	2.55892800
H	-3.99516200	0.62065100	3.21163900
H	-1.79054100	1.41722300	4.62675800
H	-2.05331200	-0.33254600	4.40321700
H	2.19120800	-2.49690500	-2.91059800
H	0.47098000	-2.22888700	-3.32422100
H	-0.23261400	-4.57604200	-0.78891800
H	1.30609400	-4.25460900	0.07539800
H	0.06434700	-4.62381400	-3.51160900
H	1.23242700	-6.13451000	-1.94028700
H	2.61459700	-5.01299900	-1.83590500
H	2.25440100	1.35290400	-1.78720400
H	2.21974900	0.24751300	-3.18784400
H	4.07608700	-2.00199200	-1.61740700
H	3.72619000	-1.54131700	0.08196600
H	4.61150400	0.10718900	-3.27392000
H	4.52702000	1.70213400	-2.48198300
H	5.82124500	-0.31937700	-1.18531300
H	4.67510900	0.69302000	-0.26029600
C	2.70238900	4.06635400	-1.53724700
C	2.79287000	1.63552100	2.92690200
H	5.32525900	4.24025000	-1.05030200
H	5.41586200	2.21055800	2.73937400
H	6.60629500	3.39746600	0.90373500
H	0.74596300	4.48824900	0.79149800
H	-1.28095800	2.29010200	-0.00731800
H	-1.62987700	3.30388000	1.40468700
H	-1.62007300	5.10000600	-2.50776400
H	-1.04705100	3.41988900	-2.30065800
H	-0.05288200	4.78356400	-1.72416900
H	-2.25406900	6.47333600	-0.42208900
H	-0.70864300	6.06973700	0.35191700
H	-2.24330900	5.69911700	1.18284400
H	-3.77101800	4.75583800	-1.31789000
H	-4.15105600	0.78424900	-1.94626300
H	-5.63171200	-1.15439600	-1.51363200
H	-7.07784200	-1.20875900	0.52205800
H	-7.06963600	0.72868700	2.09282500
H	-5.60509800	2.68088100	1.66502300
H	1.74212400	-4.56056500	-4.10749700
H	3.38532100	4.17426200	-2.39287800
H	1.84732500	3.44750000	-1.83840500
H	2.29791100	5.06775700	-1.31625300
H	3.44611700	1.56588100	3.80747700
H	1.87627900	2.17273100	3.21300100
H	2.49840000	0.61374000	2.64306900

TS III

E_{vacuum} = -26493.1938

E_{sol} = -26493.2099

$-TS$ = -0.1609

G_{sol} = -26492.4326

C	-3.78047000	4.41136300	-0.25086200
C	-3.65656900	3.90448400	-1.53818800
C	-3.11497300	2.63380000	-1.76767000
C	-2.69414300	1.85569300	-0.66488200
C	-2.81715900	2.36936700	0.64737600
C	-3.36103300	3.64234300	0.83086700
C	-2.10172100	0.50414800	-0.87501200
C	-2.78294800	-0.44837300	-1.58074500
C	-2.22413800	-1.76389200	-2.01540500

C	-3.30321600	-2.84029600	-2.19144300
C	-4.22791800	-2.77850700	-0.99150300
C	-4.21289100	-1.77100400	-0.14508400
C	-4.75953300	-1.14655100	0.99610800
O	-0.91049100	0.28313700	-0.32908700
Sm	1.16404700	0.00633100	0.03034500
O	1.10097100	2.38581800	-0.65454400
C	0.49166200	2.82303300	-1.89070600
C	0.02450500	4.24034700	-1.62268100
C	1.06359100	4.73284400	-0.62143400
C	1.25344700	3.50404500	0.24562700
I	1.56880900	-0.89150200	-2.87713500
I	1.38629200	0.91188300	2.98920000
O	3.00178800	-1.72438200	0.62931600
C	3.92415400	-2.31495300	-0.30848800
C	4.55167900	-3.48079000	0.43070800
C	4.59121400	-2.95674100	1.86220400
C	3.27498500	-2.20665800	1.96283700
O	0.20419100	-2.10715300	0.88642200
C	0.36971800	-3.45669100	0.40832900
C	-0.65197300	-4.29714500	1.17051000
C	-0.97772400	-3.42924600	2.38308500
C	-0.93331500	-2.04548900	1.77554900
O	3.53104600	1.00642900	-0.18268900
C	3.99197200	1.72581000	-1.34462100
C	5.49129400	1.84832900	-1.16282000
C	5.59898000	2.00278200	0.35049900
C	4.55111200	1.01817100	0.83678600
C	-2.65969100	-4.22034100	-2.31995000
H	4.67423200	-1.55482500	-0.58837000
H	3.36215700	-2.58400700	-1.21205400
H	2.44490100	-2.86606100	2.26673100
H	3.28781600	-1.34508600	2.64338800
H	3.90588700	-4.37151500	0.36335300
H	5.53889600	-3.74714600	0.02877900
H	4.67832300	-3.74600100	2.62150500
H	5.44015600	-2.26618900	1.99340500
H	1.40597300	-3.75643300	0.62253600
H	0.22310000	-3.46964600	-0.68257300
H	-1.83476900	-1.81738900	1.18439400
H	-0.74258800	-1.23244200	2.48792700
H	-1.56087200	-4.43751400	0.56681500
H	-0.25895200	-5.28990900	1.42955600
H	-1.95254600	-3.66307700	2.83165800
H	-0.20610800	-3.52599600	3.16384900
H	3.50682200	2.71572400	-1.35912200
H	3.67656700	1.16005900	-2.23218700
H	4.96666300	0.00056000	0.92140600
H	4.07592700	1.28567400	1.79068300
H	5.99602900	0.92642800	-1.49469500
H	5.91345600	2.69181700	-1.72622400
H	6.59743000	1.77987900	0.75132200
H	5.33231900	3.02915200	0.65024600
H	-0.31524100	2.12292500	-2.13525600
H	1.25862400	2.77170900	-2.68136500
H	2.24083600	3.41987000	0.72054100
H	0.48345400	3.42955900	1.02929900
H	-0.02387700	4.84099000	-2.54128800
H	-0.97503200	4.22771000	-1.16317800
H	2.00436200	4.99927700	-1.13125900
H	0.73097000	5.60118900	-0.03659700
C	-2.98133200	2.17486600	-3.19570000
C	-2.42353000	1.56676500	1.85025200
H	-3.97176800	4.50687600	-2.39406400
H	-3.47079600	4.02476800	1.84868200
H	-4.20458700	5.40480300	-0.08907900
H	-3.71001600	-0.11456300	-2.04957900
C	-4.13874000	-2.57211600	-3.45139300
H	-1.67053000	-1.65110700	-2.96483700
H	-1.48028100	-2.09449400	-1.27586200
H	-2.91126800	1.96206400	2.75171500
H	-2.71706800	0.51469400	1.73955600
H	-1.33618500	1.58350800	2.01771300
H	-2.13315500	1.49131300	-3.33761000
H	-3.87935600	1.63281600	-3.53511900
H	-2.85687200	3.04035200	-3.86344900
C	-5.47253200	0.07033900	0.87176300
C	-5.92738100	0.74234400	1.99590800
C	-5.68525500	0.23478400	3.27553300
C	-4.98452700	-0.96516300	3.41665300
C	-4.52376700	-1.64746800	2.29877100
H	-5.63730000	0.48583400	-0.12323000
H	-6.46504400	1.68502000	1.87480000
H	-6.03627600	0.77359000	4.15741400
H	-4.79460400	-1.37201200	4.41241300
H	-3.97998800	-2.58688900	2.40994400
H	-4.98802300	-3.57395300	-0.90691200
H	-2.07030900	-4.47065300	-1.42546500
H	-3.42045200	-5.00583500	-2.45492500
H	-1.98321100	-4.25166900	-3.18882200
H	-3.50940100	-2.63901000	-4.35344500
H	-4.95096000	-3.30996400	-3.54816700
H	-4.59976400	-1.57368000	-3.42570400

TS III'

E_{vacuum} = -26493.1898

E_{sol} = -26493.2057

$-TS$ = -0.1594

G_{sol} = -26492.4264

C	-2.25420100	2.14573500	-1.74493900
C	-2.83778700	0.76720400	-1.62315600
C	-3.95324000	1.60901500	0.26316000
C	-3.92084700	2.86551600	-0.13543200
C	-3.22296800	3.28093800	-1.40430500
C	-2.00583000	-0.33055900	-1.47670000
C	-2.38393300	-1.71120500	-1.91339900
C	-3.47950600	-2.42310200	-1.37931800
C	-3.70550000	-3.74242800	-1.78734900
C	-2.88651400	-4.35971000	-2.72429300
C	-1.84929000	-3.63420100	-3.30019100
C	-1.59399700	-2.31453100	-2.92140200
H	-4.40954800	3.65099500	0.46225700
H	-1.38581500	2.20574300	-1.07397300
O	-0.75865900	-0.19794000	-1.02990100
Sm	1.07414500	-0.01146700	0.04761900
O	0.04263100	-1.98514300	1.12755500
C	-1.31847700	-1.92988400	1.62105000
C	-1.81284700	-3.37016300	1.64560300
C	-0.89428900	-4.06373300	0.64315600
C	0.41859900	-3.35577800	0.89186300
I	-0.01726900	1.97852800	2.08588500
I	2.90668400	-1.81095100	-1.71227500
O	3.28759800	1.31976000	0.35749200
C	3.69980000	2.01279700	1.54761600

C	5.23121700	2.12297400	1.47268600
C	5.59117300	1.43789100	0.14812800
C	4.29721600	1.53663800	-0.63281400
O	1.34286100	1.81340200	-1.61883700
C	1.34381800	3.23266500	-1.34489700
C	1.21728100	3.91249000	-2.70032900
C	1.77598800	2.86324400	-3.65708700
C	1.24934700	1.58617900	-3.03851300
O	2.46802000	-0.89817000	2.03884000
C	1.91624800	-1.10972300	3.35918600
C	3.05587000	-1.66442400	4.19707900
C	3.89823000	-2.38493700	3.14983500
C	3.80944800	-1.42036800	1.98616700
H	3.32593300	1.43173200	2.39898300
H	3.20792800	2.99826000	1.57399600
H	4.17082800	2.54185600	-1.07748900
H	4.17097600	0.76990800	-1.40694800
H	5.53878200	3.17874800	1.46562900
H	5.72242400	1.64763300	2.33352300
H	6.43538300	1.91416400	-0.36922100
H	5.84085800	0.37728600	0.30203700
H	2.29189600	3.47172000	-0.83954100
H	0.51970300	3.45739600	-0.65466100
H	0.19407700	1.40774200	-3.29733600
H	1.83367500	0.68425100	-3.26611900
H	0.15846600	4.10598700	-2.92917000
H	1.75312400	4.87095600	-2.73318300
H	1.44090700	2.99484900	-4.69496600
H	2.87786800	2.86734200	-3.64597300
H	1.08936600	-1.82891300	3.26306800
H	1.50878000	-0.15321700	3.71537700
H	4.52150500	-0.58634600	2.11023400
H	3.95570700	-1.86940600	0.99502500
H	3.63613300	-0.84733400	4.65522500
H	2.69557500	-2.31730700	5.00388700
H	4.93458000	-2.56964800	3.46456700
H	3.44176600	-3.35098500	2.87997600
H	-1.88689500	-1.29370500	0.93135100
H	-1.30711100	-1.44917000	2.61004000
H	0.93252900	-3.74189600	1.79251600
H	1.11696700	-3.36936900	0.04465000
H	-1.68681900	-3.80562900	2.64969300
H	-2.87481300	-3.43719200	1.37672100
H	-0.82235200	-5.14916600	0.79739900
H	-1.23264100	-3.87585500	-0.38667500
C	-4.42325500	-1.80854000	-0.39408900
C	-0.53212100	-1.54578900	-3.64836800
H	-4.54510400	-4.28872300	-1.35035000
H	-1.22903200	-4.08767000	-4.07710800
H	-3.07062400	-5.39363100	-3.02363700
H	-3.78557200	0.56932000	-2.13104900
C	-4.52720500	0.88683800	1.35441600
C	-3.72813500	0.46567000	2.43845600
C	-4.27305200	-0.29756900	3.46387300
C	-5.62070300	-0.66401700	3.43844100
C	-6.42692100	-0.23902600	2.37982900
C	-5.89170500	0.52548500	1.35082600
H	-2.67693000	0.75924000	2.45563300
H	-3.63975100	-0.60637700	4.29935900
H	-6.04336800	-1.26894000	4.24308000
H	-7.48579800	-0.50699200	2.35921500
H	-6.51947700	0.85185100	0.51925000
H	-1.88421100	2.29415300	-2.77840500
C	-4.25719200	3.48891300	-2.51686800
C	-2.45039000	4.58054900	-1.17860200
H	-0.27995800	-2.04116100	-4.59642200
H	-0.87827100	-0.52509600	-3.87046400
H	0.39147900	-1.46220400	-3.05854000
H	-5.05198800	-1.03842000	-0.86504900
H	-5.09439000	-2.56474300	0.03651200
H	-3.90192500	-1.30331900	0.42672300
H	-4.80560300	2.55949400	-2.73111000
H	-4.99735300	4.25153700	-2.22707500
H	-3.77085300	3.82683800	-3.44732400
H	-1.73467700	4.47044600	-0.34894400
H	-1.89558900	4.87123500	-2.08603000
H	-3.13228600	5.40952100	-0.92999900

Int IV

E_{vacuum} = -26493.2486

E_{sol} = -26493.2640

$-TS$ = -0.1585

G_{sol} = -26492.4808

C	-3.37458000	4.77508300	0.17133800
C	-3.55325400	4.21671700	-1.09048000
C	-3.17172800	2.90261900	-1.36854800
C	-2.60311400	2.09682700	-0.33740800
C	-2.42114100	2.67484500	0.95425800
C	-2.80515800	3.99715200	1.17755500
C	-2.22765200	0.70778500	-0.58948700
C	-3.17391800	-0.25024600	-1.26961400
C	-2.55724800	-1.06020100	-2.42182300
C	-3.34639600	-2.37755600	-2.50934800
C	-3.85002000	-2.50571800	-1.09255600
C	-3.77253200	-1.35713800	-0.39733300
C	-4.32720800	-1.13093300	0.94321600
O	-1.08034700	0.22973100	-0.12635700
Sm	1.04631700	-0.04197600	0.07561100
O	0.95134200	2.35210700	-0.48894900
C	0.39949100	2.85938400	-1.72742500
C	0.09250500	4.31846800	-1.45478100
C	1.16988000	4.69050700	-0.44156600
C	1.22824200	3.44238500	0.41716400
I	1.29817200	-0.72111400	-2.91770200
I	1.65511800	0.65672400	3.04031300
O	2.87375600	-1.88451500	0.32352700
C	3.72449300	-2.40797900	-0.71804500
C	4.44947100	-3.58919800	-0.09793800
C	4.54218800	-3.17457600	1.36620500
C	3.19630500	-2.51158500	1.58300600
O	0.19450800	-2.16527000	0.97594300
C	0.11155200	-3.35819200	0.16914600
C	-1.05288300	-4.15482700	0.73588500
C	-1.09166800	-3.68785400	2.18845100
C	-0.78906300	-2.21332800	2.03500100
O	3.42344100	0.93866200	-0.24495700
C	3.80569200	1.74796900	-1.37463000
C	5.32032700	1.74194600	-1.36951800
C	5.61271200	1.73743600	0.12687200
C	4.54683900	0.79102400	0.64936400
C	-2.47920900	-3.55142200	-2.95569900
H	4.41948000	-1.60925800	-1.02776600
H	3.09628400	-2.65790600	-1.58227100
H	2.41178700	-3.25069600	1.81768200

H	3.18123400	-1.73544000	2.35968600
H	3.84568900	-4.50581900	-0.19739300
H	5.42399100	-3.77348800	-0.57071200
H	4.70841600	-4.01433500	2.05498100
H	5.35898900	-2.44903500	1.51225400
H	1.06997800	-3.89445700	0.26453000
H	-0.01877100	-3.06367200	-0.88301500
H	-1.67328800	-1.64099000	1.71487500
H	-0.33964700	-1.73267100	2.91362400
H	-1.98409600	-3.86794500	0.22456700
H	-0.90829300	-5.23773100	0.61958900
H	-2.05812200	-3.86135200	2.67971800
H	-0.30727500	-4.18370200	2.78332400
H	3.41212900	2.76771800	-1.22893200
H	3.34627800	1.30702200	-2.26993800
H	4.88370700	-0.25682400	0.60552900
H	4.19975400	1.00630100	1.66885200
H	5.70155800	0.82256700	-1.84299600
H	5.74449200	2.60396700	-1.90266800
H	6.62736500	1.40136000	0.38157600
H	5.47252000	2.74665200	0.54646000
H	-0.48019100	2.25372500	-1.97590000
H	1.15875400	2.72446300	-2.51480300
H	2.20104000	3.24657500	0.88849300
H	0.45264100	3.44967000	1.19829200
H	0.12360300	4.92361100	-2.37125700
H	-0.90570200	4.41776200	-1.00476600
H	2.13677500	4.86651500	-0.94164200
H	0.92184000	5.58252400	0.14962700
C	-3.29569900	2.42094200	-2.78803100
C	-1.91532100	1.87263300	2.11365200
H	-3.97225300	4.82289300	-1.89821000
H	-2.68159300	4.41283900	2.18124800
H	-3.67589300	5.80545400	0.36970100
H	-4.02976100	0.34383100	-1.62991600
C	-4.54973200	-2.24201200	-3.45305200
H	-2.54301000	-0.50986400	-3.37375400
H	-1.51200500	-1.27476400	-2.15932200
H	-5.19624600	-3.13304700	-3.40535700
H	-4.21281900	-2.12329800	-4.49565800
H	-5.16466700	-1.36790900	-3.18809900
H	-3.05642700	-4.49048200	-2.97532400
H	-1.62031200	-3.69088600	-2.28287400
H	-2.08087900	-3.37850600	-3.96796000
C	-4.78930100	0.13615800	1.32868500
C	-5.33473300	0.34320900	2.59337700
C	-5.42647300	-0.70720400	3.50316600
C	-4.97697600	-1.97515900	3.13213700
C	-4.43597900	-2.18233100	1.86844200
H	-4.72005400	0.97331000	0.63358200
H	-5.68239000	1.34085900	2.86888000
H	-5.84356000	-0.54068000	4.49821400
H	-5.04326400	-2.80738400	3.83642200
H	-4.07841700	-3.17559100	1.58894200
H	-4.34989600	-3.41175700	-0.73625100
H	-0.82171800	1.75741200	2.10878100
H	-2.18699900	2.35570100	3.06309600
H	-2.33320400	0.85680800	2.11110700
H	-2.46175400	1.75836200	-3.06378600
H	-4.22340100	1.85298000	-2.96945100
H	-3.30234500	3.27430800	-3.48136900

Int IV'

E_{vacuum} = -26493.2354

E_{sol} = -26493.2517

$-TS$ = -0.1593

G_{sol} = -26492.4692

C	-2.91934300	2.01490300	-1.76670600
C	-3.35286900	0.76907500	-0.96403300
C	-3.82345700	1.40912700	0.34565600
C	-4.04396000	2.71879900	0.17494300
C	-3.75224500	3.19338500	-1.22867000
C	-2.30549300	-0.31435800	-0.97957200
C	-2.54494900	-1.56279200	-1.73353300
C	-3.54000600	-2.49329900	-1.33212200
C	-3.67397700	-3.69942900	-2.02497000
C	-2.86590600	-4.00407100	-3.11587400
C	-1.93887600	-3.06379000	-3.55457900
C	-1.78063700	-1.84244100	-2.89675100
H	-4.45815900	3.37628000	0.94445900
H	-1.85626000	2.18536500	-1.53705700
O	-1.05050800	0.00498400	-0.66277300
Sm	0.97474400	0.02696500	0.03921600
O	0.23986100	-2.27244700	0.59325700
C	-1.00781300	-2.52566400	1.28413800
C	-1.28724500	-4.01183700	1.11227700
C	-0.49695500	-4.35801100	-0.14619800
C	0.73563400	-3.49755500	0.01889400
I	0.29783200	1.43450300	2.66682600
I	2.76184000	-1.13251400	-2.26596500
O	3.17433400	1.42336900	0.39366300
C	3.77707300	1.78731000	1.64619400
C	5.28064000	1.94882800	1.36835900
C	5.40559800	1.72931000	-0.14576200
C	3.99447400	1.98771600	-0.63215400
O	0.96361800	2.15299100	-1.25727200
C	0.85156800	3.44637600	-0.62038900
C	0.86636900	4.47188600	-1.75095800
C	1.46281400	3.69711000	-2.92461900
C	0.90815500	2.30917100	-2.68627700
O	2.66388900	-1.25415700	1.54772100
C	2.27291500	-1.82160500	2.81623600
C	3.56349000	-2.28537100	3.46176100
C	4.36964700	-2.72329300	2.24426100
C	4.01992200	-1.64204100	1.23990300
H	3.52366400	0.99882800	2.36381800
H	3.31596300	2.72243200	2.00319300
H	3.78766100	3.07208200	-0.71279700
H	3.74199700	1.48506000	-1.57328500
H	5.61609500	2.95641500	1.65340600
H	5.88643400	1.23208300	1.94104800
H	6.14501600	2.38945800	-0.61978300
H	5.67822900	0.68863100	-0.37752700
H	1.70523100	3.54151000	0.06588100
H	-0.06997300	3.46353500	-0.02033000
H	-0.14058900	2.22646400	-3.01665500
H	1.49241800	1.48982500	-3.12620400
H	-0.15920600	4.78828100	-1.99090600
H	1.44116800	5.36918000	-1.48390900
H	1.17178300	4.10253900	-3.90333400
H	2.56262800	3.68017900	-2.87204100
H	1.59371900	-2.66778300	2.62333700
H	1.72944700	-1.04691700	3.37454100
H	4.66839400	-0.75966900	1.36115600

H	4.05377400	-1.95751900	0.18917700
H	4.06250400	-1.44667800	3.97398000
H	3.39778800	-3.08487400	4.19692200
H	5.45135500	-2.78826900	2.42587700
H	4.02286600	-3.70688400	1.88809400
H	-1.77235900	-1.89816100	0.81081300
H	-0.88856900	-2.21556700	2.33330500
H	1.46435500	-3.94800900	0.71900100
H	1.24975400	-3.24250600	-0.91661400
H	-0.91090500	-4.58391100	1.97566300
H	-2.36294800	-4.20902700	1.01190300
H	-0.25397000	-5.42648100	-0.22802500
H	-1.05078700	-4.05211600	-1.04605700
C	-4.45296400	-2.23164100	-0.17067900
C	-0.88105500	-0.80761200	-3.50109800
H	-4.42769100	-4.41618700	-1.68861300
H	-1.34193200	-3.26087700	-4.44876300
H	-2.97734000	-4.95684500	-3.63749900
H	-4.25046600	0.32479300	-1.43205500
C	-4.17766700	0.64465000	1.55561700
C	-3.19521000	0.00773900	2.32070000
C	-3.54356900	-0.73480100	3.44559800
C	-4.88179000	-0.86715100	3.81456800
C	-5.86825300	-0.23087600	3.06270000
C	-5.51745800	0.52440000	1.94532000
H	-2.14842800	0.13536700	2.04804100
H	-2.75963100	-1.20462500	4.04434000
H	-5.15551000	-1.45621100	4.69247900
H	-6.91857600	-0.32234600	3.34793400
H	-6.28857400	1.01752000	1.34915600
H	-3.02504600	1.87640700	-2.85378800
C	-5.06845100	3.33885400	-2.00330000
C	-2.99053600	4.51481100	-1.25687600
H	-3.60704100	5.33815600	-0.86134900
H	-2.70093200	4.78240400	-2.28680000
H	-2.07971400	4.45451000	-0.64220200
H	-5.72975600	4.07853000	-1.52445900
H	-5.61117600	2.38181500	-2.04556300
H	-4.88098900	3.67257100	-3.03728700
H	-0.70580800	-1.02615600	-4.56418900
H	-1.32821700	0.19325100	-3.41942200
H	0.10379100	-0.76954900	-3.01472700
H	-5.19339500	-1.44817600	-0.39778700
H	-5.01198600	-3.13921300	0.09747300
H	-3.91210100	-1.87894200	0.71821900

Product

E_{vacuum} = -26493.2541

E_{sol} = -26493.2754

$-TS$ = -0.1634

G_{sol} = -26492.4961

C	-3.15899900	4.76510000	0.04303800
C	-3.28753000	4.23162200	-1.23507700
C	-3.03158800	2.88047500	-1.47766700
C	-2.62290600	2.06236500	-0.40091500
C	-2.45722200	2.59918800	0.89799600
C	-2.74708500	3.95321900	1.09332200
C	-2.36932600	0.61176900	-0.61594500
C	-3.42618900	-0.24892100	-1.29552000
C	-2.84868900	-1.12278800	-2.42407800
C	-3.57149600	-2.48402800	-2.34563800
C	-4.05324900	-2.48740100	-0.91795600
C	-4.00336300	-1.27999800	-0.33335300
C	-4.50702100	-0.92406600	0.99950500
O	-1.34259600	0.08166400	-0.19767200
Sm	1.18384700	-0.06511500	0.09571700
O	0.84706800	2.36828300	-0.70711100
C	0.52292900	2.82009400	-2.03208600
C	0.36614100	4.32706600	-1.92193800
C	1.33588800	4.66458600	-0.79350500
C	1.12948600	3.49122300	0.14617900
I	1.13327400	-0.99011900	-2.98580500
I	1.71550600	0.89589500	3.12419400
O	2.99073400	-1.92434100	0.41841700
C	3.84712300	-2.47630200	-0.59380600
C	4.51313800	-3.67607600	0.05573000
C	4.62629300	-3.21745000	1.50676300
C	3.30952800	-2.48720800	1.70351300
O	0.17909500	-2.16729200	1.16534000
C	0.06090900	-3.38129000	0.40780400
C	-1.13970100	-4.11039100	0.98935700
C	-1.12433700	-3.63249600	2.43905700
C	-0.74515000	-2.17480700	2.26501700
O	3.53976100	0.97491200	-0.32592900
C	3.93566800	1.55912100	-1.57424600
C	5.44968200	1.47533900	-1.58658300
C	5.77221700	1.70489700	-0.11279800
C	4.65813900	0.93447600	0.57820000
C	-2.63443100	-3.64474700	-2.67081800
H	4.58880000	-1.71049100	-0.88416900
H	3.23201100	-2.70446900	-1.47511700
H	2.49911200	-3.17840300	1.99212000
H	3.33821900	-1.66951400	2.43819100
H	3.86130100	-4.56214400	-0.01629200
H	5.47768300	-3.92454700	-0.40844400
H	4.76094200	-4.03955300	2.22338100
H	5.47437200	-2.52280100	1.62367100
H	0.99270900	-3.95962400	0.53537100
H	-0.03799600	-3.12251800	-0.65774100
H	-1.62383800	-1.56454200	1.99505600
H	-0.24105600	-1.71434600	3.12569100
H	-2.06133100	-3.77209500	0.48998600
H	-1.06354300	-5.20079100	0.87694400
H	-2.08510800	-3.75761500	2.95748000
H	-0.35218500	-4.16629400	3.01671100
H	3.59798100	2.61090700	-1.60163300
H	3.43645300	1.00110700	-2.38034500
H	4.93210000	-0.12218500	0.73548200
H	4.33690200	1.35308200	1.54234700
H	5.77260700	0.47015700	-1.90331800
H	5.90901200	2.21107300	-2.26135000
H	6.77002700	1.35246600	0.18340100
H	5.70580300	2.77823800	0.12789600
H	-0.38499400	2.29922300	-2.36216500
H	1.34082500	2.53099700	-2.71191200
H	1.99968900	3.23585100	0.76635300
H	0.26450100	3.65503100	0.81069200
H	0.59213900	4.83976100	-2.86736300
H	-0.66262100	4.58068700	-1.62366500
H	2.37311700	4.68597600	-1.16615200
H	1.12732700	5.62828000	-0.30865700
C	-3.14475200	2.37444900	-2.89141500
C	-2.02050800	1.78271700	2.07628600
H	-3.58208200	4.87301700	-2.06913100

H	-2.64602200	4.36970600	2.09796700
H	-3.37419300	5.82107300	0.21924800
H	-4.24289500	0.40108300	-1.64632400
C	-4.78875600	-2.52346100	-3.27972000
H	-2.95215400	-0.65593000	-3.41368600
H	-1.77265700	-1.26293200	-2.24326500
H	-5.37715800	-3.44245500	-3.12825200
H	-4.46704300	-2.49811300	-4.33322800
H	-5.45534700	-1.66528700	-3.10049600
H	-3.15240900	-4.61292100	-2.57709900
H	-1.76192000	-3.64922400	-2.00225300
H	-2.25625400	-3.55951600	-3.70133800
C	-5.04740400	0.34614900	1.24851900
C	-5.51999400	0.68530000	2.51326400
C	-5.45878600	-0.23554700	3.55659800
C	-4.93018800	-1.50507900	3.32165100
C	-4.46108200	-1.84488500	2.05767200
H	-5.09945000	1.08210200	0.44473700
H	-5.93173100	1.68187500	2.68399700
H	-5.81682100	0.03501600	4.55157500
H	-4.87221100	-2.23227900	4.13414400
H	-4.03122700	-2.83298600	1.88322600
H	-4.50652300	-3.37057400	-0.45866200
H	-0.92149500	1.71012100	2.14174500
H	-2.36496600	2.25040600	3.00869500
H	-2.40928000	0.75750500	2.04777600
H	-2.43128700	1.56961300	-3.11332600
H	-4.15412300	1.98948200	-3.11031300
H	-2.95242400	3.19226700	-3.60000700

Product'

E_{vacuum} = -26493.2443

E_{sol} = -26493.2655

$-TS$ = -0.1643

G_{sol} = -26492.4870

C	-3.41257500	2.01863600	-1.61148200
C	-3.72235800	0.71540600	-0.84099700
C	-4.09214100	1.24701000	0.53811600
C	-4.39431400	2.54805800	0.46323200
C	-4.25756200	3.11485700	-0.92898800
C	-2.54094600	-0.22650400	-0.95034500
C	-2.69249800	-1.44155400	-1.81412800
C	-3.53874500	-2.49866400	-1.41990300
C	-3.62731600	-3.62106900	-2.24572900
C	-2.92457100	-3.68632000	-3.44530900
C	-2.12138800	-2.62255800	-3.83648600
C	-1.98708900	-1.48596700	-3.03216900
H	-4.76335900	3.13642200	1.30709700
H	-2.34604900	2.24466000	-1.45688500
O	-1.46594800	0.05422000	-0.43528500
Sm	1.13589600	0.08021500	0.09939400
O	0.39401200	-2.37648200	0.32634600
C	-0.72635500	-2.84740600	1.09567100
C	-1.00741800	-4.27018100	0.61424700
C	-0.29644600	-4.32390100	-0.73698400
C	0.90752400	-3.44344300	-0.48051600
I	0.49116000	0.82783700	3.13650200
I	2.68812800	-0.57959700	-2.69684500
O	3.13834100	1.72456900	0.50069600
C	3.88545000	1.78160600	1.72999400
C	5.31902200	2.17420700	1.34648900
C	5.32356100	2.09075900	-0.18162900
C	3.87963400	2.40513900	-0.51073600
O	0.48122100	2.36375600	-0.98931200
C	0.38864300	3.49311500	-0.09689600
C	0.43333000	4.73561100	-0.98190600
C	1.10336900	4.22643700	-2.25665200
C	0.55439100	2.81643600	-2.34635200
O	3.08041700	-1.37056100	1.17226700
C	2.85631500	-2.15654400	2.35859000
C	4.19455100	-2.79755400	2.68456300
C	4.81374300	-2.94831000	1.29859100
C	4.38282500	-1.65330200	0.63736700
H	3.80546300	0.78998600	2.19341900
H	3.40723300	2.50600400	2.40688400
H	3.68150600	3.49327900	-0.44828300
H	3.54742000	2.01678600	-1.48308600
H	5.53781300	3.20181900	1.67438000
H	6.06666100	1.51602900	1.81155100
H	6.03277200	2.78531000	-0.65313400
H	5.55514100	1.07206400	-0.52879100
H	1.23995300	3.43227100	0.59824600
H	-0.53714800	3.40129900	0.49181500
H	-0.46041500	2.81422600	-2.78621900
H	1.18538400	2.10517500	-2.89875700
H	-0.58508400	5.08706200	-1.20812100
H	0.97445300	5.56357300	-0.50363000
H	0.86541800	4.82951100	-3.14391300
H	2.19761900	4.20012900	-2.14303500
H	2.09000900	-2.91445200	2.12582800
H	2.45911400	-1.49134500	3.13865100
H	5.06597000	-0.82747100	0.90758500
H	4.29817800	-1.69552800	-0.45740700
H	4.80640600	-2.12187300	3.30409900
H	4.08191500	-3.74643500	3.22729300
H	5.90548800	-3.07331700	1.30997500
H	4.37469600	-3.81072200	0.77072100
H	-1.56298500	-2.16169000	0.90707300
H	-0.47474300	-2.78868700	2.16623300
H	1.69405800	-3.98271400	0.08182400
H	1.35807900	-2.99459200	-1.37713300
H	-0.56992600	-5.00618600	1.30709700
H	-2.08463300	-4.47446200	0.54260500
H	-0.02226600	-5.34280800	-1.04404000
H	-0.92292000	-3.87846400	-1.52367600
C	-4.33488000	-2.45746000	-0.14713800
C	-1.12505200	-0.35360100	-3.50308300
H	-4.26178300	-4.45494100	-1.93677700
H	-1.58234700	-2.66177000	-4.78548100
H	-3.00970200	-4.56996100	-4.08098300
H	-4.59931300	0.20635000	-1.27677700
C	-4.24744100	0.37276300	1.71885600
C	-3.12680000	-0.08389700	2.42075900
C	-3.27835700	-0.94520500	3.50468000
C	-4.54838900	-1.37076700	3.89193500
C	-5.67123800	-0.90881000	3.20567500
C	-5.52225400	-0.03657400	2.12920800
H	-2.12725100	0.25680000	2.14472000
H	-2.38790700	-1.26502900	4.05048600
H	-4.66639900	-2.05048000	4.73855300
H	-6.67026000	-1.22581300	3.51270500
H	-6.39945700	0.32620200	1.58824200
H	-3.60477100	1.93676800	-2.69141700
C	-5.64184500	3.23460500	-1.57746300

C	-3.55321700	4.46821300	-0.94710900
H	-4.16901600	5.24117400	-0.46091300
H	-3.35786200	4.79800000	-1.98050300
H	-2.59319600	4.41546100	-0.41248500
H	-6.28912000	3.91292600	-0.99990000
H	-6.14455700	2.25624800	-1.62863200
H	-5.56191700	3.63464000	-2.60129200
H	-1.10721400	-0.31677200	-4.60125600
H	-1.47109700	0.62082100	-3.13558500
H	-0.07873200	-0.47995100	-3.17477400
H	-5.20221000	-1.78393700	-0.23384900
H	-4.72334000	-3.45519000	0.09818500
H	-3.75289500	-2.10005100	0.71368700

2-Methylphenyl Dimethylcyclopropyl Ketone Reactant

E_{vacuum} = -26145.6092

E_{sol} = -26145.6286

$-TS$ = -0.1466

G_{sol} = -26144.9861

C	-5.61625200	-2.70058600	1.04797300
C	-5.91844300	-1.34016600	1.08755500
C	-5.08396500	-0.44408400	0.43297500
C	-3.92731600	-0.88411500	-0.23056700
C	-3.62270800	-2.26066200	-0.28891400
C	-4.49466000	-3.14550700	0.35536800
C	-3.02983600	0.14792000	-0.80804900
C	-3.67709100	1.31320800	-1.44160000
C	-2.86337100	2.47972000	-1.93795300
C	-3.69271800	2.67792700	-0.71500000
C	-4.97791200	3.45799500	-0.83269800
O	-1.80315000	0.04086700	-0.72005500
Sm	0.65598800	0.03583400	-0.03852500
O	0.53014500	-0.70582400	-2.52452300
C	-0.13497200	-0.01558200	-3.57863100
C	-0.89765300	-1.10151500	-4.33221400
C	-0.08522600	-2.38343000	-4.05106500
C	1.01281100	-1.92366500	-3.08848500
I	0.85887600	3.16294400	-0.69802100
I	0.99171700	-3.08731100	0.81098200
O	2.35451900	0.48697700	1.91422000
C	3.32098000	1.55384700	1.86114900
C	4.12962400	1.44107900	3.14435900
C	4.04319200	-0.05180400	3.44768300
C	2.61366800	-0.35775100	3.04535300
O	-0.69904900	0.55184800	2.08419400
C	-0.48128100	1.65245800	2.98329600
C	-1.52112500	1.49006800	4.07959500
C	-1.69480500	-0.02579800	4.12729600
C	-1.63045800	-0.38469200	2.65498800
O	3.08794100	-0.11456300	-0.98123400
C	3.52884900	0.62467900	-2.13537400
C	4.95746600	0.17023200	-2.39520700
C	4.95137600	-1.24730800	-1.82961800
C	4.08491100	-1.07205100	-0.59808900
H	3.94280000	1.40278900	0.96361600
H	2.78350600	2.50483000	1.74051300
H	1.90840500	-0.09835800	3.85766200
H	2.42890300	-1.39684100	2.73899900
H	3.65485200	2.02052200	3.95282300
H	5.15729700	1.81091400	3.02318700
H	4.25035000	-0.30364700	4.49709000
H	4.74522100	-0.61821000	2.81427100
H	0.54567600	1.57786600	3.37591400
H	-0.56294800	2.58688500	2.40969000
H	-2.61003500	-0.25747400	2.16396800
H	-1.25812400	-1.39963100	2.45019400
H	-2.46723600	1.97276500	3.78662100
H	-1.19523500	1.92860800	5.03299000
H	-2.63413200	-0.34874100	4.59686200
H	-0.85836000	-0.49353600	4.67147800
H	2.85781000	0.37348600	-2.97325100
H	3.42058300	1.69769100	-1.92207800
H	4.67240900	-0.66333000	0.24521100
H	3.56701500	-1.98077600	-0.25979300
H	5.67005800	0.79819800	-1.83641700
H	5.22476900	0.22233600	-3.45979700
H	5.95230900	-1.63389100	-1.59253600
H	4.47090200	-1.94514700	-2.53464800
H	-0.76453000	0.75834200	-3.12353100
H	0.61932400	0.47375000	-4.22282100
H	1.95745400	-1.71956200	-3.62746300
H	1.21099300	-2.61369300	-2.25676300
H	-0.97714200	-0.87175000	-5.40378900
H	-1.92027400	-1.19255100	-3.93756800
H	0.34336800	-2.81864000	-4.96452800
H	-0.71387000	-3.15273400	-3.58180900
H	-5.30795900	0.62383300	0.45469700
C	-2.44098000	-2.80628900	-1.02643300
H	-6.80091600	-0.98134200	1.61984400
H	-4.27972500	-4.21555100	0.30736600
H	-6.26508300	-3.42201000	1.54900900
H	-4.61314100	1.07627300	-1.95211300
C	-3.00505500	2.79574300	0.62012700
H	-3.19542200	2.94747000	-2.86682400
H	-1.78094200	2.42732100	-1.79163300
H	-2.69615400	3.83871200	0.78992300
H	-3.68501800	2.50187000	1.43795200
H	-2.10022200	2.17808800	0.67179400
H	-5.71115600	3.14153800	-0.07225400
H	-4.78303400	4.53147100	-0.67761200
H	-5.44000900	3.33873000	-1.82383700
H	-2.15222400	-2.17553500	-1.87534600
H	-2.65368200	-3.82156600	-1.39072000
H	-1.55562600	-2.87364700	-0.37067000

Int I

E_{vacuum} = -26145.6015

E_{sol} = -26145.6168

$-TS$ = -0.1415

G_{sol} = -26144.9704

C	6.01823200	-2.29790400	0.74542200
C	6.23768000	-0.99463100	0.29556400
C	5.19336900	-0.08584200	0.26279400
C	3.87017400	-0.43100100	0.65912300
C	3.67140900	-1.74987800	1.18252500
C	4.74179100	-2.64219800	1.19575000
C	2.83780100	0.56095600	0.52777400
C	3.24337200	1.98847300	0.42492800
C	3.58828200	2.65392700	-0.91129700
C	2.34798900	3.04339800	-0.16235800
H	2.33299400	4.02330200	0.32091900
O	1.54905700	0.25016900	0.45500900

Sm	-0.53827800	-0.03088800	0.06791700
O	0.41437900	-2.00754000	-1.08536300
C	1.61428500	-1.87996100	-1.89174100
C	2.22609000	-3.26608700	-1.90868400
C	0.99209100	-4.16034100	-1.84015300
C	0.10334200	-3.40108100	-0.87235100
I	-0.50776800	1.60449700	-2.54659200
I	-1.26312900	-1.75440200	2.52005200
O	-2.92899700	0.95499700	0.37924200
C	-3.75127600	1.51136800	-0.66629700
C	-4.86298500	2.25510100	0.04863100
C	-5.05426800	1.40209700	1.29752600
C	-3.62573200	1.02388600	1.64241000
O	-0.56133100	1.98393000	1.49709600
C	-1.00800100	3.31047300	1.14770200
C	-0.35664400	4.24194200	2.15867500
C	-0.14349600	3.32719600	3.36176700
C	0.25561700	2.03110700	2.68869100
O	-2.36244800	-1.39794500	-1.17893500
C	-2.36936000	-1.66395000	-2.59831100
C	-3.72788900	-2.27446300	-2.88830000
C	-4.02601900	-3.01019300	-1.58649500
C	-3.49736300	-2.03242600	-0.55506000
H	-4.14473000	0.68271700	-1.28011100
H	-3.11291300	2.13463200	-1.30543800
H	-3.13526000	1.79051300	2.26573700
H	-3.51567900	0.05461400	2.14600000
H	-4.53304000	3.27058800	0.32213700
H	-5.76700800	2.34766600	-0.56893500
H	-5.55590600	1.92725500	2.12196200
H	-5.64441500	0.50190400	1.06028700
H	-2.10676500	3.32573800	1.22334000
H	-0.72518600	3.51212900	0.10459500
H	1.31365100	2.02840500	2.38534100
H	0.03834800	1.12297400	3.26739500
H	0.61235500	4.59930700	1.77975700
H	-0.98334100	5.11784800	2.37586500
H	0.62664800	3.69098800	4.05544100
H	-1.08160200	3.19600700	3.92521100
H	-1.54919900	-2.36633200	-2.82267700
H	-2.16812000	-0.71885800	-3.11950400
H	-4.24497000	-1.25702100	-0.31642100
H	-3.15662200	-2.48717800	0.38476500
H	-4.47610800	-1.48396600	-3.06165600
H	-3.71130800	-2.92570700	-3.77297900
H	-5.09066600	-3.23786800	-1.43833900
H	-3.46632800	-3.95880900	-1.54207200
H	2.25267200	-1.12081600	-1.42427600
H	1.30920700	-1.53652300	-2.89380000
H	-0.97274600	-3.53780200	-1.04192300
H	0.32171000	-3.64819700	0.17840800
H	2.84033700	-3.43454200	-2.80349100
H	2.86445900	-3.40569700	-1.02243900
H	0.51280900	-4.23226100	-2.83039600
H	1.20579000	-5.17909600	-1.48872100
H	7.23021900	-0.68577000	-0.04035800
H	4.57136400	-3.64080200	1.60889900
H	6.82994100	-3.02697500	0.77251500
H	3.89700000	2.31802100	1.24231100
H	1.39661400	2.71412600	-0.58262800
C	4.74716500	3.61863000	-0.91620000
C	3.43853900	1.83894800	-2.16977400
H	5.38113500	0.92012400	-0.11033400
H	2.48713500	-3.13767800	2.31752200
H	1.56251200	-2.24865300	1.08638800
H	2.03620600	-1.43216500	2.55379100
C	2.38015100	-2.16740500	1.81172400
H	4.82307000	4.16574700	0.03609400
H	5.70394800	3.09423300	-1.08032300
H	4.63587000	4.36110900	-1.72382500
H	3.28887600	2.49527100	-3.04208100
H	4.33754000	1.22692700	-2.35093100
H	2.56888300	1.17081600	-2.11551400

TS I

E_{vacuum} = -26145.5800
E_{sol} = -26145.5939
$-TS$ = -0.1427
G_{sol} = -26144.9509

C	5.72872300	-2.37753400	0.44988300
C	5.86602600	-1.20729200	-0.29426100
C	4.90331900	-0.20852200	-0.18494500
C	3.78752100	-0.34754200	0.65957600
C	3.67470000	-1.51521300	1.45045000
C	4.64545900	-2.51200500	1.31835600
C	2.78420500	0.73454000	0.67846200
C	3.20619700	2.06135400	0.76424000
C	3.31463100	3.27425200	-0.78399100
C	2.38533200	3.24587500	0.37411600
H	2.43077800	4.11398800	1.04660800
O	1.49638900	0.40978000	0.50347200
Sm	-0.51130100	-0.11002100	0.06010800
O	0.80464300	-1.74036000	-1.27132500
C	1.87309100	-1.33672800	-2.15948200
C	2.82695000	-2.51655300	-2.20434100
C	1.89588000	-3.69825300	-1.95257600
C	0.95932600	-3.12649400	-0.90760800
I	-0.81535900	1.81797200	-2.32825300
I	-1.21610800	-2.11895600	2.28853100
O	-3.03342100	0.47584300	0.37288500
C	-4.00119000	0.78505900	-0.63367000
C	-4.79837100	1.92002900	-0.02286100
C	-4.87448100	1.51779300	1.45765900
C	-3.72979900	0.50562400	1.62912800
O	-0.79876300	1.72849500	1.70301500
C	-1.38501200	3.02952200	1.49251500
C	-0.69748200	3.94762500	2.48758400
C	-0.37306900	2.99260600	3.63290500
C	0.04091200	1.74210100	2.88348000
O	-1.98552500	-1.65528900	-1.35847000
C	-1.80683100	-1.88369900	-2.75890400
C	-3.22589300	-1.96015700	-3.28624900
C	-3.96915200	-2.68861400	-2.15660400
C	-3.05880400	-2.50740200	-0.93185800
H	-4.63776900	-0.10181300	-0.81383200
H	-3.45402000	1.04628700	-1.54759800
H	-2.99210600	0.76720900	2.39653200
H	-4.09948900	-0.50835900	1.85273700
H	-4.24201100	2.86079400	-0.15101900
H	-5.78554000	2.04285300	-0.48935200
H	-4.76419300	2.38396400	2.12556000
H	-5.83953200	1.04734300	1.69480400
H	-2.46603900	2.95453700	1.69237000
H	-1.23837500	3.30560900	0.43850700

H	1.09161100	1.77852400	2.55665300
H	-0.13922500	0.80120300	3.42225300
H	0.22944300	4.35365500	2.05558000
H	-1.33780500	4.79010600	2.78321600
H	0.42237600	3.35919700	4.29585700
H	-1.26995900	2.80135000	4.24419600
H	-1.25956800	-2.83305200	-2.90967100
H	-1.21903800	-1.04817500	-3.15866700
H	-3.53152500	-2.02109300	-0.07111700
H	-2.63491200	-3.46311500	-0.58261400
H	-3.61460900	-0.93970500	-3.42076600
H	-3.29070000	-2.47953700	-4.25223100
H	-4.97197600	-2.27054200	-1.99082300
H	-4.09567700	-3.75680300	-2.38436800
H	2.32704500	-0.42342900	-1.75612200
H	1.42185500	-1.11471400	-3.14051700
H	-0.04161200	-3.57888100	-0.88358700
H	1.39555900	-3.18348500	0.10249400
H	3.36453000	-2.57656000	-3.16042500
H	3.56754400	-2.43418800	-1.39505500
H	1.33956100	-3.96285900	-2.86695400
H	2.41758600	-4.59541400	-1.59243600
H	6.71491500	-1.07617400	-0.96885400
H	4.56157100	-3.40776400	1.94000200
H	6.47034200	-3.17530200	0.37202200
H	4.20095300	2.24677500	1.17259000
H	1.35141000	2.98491200	0.12032800
C	4.51998600	4.15109300	-0.72824700
C	2.94605000	2.64238600	-2.07939500
H	4.98568000	0.70161400	-0.78294900
H	2.84266300	-2.43383500	3.20858800
H	1.61841200	-1.91666400	2.03618200
H	2.43755800	-0.70646600	3.00861300
C	2.59254100	-1.65586300	2.47352200
H	4.84151600	4.34570000	0.30728900
H	5.37416300	3.71692800	-1.27470800
H	4.31513000	5.13825300	-1.18999300
H	2.50623200	3.37990600	-2.77901500
H	3.82591400	2.20610200	-2.58331500
H	2.18856000	1.85891000	-1.94635000

Int II

E_{vacuum} = -26145.5945

E_{sol} = -26145.6091

$-TS$ = -0.1455

G_{sol} = -26144.9681

C	5.81490100	-2.23053900	0.40584100
C	5.82764100	-1.13830900	-0.45764100
C	4.83298900	-0.16950600	-0.34710000
C	3.82434100	-0.26351100	0.62040800
C	3.82826100	-1.35083200	1.51863500
C	4.82591500	-2.32209400	1.38372600
C	2.76790600	0.78837200	0.66350900
C	3.10845200	2.09752500	0.68741400
C	2.18158500	4.08810400	-0.59069200
C	2.13720900	3.22892300	0.63943100
H	2.28653700	3.88024600	1.53058300
O	1.50650000	0.35465000	0.63835000
Sm	-0.46859200	-0.14275300	0.06016300
O	0.87383100	-1.69202800	-1.33129700
C	1.91629100	-1.25220000	-2.23276300
C	2.88689800	-2.41565200	-2.31695900
C	1.97476200	-3.61642300	-2.09020900
C	1.04589900	-3.08802200	-1.01576800
I	-0.43343400	1.90967100	-2.22033400
I	-1.02246400	-2.33794700	2.17329800
O	-2.98752800	0.43831000	0.28499200
C	-3.80453700	1.03989700	-0.73825400
C	-5.06454700	1.49270300	-0.02638200
C	-5.22037400	0.41629700	1.04263400
C	-3.78111400	0.17518400	1.46166500
O	-0.90088900	1.56475600	1.81456200
C	-1.51431400	2.85744000	1.63062800
C	-0.97338700	3.72762900	2.75295400
C	-0.69474200	2.70459300	3.85113400
C	-0.15524800	1.53460000	3.05336700
O	-1.93784900	-1.61277100	-1.46868800
C	-1.72888500	-1.73235200	-2.89020800
C	-3.00389200	-2.35379800	-3.42327700
C	-3.38447900	-3.28750500	-2.27887000
C	-3.04347400	-2.44455000	-1.06290100
H	-4.02200200	0.27771900	-1.50699300
H	-3.22090100	1.84137500	-1.20945500
H	-3.45942200	0.86961000	2.25562500
H	-3.56448700	-0.84754600	1.79798200
H	-4.90896900	2.47952300	0.43915400
H	-5.92399500	1.56987400	-0.70649000
H	-5.85434900	0.71811600	1.88765800
H	-5.65317900	-0.49786100	0.60453200
H	-2.60696900	2.73011800	1.70289200
H	-1.26597800	3.21626200	0.62075500
H	0.91406800	1.64769500	2.81486800
H	-0.32096100	0.54893200	3.50944400
H	-0.03789600	4.21545700	2.44080200
H	-1.68650700	4.50909300	3.04954800
H	0.02153700	3.05822800	4.60511000
H	-1.62719300	2.42332800	4.36698900
H	-0.85901600	-2.38736300	-3.06412000
H	-1.50298100	-0.72948100	-3.27860600
H	-3.88257600	-1.78818800	-0.77921300
H	-2.73283400	-3.01537000	-0.17713400
H	-3.77753600	-1.58200800	-3.56737200
H	-2.85024100	-2.86734100	-4.38234300
H	-4.43865000	-3.59709800	-2.28853700
H	-2.76213600	-4.19654400	-2.30499000
H	2.35550500	-0.33203400	-1.82724800
H	1.44270500	-1.02055200	-3.20074700
H	0.04936400	-3.55076000	-0.99549100
H	1.49118000	-3.17186100	-0.01141300
H	3.41933400	-2.44100600	-3.27758500
H	3.63182300	-2.34547600	-1.51064500
H	1.41039800	-3.86081600	-3.00549200
H	2.51162900	-4.51797900	-1.76501700
H	6.60076500	-1.04426400	-1.22299800
H	4.83654900	-3.16153200	2.08411300
H	6.58083200	-3.00536300	0.32992500
H	4.17464700	2.33336700	0.72790100
H	1.11608100	2.81974900	0.74072100
C	3.17651400	3.86629400	-1.67126100
C	1.33886100	5.31396300	-0.58534800
H	4.81701300	0.67930700	-1.03418700
H	3.17814800	-2.15414900	3.40405800
H	1.84573600	-1.78655000	2.28513900
H	2.67619400	-0.46769500	3.10588400

C	2.83292200	-1.44954500	2.63435100
H	3.38621800	2.79332200	-1.80451100
H	2.81411200	4.26663900	-2.63149700
H	4.15246700	4.35842100	-1.46749500
H	1.81055400	6.15059000	-0.02562500
H	1.14085200	5.67909000	-1.60504600
H	0.36315800	5.12861700	-0.10242700

PreTS II

E_{vacuum} = -26453.8571
E_{sol} = -26453.8725
$-TS$ = -0.1637
G_{sol} = -26453.1276

C	5.18136700	2.89861600	2.59082100
C	5.00090300	3.50440800	1.35069700
C	3.76189300	3.41339800	0.71982600
C	2.69550800	2.72306600	1.30700500
C	2.86198500	2.14927200	2.58423200
C	4.11332700	2.24175100	3.19900700
C	1.40431400	2.60254700	0.56864600
C	0.72503800	3.71475000	0.20638400
C	-0.56550200	3.73499300	-0.56230000
C	-0.46752300	4.55375800	-1.81665700
C	-0.59888000	6.03512300	-1.71999300
O	0.99615100	1.35737000	0.31542100
Sm	0.49458100	-0.64389900	-0.18679000
O	-1.17678000	-0.13594000	1.57260100
C	-1.79780600	1.17704300	1.61821700
C	-2.72829600	1.12560600	2.81256000
C	-1.96971800	0.20130900	3.75963700
C	-1.43504200	-0.84527100	2.80176400
I	-1.50393500	0.16730000	-2.39247100
I	2.16570900	-2.21842300	1.90266800
O	1.04455800	-2.52663400	-1.84128800
C	0.18397400	-3.08127000	-2.85719000
C	0.87611300	-4.35173600	-3.35760400
C	1.95096400	-4.61209600	-2.30324000
C	2.30892400	-3.20398800	-1.88032000
O	2.65488000	-0.33210900	-1.36934500
C	2.57474600	0.33931100	-2.63974100
C	3.48502000	1.53873900	-2.49236200
C	4.62127300	0.95583900	-1.65673600
C	3.88730200	0.03055200	-0.69728400
O	-1.13801700	-2.61871400	0.08775500
C	-2.56952700	-2.51271700	0.01283700
C	-3.13033200	-3.66908800	0.85561900
C	-1.88089700	-4.39440600	1.37286100
C	-0.82557500	-3.98669900	0.36553600
H	-0.79009300	-3.26848400	-2.38530400
H	0.03165200	-2.32899300	-3.64419500
H	2.96855000	-2.71201800	-2.61754000
H	2.75606800	-3.12262000	-0.88103700
H	1.34295900	-4.17249000	-4.33816700
H	0.17244200	-5.18761000	-3.47460100
H	2.81348400	-5.17229200	-2.69002100
H	1.53959600	-5.16410800	-1.44371300
H	2.92477300	-0.35122800	-3.42879700
H	1.52022600	0.58825300	-2.83242700
H	3.61998000	0.53307200	0.24241600
H	4.43087700	-0.89528600	-0.46275500
H	2.95868300	2.32975700	-1.93670000
H	3.81053700	1.94394800	-3.46032400
H	5.19771800	1.71678800	-1.11420100
H	5.31370200	0.38742100	-2.29816000
H	-2.83369100	-1.51515400	0.38255800
H	-2.87331000	-2.57347900	-1.04475500
H	-0.90589300	-4.57875100	-0.56585900
H	0.20471000	-4.01871200	0.74198700
H	-3.74279800	-4.33659500	0.23187100
H	-3.77000700	-3.30849000	1.67380600
H	-2.00859200	-5.48379300	1.43619800
H	-1.59062300	-4.02594000	2.36822000
H	-0.99920900	1.92297000	1.73837000
H	-2.29949400	1.34553400	0.65643800
H	-2.19150300	-1.62217100	2.60116500
H	-0.50004100	-1.32826900	3.11773100
H	-3.69655200	0.68307800	2.53139200
H	-2.91750000	2.12488100	3.22734200
H	-2.59984700	-0.24024900	4.54406100
H	-1.13814800	0.73686500	4.24357300
H	3.60668900	3.87765400	-0.25660700
C	1.71559100	1.49799600	3.29685100
H	5.82118300	4.04379700	0.87211600
H	4.24584800	1.79743900	4.18870800
H	6.14816400	2.95052400	3.09586500
H	1.15897600	4.67402500	0.50212700
C	0.26407900	3.97063900	-2.97607300
H	-1.36882700	4.14624700	0.07746300
H	-0.84743000	2.70204800	-0.81681700
H	0.02235200	4.49076700	-3.91718900
H	1.36438300	4.05027600	-2.84270800
H	0.03123400	2.90049400	-3.09571300
H	0.33992800	6.51104700	-1.36190800
H	-0.82256700	6.49270100	-2.69762200
H	-1.38733700	6.33047000	-1.00805200
H	0.80034300	2.09809000	3.18024000
H	1.93142000	1.39313100	4.36945100
H	1.51344100	0.49403200	2.89510800
C	-3.80410300	3.43755800	-1.28640500
C	-4.44746700	2.65266400	-0.62078800
C	-5.15083000	1.68391600	0.15628200
C	-5.93997600	2.06863400	1.25190900
C	-6.58642300	1.10718400	2.02228600
C	-6.45785400	-0.24634600	1.71058000
C	-5.68023600	-0.63420200	0.61848700
C	-5.02818800	0.31929700	-0.15714500
H	-3.18924300	4.09618100	-1.87271200
H	-4.40601200	0.02910500	-1.00625500
H	-5.58697200	-1.69233100	0.36441900
H	-6.96846900	-0.99844800	2.31526100
H	-7.19571400	1.41696400	2.87356100
H	-6.03330200	3.12849900	1.49279100

TS II

E_{vacuum} = -26453.8515
E_{sol} = -26453.8670
$-TS$ = -0.1609
G_{sol} = -26453.1206

C	2.51107300	5.65944100	1.72157600
C	1.79013800	5.85963100	0.54774600
C	0.82731400	4.92589800	0.16977500
C	0.57009100	3.79050900	0.94656100
C	1.26876200	3.60780600	2.15792600
C	2.23759400	4.54855500	2.51761400

C	-0.43674300	2.79642000	0.47260200
C	-1.71886200	3.17820800	0.27669200
C	-2.82296700	2.31642100	-0.26126100
C	-3.45998000	2.93943100	-1.46896400
C	-4.40823700	4.07094100	-1.24905300
O	0.01931800	1.55493900	0.27927500
Sm	0.74887800	-0.38387400	-0.15077000
O	-0.72031400	-0.86106700	1.80274800
C	-1.88299900	-0.04777100	2.12643700
C	-2.14555800	-0.26232400	3.61394900
C	-0.82607300	-0.83342400	4.12672100
C	-0.37852500	-1.66340900	2.94477400
I	-1.34331000	-0.92265900	-2.34763700
I	3.16430000	-0.68821700	1.78525800
O	2.25747900	-1.74161100	-1.76218200
C	1.86030100	-2.69272800	-2.75945300
C	3.07771800	-3.60274700	-2.98716400
C	4.19202700	-2.95001100	-2.15459200
C	3.66484200	-1.54650500	-1.92608400
O	2.26727000	1.02787200	-1.48555900
C	1.85783000	1.41055900	-2.81202000
C	1.86754300	2.92256000	-2.78290200
C	3.10616700	3.20108300	-1.93545900
C	3.06069200	2.08952100	-0.89517800
O	0.46835700	-2.91335400	0.21294900
C	-0.79696600	-3.59520100	0.17596400
C	-0.62219700	-4.86818900	1.01809100
C	0.84778400	-4.82979300	1.45983000
C	1.47832000	-3.89972800	0.44206500
H	0.96566300	-3.20181800	-2.38272000
H	1.57221900	-2.14832000	-3.67357600
H	3.84515600	-0.89400100	-2.80044700
H	4.04005200	-1.05999500	-1.01772300
H	3.33783500	-3.63385800	-4.05491500
H	2.88454000	-4.63746700	-2.67026400
H	5.16871800	-2.95650900	-2.65787400
H	4.30720400	-3.45612700	-1.18442800
H	2.58812000	1.00930400	-3.53849900
H	0.87454400	0.95898700	-3.00768400
H	2.55509600	2.40472300	0.02703500
H	4.04623800	1.67614300	-0.63861100
H	0.95943800	3.28508900	-2.27714600
H	1.91323100	3.36496700	-3.78759400
H	3.09359600	4.19098000	-1.46075400
H	4.01479700	3.12238100	-2.55341400
H	-1.54677100	-2.89294700	0.56035400
H	-1.04764200	-3.81532000	-0.87407600
H	1.69733300	-4.42762500	-0.50440300
H	2.37651400	-3.37437600	0.79097800
H	-0.82974300	-5.76044400	0.40984500
H	-1.30903100	-4.89195800	1.87605200
H	1.32011600	-5.82174300	1.47194800
H	0.94769400	-4.39479300	2.46507400
H	-1.62201000	0.98762900	1.88067300
H	-2.71860700	-0.37115500	1.49126200
H	-0.93211400	-2.61855600	2.89388900
H	0.70185100	-1.85250900	2.90593500
H	-2.95865800	-0.98924300	3.75704500
H	-2.43677700	0.67220500	4.11303700
H	-0.93545500	-1.43015100	5.04298600
H	-0.09097000	-0.03624300	4.31531800
H	0.25879000	5.06426700	-0.75254900
C	0.95777800	2.45805900	3.06752700
H	1.97711200	6.73683500	-0.07542400
H	2.77968600	4.41085900	3.45667700
H	3.27560700	6.37573100	2.02971000
H	-1.95561900	4.21965600	0.51183300
C	-2.68126600	2.92672100	-2.74126600
H	-3.60075400	2.16413900	0.50968900
H	-2.42571100	1.32548100	-0.52695200
H	-3.31575900	3.16101900	-3.61235800
H	-1.87770300	3.69227300	-2.71953700
H	-2.19845400	1.95124500	-2.90465000
H	-3.86648100	5.01374300	-1.02606900
H	-5.02209300	4.27204700	-2.14275900
H	-5.08377700	3.87558000	-0.40153200
H	-0.12920600	2.29672400	3.12633400
H	1.33772100	2.64816400	4.08128500
H	1.41468600	1.52249600	2.70947200
C	-5.14021500	1.22155800	-1.91824400
C	-5.17470200	0.36234100	-1.04417700
C	-5.10204500	-0.55555900	0.03419700
C	-5.65274900	-0.23150400	1.29067600
C	-5.58452000	-1.13406300	2.34524800
C	-4.97038300	-2.37632600	2.17351600
C	-4.42152700	-2.70550900	0.93256400
C	-4.48145800	-1.81013000	-0.12971600
H	-5.37398400	1.77185100	-2.81139600
H	-4.03902100	-2.05747400	-1.09476900
H	-3.94847400	-3.67945000	0.78742300
H	-4.92610600	-3.08675200	3.00153000
H	-6.01674200	-0.86675900	3.31185600
H	-6.13371900	0.73900000	1.42177000

Int III

E_{vacuum} = -26453.9070

E_{sol} = -26453.9219

$-TS$ = -0.1584

G_{sol} = -26453.1699

C	2.34845200	5.71856900	1.59944900
C	1.64197200	5.86222200	0.40877100
C	0.70033500	4.89849500	0.05244300
C	0.45080800	3.79020800	0.86873600
C	1.13363700	3.66445600	2.09630700
C	2.08167700	4.63402400	2.43351400
C	-0.53236500	2.75929900	0.42473100
C	-1.82167000	3.10751600	0.20886800
C	-2.90927800	2.17601100	-0.20542200
C	-3.78078500	2.65178400	-1.38002200
C	-4.47220800	3.98121800	-1.05939200
O	-0.04943500	1.52354100	0.28505700
Sm	0.77119200	-0.39010600	-0.12212100
O	-0.68102900	-0.87651100	1.86086500
C	-1.91847100	-0.15375500	2.10760400
C	-2.32863100	-0.51471200	3.52588900
C	-0.99373100	-0.86018000	4.17735600
C	-0.29507500	-1.58944600	3.05043600
I	-1.21883700	-0.93950200	-2.41566700
I	3.18326700	-0.50779800	1.84231100
O	2.34092900	-1.74048400	-1.64772400
C	1.98252400	-2.75537100	-2.59718300
C	3.23724400	-3.62342400	-2.78490900
C	4.31507000	-2.90375400	-1.96048200
C	3.74387000	-1.50799400	-1.80866200

O	2.27149800	1.06047100	-1.47821200
C	1.94310700	1.39160700	-2.83899800
C	1.92593400	2.90365200	-2.85715200
C	3.11379200	3.22895200	-1.95560000
C	3.03975200	2.14161700	-0.89020600
O	0.50714800	-2.91474100	0.27102300
C	-0.76821300	-3.59615300	0.24441800
C	-0.47781500	-5.03964900	0.63352100
C	0.81858300	-4.92119700	1.43032700
C	1.54207800	-3.83180300	0.66685300
H	1.11311400	-3.28355600	-2.18901200
H	1.66773500	-2.26791600	-3.53374800
H	3.91197900	-0.89563400	-2.71367700
H	4.09366100	-0.96527800	-0.92251900
H	3.51237100	-3.67256800	-3.84824100
H	3.08032500	-4.65635700	-2.44281900
H	5.30230900	-2.90619600	-2.44272600
H	4.42174700	-3.35957200	-0.96453500
H	2.72618100	0.98510800	-3.50576800
H	0.98301000	0.91520900	-3.08120600
H	2.50405800	2.47384700	0.00861100
H	4.02008400	1.74388000	-0.59166800
H	0.98662500	3.26403000	-2.40989600
H	2.01647600	3.31742700	-3.87100700
H	3.05812000	4.22965500	-1.50750700
H	4.05364000	3.15510400	-2.52551000
H	-1.42970000	-3.08530400	0.95976300
H	-1.19900100	-3.48068300	-0.76021800
H	2.02971700	-4.23741200	-0.23673200
H	2.28219100	-3.26551100	1.24835700
H	-0.31450400	-5.65590700	-0.26473700
H	-1.30434100	-5.48811500	1.20209600
H	1.39349500	-5.85654900	1.47357600
H	0.61789400	-4.59377600	2.46251200
H	-1.69852600	0.91481500	1.98927200
H	-2.65232000	-0.45899200	1.35040200
H	-0.65138600	-2.63248300	2.97319600
H	0.80156700	-1.57816400	3.10656700
H	-2.99461800	-1.39123000	3.52142900
H	-2.86036900	0.31134800	4.01741800
H	-1.09040700	-1.47917200	5.08009300
H	-0.43374700	0.05161400	4.43767300
H	0.14377200	4.99170800	-0.88276100
C	0.82560500	2.54478700	3.04417700
H	1.82410900	6.71830800	-0.24450500
H	2.61251100	4.54093100	3.38438200
H	3.09665400	6.45874600	1.89076800
H	-2.07656700	4.14989500	0.41613600
C	-2.93972600	2.79699500	-2.64991100
H	-3.58838300	1.99820700	0.65050500
H	-2.47472500	1.20168700	-0.47283900
H	-3.55918400	3.12510700	-3.50013900
H	-2.14714800	3.54598400	-2.49226100
H	-2.46089500	1.84197100	-2.91179400
H	-3.73673600	4.79399000	-0.95817600
H	-5.16889900	4.26434300	-1.86447200
H	-5.04657200	3.91386500	-0.12202700
H	-0.26183400	2.38862400	3.11456200
H	1.21229600	2.76579900	4.04892900
H	1.27696800	1.59768100	2.71158400
C	-4.88066300	1.62318300	-1.63299900
C	-5.08906500	0.54044300	-0.93397400
C	-5.09213600	-0.50426100	-0.03129300
C	-5.75639500	-0.38407900	1.22745100
C	-5.74651300	-1.42968800	2.13394300
C	-5.09360800	-2.63276700	1.83285300
C	-4.44543500	-2.77186500	0.59812800
C	-4.43737700	-1.73883900	-0.32348200
H	-5.55038300	1.85716600	-2.47549000
H	-3.91428800	-1.84056100	-1.27447300
H	-3.94259200	-3.71002600	0.35132800
H	-5.09873500	-3.45578300	2.54987800
H	-6.25497600	-1.31285700	3.09364700
H	-6.26500200	0.55218300	1.46256300

Isopropyl Dimethylcyclopropyl Ketone Reactant

E_{vacuum} = -25993.2400
E_{sol} = -25993.2675
$-TS$ = -0.1423
G_{sol} = -25992.6481

H	-6.64439300	0.71974200	-1.37540400
H	-4.31480700	1.79502000	-2.05699300
H	-2.91558200	-1.47334200	-0.96209300
C	-3.48763700	1.47500600	1.98628100
C	-3.94421000	0.76139700	-2.15582200
H	-2.85942200	0.74501400	-1.99431800
C	-3.18476000	0.60514100	0.78623500
C	-4.27146600	-0.24078600	0.27151600
C	-4.67578900	-0.16810700	-1.22181300
C	-3.96976000	-1.36585600	-0.69723700
H	-4.53208400	-2.29215500	-0.56020800
O	-2.06669000	0.60435000	0.27490700
Sm	0.36034100	0.02188000	-0.02151800
O	0.21221000	2.56776200	-0.45728300
C	-0.67156900	3.18662700	-1.40673300
C	-0.64338100	4.67486400	-1.08794600
C	0.74151700	4.84745000	-0.47028100
C	0.87662300	3.56453800	0.32885400
I	-0.10979800	-0.57324500	-3.13309800
I	0.96071300	0.51893200	3.09945300
O	2.12501700	-1.91250900	0.00061200
C	2.89444800	-2.33192800	-1.13714600
C	3.57869200	-3.61744600	-0.70616100
C	3.79528500	-3.36769100	0.78403300
C	2.50855900	-2.65744000	1.16668500
O	-0.81811400	-2.22510300	0.56792400
C	-0.78435400	-3.39281600	-0.27005300
C	-1.67336000	-4.41479100	0.41969000
C	-1.50937100	-4.03523900	1.88899900
C	-1.47941600	-2.52029700	1.80704000
O	2.73305400	0.97227100	-0.54976600
C	3.07366500	1.60918600	-1.79168700
C	4.59130600	1.64181800	-1.81974900
C	4.92260900	1.79730900	-0.33775300
C	3.88343500	0.89523200	0.30559900
H	3.62990900	-1.54262200	-1.37409200
H	2.21229100	-2.43094400	-1.99360700
H	1.70602600	-3.37935700	1.40285100
H	2.59681000	-1.95561400	2.00860500
H	2.90804000	-4.47939100	-0.85652600
H	4.50616100	-3.80794700	-1.26378300
H	3.96039400	-4.28345300	1.36845700

H	4.66173000	-2.70409000	0.93961100
H	0.26098200	-3.73841000	-0.33623600
H	-1.11157600	-3.10544500	-1.27991100
H	-2.50200400	-2.10444200	1.77149500
H	-0.92028300	-2.02415800	2.61387100
H	-2.72226900	-4.28480500	0.10829700
H	-1.37685000	-5.44824700	0.19284400
H	-2.31674900	-4.40704700	2.53504300
H	-0.55306900	-4.41670500	2.28264300
H	2.64805300	2.62821400	-1.79292400
H	2.60574700	1.03801300	-2.60682100
H	4.22601800	-0.15393300	0.33994800
H	3.57625300	1.18921900	1.31978200
H	4.98916100	0.68828600	-2.20404700
H	4.98370200	2.45230000	-2.44943100
H	5.95025600	1.50638300	-0.07945700
H	4.77449800	2.84255500	-0.02046700
H	-1.66736200	2.73375900	-1.29991700
H	-0.29789700	2.96173100	-2.41882800
H	1.91123700	3.23062900	0.48648000
H	0.38708100	3.63745300	1.31475500
H	-0.81004000	5.29613200	-1.97868500
H	-1.41897400	4.92620900	-0.34675200
H	1.51094400	4.89339400	-1.25850300
H	0.83596800	5.74639500	0.15449400
H	-4.11540900	0.43968000	-3.19481300
H	-6.65087200	-0.95605200	-0.76632500
H	-6.34978300	-0.63432200	-2.49101700
H	-5.11701700	-0.36209700	0.95201600
C	-6.16136700	-0.26594600	-1.46976500
H	-4.54457800	1.78469900	1.89971400
C	-3.33740000	0.61432600	3.24893300
C	-2.58470700	2.69754600	2.04225600
H	-2.28703200	0.30610900	3.37216300
H	-3.62545200	1.19900500	4.13525600
H	-3.97236000	-0.28475600	3.21568300
H	-2.67948800	3.30775700	1.13129800
H	-2.85146400	3.32425600	2.90607400
H	-1.53346700	2.39263000	2.15207700

Int I

E_{vacuum} = -25993.2270

E_{sol} = -25993.2409

$-TS$ = -0.1376

G_{sol} = -25992.6180

H	-6.68229200	0.61200700	-1.15781300
H	-4.41730200	1.80074700	-1.92590300
H	-2.84882300	-1.44828300	-1.04255800
C	-3.27684600	1.52772200	2.01033000
C	-4.03464000	0.77661800	-2.07536300
H	-2.94264600	0.79149500	-1.96450000
C	-3.02862700	0.65548400	0.80805000
C	-4.10859600	-0.22947700	0.32705500
C	-4.66870700	-0.17999700	-1.10068300
C	-3.86260600	-1.36087100	-0.64424500
H	-4.38434700	-2.30697100	-0.47504600
O	-1.76082600	0.41445900	0.45951300
Sm	0.25444500	-0.00165200	0.04756400
O	0.18011800	2.40857800	-0.54096700
C	-0.89827300	2.94312000	-1.34635400
C	-0.83178400	4.45263100	-1.16864600
C	0.63084800	4.68556200	-0.80080000
C	0.91894400	3.47951700	0.07008300
I	-0.25678100	-0.77240700	-2.89942000
I	1.27779800	0.75553200	2.85683300
O	2.10661200	-1.81703800	0.04596100
C	2.75413200	-2.35770400	-1.12137700
C	3.45007000	-3.61943500	-0.64574600
C	3.81825400	-3.25127900	0.78802600
C	2.59554700	-2.47101300	1.23436300
O	-0.54309600	-2.21912100	0.83885700
C	-0.59105200	-3.43547500	0.06591300
C	-1.72907100	-4.24749200	0.65948400
C	-1.72178200	-3.79215800	2.11588700
C	-1.44119800	-2.31054700	1.96842400
O	2.55536300	0.86236700	-0.82233500
C	2.76976200	1.45442000	-2.12008600
C	4.27562500	1.57417300	-2.26347100
C	4.70823700	1.80765600	-0.81969900
C	3.78288200	0.86949000	-0.06816800
H	3.47571600	-1.61190400	-1.49743600
H	1.98971200	-2.51232000	-1.89470000
H	1.80069700	-3.13337400	1.61669100
H	2.79016500	-1.69809800	1.99027900
H	2.75166200	-4.47190800	-0.65659500
H	4.31386300	-3.88121100	-1.27226800
H	4.01677700	-4.11932800	1.43158300
H	4.71269400	-2.60727500	0.80119000
H	0.37783200	-3.95093300	0.17862400
H	-0.73051800	-3.16811700	-0.99139800
H	-2.35096600	-1.74045200	1.73102800
H	-0.93688200	-1.84757200	2.82759000
H	-2.68103200	-3.97337300	0.18053200
H	-1.57695500	-5.32822400	0.53262100
H	-2.66836500	-3.98400300	2.63936300
H	-0.91206400	-4.28919100	2.67476400
H	2.27922600	2.44191400	-2.13598200
H	2.28313100	0.81386000	-2.86796500
H	4.18555400	-0.15749900	-0.04338200
H	3.54643000	1.17417100	0.96067500
H	4.70276900	0.63201900	-2.64394800
H	4.56667700	2.38071400	-2.95042400
H	5.76837100	1.58817100	-0.63214800
H	4.52008700	2.85330000	-0.52582500
H	-1.83803800	2.49705600	-0.99527100
H	-0.71739600	2.62564300	-2.38510300
H	1.97316700	3.17648000	0.10609400
H	0.56307800	3.61837400	1.10395600
H	-1.14702600	4.98886900	-2.07396900
H	-1.48227600	4.76914800	-0.33898800
H	1.26587900	4.67148700	-1.70190900
H	0.80413200	5.63342300	-0.27289200
H	-4.25496400	0.47881400	-3.11346800
H	-6.56618800	-1.03470800	-0.49137400
H	-6.40844400	-0.77152500	-2.24438100
H	-4.88915700	-0.41308600	1.07621800
C	-6.15804400	-0.35364800	-1.25444400
H	-4.35848800	1.75304600	2.01618800
C	-2.93702100	0.79629900	3.31686900
C	-2.51541100	2.84777000	1.93064200
H	-1.86309900	0.55443000	3.34859800
H	-3.16714400	1.42036300	4.19618200
H	-3.50943400	-0.14077900	3.40978900
H	-2.81971000	3.42447900	1.04324900

H	-2.69993000	3.46685900	2.82269800
H	-1.43281500	2.65788200	1.87102700

TS I

E_{vacuum} = -25993.2124
E_{sol} = -25993.2262
$-TS$ = -0.1383
G_{sol} = -25992.6059

H	-6.72871200	0.90752500	-0.91892800
H	-4.47639900	2.09297300	-1.52806200
H	-2.97888300	-1.24679300	-1.02815100
C	-3.13756900	1.28137400	2.39925700
C	-4.10477600	1.09605500	-1.82570800
H	-3.01582300	1.06782400	-1.69182100
C	-2.95066800	0.38128000	1.20178400
C	-4.01440100	-0.34694300	0.66782700
C	-4.76830800	0.00542300	-1.05756800
C	-3.97660300	-1.15866000	-0.58254200
H	-4.49926000	-2.12480000	-0.55364100
O	-1.72431300	0.34867800	0.66128900
Sm	0.24288900	-0.05356100	0.03407200
O	0.19749200	2.40904400	-0.31551500
C	-0.89086100	3.04235200	-1.03142700
C	-0.79418400	4.52240500	-0.69596000
C	0.68481800	4.69321300	-0.36049200
C	0.97718300	3.40201700	0.37522000
I	-0.46218500	-0.43772900	-2.93458600
I	1.64738800	0.41924300	2.74686000
O	1.94955500	-1.96341800	-0.35975500
C	2.46086700	-2.40628000	-1.63426700
C	3.32866200	-3.61811200	-1.33152800
C	3.78313400	-3.34565200	0.09881400
C	2.53115500	-2.74245300	0.70161200
O	-0.54106600	-2.28902600	0.77516400
C	-0.83110900	-3.38347600	-0.11696400
C	-1.93253300	-4.17642800	0.56439700
C	-1.63785500	-3.92861000	2.04090900
C	-1.24065100	-2.46634000	2.02878500
O	2.51461600	0.81670100	-0.91092000
C	2.68890200	1.58402000	-2.10712700
C	3.87544800	2.51700300	-1.83200100
C	4.52612600	1.93322200	-0.56295900
C	3.82577600	0.59453600	-0.39056100
H	3.03783500	-1.57706200	-2.07358300
H	1.61112000	-2.61063800	-2.29914500
H	1.81223600	-3.52219500	1.00849500
H	2.70143800	-2.07125100	1.55433400
H	2.72778700	-4.54104800	-1.36895300
H	4.15528300	-3.72557600	-2.04726600
H	4.11669600	-4.24469000	0.63511100
H	4.60542600	-2.61230600	0.11316900
H	0.08882000	-3.98112700	-0.23724700
H	-1.11275200	-2.97022200	-1.09641800
H	-2.11908100	-1.80243400	2.03058100
H	-0.55411200	-2.16603400	2.83232100
H	-2.91696600	-3.76206700	0.29940000
H	-1.91571200	-5.23773100	0.28070100
H	-2.49903100	-4.11778300	2.69619400
H	-0.80031800	-4.55933000	2.38131300
H	1.73910600	2.09152900	-2.30926500
H	2.89142800	0.89341400	-2.94342000
H	4.32245800	-0.19778500	-0.98154000
H	3.71645700	0.27108700	0.65222900
H	4.56807700	2.52613700	-2.68521700
H	3.54575600	3.55318700	-1.67030000
H	5.61553300	1.82101600	-0.65059000
H	4.32703300	2.56870200	0.31269400
H	-1.82710000	2.57348300	-0.70470500
H	-0.74469100	2.83453800	-2.10313100
H	2.02385300	3.07665100	0.34839900
H	0.65485600	3.44129900	1.42867000
H	-1.13241100	5.15622700	-1.52685200
H	-1.41049900	4.75554100	0.18591700
H	1.28787400	4.75643800	-1.28141000
H	0.89628800	5.58072800	0.25158100
H	-4.29340800	0.99219000	-2.91225500
H	-6.64418700	-0.76578000	-0.31122100
H	-6.62580100	-0.45131600	-2.05773100
H	-4.91814000	-0.41440400	1.27728400
C	-6.25819400	-0.07725300	-1.08004000
H	-4.21978100	1.30130100	2.61415400
C	-2.40919300	0.74235000	3.63234200
C	-2.68565800	2.71019700	2.09792900
H	-1.32874500	0.65263400	3.43898300
H	-2.54128700	1.41423200	4.49607400
H	-2.79148900	-0.25100300	3.91500500
H	-3.23546900	3.12894900	1.24001200
H	-2.84639700	3.36951500	2.96568400
H	-1.61113300	2.71957200	1.85904600

Int II

E_{vacuum} = -25993.2345
E_{sol} = -25993.2483
$-TS$ = -0.1397
G_{sol} = -25992.6283

H	-5.99972800	-1.54598900	-2.50894600
H	-5.37634800	0.88593500	-2.55972400
H	-2.97246500	-1.07827500	-0.01420700
C	-2.71222000	2.40391200	2.21585200
C	-4.52816600	0.69005500	-1.88260900
H	-4.37531100	1.57334300	-1.24319800
C	-2.74086200	1.18616300	1.31561200
C	-3.88562500	0.50952100	1.07148100
C	-4.74728000	-0.53711200	-1.06887100
C	-3.99467200	-0.71384600	0.21869900
H	-4.48277700	-1.52685900	0.79143500
O	-1.56194900	0.81828500	0.81230600
Sm	0.26032900	0.03461000	0.09665200
O	0.16516800	2.16139000	-1.16835100
C	-1.02200900	2.53746900	-1.90737500
C	-0.90963200	4.03866400	-2.11609100
C	0.59961200	4.26426900	-2.09440000
C	1.03689800	3.29513300	-1.01408700
I	-0.91736100	-1.38400300	-2.39461500
I	1.94060300	1.35962300	2.31105700
O	1.90873400	-1.94422100	0.25447900
C	2.25969000	-2.84071700	-0.81761800
C	2.92072500	-4.02959200	-0.14537200
C	3.59535100	-3.37457400	1.05581000
C	2.55719000	-2.35134200	1.47682400
O	-0.55790000	-1.78905200	1.57810100
C	-0.88487000	-3.13198500	1.16523800
C	-1.96374300	-3.59546100	2.12708300
C	-1.59952400	-2.83755100	3.40012400

C	-1.19779500	-1.48765100	2.84124700
O	2.36986400	0.37433400	-1.38808200
C	2.34064600	0.58535400	-2.81472900
C	3.77178300	0.38954400	-3.27557200
C	4.55452700	0.92028900	-2.07931300
C	3.73214000	0.39941400	-0.91495200
H	2.95578400	-2.31723100	-1.49550100
H	1.34530500	-3.07344200	-1.37960300
H	1.79483600	-2.78791700	2.14368800
H	2.96241700	-1.45129400	1.95889600
H	2.16082200	-4.75448800	0.18871500
H	3.61742900	-4.55299700	-0.81453000
H	3.84014200	-4.07711400	1.86422900
H	4.52728100	-2.87349800	0.74704700
H	0.02661400	-3.74871900	1.24859600
H	-1.20054700	-3.10187000	0.11266300
H	-2.07375700	-0.85196000	2.64306100
H	-0.47438700	-0.93018300	3.45212200
H	-2.95396700	-3.27827600	1.76550200
H	-1.97201100	-4.68751900	2.24727500
H	-2.42932400	-2.75760500	4.11551500
H	-0.74970000	-3.32055900	3.90968800
H	1.99048200	1.61308500	-3.01004100
H	1.61461600	-0.11811200	-3.24405800
H	4.02320800	-0.62865500	-0.64136000
H	3.76677000	1.02038200	-0.00948300
H	3.98314300	-0.68112300	-3.43047900
H	3.98433900	0.91778900	-4.21522200
H	5.59695200	0.57520100	-2.04075900
H	4.56142200	2.02249000	-2.08446900
H	-1.89736100	2.22835900	-1.32124500
H	-1.01319400	1.97366600	-2.85318800
H	2.07012500	2.93541600	-1.10712600
H	0.90658300	3.71209000	-0.00224300
H	-1.38811000	4.36169300	-3.05054700
H	-1.38349000	4.57897400	-1.28262700
H	1.04681200	3.98900000	-3.06373900
H	0.88520800	5.30022200	-1.86585100
H	-3.62539700	0.58552700	-2.52154200
H	-5.61718500	-2.51155100	-1.05482100
H	-4.38623600	-2.26044300	-2.31123400
H	-4.80497400	0.90306600	1.51453100
C	-5.21790500	-1.76745100	-1.76399000
H	-3.76151700	2.65150700	2.44836900
C	-1.98277800	2.10030200	3.52407400
C	-2.07632000	3.59578000	1.50646000
H	-0.93728600	1.81475600	3.33049100
H	-1.97265600	2.98539200	4.17999800
H	-2.47505600	1.27983600	4.06910900
H	-2.62871100	3.85069700	0.58811300
H	-2.06801600	4.48308000	2.15839700
H	-1.03528400	3.36308500	1.23566300

PreTS II

E_{vacuum} = -26301.4941

E_{sol} = -26301.5090

$-TS$ = -0.1597

G_{sol} = -26300.7876

H	-0.14816300	4.94684000	1.37911300
H	1.25986500	2.60674000	-0.10045200
H	2.11849400	3.73445300	0.97627500
C	6.57896800	-1.40580900	0.95853700
H	0.60274400	3.33250100	-2.38467800
H	2.50193400	6.39396200	-1.52722000
C	-1.05532400	3.08480700	1.09142000
C	-0.01862900	3.94351400	0.96284000
C	1.27745300	3.64720200	0.26164200
C	1.51676700	4.58031900	-0.89156100
C	2.10403500	5.92220300	-0.61395300
O	-1.03109000	1.83518300	0.62941000
Sm	-0.82555600	-0.20166700	0.10157300
O	-2.68682800	0.46246400	-1.39421100
C	-2.58698400	1.63831800	-2.23320900
C	-4.01732900	1.98933700	-2.60805900
C	-4.71885800	0.63562700	-2.53877700
C	-4.05030600	0.00799700	-1.33186500
I	1.05816300	0.25292800	-2.31378100
I	-2.83703500	-1.21051300	2.20600700
O	0.51197600	-2.35671100	0.54969600
C	1.39140900	-3.02409700	-0.37846700
C	2.21137800	-3.98756300	0.46062000
C	1.24129900	-4.33678000	1.58407600
C	0.57224400	-3.00027500	1.83930400
O	0.96716100	0.09425300	1.78314000
C	2.37780400	0.00930400	1.47517300
C	3.07520900	0.86451000	2.51909700
C	2.10864700	0.79871000	3.69785200
C	0.76911800	0.85710600	2.99351300
O	-1.69281900	-2.21461500	-1.30936100
C	-1.78372500	-2.23616300	-2.74867900
C	-1.96450700	-3.69622700	-3.11466200
C	-2.78144500	-4.20850500	-1.93343800
C	-2.13495400	-3.47773300	-0.77117200
H	0.76981500	-3.54941600	-1.12368600
H	1.98294700	-2.26125300	-0.90160600
H	1.16733900	-2.36579400	2.51710100
H	-0.45180300	-3.06243300	2.23172200
H	3.10292100	-3.47992300	0.86229300
H	2.55024700	-4.85781900	-0.11873800
H	1.73120500	-4.73545900	2.48303800
H	0.50191700	-5.07889800	1.24114600
H	2.67783100	-1.04922100	1.54188200
H	2.53182800	0.35872700	0.44591000
H	0.49676700	1.88824900	2.71969600
H	-0.06382100	0.39368800	3.53992500
H	3.17130700	1.89826700	2.15433000
H	4.08074100	0.48786800	2.74827500
H	2.23600100	1.62125400	4.41497800
H	2.22047400	-0.15364700	4.24185900
H	-2.65609400	-1.63423300	-3.05482500
H	-0.87573800	-1.76607000	-3.14928200
H	-1.25284400	-4.02060900	-0.39303300
H	-2.80408800	-3.27075400	0.07503500
H	-0.98790800	-4.20564800	-3.15809200
H	-2.45958700	-3.82543900	-4.08701000
H	-2.74932400	-5.29978700	-1.80935600
H	-3.83690800	-3.90898200	-2.03898100
H	-2.06554300	2.41785500	-1.66122000
H	-1.97172600	1.36855600	-3.10554500
H	-4.03462800	-1.08989600	-1.32909200
H	-4.49836600	0.34826800	-0.38421100
H	-4.07932400	2.46529500	-3.59610400
H	-4.45162200	2.67728300	-1.86688300
H	-4.51125300	0.04487200	-3.44611800

H	-5.80837500	0.71150300	-2.41957300
H	-0.35273800	4.76427400	-1.94652100
H	1.34740100	6.62628800	-0.20444000
H	1.08674700	4.95283900	-2.97362900
H	2.91420000	5.86897800	0.13240700
C	0.68263600	4.39718700	-2.11240300
C	-2.31952000	3.48619000	1.82558600
C	4.74784700	1.40649500	-0.68203300
C	-2.41488900	2.77966700	3.17737900
C	-3.55827900	3.20792100	0.98018800
H	6.50429500	0.73670100	1.23649300
C	4.25664600	2.48856600	-0.93094500
H	-2.25207700	4.57269600	2.00289100
H	3.76266400	3.41813200	-1.15788700
H	4.07335900	-0.82417300	-1.95046700
H	-2.44535700	1.68713400	3.04566100
H	-1.55354900	3.03176300	3.81526600
H	-3.63433600	2.13152800	0.76491300
H	-3.51631300	3.75403800	0.02444900
H	4.86898900	-3.12773700	-1.42930400
H	6.46265600	-3.50151000	0.44869300
H	7.28214800	-1.56332700	1.77874900
C	6.14797800	-0.11735100	0.65846200
C	5.24247600	0.09921200	-0.39256600
C	4.78613100	-0.99820600	-1.14260900
C	5.22656500	-2.28197500	-0.83806500
C	6.11963700	-2.49196600	0.21343600
H	-4.47310300	3.51471600	1.51060500
H	-3.33174100	3.07828300	3.71039400

TS II

E_{vacuum} = -26301.4875
E_{sol} = -26301.5029
$-TS$ = -0.1563
G_{sol} = -26300.7794

H	2.07406400	4.05294000	1.74405200
H	2.20594000	1.53543700	-0.07653900
H	3.52569500	2.00935900	1.02103200
C	6.12538900	-2.02454100	1.07477200
H	1.87704000	2.73603600	-2.20688700
H	4.87588500	4.67310700	-1.12890300
C	0.39104400	2.89069500	1.32311500
C	1.71225100	3.16211000	1.22224100
C	2.70345500	2.39809400	0.39163500
C	3.29034100	3.27512600	-0.67668400
C	4.31829500	4.27591800	-0.26432500
O	-0.18749100	1.83890000	0.74466600
Sm	-0.90556500	-0.03852000	0.09226500
O	-2.37008400	1.47724300	-1.21172200
C	-1.82277000	2.56347900	-1.99699900
C	-2.97792300	3.52656800	-2.21308200
C	-4.19137900	2.60275900	-2.15696800
C	-3.79185800	1.63922400	-1.05688600
I	0.91425600	-0.17600700	-2.40858000
I	-3.10660800	-0.34287900	2.23044800
O	-0.50083200	-2.58260300	0.26133200
C	0.03368400	-3.44460600	-0.76234800
C	0.44848700	-4.71338000	-0.04138600
C	-0.58530500	-4.78904200	1.07740000
C	-0.71856300	-3.33127500	1.47619600
O	0.79365200	-0.65912600	1.78155000
C	2.08387400	-1.19963300	1.41762600
C	3.01917600	-0.83673500	2.55876400
C	2.06273800	-0.69954000	3.73946400
C	0.87095700	-0.03710900	3.08213500
O	-2.56788900	-1.37652400	-1.39482900
C	-2.73260100	-1.19328700	-2.81601800
C	-3.46665900	-2.43044200	-3.29420200
C	-4.35705600	-2.73910400	-2.09542000
C	-3.42487400	-2.44171400	-0.93563000
H	-0.75921400	-3.63631000	-1.50635700
H	0.84991600	-2.90984800	-1.26521800
H	0.05010100	-3.03182200	2.20700700
H	-1.70217600	-3.04908400	1.87609900
H	1.46167500	-4.60140600	0.37834400
H	0.44938300	-5.58915700	-0.70487500
H	-0.28091100	-5.42449200	1.92035000
H	-1.54257600	-5.17388200	0.68950100
H	1.97318900	-2.29014300	1.30460700
H	2.38569100	-0.77955800	0.44923200
H	1.03459900	1.04293100	2.94541700
H	-0.09396800	-0.20318400	3.57989200
H	3.51146600	0.12531800	2.35211300
H	3.80273600	-1.59235800	2.69749400
H	2.47000300	-0.09834200	4.56395300
H	1.78587600	-1.68948600	4.13826700
H	-3.33030900	-0.28175000	-2.98597100
H	-1.73546200	-1.04898200	-3.25295600
H	-2.79424900	-3.31292800	-0.69289600
H	-3.92283100	-2.10358200	-0.01665900
H	-2.75600600	-3.25460200	-3.46951600
H	-4.02109500	-2.25394100	-4.22623500
H	-4.73307000	-3.77132900	-2.07386400
H	-5.22480500	-2.05984600	-2.07732000
H	-0.97781000	2.98925500	-1.43912700
H	-1.44574200	2.13433800	-2.93843400
H	-4.25006200	0.64347600	-1.12475100
H	-3.99622300	2.04324200	-0.05219400
H	-2.88661200	4.07290300	-3.16171200
H	-3.02383300	4.26206800	-1.39566000
H	-4.31478200	2.06767400	-3.11290400
H	-5.13123300	3.12527100	-1.93198100
H	1.64241500	4.36378300	-1.52477800
H	3.85083700	5.15125500	0.23249500
H	2.97764300	4.07634900	-2.65938600
H	5.04218000	3.84552500	0.44538100
C	2.40978000	3.62268200	-1.82982700
C	-0.53376400	3.77923700	2.13316200
C	4.99407300	0.63921500	-1.27778200
C	-1.02027000	3.08252700	3.40273200
C	-1.71616200	4.24059300	1.28555000
C	4.83730100	1.73661000	-1.80105800
H	4.94010800	2.55063700	-2.49497100
H	0.05401300	4.66587200	2.42422100
H	3.68956900	-1.59669600	-1.91705600
H	3.93449200	-3.81335500	-0.82030100
H	-1.57024300	2.16001000	3.16068100
H	-0.17656000	2.82231500	4.06041100
H	-2.31044400	3.37234600	0.96335800
H	-1.37500600	4.78041500	0.38784800
H	5.49396800	-4.09232900	1.10688300
H	6.81368100	-2.13933600	1.91492500
H	6.57255500	0.07193600	0.81143500
C	5.99442500	-0.78434300	0.46081500

C	5.11660400	-0.61549800	-0.62810000
C	4.37710400	-1.72734800	-1.08035800
C	4.51267000	-2.96117600	-0.45494500
C	5.38516200	-3.11921700	0.62388900
H	-2.37450200	4.91059300	1.86017600
H	-1.69994600	3.73841000	3.96999500

Int III

E_{vacuum} = -26301.5408

E_{sol} = -26301.5550

$-TS$ = -0.1545

G_{sol} = -26300.8267

H	2.24210100	3.99842900	1.60840000
H	2.30850200	1.33850200	0.00597000
H	3.61473900	1.88428000	1.07448100
C	6.46325000	-1.97304800	0.90754700
H	1.97570800	2.43190000	-2.24706100
H	4.89651400	4.57695400	-1.18244700
C	0.53320100	2.86253500	1.24821100
C	1.85809900	3.10139900	1.11515700
C	2.82560500	2.23448100	0.38163100
C	3.53648900	2.91180000	-0.80595300
C	4.31879100	4.14775300	-0.34827900
O	-0.07572800	1.80010400	0.72410800
Sm	-0.92555400	-0.03532300	0.10754500
O	-2.24310100	1.55454400	-1.26062600
C	-1.58871600	2.57283100	-2.05515600
C	-2.66331400	3.60538600	-2.35248000
C	-3.94231800	2.77371900	-2.30759600
C	-3.65175200	1.82882000	-1.15850700
I	0.88262200	-0.42846700	-2.38311800
I	-3.12395400	-0.07170000	2.26015300
O	-0.80843800	-2.59612400	0.37338000
C	-0.41526000	-3.54443400	-0.63824000
C	-0.09288100	-4.82223000	0.11332200
C	-1.06784900	-4.75068300	1.28414000
C	-1.03404100	-3.27336700	1.62805600
O	0.81782800	-0.76813000	1.70900900
C	2.00258200	-1.50214400	1.32380200
C	3.06060900	-1.13360800	2.34651900
C	2.22123100	-0.82434800	3.58203900
C	1.04129100	-0.09715500	2.97004500
O	-2.71688000	-1.26000000	-1.34220100
C	-2.83164400	-1.14959500	-2.77531500
C	-3.67983400	-2.33232600	-3.19799500
C	-4.63133400	-2.46290100	-2.01371200
C	-3.71010400	-2.17900400	-0.84086500
H	-1.26130500	-3.68256900	-1.33373800
H	0.42333900	-3.11392300	-1.20131000
H	-0.20109700	-3.02672900	2.30668900
H	-1.96243400	-2.87835700	2.06280800
H	0.94731900	-4.80178000	0.47707600
H	-0.21640600	-5.71670000	-0.51263800
H	-0.78375100	-5.38282000	2.13661900
H	-2.07841900	-5.04941400	0.96096600
H	1.76082800	-2.57813200	1.35177000
H	2.26580400	-1.22786500	0.29424600
H	1.27553500	0.95911500	2.76697800
H	0.10694100	-0.15926400	3.54483100
H	3.60454700	-0.23641500	2.01700700
H	3.79490200	-1.93776700	2.48628500
H	2.74620100	-0.20817500	4.32484800
H	1.89279700	-1.75394900	4.07563500
H	-3.32735800	-0.19392400	-3.01757700
H	-1.81617100	-1.13568800	-3.19288800
H	-3.19374000	-3.09144300	-0.50022800
H	-4.19836700	-1.71465200	0.02651900
H	-3.05650100	-3.23623600	-3.29519000
H	-4.18628100	-2.16263100	-4.15817600
H	-5.11295400	-3.44741000	-1.93564500
H	-5.42485300	-1.70048800	-2.07506800
H	-0.73942500	2.95815800	-1.47501200
H	-1.20352000	2.08365100	-2.96313400
H	-4.18168700	0.86827300	-1.20351300
H	-3.85664900	2.28977100	-0.17854400
H	-2.49902400	4.10499600	-3.31683700
H	-2.68576000	4.37483600	-1.56588400
H	-4.07141700	2.21108300	-3.24678600
H	-4.84849100	3.37109200	-2.13729100
H	1.79088300	4.02075200	-1.45165100
H	3.63611600	4.92830400	0.02055100
H	3.01743000	3.80114700	-2.73132200
H	5.02528700	3.89357500	0.45769700
C	2.52014100	3.31352200	-1.87734000
C	-0.35324000	3.79052000	2.05494200
C	4.84151000	0.75145200	-1.00327900
C	-0.78951900	3.13220700	3.36311700
C	-1.56475800	4.23697700	1.24263800
C	4.54083100	1.94810900	-1.42562300
H	5.06459300	2.34087300	-2.31372600
H	0.25411700	4.67916500	2.29547300
H	3.72612700	-1.53330500	-1.82508300
H	4.28598600	-3.79640700	-0.98350600
H	-1.36521800	2.21461300	3.16613500
H	0.08204100	2.87184700	3.98360100
H	-2.17681100	3.36545600	0.96627400
H	-1.25530900	4.75312900	0.31989700
H	6.03177600	-4.09336700	0.77761800
H	7.23314400	-2.09835100	1.67229000
H	6.69063700	0.16924200	0.83585300
C	6.16691100	-0.70408100	0.44394300
C	5.16238300	-0.50895600	-0.55498400
C	4.49550700	-1.66754700	-1.06320300
C	4.80956200	-2.92564400	-0.58135000
C	5.78892000	-3.09585500	0.40735900
H	-2.19635300	4.92548600	1.82520800
H	-1.43116400	3.81133400	3.94701400

TS III

E_{vacuum} = -26301.5360

E_{sol} = -26301.5515

$-TS$ = -0.1517

G_{sol} = -26300.8214

C	-2.53075100	2.62745600	0.24747800
C	-2.90000100	1.63605600	1.30655500
C	-4.27124000	0.68113300	-0.35277900
C	-4.48767600	1.82318800	-0.97423700
C	-3.71045700	3.07055000	-0.62848200
C	-2.05287300	0.66482200	1.77945500
H	-5.45308800	-0.76679900	1.59394200
H	-2.09252200	3.53150300	0.71267600
C	-4.62710500	4.04502700	0.12079800
C	-3.18475800	3.73933200	-1.89904000
H	-4.01146100	4.07228100	-2.54715500

H	-2.58162300	4.62804300	-1.64668900
H	-5.27412800	1.91090600	-1.74188700
H	-1.75991700	2.17923700	-0.39673400
O	-0.94402100	0.31754900	1.14547700
Sm	0.85763000	-0.04460900	0.09751900
O	-0.05839700	-2.32851700	0.06950100
C	-1.40668000	-2.59515600	-0.39863000
C	-1.74775400	-3.97750200	0.12762700
C	-0.37873400	-4.64340000	0.22385600
C	0.49066700	-3.49833100	0.70424900
I	-0.35728800	0.51117300	-2.67550200
I	2.46578400	-0.76820200	2.62639000
O	3.02047200	1.06357300	-0.77549700
C	3.37850800	1.22207900	-2.16320900
C	4.54331400	2.19536700	-2.16896800
C	5.22391300	1.88027100	-0.84072600
C	4.03325300	1.64593900	0.06925400
O	1.00328600	2.37717400	0.64535900
C	1.03747100	3.47484300	-0.28932200
C	0.71330700	4.72123700	0.52283300
C	1.12251000	4.31709200	1.93734000
C	0.70757300	2.86097800	1.97220500
O	2.42057200	-1.69129300	-1.16019200
C	2.01890000	-2.49219100	-2.29140500
C	3.29709000	-3.12588100	-2.80740700
C	4.09917800	-3.29813100	-1.52157700
C	3.77382600	-2.01322100	-0.78382100
H	3.66771700	0.23462000	-2.56112100
H	2.48785600	1.56145200	-2.70889000
H	3.64173200	2.59129800	0.48197700
H	4.21399000	0.95489200	0.90367400
H	4.17743600	3.23502800	-2.17511800
H	5.19400700	2.06103600	-3.04396800
H	5.87634500	2.68517200	-0.47536400
H	5.83010800	0.96376100	-0.92646100
H	2.05061500	3.51389900	-0.72045300
H	0.32100800	3.27005600	-1.09782300
H	-0.37043000	2.73910800	2.16277100
H	1.27014100	2.23639500	2.67956800
H	-0.36620100	4.93098500	0.48492700
H	1.24452000	5.60711500	0.14836700
H	0.63000400	4.91069600	2.71958800
H	2.21272600	4.40928500	2.07097500
H	1.30101100	-3.25188900	-1.94091600
H	1.50666600	-1.83353500	-3.00583100
H	4.43253800	-1.18834800	-1.10500800
H	3.81297800	-2.08368700	0.31204700
H	3.81759600	-2.43998300	-3.49551200
H	3.10945600	-4.06664800	-3.34293200
H	5.17837100	-3.42466500	-1.68433400
H	3.73550200	-4.17107500	-0.95534300
H	-2.05966600	-1.79681700	-0.02727600
H	-1.39131000	-2.55094800	-1.49867600
H	1.54767300	-3.57255800	0.41554400
H	0.44093500	-3.36440100	1.79678600
H	-2.45155200	-4.50107500	-0.53124300
H	-2.21000300	-3.90445500	1.12318500
H	-0.04589200	-4.99151900	-0.76808700
H	-0.35375700	-5.49950900	0.91209000
H	-2.55219400	3.04459300	-2.47237300
H	-6.03870600	-3.17512100	1.48098900
H	-5.50790500	4.30228400	-0.48888000
H	-4.99224800	3.61005200	1.06341600
H	-4.09461900	4.98201400	0.35474500
H	-3.74271300	1.88308900	1.95865200
C	-4.63856400	-0.68788900	-0.40721100
C	-4.35186100	-1.45389100	-1.56254100
C	-4.69055000	-2.79943400	-1.62270900
C	-5.29889100	-3.42922700	-0.53302500
C	-5.56636000	-2.69104300	0.62305700
C	-5.24115000	-1.34227700	0.69082500
H	-3.85635500	-0.96285300	-2.40221700
H	-4.47732800	-3.36720200	-2.53193000
H	-5.56152000	-4.48778800	-0.58303300
H	-2.97218200	-1.94726400	2.17342500
C	-2.33349100	-0.06771900	3.07286000
H	-1.23326000	-1.86650300	2.58293500
C	-1.36787100	0.41397100	4.15898500
C	-2.24374100	-1.58143600	2.91217100
H	-0.32734000	0.19214500	3.87355400
H	-1.46091300	1.49933400	4.32030800
H	-3.36116600	0.19691400	3.37476000
H	-2.44294300	-2.08532600	3.87093900
H	-1.57068700	-0.09246300	5.11620100

TS III'

E_{vacuum} = -26301.5325
E_{sol} = -26301.5482
$-TS$ = -0.1533
G_{sol} = -26300.8198

C	6.58874600	-2.08650300	0.92956900
C	6.50624400	-2.91421600	-0.19400700
C	5.83816800	-2.45579200	-1.33153300
C	5.24723900	-1.19905900	-1.34361300
H	6.05842700	-0.19235800	1.81187600
H	7.11720500	-2.43156100	1.82112000
C	2.10729100	0.13180800	1.53827000
C	2.88911000	1.24677000	1.35649800
C	2.45564700	2.39524400	0.50156400
C	3.61761400	3.15122600	-0.15746300
C	4.65047200	2.13296800	-0.58733500
C	4.61957600	0.88205500	-0.17505300
C	5.30706700	-0.35475600	-0.21083100
O	0.96080600	-0.02607700	0.89142800
Sm	-0.97606000	-0.09383400	0.03087700
O	-1.49662500	0.57247300	2.35469900
C	-0.81602200	1.68391700	2.98889600
C	-1.11284200	1.54838800	4.47492500
C	-2.42354900	0.76660600	4.48875500
C	-2.21585900	-0.19404500	3.33553400
I	-1.23065500	2.89456300	-0.74716000
I	-1.32601800	-3.07502100	0.68533300
O	-2.29508400	-0.57688000	-2.12547300
C	-3.16199200	0.33982100	-2.82493400
C	-3.34907400	-0.25864400	-4.20676400
C	-3.26537800	-1.75324600	-3.91530700
C	-2.16260100	-1.80235600	-2.87559200
O	0.51311700	-0.48289700	-1.89633000
C	0.71140500	0.37237300	-3.04215900
C	2.04296600	-0.05522700	-3.63948700
C	2.12631900	-1.51989800	-3.21997100
C	1.58995900	-1.44432000	-1.80761400
O	-3.53611400	0.03347000	0.41631600
C	-4.24422300	1.20660900	0.86826700

C	-5.69942900	0.94316400	0.53694000
C	-5.79327000	-0.56397700	0.74892700
C	-4.46898500	-1.04363800	0.18258700
C	3.10683100	3.94227700	-1.36207900
H	-4.11726500	0.40031500	-2.27593800
H	-2.69266500	1.33258700	-2.81034500
H	-1.16279200	-1.81742900	-3.34083800
H	-2.23120000	-2.63928400	-2.16726900
H	-2.52734700	0.04871900	-4.87387700
H	-4.29574600	0.05130400	-4.67029500
H	-3.03395300	-2.36504200	-4.79802300
H	-4.21438200	-2.11627500	-3.48811200
H	-0.12885800	0.20233600	-3.73643500
H	0.68087900	1.41860100	-2.70697100
H	2.34388400	-1.06594000	-1.10062400
H	1.16459300	-2.37883400	-1.41731700
H	2.86447700	0.51653800	-3.17824800
H	2.07934900	0.10103800	-4.72629300
H	3.14082500	-1.93711900	-3.25754700
H	1.47356600	-2.14579700	-3.85069000
H	-4.09235200	1.31238000	1.95600100
H	-3.80425700	2.07830900	0.36586300
H	-4.52791900	-1.21680000	-0.90456300
H	-4.06626900	-1.95099100	0.65225100
H	-5.90642700	1.19996800	-0.51480400
H	-6.38418500	1.52390500	1.17019200
H	-6.65280500	-1.03105300	0.24871400
H	-5.85983600	-0.79520300	1.82448700
H	0.25333600	1.61834400	2.74362500
H	-1.22597600	2.60623400	2.55030600
H	-3.13765100	-0.55576900	2.86205500
H	-1.60895800	-1.06655800	3.62680500
H	-1.18112000	2.52572300	4.97156800
H	-0.32310800	0.96603200	4.97353500
H	-3.27639000	1.43455000	4.28450300
H	-2.61306400	0.24641000	5.43769900
C	6.00050100	-0.83013600	0.92796200
H	6.96202700	-3.90570300	-0.18355600
H	5.78273500	-3.08674600	-2.22203300
H	4.73952900	-0.83411900	-2.23734100
H	5.48853000	2.50295200	-1.20206500
H	3.70181000	1.39166500	2.07290200
C	4.27842700	4.11311000	0.83852000
H	1.84036500	3.11661300	1.07041900
H	1.79302700	2.00143800	-0.28259600
H	5.16322400	4.59710200	0.39485100
H	3.57298500	4.90532900	1.13763400
H	4.60826900	3.58324200	1.74524500
H	3.91842800	4.51762700	-1.83636700
H	2.67617600	3.26828200	-2.11906000
H	2.31811200	4.64866900	-1.05872200
H	0.54505900	-1.48950000	3.15022700
C	2.53312000	-1.00768300	2.43387100
H	1.42238600	-0.31967900	4.18373400
C	2.72340500	-2.29677200	1.63361400
C	1.52970500	-1.22481300	3.56511700
H	1.76955200	-2.61454900	1.18603900
H	3.46900600	-2.16803900	0.83603900
H	3.50451200	-0.72090300	2.87061400
H	1.85072300	-2.04989000	4.21998500
H	3.07258900	-3.10732000	2.29247900

Int IV

E_{vacuum} = -26301.5835

E_{sol} = -26301.5983

$-TS$ = -0.1509

G_{sol} = -26300.8644

C	-2.821806	2.564302	0.633062
C	-3.297533	1.168050	1.090813
C	-3.898657	0.622775	-0.205560
C	-4.099844	1.607529	-1.093061
C	-3.691921	2.964563	-0.574470
C	-2.228497	0.319488	1.714790
C	-4.937476	3.742536	-0.131038
H	-3.556208	4.015942	-2.479885
H	-2.561304	4.723703	-1.189617
H	-2.054107	3.198952	-1.986122
C	-2.918377	3.770738	-1.614965
H	-5.301660	-3.509511	1.392218
H	-4.575929	1.480111	-2.068841
H	-1.782160	2.446261	0.293435
O	-1.063584	0.212568	1.075132
Sm	0.772127	-0.029918	0.084576
O	0.036068	-2.384671	0.160783
C	-1.303398	-2.746911	-0.262709
C	-1.529423	-4.152282	0.266608
C	-0.112608	-4.710894	0.354486
C	0.667155	-3.496532	0.818034
I	-0.439485	0.436392	-2.709239
I	2.420825	-0.579422	2.642456
O	2.914096	1.153470	-0.830097
C	3.261085	1.297273	-2.220454
C	4.384659	2.316965	-2.250434
C	5.090880	2.042758	-0.926586
C	3.920714	1.765562	-0.000716
O	0.865286	2.409825	0.591299
C	0.872667	3.485377	-0.368066
C	0.377797	4.704179	0.390147
C	0.859953	4.415510	1.808843
C	0.618463	2.922890	1.919219
O	2.429954	-1.622021	-1.137631
C	2.066919	-2.470232	-2.246034
C	3.370291	-3.079022	-2.729518
C	4.169169	-3.174461	-1.433537
C	3.792473	-1.874916	-0.747484
H	3.590536	0.315210	-2.601381
H	2.355685	1.588080	-2.769795
H	3.504746	2.695876	0.422595
H	4.139065	1.080435	0.829682
H	3.976032	3.340554	-2.263002
H	5.031366	2.200042	-3.130954
H	5.713029	2.877093	-0.574509
H	5.734696	1.152194	-1.012125
H	1.907058	3.620466	-0.726049
H	0.241782	3.191270	-1.218620
H	-0.421886	2.689599	2.189023
H	1.292758	2.396059	2.608272
H	-0.721121	4.748568	0.363006
H	0.770861	5.640615	-0.029106
H	0.318107	4.980807	2.579332
H	1.933875	4.642973	1.908471
H	1.368939	-3.240033	-1.878107
H	1.542092	-1.851240	-2.986617
H	4.420768	-1.039405	-1.100945

H	3.830740	-1.899578	0.350631
H	3.872443	-2.402363	-3.440008
H	3.219160	-4.045417	-3.229858
H	5.253272	-3.268329	-1.585607
H	3.832871	-4.038048	-0.836789
H	-2.001133	-2.003186	0.138747
H	-1.327003	-2.696288	-1.362119
H	1.727947	-3.495333	0.534865
H	0.600461	-3.351887	1.908387
H	-2.196347	-4.729824	-0.386255
H	-1.987639	-4.113637	1.266728
H	0.237537	-5.039750	-0.638067
H	-0.015472	-5.556970	1.048835
H	-5.629800	3.895917	-0.974258
H	-4.696992	-1.131245	1.697994
H	-5.484254	3.201147	0.656614
H	-2.844999	3.309285	1.444232
H	-4.659804	4.734781	0.261826
H	-4.109955	1.266736	1.835140
C	-4.251109	-0.787512	-0.388275
C	-4.187954	-1.388226	-1.655012
C	-4.529259	-2.726302	-1.827224
C	-4.932813	-3.496842	-0.735871
C	-4.988383	-2.915851	0.530574
C	-4.645217	-1.577231	0.703767
H	-3.828089	-0.800102	-2.501054
H	-4.468536	-3.175131	-2.821219
H	-5.198791	-4.547424	-0.871286
H	-0.750466	-1.665712	2.958577
C	-2.290686	-0.151793	3.141813
H	-2.403931	-2.274663	2.661109
C	-1.500293	0.763564	4.087253
C	-1.800762	-1.593113	3.280534
H	-0.431315	0.744401	3.823966
H	-1.858664	1.803872	4.028671
H	-3.353135	-0.113721	3.445043
H	-1.856632	-1.932785	4.326869
H	-1.593139	0.431186	5.134291

Int IV'

E_{vacuum} = -26301.5777

E_{sol} = -26301.5921

$-TS$ = -0.1510

G_{sol} = -26300.8582

C	5.49224400	-2.26540100	1.62893600
C	6.07734100	-2.99662700	0.59771500
C	6.01147900	-2.50496700	-0.70754300
C	5.36412400	-1.30377000	-0.97339800
H	4.41099300	-0.49219200	2.18135700
H	5.53911000	-2.63235500	2.65648700
C	2.16176300	0.71201300	1.50453900
C	3.23466300	1.48687300	0.78200600
C	2.70825100	2.64811900	-0.08498800
C	3.75588400	2.89072500	-1.18621500
C	4.39289200	1.52766600	-1.27724200
C	4.13497400	0.74552100	-0.21473400
C	4.77068300	-0.55084000	0.05515600
O	1.01207500	0.44665700	0.87595000
Sm	-0.86056800	-0.09898700	0.05398100
O	-1.54411900	1.99607700	1.20299600
C	-0.81309700	3.22999200	1.01700500
C	-1.27717600	4.14905500	2.13427800
C	-2.69057700	3.64433000	2.40976400
C	-2.51796700	2.14632000	2.25294600
I	-0.47222000	1.45189200	-2.58980300
I	-1.94232600	-1.64795900	2.49350100
O	-2.13390700	-1.85406200	-1.38436100
C	-2.67513400	-1.65006100	-2.70321100
C	-2.94734100	-3.04053000	-3.24632700
C	-3.30934200	-3.80610500	-1.97806700
C	-2.33615900	-3.22172700	-0.97109700
O	0.53719700	-1.99000500	-0.66312200
C	0.84835300	-2.27980500	-2.04095700
C	2.30416700	-2.69429100	-2.03064000
C	2.41638600	-3.41506900	-0.69128100
C	1.56513200	-2.53718200	0.20472800
O	-3.39864000	0.48818800	-0.34639100
C	-3.87210500	1.71513600	-0.93746500
C	-5.35013300	1.49428800	-1.19985500
C	-5.73187900	0.56339600	-0.05412200
C	-4.51773000	-0.34276200	0.01907900
C	3.13847800	3.34995800	-2.50345800
H	-3.60218900	-1.05778000	-2.61273600
H	-1.95254900	-1.06139300	-3.28360800
H	-1.36099900	-3.73603500	-0.99100800
H	-2.69745300	-3.21214500	0.06602900
H	-2.03574900	-3.46042000	-3.70134200
H	-3.73933600	-3.04347000	-4.00788900
H	-3.20574500	-4.89575600	-2.07314000
H	-4.34845600	-3.58849600	-1.68166100
H	0.19309000	-3.10059500	-2.38252900
H	0.64018900	-1.37845200	-2.63457600
H	2.13795800	-1.69575500	0.61792200
H	1.05978300	-3.06919000	1.02292200
H	2.94691200	-1.80172900	-2.03392000
H	2.56826500	-3.32020100	-2.89433300
H	3.44854000	-3.48782700	-0.32434600
H	1.99529600	-4.43132100	-0.76384200
H	-3.70772300	2.53445900	-0.21762300
H	-3.26895500	1.91335000	-1.83363100
H	-4.59185000	-1.17113600	-0.70568600
H	-4.31449100	-0.76695600	1.01180900
H	-5.49820000	0.99052100	-2.16898500
H	-5.91667600	2.43558100	-1.21626300
H	-6.66155300	0.00353100	-0.22616800
H	-5.84592000	1.13295000	0.88281500
H	0.25714800	2.99341900	1.04850600
H	-1.06121400	3.61687800	0.01571800
H	-3.42299100	1.60626200	1.94721400
H	-2.12779700	1.67308700	3.16824700
H	-1.23821300	5.20712400	1.84162000
H	-0.64719200	4.01515500	3.02693600
H	-3.39467700	4.03492900	1.65682900
H	-3.06744500	3.91726100	3.40501200
C	4.84813000	-1.05904400	1.36094300
H	6.58140700	-3.94219600	0.80679800
H	6.46365300	-3.06728500	-1.52748100
H	5.30959900	-0.94073400	-2.00159500
H	5.08927600	1.27833000	-2.08281700
H	3.90486000	1.88405100	1.56867200
C	4.81778700	3.90172500	-0.72990900
H	2.49988400	3.55348300	0.50664100
H	1.77091300	2.31551200	-0.55560900
H	5.64536400	3.96249600	-1.45483100

H	4.37775100	4.90822600	-0.63675300
H	5.24589400	3.62062300	0.24498300
H	3.91283500	3.48237600	-3.27716500
H	2.39772600	2.62390800	-2.86810600
H	2.62508500	4.31696300	-2.37677400
H	0.14717200	1.39837100	3.26814600
C	2.15404700	0.62674000	3.01170700
H	1.41049200	2.66108000	3.32247700
C	1.85060900	-0.78301300	3.51924900
C	1.16465200	1.62853500	3.61862000
H	0.85718400	-1.11061500	3.17902100
H	2.59186800	-1.51045400	3.15476300
H	3.16687200	0.90921300	3.35313100
H	1.16482300	1.57826100	4.71939700
H	1.85232400	-0.81417800	4.62064600

Product

E_{vacuum} = -26301.5970

E_{sol} = -26301.6179

$-TS$ = -0.1555

G_{sol} = -26300.8874

C	-3.03143400	2.54726300	0.66146200
C	-3.51706500	1.15322200	1.10819800
C	-4.00251100	0.55477200	-0.20753300
C	-4.16495200	1.51856900	-1.12449500
C	-3.82875200	2.89476500	-0.61463600
C	-2.39787500	0.33263600	1.71911600
C	-5.11570500	3.65796400	-0.27843500
H	-3.58845800	3.93883200	-2.51036800
H	-2.63764200	4.62949000	-1.17712100
H	-2.12707100	3.09210000	-1.95274300
C	-2.99299900	3.68415400	-1.61932100
H	-5.23855000	-3.66389700	1.32109000
H	-4.54792700	1.34927900	-2.13318200
H	-1.96696200	2.45640100	0.39968700
O	-1.37606800	0.13995500	1.07570500
Sm	0.85721200	-0.03618800	-0.00587800
O	0.06160300	-2.48662800	0.24770400
C	-1.17703100	-2.99478200	-0.28062500
C	-1.34539600	-4.37936700	0.32472100
C	0.09939700	-4.80780200	0.56295200
C	0.73520200	-3.50156300	1.00260700
I	-0.47039700	0.34356700	-2.89943900
I	2.31462400	-0.36076200	2.85321500
O	2.96663800	1.28533500	-0.83723400
C	3.37906000	1.34230800	-2.21221600
C	4.53256300	2.33003400	-2.24441200
C	5.16568700	2.11296400	-0.87260400
C	3.94093900	1.92062200	0.00443700
O	0.57622000	2.50461800	0.54068700
C	0.69807300	3.52664700	-0.46239200
C	0.40832400	4.83179500	0.25629300
C	0.95746500	4.54326800	1.65075200
C	0.54213800	3.09654000	1.85094300
O	2.61827800	-1.64875500	-1.06029800
C	2.32098000	-2.54878900	-2.14053000
C	3.65241800	-3.17328800	-2.52088200
C	4.37826700	-3.19684000	-1.17827600
C	3.95211600	-1.86825400	-0.57920700
H	3.69795000	0.33220400	-2.52329700
H	2.50797500	1.62466400	-2.82072800
H	3.53509800	2.89116300	0.34385900
H	4.09518800	1.28642800	0.88925800
H	4.15400000	3.36169300	-2.33192200
H	5.21832600	2.14828200	-3.08353300
H	5.79602200	2.94833500	-0.53757400
H	5.78408200	1.20044500	-0.87520400
H	1.72555500	3.50291300	-0.86252800
H	0.00592100	3.28595000	-1.28079200
H	-0.48978000	3.02787100	2.23727700
H	1.20444600	2.51220300	2.50627900
H	-0.67777800	5.01166200	0.30265700
H	0.87736900	5.69504900	-0.23590000
H	0.55769700	5.20630400	2.43045300
H	2.05605500	4.63228300	1.65788000
H	1.60627000	-3.30600700	-1.77509900
H	1.83357900	-1.97247300	-2.94030000
H	4.59745900	-1.04493000	-0.93455700
H	3.92829300	-1.84045100	0.52034400
H	4.19043900	-2.52865000	-3.23498400
H	3.53588100	-4.16499400	-2.97944700
H	5.46972000	-3.28952000	-1.26501800
H	4.01309300	-4.03264400	-0.55911500
H	-1.98294600	-2.29903100	-0.01609000
H	-1.09673600	-3.02214900	-1.37966900
H	1.81020500	-3.42434100	0.78936200
H	0.58950600	-3.30924600	2.07873800
H	-1.90735700	-5.05410100	-0.33478900
H	-1.88713400	-4.31607100	1.28192200
H	0.55531600	-5.16208900	-0.37649300
H	0.20633700	-5.60173300	1.31516800
H	-5.74964800	3.77392500	-1.17139900
H	-4.84086100	-1.24966100	1.65378600
H	-5.70718500	3.12872100	0.48511600
H	-3.13746300	3.30685900	1.45111800
H	-4.88257200	4.66585700	0.10217600
H	-4.34700200	1.22271600	1.83265400
C	-4.25310900	-0.87777000	-0.39545200
C	-4.05274500	-1.47190500	-1.65172400
C	-4.29324400	-2.82873200	-1.83902600
C	-4.72164500	-3.62461200	-0.77560500
C	-4.90647000	-3.05046800	0.48093600
C	-4.67348600	-1.68997300	0.66854300
H	-3.65201900	-0.86729100	-2.46725200
H	-4.12260100	-3.27343000	-2.82164200
H	-4.90214500	-4.69124300	-0.92364500
H	-0.75402900	-1.43888000	3.07315000
C	-2.52310800	-0.18905200	3.13068000
H	-2.26477100	-2.29016900	2.63635400
C	-1.92032200	0.87883800	4.05565300
C	-1.82638000	-1.53100800	3.30162200
H	-0.83958500	0.96947900	3.86573000
H	-2.39256300	1.86388800	3.91302600
H	-3.59761000	-0.28158800	3.36343400
H	-1.92795600	-1.87997100	4.33991200
H	-2.06039000	0.58539700	5.10651800

Product'

E_{vacuum} = -26301.5932

E_{sol} = -26301.6148

$-TS$ = -0.1558

G_{sol} = -26300.8842

C	-5.01810200	2.66641100	1.54202100
C	-5.21193400	3.54180700	0.47654100

C	-5.08831800	3.06585800	-0.82997000
C	-4.76383500	1.73514700	-1.06644800
H	-4.57347500	0.65731200	2.15190000
H	-5.11500600	3.02233300	2.56955500
C	-2.39855800	-0.81116600	1.51484400
C	-3.56309800	-1.46095200	0.79099100
C	-3.11520100	-2.66251100	-0.06389100
C	-4.11143300	-2.73184300	-1.23986000
C	-4.51264900	-1.28321700	-1.34937900
C	-4.22600200	-0.56619800	-0.25057100
C	-4.56624300	0.83876900	-0.00296100
O	-1.43729100	-0.39690000	0.88394200
Sm	0.92975000	0.06173100	0.05221900
O	1.18907100	-2.15537400	1.41699000
C	0.72614900	-3.45277600	1.00636100
C	1.18129800	-4.40444100	2.09819600
C	2.49651300	-3.76720500	2.53743900
C	2.17243800	-2.28383000	2.46112400
I	0.30666300	-1.70151300	-2.56860000
I	1.99516900	1.76759000	2.55397200
O	2.16741500	1.68209200	-1.58333100
C	2.76644100	1.35083900	-2.84618400
C	3.08011700	2.68285400	-3.50469000
C	3.38800100	3.55965100	-2.29419300
C	2.34594000	3.08093300	-1.30007700
O	-0.57366200	2.05744600	-0.53116900
C	-1.19109700	2.17292800	-1.81191200
C	-1.60516400	3.64507900	-1.93328200
C	-1.52007000	4.18004200	-0.48941000
C	-1.29465300	2.92454800	0.34294100
O	3.43620400	-0.71217700	-0.15372000
C	3.85645500	-1.92850400	-0.78924200
C	5.26653200	-1.65611100	-1.27774200
C	5.79346500	-0.73433500	-0.18150800
C	4.57510400	0.12201800	0.12435300
C	-3.46672600	-3.27201400	-2.51232100
H	3.68673200	0.77011000	-2.65664900
H	2.06620900	0.70973400	-3.40000000
H	1.38044300	3.59327200	-1.44754700
H	2.63218900	3.18034400	-0.24275800
H	2.19455200	3.06542900	-4.03811100
H	3.90711900	2.61179100	-4.22477000
H	3.31274200	4.63731900	-2.49546900
H	4.40375100	3.35291700	-1.91873100
H	-0.45963700	1.83597300	-2.55825500
H	-2.06281900	1.49796300	-1.85152800
H	-2.25241200	2.45172700	0.62120900
H	-0.67698000	3.06592300	1.24016800
H	-2.62073000	3.72982500	-2.34417700
H	-0.93137000	4.19829300	-2.60339900
H	-2.42878900	4.71394600	-0.17967000
H	-0.66787800	4.86651000	-0.37679600
H	3.84842200	-2.74331500	-0.04223600
H	3.12925500	-2.16498600	-1.58000200
H	4.52046600	1.00611300	-0.53290600
H	4.50265600	0.46854300	1.16488100
H	5.23986000	-1.13132900	-2.24668400
H	5.85469900	-2.57561900	-1.40471800
H	6.66116900	-0.13297600	-0.48647600
H	6.08323100	-1.32328500	0.70383200
H	-0.36467200	-3.40234100	0.88201400
H	1.16631900	-3.69399300	0.02453900
H	3.02727500	-1.64417500	2.20366000
H	1.73451500	-1.90561900	3.39978100
H	1.29073900	-5.43595900	1.73585000
H	0.46082900	-4.40808300	2.93225700
H	3.29908700	-4.02630500	1.82832500
H	2.82037800	-4.07226900	3.54204900
C	-4.70140400	1.33079100	1.30323600
H	-5.45728600	4.58931000	0.66079800
H	-5.23705100	3.74311300	-1.67370500
H	-4.64197700	1.38184500	-2.09207700
H	-5.06217700	-0.89942000	-2.21247800
H	-4.31209500	-1.76503300	1.54343200
C	-5.34654400	-3.56871500	-0.87828600
H	-3.06598000	-3.59974400	0.51117400
H	-2.11636400	-2.43783200	-0.47008400
H	-6.11319400	-3.49874400	-1.66586000
H	-5.07346700	-4.63071300	-0.76937500
H	-5.80425600	-3.23011900	0.06460600
H	-4.18719000	-3.27264700	-3.34615600
H	-2.58927400	-2.67412300	-2.79978700
H	-3.13001200	-4.31040400	-2.36280300
H	-0.63829200	-2.00539900	3.15546200
C	-2.40931900	-0.76696600	3.02781800
H	-2.15389600	-2.95010400	3.12814400
C	-1.76487600	0.49744200	3.57457000
C	-1.68015700	-2.03007300	3.50610300
H	-0.71359700	0.58796200	3.26193800
H	-2.29309500	1.39729500	3.22508800
H	-3.45917700	-0.83343600	3.36124100
H	-1.68251300	-2.07255500	4.60525800
H	-1.79189200	0.48997000	4.67437600

Isopropyl Bicyclo[1.1.0]Butyl Ketone Reactant

E_{vacuum} = -25952.6958

E_{sol} = -25952.7160

$-TS$ = -0.1375

G_{sol} = -25952.1447

C	-3.34512600	0.18138700	-0.30709300
O	-2.12591500	0.00946400	-0.26130400
Sm	0.35497500	0.00399200	-0.11651400
O	-0.20922800	-2.11777200	-1.53710400
C	-1.19298400	-2.13144300	-2.57018400
C	-2.32999800	-3.00588500	-2.03521300
C	-1.65913100	-3.84250200	-0.92537300
C	-0.19017600	-3.44214200	-1.00773100
I	0.55113600	1.78408900	-2.74990600
I	0.18417100	-1.79764400	2.58731300
O	2.40514100	1.25936500	0.88993600
C	3.50477200	1.74330600	0.09887300
C	4.48343100	2.34946300	1.09196200
C	4.23057800	1.50644900	2.33915400
C	2.72408600	1.33402600	2.28645100
O	-0.45195200	2.02742500	1.29456500
C	-0.04353300	3.38187600	1.03475800
C	-0.78850000	4.23743500	2.04866900
C	-0.98682400	3.26325900	3.20727300
C	-1.27272400	1.97120300	2.46580400
O	2.56210300	-1.23185100	-0.77243500
C	2.89793800	-1.56676300	-2.13317600
C	4.09080200	-2.50907900	-2.04403600

C	3.90084200	-3.14427600	-0.66897700
C	3.39489900	-1.96303700	0.13544400
H	3.94047800	0.88551400	-0.43936300
H	3.10952900	2.44863200	-0.64622600
H	2.20736000	2.20782700	2.72535100
H	2.34377700	0.42336900	2.77177600
H	4.23186400	3.40439700	1.28852900
H	5.52137600	2.31038700	0.73342600
H	4.56975700	1.98036800	3.27069100
H	4.72910400	0.52741600	2.25142500
H	1.04900000	3.43668500	1.16962400
H	-0.26990400	3.62081500	-0.01433200
H	-2.33453700	1.91672600	2.15942900
H	-1.01526600	1.05311000	3.01376500
H	-1.76444100	4.55183700	1.64516400
H	-0.22900600	5.14256600	2.32263200
H	-1.79784200	3.54923000	3.89147600
H	-0.06014200	3.16535000	3.79581500
H	2.01564100	-2.04831600	-2.58220100
H	3.09750100	-0.63554500	-2.68308200
H	4.23201200	-1.31702900	0.46226000
H	2.78724000	-2.22685600	1.01351800
H	5.03617400	-1.94349700	-2.07170500
H	4.10913900	-3.23583700	-2.86796900
H	4.81924400	-3.58088100	-0.25266900
H	3.13056700	-3.93150900	-0.70736600
H	-1.46145800	-1.08950000	-2.78356800
H	-0.74261200	-2.56843900	-3.48045500
H	0.37437900	-4.09619300	-1.70013100
H	0.31416800	-3.40704600	-0.03255400
H	-2.75924500	-3.62830000	-2.83306200
H	-3.13924100	-2.39047500	-1.61899700
H	-1.79679100	-4.92447700	-1.05901800
H	-2.05487500	-3.56959900	0.06266000
C	-3.93046100	1.30927800	-1.12774700
H	-4.99295000	1.42057900	-0.85900300
C	-3.19006200	2.61709500	-0.87123600
H	-3.62800100	3.42081100	-1.48151800
H	-3.25461200	2.91713000	0.18589700
H	-2.13027800	2.51611800	-1.14865100
C	-3.84028900	0.90964000	-2.60521200
H	-2.78393400	0.85539300	-2.91141700
H	-4.32184600	-0.06186200	-2.79733100
H	-4.33627400	1.66683400	-3.23035900
C	-4.22463200	-0.74083300	0.39346100
C	-4.64213900	-0.55183000	1.85101900
H	-4.36565100	0.30259200	2.46497500
C	-3.79929000	-1.71084100	1.46477800
H	-2.74428000	-1.69179100	1.76109100
H	-4.27032000	-2.70154900	1.44709400
C	-5.67480700	-0.62068500	0.78685900
H	-6.28272400	-1.53074600	0.70936900
H	-6.20301400	0.30300100	0.53583700

Int I

E_{vacuum} = -25952.6795

E_{sol} = -25952.6930

$-TS$ = -0.1339

G_{sol} = -25952.1199

C	3.20891700	-0.13494900	-0.50741300
O	1.88431000	-0.02795200	-0.35738100
Sm	-0.20324300	0.01845500	-0.09833000
O	0.11374200	2.01979600	-1.55768000
C	1.16650800	2.05264600	-2.53461500
C	2.18186000	3.05523700	-1.99201000
C	1.36376300	3.91648500	-1.00685600
C	-0.05579300	3.38176400	-1.14533000
I	-0.74886900	-1.86288300	-2.47076000
I	-0.19539700	1.88458300	2.39126500
O	-2.26493200	-1.09136400	1.01883900
C	-3.32769600	-1.76293300	0.31369200
C	-4.07624100	-2.54725100	1.37377000
C	-3.93775900	-1.63527600	2.58788400
C	-2.51499000	-1.12736400	2.43953700
O	0.41474200	-1.85193000	1.40295900
C	0.05255600	-3.23787000	1.23540700
C	1.15797000	-4.02862000	1.91302900
C	1.61414400	-3.06954700	3.00920900
C	1.54048700	-1.73108200	2.30134900
O	-2.43394000	1.14247000	-0.74958800
C	-2.86811600	1.34874800	-2.11356300
C	-4.20798900	2.05951300	-2.01355300
C	-4.07797300	2.80827100	-0.69111200
C	-3.34443000	1.78947800	0.15674500
H	-3.97084300	-1.00089700	-0.15926400
H	-2.87626200	-2.37093900	-0.48121000
H	-1.78248600	-1.81078600	2.90079800
H	-2.34352700	-0.12064400	2.84337500
H	-3.58013300	-3.51397300	1.55862600
H	-5.11740500	-2.74923800	1.08675600
H	-4.09976200	-2.14509900	3.54755600
H	-4.65349000	-0.79974900	2.52216300
H	-0.92291500	-3.39835000	1.72477000
H	-0.05406400	-3.43805600	0.15964900
H	2.43864600	-1.52513500	1.69978700
H	1.34501300	-0.87263000	2.95874200
H	1.97882900	-4.21813800	1.20551600
H	0.80118300	-4.99539800	2.29404400
H	2.62363000	-3.28449800	3.38584100
H	0.91723800	-3.09519200	3.86264000
H	-2.10499900	1.96234700	-2.61603000
H	-2.90753200	0.37030300	-2.61167900
H	-4.04004200	1.03518700	0.56638300
H	-2.75493200	2.20991200	0.98292500
H	-5.03130600	1.32923900	-1.95698700
H	-4.39516200	2.71321300	-2.87649300
H	-5.04091900	3.10563500	-0.25351900
H	-3.46165000	3.71318300	-0.81625900
H	1.54740300	1.03276000	-2.64466800
H	0.72596900	2.38359800	-3.49188900
H	-0.62411900	3.91366900	-1.93124700
H	-0.62615900	3.38197800	-0.20818600
H	2.62437200	3.64744800	-2.80515700
H	2.99205800	2.52985100	-1.46942400
H	1.41293800	4.99153400	-1.22892000
H	1.71331900	3.76451600	0.02391400
C	3.74229400	-1.22274500	-1.39900700
H	4.84190100	-1.15369800	-1.36312000
C	3.33227400	-2.61368300	-0.90643300
H	3.72016500	-3.40235600	-1.57097900
H	3.71533700	-2.80553700	0.10865100
H	2.23436300	-2.69886100	-0.89223500
C	3.30772500	-1.03315500	-2.85513100
H	2.21218100	-1.10279500	-2.94331500

H	3.63413100	-0.05438700	-3.23968900
H	3.74098000	-1.81442500	-3.50017200
C	4.06324400	0.72550900	0.26071900
C	4.57763700	0.70420400	1.74058300
H	4.34560500	-0.05973200	2.47846900
C	3.70650700	1.80555200	1.23674100
H	2.66697300	1.82807700	1.57856000
H	4.16336000	2.80123000	1.10944800
C	5.52040800	0.62204400	0.58489500
H	6.17174700	1.48757400	0.38411500
H	6.00429800	-0.34308300	0.39899400

TS I

E_{vacuum} = -25952.6792

E_{sol} = -25952.6934

$-TS$ = -0.1314

G_{sol} = -25952.1187

C	-3.24108900	-1.96581100	-0.39486700
O	-2.37741000	-1.34904000	0.57944700
C	-2.84551600	-1.64385700	1.91194100
C	-3.85997800	-2.75823100	1.73921300
C	-4.46453000	-2.41614800	0.38148900
Sm	-0.20348400	-0.06576300	0.08346400
O	0.41325300	1.96575700	-1.20672600
C	0.01494700	3.32542900	-0.93544600
C	1.14802700	4.18920300	-1.45845200
C	1.67333900	3.35407200	-2.62290100
C	1.57598200	1.94632100	-2.06795300
O	0.17174500	-2.19580700	1.34503200
C	1.22520200	-2.32908300	2.31216900
C	2.24664800	-3.26476500	1.66832800
C	1.44232800	-3.99714900	0.57424800
C	0.01686600	-3.50221900	0.77632100
I	-0.90012900	1.58152100	2.58434900
I	-0.18660500	-1.60616600	-2.59460900
O	-2.28947900	1.07151500	-0.94865000
C	-3.46908600	1.47396200	-0.24632000
C	-3.82990900	2.79796500	-0.88794700
C	-3.49833500	2.55153200	-2.36591500
C	-2.48548300	1.39538700	-2.33499900
H	-4.26528400	0.72086300	-0.39949300
H	-3.21244500	1.53327900	0.81907300
H	-1.49900100	1.64168300	-2.74585800
H	-2.85266600	0.49926800	-2.86073900
H	-3.19474600	3.59041900	-0.46398900
H	-4.87912800	3.07823400	-0.72143800
H	-3.08866600	3.44700600	-2.85426000
H	-4.39711200	2.25787200	-2.92740000
H	-0.92491100	3.52268000	-1.47713200
H	-0.17049700	3.41772200	0.14415700
H	2.44793700	1.67652700	-1.45410500
H	1.41424500	1.16436100	-2.82292300
H	1.92550100	4.30621000	-0.68915900
H	0.80239300	5.18913800	-1.75482600
H	2.69979000	3.61172000	-2.91807700
H	1.02451800	3.46909300	-3.50630100
H	-1.97245000	-1.90925700	2.52132500
H	-3.29687700	-0.73010200	2.33089500
H	-3.44450800	-1.23144400	-1.18501600
H	-2.70478100	-2.81136200	-0.85467200
H	-4.59362100	-2.78439400	2.55645900
H	-3.35641800	-3.73757100	1.69704900
H	-5.18568600	-1.58885600	0.48367300
H	-4.97664100	-3.25936000	-0.10185400
H	1.60203600	-1.32533700	2.52933700
H	0.78661400	-2.76249500	3.22863200
H	-0.54454900	-4.12962200	1.49364500
H	-0.55273900	-3.39804200	-0.15526000
H	2.67190800	-3.95356500	2.41167500
H	3.06835300	-2.68528100	1.22715900
H	1.50571800	-5.09152500	0.64989500
H	1.79044700	-3.70304700	-0.42598700
O	1.87697500	0.00941000	0.40802200
C	3.19643100	0.11085900	0.60406300
C	3.67950000	1.11549200	1.61701400
C	4.07504200	-0.66178900	-0.18982900
H	4.78035900	1.06306600	1.62224700
C	3.26765300	2.53979300	1.23652200
C	3.18679900	0.78551500	3.02795800
C	3.74561900	-1.50956400	-1.37772800
C	5.53567200	-0.48528800	-0.45957000
H	3.61767600	3.26843000	1.98507800
H	3.68720200	2.82829000	0.25947600
H	2.17000500	2.61297800	1.18573900
H	2.08706900	0.82474700	3.07356400
H	3.52045200	-0.21683100	3.33834200
H	3.57368800	1.51345900	3.75881300
H	2.72281100	-1.44501000	-1.76439000
H	4.15398800	-2.53370900	-1.39966800
H	6.18567300	-1.36858000	-0.35211600
H	6.01109600	0.43545300	-0.10275300
C	4.70830300	-0.41004800	-1.70777800
H	4.44893900	0.51222100	-2.22415700

Int II

E_{vacuum} = -25952.6925

E_{sol} = -25952.7058

$-TS$ = -0.1339

G_{sol} = -25952.1336

O	1.844640	-0.173851	-0.416872
Sm	-0.231904	0.000613	-0.101780
O	0.124901	1.906062	-1.690232
C	1.160124	1.845035	-2.682140
C	2.239124	2.790721	-2.169451
C	1.458130	3.795832	-1.295648
C	0.017250	3.295466	-1.354324
I	-0.902778	-1.968421	-2.371840
I	-0.043067	2.030046	2.250557
O	-2.279881	-0.976261	1.134601
C	-3.390161	-1.645575	0.504202
C	-4.169877	-2.283352	1.638920
C	-3.936153	-1.292322	2.774439
C	-2.482968	-0.913195	2.560791
O	0.371782	-1.789653	1.509689
C	-0.028375	-3.170834	1.408288
C	1.050810	-3.959031	2.130180
C	1.534982	-2.959096	3.176550
C	1.502323	-1.656529	2.401544
O	-2.436026	1.168914	-0.749703
C	-2.908212	1.324556	-2.107975
C	-4.182708	2.149902	-2.009750
C	-3.966456	2.935727	-0.720230
C	-3.289046	1.897311	0.149484
H	-3.990569	-0.891464	-0.032262

H	-2.987330	-2.350248	-0.235335
H	-1.797034	-1.630855	3.042028
H	-2.213244	0.096985	2.897009
H	-3.743132	-3.267000	1.894102
H	-5.229432	-2.428430	1.387067
H	-4.115228	-1.712219	3.773791
H	-4.585749	-0.410089	2.654284
H	-1.010765	-3.280167	1.898447
H	-0.136773	-3.422741	0.343457
H	2.409217	-1.505432	1.795646
H	1.330323	-0.761968	3.015444
H	1.866294	-4.210858	1.436044
H	0.663654	-4.893431	2.559378
H	2.537112	-3.184490	3.566193
H	0.836477	-2.920534	4.028303
H	-2.116249	1.836733	-2.674472
H	-3.049615	0.324462	-2.540602
H	-4.027239	1.202280	0.589225
H	-2.659615	2.302448	0.953750
H	-5.062662	1.494262	-1.910261
H	-4.333710	2.783894	-2.894215
H	-4.894943	3.318955	-0.274993
H	-3.288297	3.787225	-0.891309
H	1.469853	0.799945	-2.774293
H	0.730114	2.185018	-3.641608
H	-0.562560	3.798341	-2.150421
H	-0.517429	3.369630	-0.399413
H	2.776603	3.272841	-2.997790
H	2.967344	2.232096	-1.565220
H	1.535817	4.830307	-1.658142
H	1.815866	3.770945	-0.256922
C	3.650798	-1.476524	-1.378630
H	4.751246	-1.482802	-1.307719
C	3.128654	-2.834014	-0.911183
H	3.483154	-3.640373	-1.572195
H	3.470822	-3.057358	0.111610
H	2.027737	-2.845474	-0.929569
C	3.261572	-1.224640	-2.833581
H	2.167209	-1.258963	-2.952279
H	3.628374	-0.246037	-3.180860
H	3.688717	-1.999240	-3.489507
C	3.163509	-0.371836	-0.466918
C	4.015074	0.363189	0.272465
C	5.530167	0.376325	0.436284
C	3.820789	1.487338	1.275622
H	6.113240	0.642170	-0.468653
H	5.983212	-0.549812	0.844746
H	3.363961	2.416406	0.880023
H	3.230653	1.231979	2.177544
C	5.316666	1.490584	1.426102
H	6.002990	2.077014	2.038189

PreTS II

E_{vacuum} = -26260.9526

E_{sol} = -26260.9669

$-TS$ = -0.1530

G_{sol} = -26260.2925

Sm	0.92579200	-0.09358000	0.04335400
O	2.72870500	0.88667600	-1.40480500
C	3.30358400	2.15872800	-1.06985300
C	2.68702500	3.13470200	-2.06533900
C	2.32279400	2.24358000	-3.27256800
C	2.74685200	0.84396000	-2.83748600
I	2.80673700	-0.30524400	2.46619200
I	-0.84389300	-0.37402200	-2.51728000
O	0.12117500	-2.49428700	0.55063100
C	0.84673000	-3.45655900	1.33971300
C	-0.18782300	-4.47882200	1.77139700
C	-1.13516100	-4.48699700	0.57568300
C	-1.17391900	-3.01880300	0.19200800
O	-1.05332200	-0.13348300	1.51580800
C	-1.15142600	-0.68345900	2.84394400
C	-2.35574200	-0.00494100	3.47211700
C	-3.24242600	0.25894100	2.25904200
C	-2.22534100	0.66167900	1.21141000
O	2.44012600	-1.90668300	-0.99554300
C	3.87642900	-1.95748200	-0.83617100
C	4.33883300	-3.14291100	-1.66786700
C	3.26743600	-3.21461300	-2.75141000
C	2.01903100	-2.89011000	-1.95711100
H	1.62844100	-3.91150200	0.70721600
H	1.33917300	-2.91917000	2.16165000
H	-1.94205900	-2.46326900	0.75465000
H	-1.33116100	-2.82876000	-0.87826800
H	-0.71330200	-4.13605600	2.67741600
H	0.25902900	-5.45868300	1.98906500
H	-2.13468200	-4.88152700	0.80540500
H	-0.71061300	-5.08892000	-0.24406900
H	-1.30135700	-1.77263400	2.75413100
H	-0.20015000	-0.50027300	3.36333000
H	-1.95294400	1.72579900	1.27406200
H	-2.51932100	0.43423300	0.17978700
H	-2.05556100	0.94259200	3.94431900
H	-2.83175500	-0.63466300	4.23646700
H	-3.99489900	1.04191000	2.42113600
H	-3.76902400	-0.65895700	1.95235600
H	4.28483800	-1.00361800	-1.20290400
H	4.09758100	-2.03756300	0.23685200
H	1.64413600	-3.78021600	-1.42067300
H	1.19478500	-2.45714600	-2.54041100
H	4.33201800	-4.06591300	-1.06595600
H	5.35581500	-3.00238500	-2.05910600
H	3.20708000	-4.19127800	-3.25113100
H	3.44001100	-2.44392000	-3.52001700
H	3.06941700	2.36312100	-0.02103800
H	4.39867000	2.07674600	-1.19175400
H	3.77644400	0.60414900	-3.16197600
H	2.06062700	0.05141200	-3.16016400
H	3.39015900	3.93822600	-2.32534300
H	1.78630000	3.59117400	-1.63216100
H	2.83114500	2.54542200	-4.19888800
H	1.23935600	2.25872800	-3.45640500
O	0.56211300	1.96681100	0.32655000
C	0.11515000	3.19370700	0.60027300
C	0.55938900	3.80471100	1.91159800
C	-0.70401200	3.84702000	-0.24507400
H	0.14513300	4.82642300	1.94042800
C	0.00130900	3.02306500	3.09985700
C	2.08106600	3.89860100	2.00025900
C	-1.34048200	3.46716100	-1.56953700
C	-1.44127500	5.17884500	-0.18869200
H	0.33717700	3.46455700	4.05128700
H	-1.09981100	3.02536400	3.09441100
H	0.35457800	1.98077200	3.07146400

H	2.52871700	2.89277700	2.02839700
H	2.49773800	4.45066000	1.14330000
H	2.38464700	4.42081900	2.92095400
H	-1.89083600	2.50807000	-1.59110400
H	-0.64562900	3.43034100	-2.43358000
H	-2.09036900	5.34785700	0.69274600
H	-0.80845400	6.08326100	-0.29676100
C	-2.13393000	4.74182100	-1.45578300
H	-2.71972600	5.30319300	-2.18772900
C	-4.69123300	2.46187000	-0.78813300
C	-5.02657900	1.30650500	-0.62183100
H	-4.35263300	3.47057700	-0.94883600
H	-3.77961800	-0.78696000	-1.70249700
C	-5.34763200	-0.06731900	-0.40020600
C	-6.38080700	-0.43308700	0.47740300
C	-6.63509200	-1.77564300	0.73741700
C	-5.86660300	-2.76769900	0.12753600
C	-4.84615000	-2.40983400	-0.75398700
C	-4.58571200	-1.07005700	-1.02284400
H	-6.97167400	0.34771300	0.95848100
H	-7.43859900	-2.05042800	1.42354600
H	-6.06864000	-3.82048800	0.33518300
H	-4.24606000	-3.18017900	-1.24321200

PreTS II'

E_{vacuum} = -26260.9463
E_{sol} = -26260.9620
$-TS$ = -0.1542
G_{sol} = -26260.2887

Sm	-1.03110900	-0.04217800	0.11277400
O	0.40823500	-1.92928400	0.90796100
C	1.66705600	-1.66253300	1.54711900
C	2.70297300	-1.98910200	0.48380300
C	2.01819300	-3.07585200	-0.36963400
C	0.60690100	-3.16709000	0.20909000
I	-1.49771100	1.08160900	2.94269500
I	-1.04082800	-1.36038700	-2.70624700
O	-3.57586400	0.27536700	-0.24382300
C	-4.56014900	0.33622100	0.80802400
C	-5.83896100	0.80039600	0.13560500
C	-5.69553300	0.20041900	-1.25911800
C	-4.21626800	0.39772800	-1.52969300
O	-1.59640100	2.11166800	-0.98902700
C	-2.36266100	3.17685700	-0.39252500
C	-1.90039500	4.44446000	-1.08866800
C	-1.51914600	3.92825700	-2.47329200
C	-0.87289200	2.59662600	-2.14375800
O	-2.41235100	-2.06109800	0.92373000
C	-2.31574200	-2.62779400	2.25087400
C	-3.23710100	-3.83779700	2.24843400
C	-3.23493100	-4.24590800	0.77851100
C	-3.24068100	-2.89424700	0.09487500
H	-4.66670200	-0.67261300	1.24118500
H	-4.18077300	1.00496300	1.59198400
H	-4.00463400	1.40372000	-1.93067100
H	-3.76512300	-0.34276300	-2.20359900
H	-5.86651200	1.90072500	0.07849500
H	-6.73727300	0.46634300	0.67283700
H	-6.32669900	0.68412700	-2.01726400
H	-5.94385800	-0.87329300	-1.24368600
H	-3.43190400	2.97729900	-0.57762400
H	-2.18503700	3.16317100	0.69245200
H	0.18852100	2.70154300	-1.87023100
H	-0.96568600	1.83592200	-2.93083400
H	-1.01796700	4.85872800	-0.57876000
H	-2.68235700	5.21612500	-1.10564700
H	-0.83691800	4.59596600	-3.01696600
H	-2.41903400	3.77976900	-3.09214900
H	-1.26286900	-2.90107800	2.41683500
H	-2.58901300	-1.84866100	2.97582700
H	-4.25988700	-2.46849500	0.06072600
H	-2.81889300	-2.88345000	-0.91968400
H	-4.25435200	-3.55194200	2.56097800
H	-2.88506600	-4.62676500	2.92711300
H	-4.09361700	-4.86795000	0.49056000
H	-2.31219500	-4.79365800	0.52810600
H	1.65643300	-0.61913300	1.87625800
H	1.74667700	-2.32458700	2.42845000
H	0.51337800	-3.99121700	0.94010700
H	-0.17991500	-3.25209600	-0.55032400
H	3.65396300	-2.31890900	0.92242300
H	2.90119800	-1.09699700	-0.12411700
H	2.53235500	-4.04547800	-0.31629200
H	1.97449100	-2.77173000	-1.42454300
O	0.87060300	0.85984500	-0.05513000
C	1.97153700	1.57340700	-0.29899300
C	2.37532900	2.58398700	0.75368300
C	2.66155800	1.42978900	-1.44787600
H	3.35764600	2.98346100	0.44862600
C	1.37756800	3.74144900	0.79428500
C	2.50957000	1.95280600	2.13730800
C	3.87332700	2.12728700	-2.05720700
C	2.43752900	0.57723500	-2.68436100
H	1.66870500	4.48601100	1.55185600
H	1.32171000	4.25021300	-0.18077400
H	0.37589500	3.36906900	1.05980900
H	1.53242500	1.59388600	2.49534000
H	3.21052400	1.10450300	2.13141900
H	2.87818500	2.69226900	2.86554900
H	4.84730000	1.93067300	-1.56509700
H	3.79282800	3.22624000	-2.17690100
H	2.57005800	-0.51289900	-2.53636100
H	1.45697400	0.69441800	-3.18385800
C	3.60608100	1.30466900	-3.28963800
H	4.16577700	1.17155900	-4.21622200
C	5.19270400	-1.70153900	-2.36720900
C	5.67537600	-1.30325300	-1.32688100
H	4.76315100	-2.04098600	-3.28933700
H	7.96213600	-2.12236600	-0.20854200
C	6.23847100	-0.82925500	-0.10324000
C	5.58043500	0.16490200	0.63982400
C	6.13419200	0.62755600	1.82867400
C	7.34225600	0.10760600	2.29273700
C	7.99825100	-0.88202900	1.56108700
C	7.45344800	-1.35005900	0.37009900
H	4.63199700	0.56102800	0.27258500
H	5.61484600	1.40099200	2.39771400
H	7.77319300	0.47405100	3.22646000
H	8.94374100	-1.29261800	1.92066900

TS II

E_{vacuum} = -26260.9429
E_{sol} = -26260.9575
$-TS$ = -0.1501

G_{sol} = -26260.2810

Sm	0.90876300	0.10098700	0.02849200
O	1.76661700	1.83620000	-1.56844700
C	1.66977700	3.23495300	-1.26450000
C	0.58114200	3.75268600	-2.19532300
C	0.63799600	2.78361800	-3.39580300
C	1.70426300	1.76516700	-2.99841200
I	2.86050400	0.98787500	2.23351500
I	-0.67881800	-1.12988000	-2.33050500
O	1.56979500	-2.32792100	0.57686300
C	2.77549000	-2.72669500	1.25785300
C	2.50862300	-4.13849600	1.74207000
C	1.61839200	-4.68334800	0.62975800
C	0.74816100	-3.48296100	0.30298500
O	-0.65051100	-0.95713700	1.63819700
C	-0.27133100	-1.40579400	2.95308300
C	-1.48336200	-1.14966500	3.82598400
C	-2.62934700	-1.38466600	2.84756100
C	-2.09180600	-0.79717500	1.55437700
O	3.05075200	-0.65540200	-1.21541900
C	4.30673000	0.06058100	-1.19249300
C	5.24628300	-0.73038800	-2.08832400
C	4.28038800	-1.38973200	-3.06751100
C	3.12522900	-1.74327500	-2.15350700
H	3.61179700	-2.69393800	0.53836600
H	2.97521500	-1.99244200	2.04972300
H	-0.14513900	-3.42755100	0.94659200
H	0.41823300	-3.42487400	-0.74290200
H	1.96279700	-4.11934500	2.69923500
H	3.43424500	-4.71172500	1.88960000
H	1.02471300	-5.55899100	0.92627300
H	2.22830700	-4.96613900	-0.24351600
H	-0.03280900	-2.48297500	2.90122300
H	0.62993100	-0.85310600	3.25493100
H	-2.30679800	0.27671800	1.45259500
H	-2.43713900	-1.31845800	0.65185600
H	-1.48101400	-0.10923200	4.18099700
H	-1.51097500	-1.81398700	4.70089500
H	-3.57146100	-0.90845400	3.14982200
H	-2.82166500	-2.46219300	2.72795700
H	4.11737300	1.07498900	-1.57532200
H	4.63431600	0.14245400	-0.14717400
H	3.32370600	-2.68008000	-1.60217900
H	2.14857600	-1.82649200	-2.64978000
H	5.78099000	-1.49893100	-1.50716400
H	5.99667200	-0.08878600	-2.57032800
H	4.69792600	-2.26832000	-3.57836700
H	3.95154000	-0.66900400	-3.83354400
H	1.44463400	3.32669400	-0.19852900
H	2.65184500	3.69601600	-1.47493800
H	2.69911000	2.03271900	-3.40049600
H	1.45679600	0.73252900	-3.27280800
H	0.76014000	4.79838300	-2.48165000
H	-0.39392100	3.69460300	-1.69168800
H	0.89867300	3.28428100	-4.33875700
H	-0.32681000	2.27657500	-3.53571500
O	-0.39651200	1.70216400	0.43513800
C	-1.36318600	2.58396300	0.71434200
C	-1.17944500	3.41950800	1.96221900
C	-2.44969200	2.71216300	-0.06662400
H	-2.05221700	4.09112900	2.02074100
C	-1.16289500	2.53953400	3.21014100
C	0.08345000	4.27552700	1.89637900
C	-2.89561900	2.02123500	-1.34085600
C	-3.68120600	3.60734400	-0.03140600
H	-1.03554600	3.14828900	4.11921000
H	-2.10369800	1.97544000	3.30684800
H	-0.32403000	1.82771200	3.16245200
H	0.98209300	3.63892000	1.88554900
H	0.08730900	4.91343400	0.99874000
H	0.15455700	4.93120400	2.77840500
H	-3.08726400	0.93669100	-1.25008700
H	-2.23751300	2.14599200	-2.22093200
H	-4.35529500	3.47462300	0.83621800
H	-3.49436500	4.69350000	-0.13822600
C	-4.11763400	2.90734500	-1.30471000
H	-4.63475100	3.35434600	-2.15842700
C	-6.00771500	1.53406700	-0.77743900
C	-5.72286300	0.36024600	-0.55830900
H	-6.61419200	2.40914600	-0.92409600
H	-4.22336600	-1.12332100	-2.21841900
C	-5.26995300	-0.96801600	-0.33266400
C	-5.59802800	-1.63911700	0.86113200
C	-5.16411600	-2.94169100	1.07745500
C	-4.38900400	-3.59552300	0.11814800
C	-4.05606400	-2.93760600	-1.06655000
C	-4.49570600	-1.63925600	-1.29745100
H	-6.20293400	-1.12351500	1.60861400
H	-5.43187700	-3.45170600	2.00535600
H	-4.04662400	-4.61749200	0.29307700
H	-3.43558800	-3.43070700	-1.81658000

TS II'

E_{vacuum} = -26260.9376

E_{sol} = -26260.9533

$-TS$ = -0.1510

G_{sol} = -26260.2777

Sm	-0.98490800	-0.07780600	0.06400400
O	0.53562000	-2.04128600	0.25524600
C	1.65535500	-1.97918000	1.15354100
C	2.85132800	-1.75903400	0.24599100
C	2.44169000	-2.45405000	-1.06798700
C	0.98333000	-2.85487200	-0.83934800
I	-1.72364000	-0.19560300	3.05386500
I	-0.67806800	-0.17180100	-3.03003000
O	-3.51813400	0.19898000	-0.38543400
C	-4.56827700	-0.22403100	0.50676600
C	-5.83505300	0.40960100	-0.03705200
C	-5.55790400	0.42337900	-1.53669400
C	-4.08394000	0.78070300	-1.57719200
O	-1.67767400	2.29864900	-0.16394100
C	-2.57908400	2.99465500	0.71940500
C	-2.20997300	4.46294400	0.59974800
C	-1.68464800	4.54740600	-0.83074400
C	-0.92668800	3.24090200	-0.96310100
O	-2.23463600	-2.33101300	-0.12135900
C	-2.18611900	-3.36841400	0.88432300
C	-3.00569500	-4.51987000	0.32413400
C	-2.85109400	-4.32396300	-1.18080800
C	-2.91504800	-2.81448300	-1.29182400
H	-4.61941800	-1.32581400	0.48541000
H	-4.29572200	0.08531300	1.52455400
H	-3.92570500	1.87198600	-1.54014000
H	-3.53543700	0.38373900	-2.44195400

H	-5.94454000	1.43849000	0.34273600
H	-6.73634200	-0.15295500	0.24293300
H	-6.17610800	1.13920000	-2.09577000
H	-5.72317800	-0.57776300	-1.96697000
H	-3.61032600	2.80409200	0.37699200
H	-2.46197800	2.57874500	1.73043900
H	0.09553100	3.30478800	-0.55836800
H	-0.88098400	2.83958600	-1.98475200
H	-1.41321900	4.71444300	1.31540600
H	-3.06718300	5.12227300	0.79399000
H	-1.04140800	5.41988500	-1.00967500
H	-2.52022000	4.58468800	-1.54861200
H	-1.12942800	-3.63797900	1.03113600
H	-2.56941700	-2.95021600	1.82534700
H	-3.96068300	-2.45704400	-1.27353800
H	-2.41230400	-2.39037900	-2.17190900
H	-4.06451700	-4.42009000	0.61289000
H	-2.64851500	-5.49494300	0.68292800
H	-3.62828400	-4.82408000	-1.77501600
H	-1.86817400	-4.68795000	-1.52100800
H	1.46397400	-1.17419200	1.86983200
H	1.70891500	-2.94233700	1.69352900
H	0.89028700	-3.91415300	-0.53689200
H	0.32562600	-2.65403700	-1.69419800
H	3.77176600	-2.16913700	0.67812500
H	2.99977100	-0.68337000	0.08410900
H	3.06251400	-3.33362200	-1.29023800
H	2.51857600	-1.76308200	-1.91870100
O	0.83217900	0.92698300	0.44450100
C	1.86465800	1.75824700	0.59530100
C	2.09537500	2.31224600	1.98386200
C	2.65725400	2.09701000	-0.43944000
H	2.99973000	2.94112100	1.92620600
C	0.92493300	3.18640600	2.42933800
C	2.34800700	1.19439500	2.99386700
C	3.81742300	3.06768900	-0.60242300
C	2.67247700	1.71512700	-1.90677700
H	1.09103300	3.57958500	3.44463600
H	0.79204500	4.04374600	1.75082500
H	-0.00528100	2.59729400	2.44674500
H	1.43980100	0.58668700	3.12936300
H	3.16868800	0.53764100	2.66468900
H	2.61503900	1.61254500	3.97715400
H	4.73917400	2.84345200	-0.03415900
H	3.57412900	4.13312100	-0.42465600
H	2.88237300	0.65099900	-2.12136300
H	1.76619900	1.97373800	-2.48472600
C	3.84588800	2.65758100	-2.06151500
H	4.06452600	3.30166700	-2.91817100
C	5.84365500	1.37951100	-2.26950400
C	5.98634500	0.51534900	-1.40844500
H	6.09784200	2.00473800	-3.10563600
H	6.39129300	-2.10935100	-1.68639100
C	6.06698000	-0.43847000	-0.35896600
C	5.94565600	-0.04042800	0.98592600
C	6.05824600	-0.97443700	2.00872900
C	6.29398300	-2.31871900	1.71509800
C	6.41097500	-2.72417700	0.38462400
C	6.29708600	-1.79725000	-0.64537500
H	5.75519800	1.00914700	1.21307300
H	5.95789000	-0.65033300	3.04650200
H	6.38493300	-3.04859100	2.52172000
H	6.59477800	-3.77404200	0.14718800

Int III

E_{vacuum} = -26261.0057

E_{sol} = -26261.0200

$-TS$ = -0.1491

G_{sol} = -26260.3389

Sm	-1.01002900	-0.05752800	0.07311700
O	-2.07261200	-2.19152700	-0.70272700
C	-1.85462600	-3.41829500	0.00870200
C	-0.91758400	-4.22055300	-0.88627200
C	-1.20371100	-3.67187900	-2.30082900
C	-2.24697700	-2.58186800	-2.07028000
I	-2.49522100	-0.15287100	2.77391900
I	0.10922400	0.34821000	-2.80524200
O	-1.68340700	2.43454300	-0.03883800
C	-2.77336600	3.03642500	0.68738900
C	-2.55395900	4.53325700	0.57144400
C	-1.90632100	4.64840100	-0.80444300
C	-0.99969300	3.43203300	-0.82469100
O	0.76090600	1.44566400	0.90094700
C	0.64974100	2.28313400	2.06951000
C	2.07318300	2.48595900	2.55589100
C	2.87074800	2.40589000	1.25833100
C	2.15103500	1.29937700	0.51690800
O	-3.35480100	0.30935900	-0.94336600
C	-4.55890300	-0.33914000	-0.47448100
C	-5.67125500	0.15462800	-1.38557500
C	-4.91945500	0.46019300	-2.67722500
C	-3.63529800	1.05715400	-2.13924300
H	-3.72051600	2.72374600	0.21551500
H	-2.75747300	2.64461800	1.71314400
H	-0.02453400	3.63764700	-0.35188500
H	-0.81515100	3.01082500	-1.82205800
H	-1.85965500	4.88200500	1.35313700
H	-3.48942900	5.10089100	0.67084600
H	-1.35144500	5.58439700	-0.95664400
H	-2.66984300	4.57276800	-1.59571700
H	0.18424900	3.23625200	1.76560300
H	-0.01233000	1.78279500	2.79093000
H	2.49341800	0.29738800	0.81897400
H	2.20124600	1.37638700	-0.57728100
H	2.36370300	1.66996800	3.23456800
H	2.19518300	3.43786600	3.09110800
H	3.93370500	2.17913600	1.40504700
H	2.80019000	3.35304700	0.69915300
H	-4.40333900	-1.42574200	-0.55392900
H	-4.69162300	-0.08688400	0.58650500
H	-3.77017300	2.12131600	-1.87446800
H	-2.76537700	0.96653000	-2.80431100
H	-6.12285500	1.07553300	-0.98265200
H	-6.47076800	-0.58963900	-1.50360200
H	-5.45376800	1.14555500	-3.34955500
H	-4.70436100	-0.46820900	-3.23062000
H	-1.44572000	-3.16389600	0.99115000
H	-2.83368800	-3.91424700	0.13698300
H	-3.27737900	-2.96471900	-2.19161200
H	-2.10401700	-1.69124500	-2.69449100
H	-1.10787000	-5.29946800	-0.80051200
H	0.12504500	-4.03010900	-0.59640800
H	-1.57642500	-4.44063400	-2.99204100
H	-0.29851600	-3.22927900	-2.73927200

O	0.44082800	-1.44813400	0.71321100
C	1.45992700	-2.21446700	1.10472100
C	1.54432100	-2.53651800	2.57942200
C	2.37701200	-2.65767700	0.22362900
H	2.43532200	-3.17275700	2.71341700
C	1.73203700	-1.26126000	3.39956500
C	0.31994300	-3.31175900	3.06117000
C	2.57889500	-2.40035500	-1.24671600
C	3.64283700	-3.47650000	0.34037300
H	1.80364000	-1.49180100	4.47409000
H	2.65069400	-0.73199600	3.10125900
H	0.87251400	-0.58804600	3.25729100
H	-0.58699700	-2.69273900	2.97618700
H	0.17769800	-4.23475300	2.47738900
H	0.42947500	-3.59366700	4.12011100
H	2.57500900	-1.34711200	-1.56649100
H	1.89088300	-2.94755700	-1.91310100
H	4.38439300	-3.15611000	1.08984300
H	3.48023900	-4.56190700	0.45530000
C	3.97986600	-3.06411500	-1.13385700
H	4.13283300	-3.92026200	-1.80506200
C	5.15224000	-2.12833600	-1.26170500
C	5.18444800	-0.87763700	-0.87965600
H	6.07192900	-2.55187900	-1.69955800
H	6.41231000	-0.08148200	1.35349300
C	5.53386100	0.42197500	-0.56870900
C	5.24053900	1.49968100	-1.45715300
C	5.60976500	2.79482400	-1.13760700
C	6.27568200	3.07607300	0.06234000
C	6.55854300	2.03351200	0.95442400
C	6.19042400	0.73146900	0.66050600
H	4.72359900	1.28044700	-2.39264200
H	5.38087100	3.60398100	-1.83451500
H	6.56813300	4.09953300	0.30252900
H	7.07596100	2.24625700	1.89257200

Int III’

E_{vacuum} = -26261.0030
E_{sol} = -26261.0210
$-TS$ = -0.1488
G_{sol} = -26260.3395

Sm	-0.99891700	-0.06728400	0.09752500
O	0.66177300	-1.39401800	1.40956400
C	1.43803300	-0.76573000	2.44173100
C	2.84947800	-0.67301200	1.87150600
C	2.88685800	-1.77516500	0.79266700
C	1.51257600	-2.42396000	0.88488100
I	-2.67079900	0.64286100	2.58746800
I	0.33601500	-1.11970600	-2.50861400
O	-3.25387400	-0.59642800	-1.05164700
C	-4.47493300	-0.94070500	-0.36670900
C	-5.55784500	-0.89398800	-1.42871200
C	-4.79195800	-1.32164900	-2.67625100
C	-3.46177900	-0.62009400	-2.47826800
O	-1.87272900	1.82962800	-1.25348100
C	-3.09042000	2.54878900	-0.97508700
C	-2.87474000	3.94335800	-1.53705900
C	-1.91158000	3.68028100	-2.69164000
C	-1.00777900	2.61336400	-2.10556200
O	-1.78870400	-2.48176800	0.52060800
C	-1.91851900	-3.07227300	1.83436500
C	-2.32333000	-4.51858300	1.59595300
C	-1.72641600	-4.80062500	0.22065800
C	-1.98200000	-3.48705200	-0.48900100
H	-4.36000300	-1.95190900	0.05876600
H	-4.61054900	-0.23535700	0.46381900
H	-3.48949200	0.42052200	-2.84379500
H	-2.60001800	-1.12235400	-2.93785000
H	-5.93794300	0.13390700	-1.54580700
H	-6.40906100	-1.54455800	-1.18506500
H	-5.28000400	-1.03295000	-3.61732400
H	-4.65267600	-2.41492900	-2.68621600
H	-3.91965100	2.02782900	-1.48315600
H	-3.26884900	2.52023600	0.10961200
H	-0.20141600	3.03964000	-1.48819700
H	-0.56838800	1.92658800	-2.84180000
H	-2.39988400	4.58807100	-0.78269000
H	-3.81731400	4.41461400	-1.84801100
H	-1.35180500	4.57193800	-3.00565900
H	-2.45423300	3.28951800	-3.56779400
H	-0.94138500	-2.98600400	2.33269700
H	-2.64997800	-2.48213900	2.40351900
H	-3.02146500	-3.42958500	-0.85986900
H	-1.29704000	-3.26592700	-1.31929800
H	-3.42052900	-4.61404400	1.55987100
H	-1.95239200	-5.18712100	2.38493600
H	-2.18604700	-5.65517100	-0.29480600
H	-0.64279000	-4.98527800	0.29742900
H	0.96770300	0.19568700	2.66816700
H	1.39349400	-1.41279800	3.33613300
H	1.49935000	-3.27387200	1.59279500
H	1.10188700	-2.73772300	-0.08266300
H	3.60640500	-0.82634400	2.65249100
H	3.01782900	0.31650400	1.42459700
H	3.69735400	-2.49955700	0.94808500
H	3.01779400	-1.33793400	-0.20650600
O	0.41412000	1.44304500	0.51211000
C	1.25837600	2.46909600	0.63123800
C	0.93901300	3.49704400	1.69321600
C	2.33460900	2.58400500	-0.17071300
H	1.74088100	4.25339400	1.65123900
C	-0.39224500	4.18686500	1.40057200
C	0.92926100	2.88080900	3.09067300
C	3.45572800	3.59412700	-0.28774100
C	2.86747100	1.72527000	-1.28803100
H	-0.62483500	4.93674700	2.17287400
H	-0.36539700	4.69944800	0.42600500
H	-1.20973700	3.44921700	1.39275600
H	0.11079800	2.15012200	3.18492400
H	1.88351500	2.37700300	3.31053200
H	0.76988400	3.65614700	3.85632600
H	4.13184000	3.66014400	0.58131100
H	3.16220900	4.61859600	-0.57171100
H	3.19399400	0.71483400	-0.99225800
H	2.21008600	1.60521600	-2.16307600
C	4.03030600	2.73254000	-1.46393500
H	3.99921400	3.27192800	-2.42279300
C	5.42398600	2.20837100	-1.25047700
C	5.72712300	1.02797500	-0.77903300
H	6.24347000	2.90475400	-1.49367800
H	5.96700300	-1.22650200	-2.20028400
C	6.01579400	-0.21841000	-0.27444200
C	6.21813800	-0.41559300	1.12686900
C	6.51038400	-1.67148300	1.62641000

C	6.60918300	-2.77837400	0.77219800
C	6.41245800	-2.60565500	-0.60497700
C	6.12465600	-1.35898300	-1.12920000
H	6.13777500	0.44533000	1.79194400
H	6.66553700	-1.79860800	2.70022600
H	6.84132000	-3.76550900	1.17531000
H	6.48589600	-3.46440100	-1.27571800

TS III

E_{vacuum} = -26260.9810
E_{sol} = -26260.9967
$-TS$ = -0.1469
G_{sol} = -26260.3147

C	-2.10951400	1.45852100	1.17821600
O	-1.09563900	0.73950700	0.78775300
Sm	0.76566900	-0.04239300	0.04706200
O	1.05982700	2.43586500	-0.21522300
C	0.80208900	3.37127200	0.84694500
C	1.12466600	4.62171100	-1.12391600
C	1.24096600	3.13909000	-1.46071100
I	2.16983600	0.15484000	2.82793100
I	-0.23102000	-0.35249600	-2.87435600
O	2.22422700	-2.11465900	-0.42986800
C	3.47890400	-2.40816500	0.21309700
C	3.70801800	-3.89013100	-0.01681700
C	3.06192500	-4.09473500	-1.38357800
C	1.83344000	-3.20943100	-1.28246500
O	-0.35544800	-2.15236000	0.73958600
C	0.04424700	-3.01658000	1.81871200
C	-1.22976000	-3.70959600	2.26170000
C	-1.99713100	-3.82534300	0.94847600
C	-1.68416100	-2.49996100	0.27909400
O	3.07295700	0.57256900	-0.96248600
C	4.07851200	1.36404400	-0.29896400
C	5.34250800	1.17358800	-1.11652200
C	4.77992700	0.99856800	-2.52343900
C	3.54450900	0.15810200	-2.25972100
H	4.26720100	-1.79952600	-0.26332700
H	3.39940200	-2.10847600	1.26720100
H	0.98837600	-3.73358600	-0.80577000
H	1.48559100	-2.78705900	-2.23510700
H	3.18358000	-4.48357200	0.74956700
H	4.77297600	-4.15849200	0.01540200
H	2.80565100	-5.14081900	-1.60061600
H	3.73167100	-3.73561900	-2.18194700
H	0.78479900	-3.73985100	1.43234600
H	0.52458100	-2.39829900	2.58997200
H	-2.37404400	-1.70105700	0.58620800
H	-1.65947200	-2.53516200	-0.81780000
H	-1.78067600	-3.07409900	2.97199900
H	-1.03130600	-4.67633800	2.74487900
H	-3.07711400	-3.96645200	1.07883200
H	-1.60884400	-4.66531700	0.34893700
H	3.74848400	2.41691900	-0.29926700
H	4.14476600	1.02395900	0.74349500
H	3.78659000	-0.91747700	-2.21508100
H	2.72607600	0.29535800	-2.97943700
H	5.87315700	0.26119900	-0.79892300
H	6.03531800	2.02056700	-1.01756800
H	5.47451100	0.51234200	-3.22225100
H	4.49818100	1.97560400	-2.94898400
H	-0.28800800	3.47509300	0.97810800
H	1.23556600	2.94946600	1.76398500
H	2.23792600	2.87482000	-1.84651200
H	0.48752400	2.78501400	-2.17878200
H	1.03639800	5.54386900	0.87915800
H	1.80216000	5.23764900	-1.73129200
H	0.09788500	4.97698300	-1.30196300
C	-2.54441500	1.33181600	2.62125100
H	-3.55948100	1.75701700	2.69495500
C	-1.60737200	2.15144600	3.51315700
H	-1.91518500	2.08404100	4.56872700
H	-0.57609200	1.77157100	3.43574300
H	-1.61011000	3.21477200	3.22629900
C	-2.58126300	-0.12425200	3.07349800
H	-1.57951400	-0.57056300	2.98514400
H	-2.89544000	-0.19361000	4.12646800
H	-3.28216900	-0.71606800	2.46536900
C	-2.77425100	2.29624600	0.30561600
C	-2.44096000	2.83920800	-1.07125300
C	-3.80130000	3.39908300	0.49275700
C	-4.43883600	1.07015100	-0.76771300
C	-3.89958300	3.37985200	-1.06151400
H	-1.71065700	3.66539100	-0.99638100
H	-2.14816700	2.14484800	-1.86940700
H	-4.72263800	3.20395500	1.05902000
H	-3.32485300	4.32767000	0.85698700
C	-4.76723200	2.18697700	-1.38488600
H	-4.14928400	4.30132100	-1.60354300
H	-5.67364300	-0.16297500	1.27838400
H	-5.61571300	2.27063800	-2.07641500
H	2.52918500	4.63038500	0.54176600
C	1.44094100	4.65854400	0.36964000
C	-4.70514000	-0.31366200	-0.65075000
C	-4.28665600	-1.21225400	-1.66139200
C	-4.56086000	-2.56898600	-1.55693700
C	-5.23299600	-3.07580600	-0.44071800
C	-5.62111400	-2.20498300	0.58197000
C	-5.35942900	-0.84466300	0.48583200
H	-3.74360300	-0.81883400	-2.52197400
H	-4.24515400	-3.24262200	-2.35698600
H	-5.45169400	-4.14306200	-0.36701800
H	-6.14287200	-2.59274600	1.45999000

TS III’

E_{vacuum} = -26260.9845
E_{sol} = -26261.0000
$-TS$ = -0.1470
G_{sol} = -26260.3181

C	2.07981300	1.69044700	0.61240600
O	1.08470000	0.87053100	0.39769100
Sm	-0.81911400	-0.05616000	0.01762100
O	0.67693100	-2.00542100	-0.10025900
C	1.77745200	-2.16244400	0.82447500
C	2.07617600	-3.73931400	-0.89114700
C	0.80373600	-2.95621200	-1.17912300
I	-1.45023400	-0.42452800	3.03514000
I	-0.69069900	0.15011400	-3.07536600
O	-3.36633600	0.35670200	-0.28063300
C	-4.39932600	-0.12198500	0.60290700
C	-5.64880800	0.64689900	0.21527100
C	-5.43775700	0.84954100	-1.28147500
C	-3.94838400	1.13023400	-1.34877500
O	-1.42813100	2.36081400	0.18743700

C	-2.27849400	2.97042900	1.17928600
C	-1.94141000	4.45091300	1.14575100
C	-1.51980400	4.65096400	-0.30726900
C	-0.74715800	3.37579600	-0.58042300
O	-2.15593600	-2.24704300	-0.29896900
C	-2.20245800	-3.32344700	0.66038300
C	-3.25494700	-4.28418900	0.13704400
C	-3.13530900	-4.09494000	-1.37182600
C	-2.90583900	-2.59921300	-1.47724800
H	-4.52317300	-1.20621600	0.43893600
H	-4.06312100	0.03219500	1.63714200
H	-3.72601600	2.19601300	-1.16828000
H	-3.46204600	0.83078600	-2.28691700
H	-5.67970200	1.61898500	0.73394700
H	-6.56905600	0.10076300	0.46452700
H	-6.03639700	1.66626300	-1.70780300
H	-5.68064500	-0.07374600	-1.83239000
H	-3.32684400	2.78347800	0.89206100
H	-2.08783100	2.48015900	2.14421700
H	0.29394400	3.44028200	-0.22626400
H	-0.74832200	3.05116200	-1.63020600
H	-1.09883500	4.66554200	1.82048700
H	-2.79214300	5.07668500	1.44868600
H	-0.90870200	5.54980600	-0.46741800
H	-2.40359500	4.71590100	-0.96275800
H	-1.20330800	-3.78912200	0.70399400
H	-2.42292800	-2.89091600	1.64561100
H	-3.85900800	-2.04263700	-1.46568500
H	-2.32922000	-2.27732500	-2.35522100
H	-4.25711700	-3.98219100	0.48240000
H	-3.07909800	-5.31636700	0.46980000
H	-4.02267000	-4.42190800	-1.93121400
H	-2.26498700	-4.64703200	-1.76230300
H	2.56723000	-1.44087200	0.56442100
H	1.39387300	-1.93817000	1.82850900
H	-0.09063800	-3.59853200	-1.16133400
H	0.81994600	-2.40366200	-2.12923300
H	3.25970600	-3.74792800	0.96678100
H	2.00329700	-4.78287900	-1.22771800
H	2.93416600	-3.27270400	-1.39629800
C	2.41227200	2.03545900	2.04623400
H	3.38129000	2.56216000	2.03260300
C	2.53443200	0.79068100	2.92124900
H	2.83760300	1.07048700	3.94234900
H	1.56659700	0.27231600	2.98911600
H	3.28031200	0.08883500	2.52146300
C	1.35330000	2.98398900	2.61314400
H	0.36601000	2.49639700	2.59869500
H	1.58248300	3.24664400	3.65784500
H	1.29860900	3.91816800	2.03181400
C	2.81185700	2.21819500	-0.43467900
C	3.72041200	3.43274600	-0.55078500
C	2.58389500	2.24875900	-1.93519700
C	4.67896500	0.92694600	-0.89482900
C	3.98266600	2.92460300	-1.99991400
H	3.12594000	4.36485400	-0.54908300
H	4.59075500	3.54189500	0.11117500
H	2.43477700	1.30798100	-2.47882800
H	1.77637000	2.95359800	-2.20050600
C	4.98472700	1.80904600	-1.82779700
H	4.21157700	3.64315400	-2.79736600
H	4.80445000	-1.47490100	-2.07033500
H	5.91749700	1.78510500	-2.40662300
H	1.56376500	-4.28797700	1.15488700
C	2.22996700	-3.59135200	0.61973000
C	5.11355800	-0.28234600	-0.29502700
C	5.52188000	-0.32389800	1.05815700
C	5.93119000	-1.51453900	1.64291100
C	5.95022700	-2.69991000	0.90249200
C	5.54728300	-2.67742800	-0.43534800
C	5.12127400	-1.49388600	-1.02570800
H	5.52081100	0.60285400	1.63437300
H	6.24698100	-1.52008100	2.68850700
H	6.28147200	-3.63249400	1.36324200
H	5.56832900	-3.59685000	-1.02571000

Int IV

E_{vacuum} = -26261.0163

E_{sol} = -26261.0313

$-TS$ = -0.1462

G_{sol} = -26260.3461

C	-1.67438451	1.87915675	-1.06480152
O	-0.86884588	1.11167561	-0.32921742
Sm	0.44534351	-0.12451989	0.73712291
O	2.07608532	1.45068953	-0.29655908
C	1.94302793	2.88103301	-0.17581212
C	3.52393456	2.44750611	-1.87399277
C	3.08233244	1.12066202	-1.27544504
I	0.43368541	1.64735132	3.28372969
I	0.78389271	-2.13879494	-1.57435663
O	0.71880746	-2.15900355	2.33272968
C	1.24572751	-2.11335016	3.67157141
C	0.80489571	-3.41227902	4.31995202
C	0.81542797	-4.36968267	3.13250269
C	0.26904486	-3.49269612	2.02087883
O	-1.70064073	-1.14846743	1.39143753
C	-2.28636341	-1.13803359	2.70728793
C	-3.78316209	-1.21874995	2.47594528
C	-3.86056206	-2.05281286	1.20133989
C	-2.69544606	-1.50789373	0.39945570
O	2.92112380	-0.65920463	1.35090634
C	3.79996344	0.23193259	2.06575856
C	5.00741131	-0.60785149	2.44063070
C	5.07820276	-1.59088600	1.27659665
C	3.60990273	-1.88343396	1.03199147
H	2.34602549	-2.04478560	3.61310911
H	0.86831844	-1.20196405	4.15510050
H	-0.83354142	-3.49102963	2.00403120
H	0.62870664	-3.74506778	1.01391221
H	-0.21511673	-3.31320228	4.72533664
H	1.46867651	-3.71611587	5.14111840
H	0.21020275	-5.27366261	3.28622395
H	1.84642629	-4.68559818	2.90389726
H	-1.91319775	-2.01810982	3.26037935
H	-1.95146874	-0.22801894	3.22423641
H	-2.96193714	-0.60198854	-0.16230675
H	-2.23414253	-2.22710788	-0.28993554
H	-4.19377872	-0.21281215	2.29936703
H	-4.31193129	-1.66356211	3.33033989
H	-4.80821354	-1.94507759	0.65925113
H	-3.71241089	-3.12154069	1.42875692
H	4.07423538	1.06233856	1.39317020
H	3.24385374	0.64796834	2.91691173
H	3.23875230	-2.67841591	1.70136024

H	3.35565111	-2.15722854	-0.00093246
H	4.82530355	-1.14524112	3.38558115
H	5.91489839	-0.00121392	2.56558260
H	5.65445141	-2.49991642	1.49757319
H	5.52799795	-1.10950859	0.39287502
H	1.06239698	3.20194245	-0.75380305
H	1.77125747	3.10670483	0.88556632
H	3.90657616	0.60982161	-0.75137793
H	2.64444696	0.42135661	-2.00117022
H	3.13010082	4.47170372	-1.08589737
H	4.57819983	2.43029823	-2.18299498
H	2.91537963	2.69084043	-2.75827619
C	-2.49156102	2.92542689	-0.36121867
H	-3.18417175	3.35222512	-1.10951757
C	-1.61341451	4.06391167	0.17844530
H	-2.22225437	4.83731664	0.67487344
H	-0.90198739	3.66789657	0.92011921
H	-1.04361051	4.54890941	-0.62975420
C	-3.31268654	2.32592440	0.78157987
H	-2.64054464	1.87269558	1.52664530
H	-3.90813934	3.10001158	1.29096588
H	-3.99795504	1.54638431	0.41494391
C	-1.48709936	1.83284795	-2.53296129
C	-0.06010997	1.65743006	-3.15385754
C	-1.60443245	3.07845002	-3.47769746
C	-2.25159342	0.78705760	-3.40848517
C	-0.78278152	2.15960783	-4.43004599
H	0.64893440	2.39263862	-2.74416847
H	0.36312180	0.64377696	-3.16124490
H	-2.61068311	3.37809045	-3.80634038
H	-1.03394795	3.94627915	-3.10854504
C	-1.77162053	1.01922406	-4.64074134
H	-0.24351601	2.55173481	-5.30141313
H	-4.47111472	1.38999982	-2.01187672
H	-2.04623499	0.55090995	-5.58508949
H	4.03303238	3.40080695	0.01492844
C	3.23469815	3.43276764	-0.74439760
C	-3.28244049	-0.14300416	-2.95803158
C	-3.19933500	-1.51134832	-3.24965177
C	-4.20963974	-2.38413488	-2.85145697
C	-5.31880702	-1.90548452	-2.15487625
C	-5.40603984	-0.54686896	-1.84789575
C	-4.39266251	0.32501522	-2.23684736
H	-2.31804448	-1.88476954	-3.77379639
H	-4.12738244	-3.44788645	-3.08484743
H	-6.11365389	-2.58984283	-1.85014457
H	-6.27298992	-0.16191009	-1.30586399

Int IV’

E_{vacuum} = -26261.0144
E_{sol} = -26261.0285
$-TS$ = -0.1456
G_{sol} = -26260.3425

C	2.23221700	1.38420800	0.63408500
O	1.00751800	0.90571000	0.39021400
Sm	-0.84436700	-0.04639500	0.04324700
O	0.71764400	-1.95204600	-0.06983600
C	1.83153300	-2.06477100	0.85722100
C	2.49488300	-2.92562000	-1.23973800
C	0.99729800	-2.70659800	-1.26618400
I	-1.43861000	-0.56673600	3.02755000
I	-0.88515700	0.26139200	-3.05081400
O	-3.43661800	0.22458600	-0.14937300
C	-4.41213800	-0.39234300	0.71398900
C	-5.72974700	0.28121600	0.37917600
C	-5.55709700	0.57986300	-1.10610100
C	-4.10066400	0.99893900	-1.16824800
O	-1.59036000	2.32017000	0.21384500
C	-2.37898900	2.88445900	1.28243800
C	-2.06722500	4.37134300	1.27330500
C	-1.73812300	4.62420800	-0.19534000
C	-0.97181800	3.36858000	-0.56193300
O	-2.05168500	-2.29644000	-0.36602600
C	-1.97234700	-3.41983300	0.53875900
C	-2.92963200	-4.45793600	-0.01918100
C	-2.87008700	-4.17268300	-1.51604900
C	-2.81150900	-2.65810700	-1.53467900
H	-4.44006600	-1.47272800	0.49178400
H	-4.07663000	-0.26443500	1.75148900
H	-3.97589200	2.06923300	-0.93028900
H	-3.60181300	0.79666100	-2.12543000
H	-5.83862200	1.21846300	0.94895700
H	-6.59464500	-0.35696900	0.60716900
H	-6.23257300	1.35952300	-1.48444200
H	-5.72091100	-0.33192400	-1.70340800
H	-3.44137800	2.68541500	1.06295800
H	-2.11269100	2.36863300	2.21580200
H	0.08840900	3.42923100	-0.27219400
H	-1.03953100	3.08008900	-1.61993200
H	-1.18895600	4.58248400	1.90212400
H	-2.90814700	4.97219500	1.64599600
H	-1.14668700	5.53506600	-0.36101000
H	-2.66097800	4.70079500	-0.79299600
H	-0.93019100	-3.77714700	0.54597200
H	-2.22130900	-3.06217200	1.54706100
H	-3.81957700	-2.21490200	-1.45162000
H	-2.31290500	-2.22300900	-2.41089400
H	-3.94972000	-4.28718000	0.36169100
H	-2.63485000	-5.48157500	0.24987200
H	-3.72913300	-4.56558600	-2.07724500
H	-1.95189300	-4.59634800	-1.95408500
H	2.31374900	-1.07902000	0.91985600
H	1.42073800	-2.33065400	1.84146400
H	0.44611300	-3.66333900	-1.21561600
H	0.63506000	-2.12627200	-2.12604100
H	3.80010500	-2.93439600	0.53477900
H	2.80537900	-3.78483100	-1.85017700
H	3.01497500	-2.02808300	-1.60881400
C	2.47088200	2.02750500	1.97791100
H	3.51966200	2.37788800	1.98360300
C	2.26203100	1.05641200	3.14034800
H	2.45587600	1.55094200	4.10554100
H	1.22534300	0.68885300	3.15047400
H	2.92145500	0.17875300	3.06757300
C	1.56965400	3.25296500	2.17793300
H	0.51608100	2.93658400	2.14673400
H	1.75243600	3.72773800	3.15537100
H	1.73082100	4.01115900	1.39627900
C	3.08725600	1.71789400	-0.53808800
C	3.58701700	3.18543900	-0.82809900
C	2.46966400	1.78919100	-1.96966800
C	4.40412000	0.94274700	-0.88472200
C	3.75191200	2.61292500	-2.26279600
H	2.77529500	3.92627500	-0.74992400

H	4.49229500	3.53135700	-0.30722500
H	2.32819300	0.84609000	-2.51366200
H	1.54546900	2.38371200	-1.99624600
C	4.81093000	1.55384200	-2.01445100
H	3.85291800	3.25738800	-3.14459000
H	5.70038100	-1.12386200	-2.00017400
H	5.70934100	1.37357400	-2.60382600
H	2.46605000	-4.12144000	0.57099900
C	2.75360800	-3.10600800	0.25216600
C	5.05017400	-0.16203200	-0.18234700
C	5.06143500	-0.24798000	1.21829900
C	5.72793600	-1.28429300	1.86763100
C	6.39890000	-2.26154100	1.13376600
C	6.39349900	-2.19277000	-0.26036000
C	5.72248800	-1.16105100	-0.90900900
H	4.56625300	0.52493900	1.80209400
H	5.72764900	-1.32254000	2.95914800
H	6.92050500	-3.07380400	1.64377700
H	6.90858400	-2.95596000	-0.84785100

Product

E_{vacuum} = -26261.0282
E_{sol} = -26261.0497
$-TS$ = -0.1508
G_{sol} = -26260.3677

C	-2.51491800	0.80550700	1.04055000
O	-1.41542700	0.41977700	0.66544100
Sm	0.92799600	-0.03881300	-0.00369200
O	0.71166500	2.56270900	-0.01312500
C	0.53548800	3.41623700	1.12571300
C	0.64187200	4.78872700	-0.78982000
C	0.89761500	3.34717000	-1.20341200
I	2.03159300	0.21713600	3.00946300
I	-0.18766100	-0.31060600	-2.99273000
O	2.73672000	-1.85999600	-0.50647900
C	3.99068200	-2.00205700	0.17641800
C	4.47164900	-3.40413200	-0.15484800
C	3.91262800	-3.59714600	-1.56222700
C	2.55323000	-2.93022700	-1.44559900
O	0.04750400	-2.40868000	0.59665400
C	0.58156800	-3.24952600	1.63009300
C	-0.47963300	-4.30822900	1.86892700
C	-1.05910300	-4.48422100	0.46757700
C	-1.06304000	-3.05495800	-0.04815400
O	3.13179300	1.04943000	-0.89722100
C	4.02814600	1.85402100	-0.11547700
C	5.34540600	1.83756000	-0.87131800
C	4.87054300	1.73688900	-2.31868100
C	3.69507100	0.78368000	-2.19093500
H	4.68987800	-1.23780500	-0.20802400
H	3.82311200	-1.80999400	1.24629400
H	1.79573600	-3.62505700	-1.04239800
H	2.16330100	-2.50134700	-2.38008700
H	4.02667000	-4.13685700	0.53806200
H	5.56460000	-3.49982400	-0.09425300
H	3.84027200	-4.64939100	-1.87025800
H	4.53920600	-3.06862700	-2.29929700
H	1.52413700	-3.70003100	1.26907100
H	0.81148700	-2.61540300	2.49821400
H	-1.98333200	-2.52631900	0.24162200
H	-0.93326400	-2.95811700	-1.13648900
H	-1.25091700	-3.92810600	2.55762300
H	-0.06424200	-5.23196700	2.29512100
H	-2.06271800	-4.93216000	0.45781000
H	-0.39648200	-5.11782600	-0.14463300
H	3.61607300	2.87716200	-0.04968700
H	4.06840000	1.43464900	0.90036800
H	4.02124400	-0.27054500	-2.22105300
H	2.90407600	0.91770500	-2.94279300
H	5.93496700	0.94708500	-0.59824200
H	5.95894500	2.72579900	-0.66659700
H	5.63825700	1.36767600	-3.01268900
H	4.53069700	2.72156000	-2.67938400
H	-0.53994500	3.45579600	1.38159200
H	1.07862500	2.96831400	1.97034200
H	1.93275300	3.19878200	-1.55243600
H	0.21780300	2.96694400	-1.97995700
H	0.61172700	5.59992100	1.26633100
H	1.21228500	5.50460500	-1.39781600
H	-0.42696300	5.03816700	-0.89038800
C	-3.06533500	0.38145800	2.38358100
H	-4.13786000	0.63620200	2.40806000
C	-2.32687900	1.19577500	3.45480200
H	-2.71244600	0.93844100	4.45257800
H	-1.24851100	0.97062100	3.42944600
H	-2.45949300	2.27916300	3.31121200
C	-2.88492100	-1.11545000	2.60460400
H	-1.82353900	-1.38414000	2.50173500
H	-3.21790400	-1.39104900	3.61609100
H	-3.47067400	-1.69777500	1.87778300
C	-3.32108100	1.74598100	0.21352100
C	-2.74875700	2.62725100	-0.92948800
C	-3.90242800	3.06936900	0.80882400
C	-4.59401400	1.18523600	-0.47500600
C	-4.10078700	3.37819800	-0.70043600
H	-1.86777200	3.20208100	-0.61202000
H	-2.57652000	2.14121300	-1.89874500
H	-4.79962200	2.99487600	1.43958300
H	-3.12801900	3.69396900	1.28189800
C	-5.09385900	2.28074700	-1.06961300
H	-4.27678900	4.39657700	-1.06642200
H	-6.96195600	0.11932600	0.28409000
H	-5.95526400	2.36371200	-1.73007000
H	2.14079800	4.82320400	0.78538600
C	1.04440200	4.77539500	0.68288400
C	-4.99327900	-0.22350000	-0.51949500
C	-4.09443700	-1.18640600	-1.00563800
C	-4.47477100	-2.52384900	-1.07600200
C	-5.74151400	-2.92215500	-0.64871500
C	-6.63656800	-1.97125400	-0.16161800
C	-6.26682800	-0.62955900	-0.10078200
H	-3.10804900	-0.87858900	-1.36524400
H	-3.77800000	-3.25927100	-1.48380900
H	-6.03358300	-3.97282500	-0.70261400
H	-7.63026800	-2.27537000	0.17363800

Product’

E_{vacuum} = -26261.0266
E_{sol} = -26261.0483
$-TS$ = -0.1497
G_{sol} = -26260.3652

C	2.40219500	1.43413200	0.85679300
O	1.46195500	0.71290500	0.54728800
Sm	-0.88534300	-0.08796700	0.03127100

O	0.64530400	-2.14877400	0.08148300
C	1.41176300	-2.60657400	1.20748900
C	2.37819900	-3.50944300	-0.73943500
C	1.09995800	-2.78575800	-1.12421200
I	-1.68267600	-0.67084600	3.08864300
I	-0.46072300	0.51208600	-3.11419500
O	-3.41875000	0.53020100	-0.31182600
C	-4.49637100	-0.12491000	0.38154500
C	-5.77167000	0.49465300	-0.16713600
C	-5.35795000	0.86620900	-1.58840100
C	-3.93604500	1.34804200	-1.37119400
O	-1.17589600	2.47066600	0.51209600
C	-2.13670400	2.94411600	1.47634500
C	-2.15426100	4.45518500	1.32110400
C	-1.83871400	4.62388100	-0.16252700
C	-0.80508000	3.53372000	-0.37944800
O	-2.21382000	-2.22718900	-0.66719500
C	-2.29386300	-3.40270300	0.15714000
C	-3.26228100	-4.32959600	-0.55705000
C	-3.01106800	-3.97259900	-2.01952900
C	-2.83554300	-2.46703900	-1.93867700
H	-4.43635000	-1.20387600	0.16242700
H	-4.34420900	0.01078500	1.46168500
H	-3.92383700	2.40479200	-1.04478200
H	-3.27015500	1.23885900	-2.23970700
H	-6.03673000	1.40118900	0.40098200
H	-6.62544100	-0.19553900	-0.11966500
H	-5.99723100	1.63102000	-2.05092300
H	-5.36378600	-0.02331600	-2.23921900
H	-3.11190200	2.49058700	1.23591500
H	-1.83274600	2.58512800	2.46958800
H	0.20428900	3.88764200	-0.10208000
H	-0.76965800	3.12625300	-1.40099800
H	-1.36249300	4.91583200	1.93401000
H	-3.11544700	4.89446500	1.62189000
H	-1.45886800	5.62124100	-0.42412200
H	-2.73485300	4.42916900	-0.77327000
H	-1.28538100	-3.84386000	0.22958100
H	-2.60584800	-3.09265700	1.16498600
H	-3.81228300	-1.94997300	-1.95828700
H	-2.19707500	-2.02909800	-2.71914900
H	-4.30201200	-4.09130700	-0.27881400
H	-3.08171800	-5.38747100	-0.32085300
H	-3.82636400	-4.26641800	-2.69515100
H	-2.08280200	-4.44766600	-2.37703100
H	2.13885900	-1.82365800	1.47920100
H	0.71861700	-2.75248000	2.04797900
H	0.31575100	-3.48818400	-1.46000100
H	1.22756900	-2.01571600	-1.89937100
H	3.02945500	-4.07342600	1.28954600
H	2.57487800	-4.38253500	-1.37719200
H	3.23965500	-2.82884000	-0.81031100
C	2.57627500	1.90944300	2.28413900
H	3.63300800	2.20042900	2.41768800
C	2.17410400	0.84843400	3.30082900
H	2.29560000	1.24272600	4.32044600
H	1.12193500	0.55156300	3.17216300
H	2.78795000	-0.06047700	3.21577300
C	1.70308600	3.16239800	2.45625800
H	0.65123800	2.90377300	2.26666000
H	1.79219100	3.53744900	3.48639400
H	1.99300800	3.97389800	1.77328900
C	3.33315100	1.92351500	-0.21099800
C	4.07120600	3.29902700	-0.17083100
C	2.74857300	2.39481600	-1.58032000
C	4.48650500	1.01187700	-0.72808800
C	4.16272100	3.03010600	-1.70175100
H	3.40506700	4.14023300	0.07441600
H	5.01271700	3.35813900	0.39401200
H	2.44911500	1.62189100	-2.29864200
H	1.94510700	3.13598600	-1.45598000
C	5.01431700	1.77331800	-1.70599500
H	4.39033100	3.82659500	-2.41924100
H	5.57739300	-0.92782000	-2.22365400
H	5.87176900	1.56389800	-2.34310700
H	1.44480400	-4.73386600	0.79962000
C	2.11350900	-3.86055900	0.72164400
C	4.89955500	-0.31007200	-0.26995100
C	4.76754400	-0.70880300	1.06743700
C	5.22796800	-1.95122300	1.49506800
C	5.83160100	-2.82501200	0.59310300
C	5.96492600	-2.44501800	-0.74326000
C	5.49820500	-1.20752600	-1.17164400
H	4.32800500	-0.02572900	1.79382100
H	5.11908800	-2.23371300	2.54430900
H	6.19183100	-3.79972900	0.92722300
H	6.42419500	-3.12663000	-1.46203000

Isopropyl Bicyclo[2.1.0]Pentyl Ketone Reactant

E_{vacuum} = -25992.0073
E_{sol} = -25992.0277
$-TS$ = -0.1396
G_{sol} = -25991.4283

C	-3.19719200	0.46633000	-0.54317100
O	-1.99402100	0.22424600	-0.44666900
Sm	0.46726600	0.00273300	-0.09836500
O	-0.27808200	-2.13445000	-1.38840800
C	-0.98257400	-2.11544900	-2.62780400
C	-2.28014700	-2.83915700	-2.31840100
C	-1.82668200	-3.93783800	-1.34525900
C	-0.49164700	-3.41513100	-0.78723900
I	1.29975700	1.30252100	-2.89683000
I	-0.21639600	-1.34399000	2.77644500
O	2.55800200	1.12565100	0.97211700
C	3.79863800	1.33535800	0.27639700
C	4.71547200	2.01346000	1.28094200
C	4.21651600	1.43382800	2.60189600
C	2.71681800	1.40030400	2.37142400
O	-0.18796500	2.31015900	0.87892600
C	0.41885200	3.54113300	0.44857900
C	-0.38070900	4.64333800	1.12404300
C	-0.83392700	3.96050500	2.41205700
C	-1.14291300	2.55907500	1.91773700
O	2.49583400	-1.62493500	-0.27709200
C	2.96159100	-2.16706500	-1.52706000
C	4.06716600	-3.14791300	-1.16344800
C	3.66133200	-3.58413800	0.24194400
C	3.14463800	-2.28106300	0.81967000
H	4.18813700	0.35177000	-0.03593800
H	3.58932500	1.92252200	-0.62910200
H	2.25542300	2.37970200	2.59654200
H	2.17956300	0.62355400	2.93498200

H	4.56706900	3.10552200	1.26358300
H	5.77723600	1.81262300	1.08148400
H	4.49556800	2.03009400	3.48170500
H	4.60301800	0.41151800	2.74477500
H	1.47202100	3.53746700	0.77647500
H	0.40257800	3.56809200	-0.65071700
H	-2.16104200	2.50178900	1.49020700
H	-1.03460600	1.76663900	2.67271800
H	-1.25408600	4.91800200	0.51029100
H	0.21527500	5.55134800	1.29025900
H	-1.69955200	4.44204700	2.88782000
H	-0.01087700	3.93382200	3.14467800
H	2.10808300	-2.66337400	-2.01586600
H	3.28368500	-1.33462600	-2.16935800
H	3.97776900	-1.64772800	1.17911500
H	2.40939300	-2.38998200	1.63003000
H	5.04369700	-2.63796700	-1.13411800
H	4.14144100	-3.97694500	-1.88084800
H	4.48639100	-4.01179300	0.82834200
H	2.84807200	-4.32687600	0.19868800
H	-1.08349700	-1.06586000	-2.93466900
H	-0.39017300	-2.64654500	-3.39702300
H	0.34647400	-4.08322800	-1.05588200
H	-0.48674200	-3.26527800	0.30255400
H	-2.76981500	-3.23660700	-3.21841800
H	-2.97794900	-2.14842300	-1.82301500
H	-1.67881600	-4.89391800	-1.86826000
H	-2.56259500	-4.10989300	-0.54784700
C	-3.69638900	1.52223800	-1.50415500
H	-4.51218700	2.06601800	-0.99619200
C	-2.59993900	2.49575300	-1.90243300
H	-3.00167100	3.26493700	-2.57851600
H	-2.17596400	2.99656200	-1.02029400
H	-1.76813800	1.98609900	-2.41177300
C	-4.29901800	0.80348500	-2.71833600
H	-3.51824300	0.24184900	-3.25470900
H	-5.09521600	0.09938800	-2.43190400
H	-4.72742900	1.53663100	-3.41770800
C	-4.17174900	-0.27922600	0.25518500
C	-4.07009400	-0.46424600	1.80791600
H	-3.26770500	-0.06780400	2.43339100
C	-3.79830400	-1.59493400	0.90310500
H	-2.74123800	-1.85475900	0.82487300
H	-4.51603000	-2.41710100	0.81371300
C	-5.67629900	-0.04524700	0.39487700
H	-6.30998900	-0.77930000	-0.12720900
H	-5.99533200	0.96222400	0.09806600
C	-5.56316400	-0.19677000	1.93540300
H	-6.14431800	-1.00941100	2.39839300
H	-5.78748700	0.74045500	2.46150500

Int I

E_{vacuum} = -25991.9915
E_{sol} = -25992.0046
$-TS$ = -0.1341
G_{sol} = -25991.4007

C	-3.06233600	0.35984500	-0.65563500
O	-1.75943600	0.19622800	-0.39695900
Sm	0.31555000	-0.00705700	-0.09615400
O	-0.12307600	-1.93667200	-1.61284100
C	-1.14028700	-1.86339900	-2.62415800
C	-2.23932000	-2.81151800	-2.15150600
C	-1.51768400	-3.75869500	-1.17004900
C	-0.06100400	-3.31950700	-1.24272300
I	1.05913900	1.86499600	-2.41901700
I	0.12977000	-1.92959500	2.34588400
O	2.43003200	0.94223700	1.08082200
C	3.55354300	1.55297000	0.41507800
C	4.38202900	2.18399800	1.51900000
C	4.12435700	1.23645200	2.68569400
C	2.65275300	0.91688000	2.50498400
O	-0.19131300	1.86502100	1.44327800
C	0.26984000	3.22468000	1.30523900
C	-0.78576500	4.08020700	1.98368200
C	-1.32788500	3.13576400	3.05336300
C	-1.33909600	1.80944800	2.31945300
O	2.46959100	-1.28208200	-0.72334200
C	2.91729800	-1.49764300	-2.08181000
C	4.19690200	-2.31097200	-1.96848300
C	3.98944900	-3.06033400	-0.65634700
C	3.32044000	-1.99706400	0.18976900
H	4.11137000	0.76402500	-0.11734500
H	3.16594500	2.25823100	-0.33183100
H	2.00785800	1.68013000	2.97309600
H	2.34367100	-0.06845500	2.87829200
H	4.00522400	3.19269700	1.75412800
H	5.44228100	2.27371200	1.24514800
H	4.33835800	1.67564900	3.66979700
H	4.73208400	0.32293600	2.58112000
H	1.24786600	3.30675000	1.80856700
H	0.40449900	3.43574300	0.23465000
H	-2.23827600	1.68272700	1.69836800
H	-1.21893600	0.92601300	2.96172500
H	-1.57954300	4.34327000	1.26889600
H	-0.36548200	5.01055200	2.38983300
H	-2.32555100	3.41563300	3.41852900
H	-0.64517200	3.09434700	3.91756500
H	2.12061800	-2.04184100	-2.61108900
H	3.04290400	-0.51684500	-2.56066400
H	4.06356400	-1.29693300	0.61195900
H	2.69183900	-2.37940200	1.00551300
H	5.07309400	-1.64715400	-1.89162500
H	4.34702900	-2.96871100	-2.83559100
H	4.92024400	-3.43292900	-0.20689800
H	3.30903800	-3.91523000	-0.79987600
H	-1.44800400	-0.81659700	-2.70905300
H	-0.68976800	-2.19036000	-3.57814300
H	0.49685600	-3.86401300	-2.02757600
H	0.47521000	-3.38912400	-0.28813600
H	-2.69252200	-3.34431300	-2.99909300
H	-3.02838800	-2.24651700	-1.63773000
H	-1.63212600	-4.82004700	-1.43062300
H	-1.89266300	-3.61572600	-0.14706000
C	-3.46129000	1.54438700	-1.49802800
H	-4.56231200	1.58996100	-1.48836100
C	-2.93753100	2.85895900	-0.91253300
H	-3.23987400	3.71792700	-1.53296700
H	-3.32791400	3.02095300	0.10477600
H	-1.83759300	2.84531700	-0.87292300
C	-3.01480200	1.39744300	-2.95474400
H	-1.91612300	1.36427100	-3.02266400
H	-3.42486800	0.47821300	-3.40160600
H	-3.35560600	2.25256100	-3.56068900
C	-4.03825500	-0.41455400	0.09904400

C	-4.47536100	-0.38429400	1.63221100
H	-4.09456100	0.24394100	2.44046000
C	-3.66540900	-1.49914400	1.06251700
H	-2.61431200	-1.50681300	1.35688400
H	-4.11737100	-2.49529900	0.95852500
C	-5.51555300	-0.58972600	-0.26846500
H	-5.74338600	-1.52339700	-0.81095300
H	-5.92854700	0.24997300	-0.84255700
C	-5.92850000	-0.53125500	1.22462000
H	-6.44883800	-1.41814000	1.62563100
H	-6.53370500	0.35818900	1.45047900

TS I

E_{vacuum} = -25991.9899
E_{sol} = -25992.0030
$-TS$ = -0.1336
G_{sol} = -25991.4001

C	-3.06077000	0.34213800	-0.68724300
O	-1.75353400	0.16451300	-0.45544400
Sm	0.31685400	-0.00671300	-0.10492900
O	-0.04480600	-1.93314300	-1.64861200
C	-1.03712200	-1.86676700	-2.68461000
C	-2.13998900	-2.82199500	-2.23730700
C	-1.42848900	-3.77671400	-1.25521800
C	0.02425000	-3.31803400	-1.28632900
I	1.08376300	1.90002000	-2.39021900
I	0.10424000	-1.96237900	2.30858700
O	2.37744100	0.96866200	1.14041100
C	3.50394500	1.61070400	0.51017700
C	4.28721400	2.25065400	1.64143000
C	4.02368600	1.28417200	2.79120700
C	2.56579700	0.92908000	2.56934300
O	-0.27359000	1.83362300	1.43955500
C	0.16777000	3.20285100	1.34069300
C	-0.91661600	4.02685400	2.01283900
C	-1.46801500	3.05121600	3.04923800
C	-1.43976000	1.73990400	2.28923700
O	2.51140400	-1.23312000	-0.68451700
C	2.99651200	-1.43232500	-2.03263700
C	4.28912900	-2.22022100	-1.89171400
C	4.06340700	-2.98222500	-0.58990900
C	3.35266400	-1.93778700	0.24552900
H	4.09428500	0.83960900	-0.01331400
H	3.11971100	2.31293700	-0.24125500
H	1.88947300	1.66855900	3.03109600
H	2.27372900	-0.06898000	2.92186100
H	3.87889900	3.24698800	1.87670500
H	5.35158500	2.36987900	1.39636000
H	4.20090900	1.71780800	3.78503100
H	4.65730800	0.38769200	2.69311800
H	1.13290000	3.29042000	1.86740100
H	0.32289100	3.43935600	0.27821700
H	-2.32428400	1.60604900	1.64870400
H	-1.31540200	0.84726200	2.91792400
H	-1.69768100	4.29361500	1.28567800
H	-0.52041800	4.95436900	2.44864400
H	-2.47869400	3.30703200	3.39572800
H	-0.80550500	3.00353600	3.92872500
H	2.22444900	-1.98997200	-2.58393600
H	3.11353800	-0.44655000	-2.50335200
H	4.07125000	-1.22630400	0.69056200
H	2.71107100	-2.33752700	1.04261100
H	5.14942000	-1.53940800	-1.78837400
H	4.47458700	-2.86904000	-2.75870600
H	4.98968000	-3.33965900	-0.11930900
H	3.40366900	-3.84918800	-0.75588500
H	-1.34861700	-0.82203200	-2.77825800
H	-0.56136800	-2.19187600	-3.62701100
H	0.60938000	-3.84939700	-2.06013100
H	0.53604900	-3.38794600	-0.31850800
H	-2.58025200	-3.34760300	-3.09611100
H	-2.93787000	-2.26355200	-1.72958900
H	-1.52213200	-4.83451000	-1.53765200
H	-1.82977200	-3.65832900	-0.23913300
C	-3.44421400	1.51466400	-1.55571400
H	-4.54401600	1.56489400	-1.56617600
C	-2.92267500	2.83488800	-0.98401300
H	-3.21095900	3.68370100	-1.62453000
H	-3.33024900	3.01545100	0.02321000
H	-1.82371100	2.81839400	-0.92621200
C	-2.97483600	1.33792800	-3.00123400
H	-1.87523600	1.31549000	-3.05438800
H	-3.37096400	0.40575900	-3.43362800
H	-3.31585400	2.17649200	-3.62936300
C	-4.01198900	-0.45233400	-0.00119900
C	-4.45239500	-0.54594400	1.71309400
H	-4.04401900	0.00099000	2.56435900
C	-3.66419400	-1.54520800	0.95392700
H	-2.60565400	-1.58971100	1.22086300
H	-4.10548300	-2.54199400	0.78140100
C	-5.51680200	-0.48792600	-0.24407500
H	-5.83732700	-1.35835600	-0.84434500
H	-5.90498700	0.41632900	-0.72922100
C	-5.88767500	-0.53720100	1.25509300
H	-6.47895800	-1.41942800	1.56560500
H	-6.43276500	0.36347800	1.57191400

Int II

E_{vacuum} = -25992.0224
E_{sol} = -25992.0388
$-TS$ = -0.1349
G_{sol} = -25991.4362

C	-3.01795600	0.47778200	-0.77104100
O	-1.71653500	0.21023700	-0.60846000
Sm	0.32948600	0.01021000	-0.11673300
O	0.13183600	-1.74957000	-1.89361200
C	-0.80790300	-1.59641500	-2.96739100
C	-1.92431600	-2.58282800	-2.64685400
C	-1.22684700	-3.65692900	-1.78455100
C	0.21311000	-3.16384700	-1.67471300
I	1.21641500	2.18896600	-2.10434500
I	0.00672200	-2.24167700	2.02039000
O	2.25262600	0.86142700	1.39573300
C	3.40435500	1.59845900	0.93597400
C	4.09311100	2.09630400	2.19319700
C	3.77217100	0.98608100	3.18767800
C	2.34135100	0.64422600	2.81874700
O	-0.43559100	1.63243600	1.59858700
C	-0.01710200	3.00840500	1.69432500
C	-1.16065800	3.73190500	2.38357500
C	-1.75442700	2.63309200	3.26078500
C	-1.64850200	1.42002500	2.35737400
O	2.60147100	-1.07848500	-0.68424300
C	3.17698400	-1.10851100	-2.01128600

C	4.43369600	-1.95994500	-1.89905900
C	4.12264300	-2.85184400	-0.70117600
C	3.39136900	-1.88445700	0.20568000
H	4.04652100	0.91172500	0.35942900
H	3.05791300	2.38904200	0.25749300
H	1.61904800	1.31157300	3.31933400
H	2.04785500	-0.39404200	3.02269900
H	3.64780600	3.04844900	2.52472200
H	5.16892100	2.26011500	2.04153000
H	3.87079600	1.28957000	4.23901500
H	4.42993700	0.11766700	3.02057000
H	0.90836700	3.04523400	2.29338500
H	0.20466000	3.37331200	0.68106700
H	-2.49225900	1.34572000	1.65355800
H	-1.53945700	0.46421600	2.88790700
H	-1.89901300	4.07027000	1.64168000
H	-0.81464200	4.60814200	2.94900400
H	-2.79039000	2.83085900	3.56829000
H	-1.14752900	2.49300200	4.17002600
H	2.42569500	-1.54939500	-2.68298200
H	3.36000500	-0.07344500	-2.33156100
H	4.10097100	-1.23471800	0.74980800
H	2.70914500	-2.35098200	0.92941300
H	5.31123900	-1.32966600	-1.68320900
H	4.63915100	-2.51699100	-2.82348000
H	5.01538400	-3.28299300	-0.22748900
H	3.45300000	-3.67722300	-0.99190800
H	-1.11648500	-0.54742200	-2.99168200
H	-0.28882300	-1.84789500	-3.90987500
H	0.85961200	-3.59756300	-2.46009900
H	0.66404600	-3.32555100	-0.68803300
H	-2.37048400	-2.99438400	-3.56282100
H	-2.71551200	-2.07682000	-2.07645400
H	-1.27249600	-4.66072100	-2.22933000
H	-1.67723000	-3.70700300	-0.78323700
C	-3.33917100	1.68576600	-1.62635100
H	-4.43538700	1.75188500	-1.69653900
C	-2.82468200	2.96927800	-0.97693900
H	-3.05787000	3.84568200	-1.60183400
H	-3.28791400	3.12271700	0.01056400
H	-1.73140000	2.92900900	-0.85658500
C	-2.78982000	1.54015400	-3.04384400
H	-1.68904400	1.53073300	-3.03612200
H	-3.15369300	0.61520300	-3.51825900
H	-3.10614200	2.38896600	-3.67011000
C	-3.96575500	-0.28785100	-0.18153100
C	-4.91460700	-1.64765000	1.50227700
H	-5.01803200	-2.36173400	2.32069600
C	-3.66265600	-1.46359800	0.71469800
H	-2.76546000	-1.29888400	1.33917600
H	-3.41063200	-2.37720400	0.13227700
C	-5.46243800	-0.09019200	-0.21765700
H	-5.91039300	-0.69927600	-1.02573700
H	-5.77299900	0.94939100	-0.39184700
C	-5.94313000	-0.63013200	1.14076900
H	-6.96979900	-1.03311100	1.10718700
H	-5.96470600	0.19312400	1.88728700

PreTS II

E_{vacuum} = -26300.2859

E_{sol} = -26300.3004

$-TS$ = -0.1553

G_{sol} = -26299.5972

C	0.20139600	3.13284700	0.82170200
O	0.57211600	1.90553600	0.43977000
Sm	0.93486000	-0.13897500	0.04133400
O	2.71869700	0.89653100	-1.38673800
C	3.33851500	2.12776500	-0.98944600
C	2.75801400	3.17224300	-1.93442000
C	2.37996100	2.35758800	-3.19033200
C	2.73716000	0.92035300	-2.81952500
I	2.86080400	-0.51466800	2.41570900
I	-0.86168800	-0.30843100	-2.50870200
O	0.15029300	-2.57102700	0.45246100
C	0.89160800	-3.57653900	1.16838800
C	-0.13963900	-4.60799700	1.58353200
C	-1.11217100	-4.56322900	0.40912900
C	-1.14588700	-3.08115700	0.07837600
O	-1.03222900	-0.26045200	1.52509200
C	-1.12212500	-0.87533700	2.82519900
C	-2.30933500	-0.21335200	3.50212400
C	-3.20559800	0.12525800	2.31468100
C	-2.19390900	0.56837400	1.27798600
O	2.42874900	-1.90772400	-1.12048000
C	3.86582700	-1.97977300	-0.97689100
C	4.31783700	-3.09916800	-1.90263200
C	3.22236400	-3.10746600	-2.96400100
C	1.99200700	-2.84233700	-2.12163200
H	1.64708900	-4.00919600	0.48966000
H	1.41576900	-3.08299900	1.99787500
H	-1.91027100	-2.54237300	0.66103400
H	-1.30645500	-2.85509400	-0.98462700
H	-0.64189800	-4.29669300	2.51373500
H	0.30433900	-5.59869100	1.75271400
H	-2.11033500	-4.95692100	0.64639100
H	-0.71119600	-5.13963400	-0.44020100
H	-1.28838900	-1.95628200	2.68232000
H	-0.16232200	-0.73138700	3.34134500
H	-1.90245300	1.62331900	1.39696600
H	-2.50387200	0.40222900	0.23918000
H	-1.98995100	0.70398800	4.01913300
H	-2.78595700	-0.87538500	4.23826100
H	-3.94582900	0.90819600	2.52693900
H	-3.74728700	-0.76870700	1.96716100
H	4.27595600	-1.00127900	-1.26881900
H	4.09747600	-2.14713100	0.08391200
H	1.64112100	-3.76584300	-1.62634000
H	1.14901400	-2.38729800	-2.65916400
H	4.33383300	-4.06233200	-1.36768000
H	5.32417400	-2.92124000	-2.30607300
H	3.15570600	-4.05040300	-3.52411000
H	3.37206400	-2.28751100	-3.68489800
H	3.11023400	2.28830000	0.06804800
H	4.43042300	2.01410500	-1.11510700
H	3.75490200	0.64834000	-3.15566900
H	2.01505100	0.17648700	-3.17759500
H	3.48315100	3.97038700	-2.14537400
H	1.86693400	3.62834100	-1.48106500
H	2.91962400	2.68289300	-4.09059900
H	1.30254100	2.43018800	-3.39498200
C	0.71997200	3.59103800	2.16935300
H	0.35605700	4.61805800	2.32437500
C	0.17326000	2.71639100	3.29625600
H	0.55648200	3.05186400	4.27274900

H	-0.92675600	2.75778800	3.32989300
H	0.48607300	1.67055000	3.15748400
C	2.24634500	3.62733500	2.20933900
H	2.66274300	2.61144800	2.12843100
H	2.65195500	4.24765300	1.39466500
H	2.59765000	4.04968000	3.16378200
C	-0.59739100	3.89610900	0.03993100
C	-2.17715500	4.47144100	-1.62635000
H	-2.72684900	4.49872400	-2.56926200
C	-1.14691800	3.44819500	-1.29320700
H	-1.55527200	2.41919100	-1.25005200
H	-0.36381100	3.38160200	-2.07666000
C	-1.10296200	5.28965200	0.33860800
H	-0.37216800	6.04656000	-0.00386200
H	-1.27859900	5.47922100	1.40716300
C	-2.38856100	5.42140500	-0.49640100
H	-2.59071200	6.45510900	-0.82244800
H	-3.26482400	5.11949900	0.11795800
C	-4.61862700	2.08588300	-1.02276900
C	-4.94470700	0.94653700	-0.75841300
H	-7.39547300	-2.24840500	1.49017400
H	-6.92319700	0.10737300	0.84671200
C	-5.25863700	-0.40618900	-0.42621300
C	-4.47361700	-1.45072500	-0.94271300
C	-4.74172900	-2.76644600	-0.57842000
C	-5.78681100	-3.05864400	0.29826500
C	-6.57354400	-2.02499400	0.80726000
C	-6.31558500	-0.70629600	0.44809300
H	-3.64748400	-1.21371200	-1.61658800
H	-4.12964900	-3.57240100	-0.98928900
H	-5.99332900	-4.09290700	0.58088200
H	-4.28760300	3.07794400	-1.27249900

PreTS II’

E_{vacuum} = -26300.2800

E_{sol} = -26300.2953

$-TS$ = -0.1561

G_{sol} = -26299.5927

C	1.89848200	1.51266700	0.88311100
O	0.85044500	0.74977200	0.55014700
Sm	-1.01602800	-0.11573400	0.05793800
O	0.46500500	-2.10137800	-0.26582600
C	1.67560700	-2.24529900	0.49462600
C	2.78711700	-1.93567600	-0.49571600
C	2.17575200	-2.30891700	-1.86188400
C	0.74650700	-2.72726900	-1.52575100
I	-1.67744800	-0.81306200	2.98846000
I	-0.89742400	0.37203100	-3.02308700
O	-3.56448200	0.30348700	-0.21431700
C	-4.59085400	-0.25243700	0.63179200
C	-5.85210300	0.51864000	0.29167100
C	-5.64563200	0.81454700	-1.18988500
C	-4.16040600	1.12045900	-1.24259200
O	-1.64002200	2.27467900	0.32936100
C	-2.49465000	2.81192100	1.35799100
C	-2.07479000	4.26276900	1.51571900
C	-1.58607200	4.61054100	0.11232500
C	-0.87993200	3.33320200	-0.29849200
O	-2.34635200	-2.26560900	-0.49151200
C	-2.26704800	-3.48678600	0.27868500
C	-3.14589900	-4.49030200	-0.45107300
C	-3.07894200	-3.99223000	-1.89126900
C	-3.11141200	-2.49153700	-1.68800200
H	-4.69195300	-1.32577600	0.39742300
H	-4.25877400	-0.16184700	1.67429600
H	-3.95094500	2.17844300	-1.01020900
H	-3.67256500	0.87681100	-2.19572400
H	-5.89717500	1.45677500	0.86852200
H	-6.76336900	-0.05615300	0.50720600
H	-6.25621600	1.64696400	-1.56599200
H	-5.87652400	-0.07721300	-1.79504000
H	-3.53994700	2.72529000	1.01653500
H	-2.36933600	2.20240800	2.26445300
H	0.15544300	3.28756200	0.07419300
H	-0.88084600	3.13923900	-1.37987700
H	-1.24994800	4.34366200	2.23902700
H	-2.90154800	4.89705500	1.86417700
H	-0.91593100	5.48069200	0.08552000
H	-2.43896700	4.81386800	-0.55548600
H	-1.21083200	-3.79485100	0.30320900
H	-2.58617900	-3.26445300	1.30614400
H	-4.14438100	-2.13328900	-1.52781200
H	-2.65466100	-1.90269500	-2.49529100
H	-4.18243100	-4.44030100	-0.08040300
H	-2.79076100	-5.52197500	-0.32212000
H	-3.90379600	-4.35014400	-2.52275800
H	-2.12864500	-4.29116900	-2.36231200
H	1.61559900	-1.56101900	1.34588400
H	1.72151100	-3.28627200	0.86266500
H	0.65723400	-3.82135600	-1.39249700
H	-0.00141400	-2.37799800	-2.24798700
H	3.70328200	-2.49486200	-0.26480800
H	3.02659600	-0.86496600	-0.46002200
H	2.71956100	-3.12028700	-2.36554500
H	2.16252000	-1.43949300	-2.53392100
C	2.12485300	1.70826700	2.36846700
H	3.06950400	2.26215700	2.48262700
C	1.00521300	2.54302800	2.98824100
H	1.16945500	2.68190900	4.06847800
H	0.95212000	3.53892600	2.52062300
H	0.03580300	2.03720000	2.86277600
C	2.27069200	0.37608100	3.10011800
H	1.32556200	-0.18726200	3.07680000
H	3.05996000	-0.24272500	2.64689300
H	2.53010700	0.54198900	4.15759500
C	2.67606500	2.08611100	-0.06541300
C	3.16039900	3.09415900	-2.14676900
H	3.09724900	3.36613600	-3.20228100
C	2.44339500	1.93320500	-1.54818600
H	1.36968300	1.89856900	-1.80783100
H	2.84373500	0.96913500	-1.92911900
C	3.81865800	3.05140500	0.14742700
H	4.78544900	2.51557000	0.16262600
H	3.74844000	3.62562300	1.08195100
C	3.78326900	3.95366600	-1.09893000
H	4.77952700	4.33879600	-1.37742600
H	3.15713700	4.85059400	-0.90184600
C	5.83960900	1.06420000	-2.22979700
C	5.94440800	0.23169000	-1.35249000
H	7.03126200	-3.97597700	0.17530400
H	6.92161900	-2.24172000	-1.60527500
C	6.04386200	-0.74902300	-0.31999900
C	5.58536200	-0.46283400	0.97592200
C	5.64983800	-1.43355200	1.96941100

C	6.16556400	-2.69846600	1.68647700
C	6.62486700	-2.98829700	0.40171400
C	6.56821400	-2.02150600	-0.59703000
H	5.15883700	0.51874500	1.18533000
H	5.28782200	-1.20199200	2.97283100
H	6.21075300	-3.45843200	2.46889000
H	5.71659500	1.80809500	-2.99267700

TS II

E_{vacuum} = -26300.2767
E_{sol} = -26300.2912
$–TS$ = -0.1516
G_{sol} = -26299.5851

C	1.49618500	-2.28826500	1.13244600
O	0.50744300	-1.50704500	0.68180700
Sm	-0.94255400	-0.11638100	0.03633900
O	-1.51521000	-2.06591900	-1.43725300
C	-1.32695600	-3.41350800	-0.98419400
C	-0.17258500	-3.94317500	-1.82543500
C	-0.24935300	-3.10448800	-3.11934800
C	-1.40089900	-2.13389200	-2.86368300
I	-2.93981600	-0.97722900	2.22790200
I	0.61179300	1.07647500	-2.38998200
O	-1.88632200	2.27726000	0.30790400
C	-3.16221000	2.60315000	0.89236100
C	-3.09353100	4.08734900	1.19692000
C	-2.21271900	4.59641600	0.06066200
C	-1.18913700	3.48287700	-0.06786700
O	0.39180700	1.27382900	1.59591200
C	-0.12013100	1.82095800	2.82634300
C	1.05265500	1.81114800	3.78688300
C	2.22994000	2.04982300	2.84728700
C	1.84224600	1.24421600	1.62063800
O	-3.08325300	0.29639400	-1.36249400
C	-4.25065800	-0.55600700	-1.33438100
C	-5.19339400	-0.00198600	-2.39263400
C	-4.23411000	0.68495900	-3.35954400
C	-3.22365200	1.27984100	-2.40118900
H	-3.95280700	2.37328100	0.15766700
H	-3.31073000	1.95548700	1.76663100
H	-0.33883700	3.61831000	0.62130600
H	-0.78776700	3.34154900	-1.08025900
H	-2.60456900	4.25812300	2.16970400
H	-4.08799200	4.55334400	1.22996700
H	-1.74582200	5.57010200	0.26349200
H	-2.80023900	4.68766900	-0.86731700
H	-0.47245200	2.84903300	2.63194900
H	-0.97272300	1.20440500	3.14573300
H	2.15948800	0.19306000	1.68752400
H	2.20492800	1.66942200	0.67542800
H	1.13657100	0.82879000	4.27355500
H	0.95293300	2.57689700	4.56868100
H	3.19565900	1.72710500	3.25825900
H	2.31329300	3.11809400	2.59298900
H	-3.91659100	-1.57935800	-1.56237300
H	-4.65835500	-0.54725000	-0.31425300
H	-3.59893300	2.22150300	-1.96056900
H	-2.22752700	1.45937900	-2.82812000
H	-5.87991200	0.73817900	-1.95119100
H	-5.80184100	-0.78981200	-2.85767600
H	-4.71316400	1.44383500	-3.99352000
H	-3.74486100	-0.05362300	-4.01485900
H	-1.13164100	-3.37786400	0.09110600
H	-2.26447400	-3.96886700	-1.16835800
H	-2.35629000	-2.51588300	-3.26902400
H	-1.21798700	-1.11779100	-3.23415600
H	-0.27156700	-5.02192000	-2.00958300
H	0.77805100	-3.77159500	-1.30120700
H	-0.43630400	-3.71522100	-4.01358400
H	0.68312400	-2.54591100	-3.28132500
C	1.24834500	-2.97212400	2.45989100
H	2.15010400	-3.55788900	2.69578000
C	1.05213000	-1.94266500	3.57113200
H	0.87815800	-2.43884600	4.53888500
H	1.94010700	-1.29927400	3.67314000
H	0.17641600	-1.31102400	3.35623300
C	0.05915700	-3.92897500	2.40037000
H	-0.87718700	-3.37704200	2.22440600
H	0.18916000	-4.67766700	1.60309100
H	-0.05267000	-4.46690200	3.35485500
C	2.64101800	-2.41883500	0.42447800
C	4.04988800	-2.45576500	-1.48036500
H	4.27892000	-2.41068700	-2.54785300
C	2.87587900	-1.73811900	-0.90093400
H	3.07257500	-0.65367800	-0.78883400
H	1.98321900	-1.77567400	-1.55170200
C	3.83477700	-3.28564400	0.74050700
H	3.59976400	-4.17000600	1.34902500
H	4.60120700	-2.70349000	1.28570500
C	4.38545400	-3.65035700	-0.64586300
H	3.85729000	-4.54585300	-1.03597100
H	5.45866500	-3.90755600	-0.63897100
C	5.92825800	-1.02644400	-1.04217900
C	5.61057700	0.11600500	-0.72274300
H	5.20767700	3.74431900	2.08677400
H	6.07215400	1.48165800	1.53421100
C	5.09626600	1.39773200	-0.39119000
C	4.26046100	2.08458400	-1.29222400
C	3.76969800	3.34536700	-0.97364000
C	4.11011900	3.95034900	0.23695300
C	4.93865100	3.27717500	1.13697300
C	5.42411000	2.01085200	0.83393000
H	3.98274700	1.60795700	-2.23238400
H	3.10567900	3.85082400	-1.67705000
H	3.73029400	4.94483600	0.48004600
H	6.56408400	-1.85235900	-1.30790300

TS II’

E_{vacuum} = -26300.2728
E_{sol} = -26300.2882
$–TS$ = -0.1523
G_{sol} = -26299.5829

C	1.77509300	1.68568600	0.94504800
O	0.76209400	0.86455900	0.64462100
Sm	-1.03958100	-0.09321500	0.08613500
O	0.40757100	-2.08989400	0.43981300
C	1.42022400	-2.05938900	1.45816100
C	2.71995400	-1.89194700	0.69129700
C	2.43071600	-2.56781900	-0.66410300
C	0.94644000	-2.92666000	-0.59399600
I	-2.11779300	-0.16224900	2.97324300
I	-0.46548000	-0.26642400	-2.97936400
O	-3.51053000	0.25626600	-0.62260000
C	-4.65960500	-0.12697800	0.16005500

C	-5.85118600	0.50083100	-0.53927800
C	-5.40592700	0.47981900	-1.99756500
C	-3.93472600	0.82788300	-1.87634000
O	-1.62849200	2.29948200	-0.24549700
C	-2.60327000	3.04220800	0.51295200
C	-2.18481100	4.49773400	0.39599000
C	-1.49479400	4.52734900	-0.96511500
C	-0.76268100	3.19967800	-0.97339200
O	-2.32784800	-2.31893400	-0.20384100
C	-2.39195700	-3.34800400	0.80983200
C	-3.20380200	-4.47683100	0.19615400
C	-2.91522900	-4.30694100	-1.29190000
C	-2.91838700	-2.79787200	-1.42447800
H	-4.72090200	-1.22817900	0.16792900
H	-4.49888900	0.21164700	1.19204600
H	-3.77568500	1.91916700	-1.83469800
H	-3.29503300	0.41769600	-2.66901200
H	-5.99547500	1.53875000	-0.19761600
H	-6.78276400	-0.05044300	-0.35091500
H	-5.95072700	1.18677300	-2.63834500
H	-5.52838600	-0.52952600	-2.42299500
H	-3.59481700	2.86506200	0.06369600
H	-2.60995300	2.65436900	1.54170900
H	0.20653100	3.25046300	-0.45236100
H	-0.60992500	2.76932700	-1.97268100
H	-1.47058700	4.75363900	1.19251800
H	-3.04167900	5.18150400	0.47011300
H	-0.81152500	5.37850200	-1.09052000
H	-2.23973200	4.56314800	-1.77662300
H	-1.35981800	-3.65325600	1.03920700
H	-2.83205600	-2.90764200	1.71510000
H	-3.94899900	-2.40683800	-1.50213800
H	-2.32693700	-2.40374400	-2.26212300
H	-4.27869200	-4.33449200	0.39320400
H	-2.91431700	-5.45886500	0.59480800
H	-3.65527800	-4.78920900	-1.94535700
H	-1.91969800	-4.70771400	-1.54244600
H	1.17829900	-1.24057500	2.14255800
H	1.37633200	-3.01822500	2.00660500
H	0.79133600	-3.98003900	-0.29597200
H	0.39078200	-2.71643000	-1.51634000
H	3.56969700	-2.34262500	1.21858600
H	2.93405800	-0.82376100	0.55430400
H	3.04839400	-3.46194900	-0.82771900
H	2.61684800	-1.87386800	-1.49556500
C	1.79450400	2.22243700	2.36069000
H	2.70872600	2.82651600	2.46492700
C	0.59187800	3.12351100	2.63236600
H	0.60973400	3.50156100	3.66669400
H	0.58933600	3.99112700	1.95366800
H	-0.34635800	2.56367700	2.50005900
C	1.86593300	1.09030700	3.38368500
H	0.93873200	0.49719500	3.37075600
H	2.71941500	0.42460700	3.17947300
H	1.98365100	1.49506000	4.40113400
C	2.71203400	2.00438300	0.02202100
C	3.64273900	2.37941200	-2.12593200
H	3.63256500	2.44328600	-3.21698200
C	2.71476300	1.47177700	-1.38961300
H	1.70352900	1.44702800	-1.83496900
H	3.06031300	0.41932900	-1.43719500
C	3.83646900	3.00209200	0.16420800
H	4.77281600	2.50086000	0.47090000
H	3.63680500	3.79949100	0.89350400
C	4.01524600	3.54207400	-1.26243700
H	5.02534300	3.94033100	-1.45672200
H	3.31049100	4.38281300	-1.43648700
C	5.80515900	1.28521000	-2.10718700
C	5.91505600	0.35720500	-1.31193900
H	6.14202700	-4.10009800	-0.18695500
H	5.92277900	-2.26290300	-1.84370900
C	5.97818700	-0.69375000	-0.36147800
C	6.07070800	-0.41073900	1.01560000
C	6.19260400	-1.44155700	1.93896100
C	6.22334500	-2.77131900	1.51447800
C	6.12082100	-3.06251600	0.15296300
C	5.99690600	-2.03892300	-0.77868400
H	6.05081200	0.62869900	1.34548400
H	6.26564800	-1.20519800	3.00241000
H	6.32631800	-3.57799600	2.24272800
H	6.08764900	2.01901000	-2.83972300

Int III

E_{vacuum} = -26300.3332
E_{sol} = -26300.3467
$-TS$ = -0.1505
G_{sol} = -26299.6357

C	1.29174600	-2.61917000	0.76799200
O	0.38255300	-1.70283000	0.41740200
Sm	-0.95907700	-0.12078300	0.01385400
O	-1.69681900	-1.75944800	-1.75124700
C	-1.54805300	-3.16945700	-1.53581200
C	-0.37809900	-3.56972400	-2.42548800
C	-0.41065100	-2.52125300	-3.55861200
C	-1.55914600	-1.59372700	-3.16850200
I	-3.01665700	-1.12737800	2.08160500
I	0.65506900	1.28204200	-2.24760000
O	-1.68615200	2.26846800	0.67230400
C	-2.92199400	2.61405800	1.32935400
C	-2.73939300	4.04437600	1.80277900
C	-1.81800300	4.61541100	0.72994000
C	-0.88330800	3.44935700	0.47056100
O	0.50074200	0.90502900	1.74542900
C	0.05220100	1.27810300	3.06334200
C	1.25529400	1.08256600	3.96605800
C	2.41036600	1.40811400	3.02487600
C	1.94560900	0.78631600	1.72212700
O	-3.06847800	0.67449400	-1.26373000
C	-4.29630800	-0.08465800	-1.35349500
C	-5.20879600	0.71228300	-2.27329600
C	-4.21243300	1.47544100	-3.14001000
C	-3.14298100	1.82290100	-2.12550200
H	-3.74096500	2.52489100	0.59589600
H	-3.10097300	1.88230200	2.12833200
H	-0.04355100	3.42636600	1.18564600
H	-0.46904800	3.40547500	-0.54553900
H	-2.24142700	4.06390200	2.78575900
H	-3.69554000	4.57744500	1.89655300
H	-1.27941900	5.52006300	1.04416600
H	-2.39115500	4.85826500	-0.17957000
H	-0.26407300	2.33540600	3.03679500
H	-0.81526900	0.65320900	3.32027500
H	2.19828100	-0.28295700	1.65615600
H	2.31162100	1.30443000	0.82580100

H	1.31207900	0.03604200	4.30043900
H	1.21486900	1.72765500	4.85467400
H	3.37670600	1.00021200	3.34975300
H	2.52694900	2.49717700	2.90941500
H	-4.04306500	-1.07280900	-1.76593400
H	-4.68722600	-0.22535000	-0.33656800
H	-3.43355700	2.70561900	-1.52736600
H	-2.14243400	1.99212700	-2.54616200
H	-5.82428200	1.41828400	-1.69287400
H	-5.88772000	0.06448100	-2.84474800
H	-4.63799900	2.36418600	-3.62618700
H	-3.79443700	0.82088600	-3.92181400
H	-1.38764000	-3.32724800	-0.46636100
H	-2.48939500	-3.66103600	-1.84177900
H	-2.51130900	-1.89426100	-3.64373500
H	-1.35956700	-0.53359400	-3.36478800
H	-0.48572400	-4.59984000	-2.79268900
H	0.55949200	-3.50425100	-1.85564800
H	-0.57702100	-2.96635600	-4.54952700
H	0.52853800	-1.95155900	-3.59461700
C	0.92867500	-3.50572900	1.94080500
H	1.75842800	-4.21599400	2.07712100
C	0.79954300	-2.67282800	3.21616100
H	0.55062000	-3.31102700	4.07860600
H	1.74214700	-2.14848300	3.43982900
H	-0.00459200	-1.92876700	3.10867200
C	-0.34188800	-4.31592200	1.69733800
H	-1.21733100	-3.65403800	1.61077400
H	-0.25859000	-4.92492800	0.78358100
H	-0.53060000	-4.99804800	2.54103500
C	2.48080600	-2.69016300	0.12584400
C	4.32153400	-2.15194100	-1.35510900
H	4.25988300	-2.87486500	-2.18699800
C	2.90176600	-1.72430500	-0.95360000
H	2.90767800	-0.69023400	-0.56961700
H	2.21547800	-1.69794400	-1.81450500
C	3.61585400	-3.64733300	0.40495800
H	3.48647300	-4.58782400	-0.16439400
H	3.71634400	-3.92968100	1.46310900
C	4.84105400	-2.89723000	-0.11333200
H	5.70466900	-3.54537800	-0.32563700
H	5.15888300	-2.14731300	0.63123900
C	5.24645700	-1.02829800	-1.77917500
C	5.22597700	0.17597200	-1.27052700
H	5.81674000	2.83854400	2.41234800
H	6.25042800	0.76823300	1.12094500
C	4.98229600	1.33021600	-0.55324100
C	4.12933500	2.35440900	-1.06188300
C	3.89007400	3.50042000	-0.32349800
C	4.49163100	3.68781800	0.92759700
C	5.34348900	2.69846300	1.43777800
C	5.59031900	1.54031100	0.72227700
H	3.64772800	2.20580700	-2.02763700
H	3.21916600	4.26148400	-0.72773200
H	4.30401100	4.59883300	1.49889300
H	6.02107700	-1.28067100	-2.51806400

Int III'

E_{vacuum} = -26300.3313

E_{sol} = -26300.3457

$-TS$ = -0.1504

G_{sol} = -26299.6348

C	1.68907100	1.85963100	0.89948100
O	0.73849100	0.95628800	0.63706600
Sm	-1.01558000	-0.09795800	0.10064600
O	0.45360600	-2.02194700	0.67567800
C	1.45991000	-1.89581600	1.69330900
C	2.76399700	-1.79177800	0.92153400
C	2.48429400	-2.58530700	-0.37101600
C	1.00256000	-2.94488500	-0.27611800
I	-2.20942200	0.00937900	2.94070500
I	-0.29808700	-0.46717600	-2.91198100
O	-3.46402000	0.10935600	-0.73986400
C	-4.63521200	-0.25157300	0.02006500
C	-5.81327800	0.28002600	-0.77617600
C	-5.29866600	0.17616600	-2.20798900
C	-3.84794300	0.58648200	-2.04418300
O	-1.68417000	2.24614400	-0.39733200
C	-2.72160600	2.98999800	0.27128800
C	-2.36381300	4.45204900	0.07068500
C	-1.63534600	4.42363500	-1.27020900
C	-0.84430800	3.13374000	-1.16926700
O	-2.23199700	-2.38049900	-0.07304500
C	-2.33676900	-3.32660500	1.01553200
C	-3.04932200	-4.54059100	0.43914100
C	-2.68302900	-4.46179400	-1.03953800
C	-2.74484800	-2.96773200	-1.28048900
H	-4.66206600	-1.35033300	0.11086500
H	-4.53345300	0.17050000	1.02877600
H	-3.73017800	1.68380800	-2.06513600
H	-3.15710900	0.15199200	-2.77896800
H	-6.01077600	1.33267200	-0.51587400
H	-6.73283100	-0.29234800	-0.59167800
H	-5.83641700	0.81705000	-2.92022200
H	-5.36558300	-0.86365100	-2.56720800
H	-3.68540400	2.73762100	-0.20208600
H	-2.75226900	2.66959100	1.32244300
H	0.10841100	3.26600200	-0.63236300
H	-0.64846700	2.64260400	-2.13243100
H	-1.68562000	4.79088100	0.86784500
H	-3.25135400	5.09972100	0.07593800
H	-0.98746000	5.29557100	-1.43465800
H	-2.35697300	4.37127600	-2.10166000
H	-1.31474000	-3.55833000	1.35064400
H	-2.86840300	-2.83901800	1.84430400
H	-3.78682000	-2.63138600	-1.43139300
H	-2.13198300	-2.60495300	-2.11699300
H	-4.14006800	-4.44663000	0.56454800
H	-2.73361700	-5.47435100	0.92445000
H	-3.36290400	-5.02375900	-1.69470900
H	-1.65822100	-4.83055700	-1.20709100
H	1.21056700	-1.01894100	2.29824900
H	1.41604400	-2.80138400	2.32547600
H	0.85142200	-3.96835100	0.11381000
H	0.45197700	-2.82136800	-1.21687700
H	3.61542300	-2.18989300	1.48885000
H	2.97305600	-0.73855700	0.69137000
H	3.10808700	-3.48570900	-0.45321700
H	2.67108000	-1.96590100	-1.25931600
C	1.65552100	2.48054600	2.28003100
H	2.54722800	3.12053400	2.36728200
C	0.41767300	3.35625100	2.46527000
H	0.39624100	3.79967200	3.47331300
H	0.40354300	4.17842700	1.73217700

H	-0.49791500	2.75685800	2.34699100
C	1.73479900	1.41743400	3.37346800
H	0.82738300	0.79426800	3.37536400
H	2.61385300	0.76878100	3.23330300
H	1.81285900	1.88852200	4.36576600
C	2.61071600	2.19566800	-0.03200500
C	3.90058100	2.22665000	-2.07258100
H	3.76746500	2.33652400	-3.16092800
C	2.68132600	1.57805700	-1.40742500
H	1.77024500	1.79208900	-1.99094400
H	2.75170600	0.47923700	-1.38150000
C	3.65650300	3.28169600	0.08112500
H	4.56779800	2.91556500	0.58983900
H	3.31516400	4.16943800	0.63378100
C	3.98073900	3.59984800	-1.38105900
H	4.95161100	4.10021700	-1.51896200
H	3.20128400	4.25759400	-1.80139700
C	5.19802900	1.48346000	-1.84113500
C	5.38213500	0.43733500	-1.08246100
H	6.21817600	-4.03045800	-0.47663100
H	5.57042800	-2.10648500	-1.89795400
C	5.76214000	-0.63638200	-0.30932600
C	6.08911700	-0.46274600	1.07073100
C	6.44432000	-1.54719100	1.85165300
C	6.49044900	-2.83834500	1.30733000
C	6.17747400	-3.02767800	-0.04534100
C	5.81806400	-1.95826100	-0.84600000
H	6.04493800	0.54113700	1.49540900
H	6.68979700	-1.39269100	2.90460000
H	6.77263800	-3.68825800	1.93082600
H	6.07919900	1.92516100	-2.33840900

TS III

E_{vacuum} = -26300.3243

E_{sol} = -26300.3394

$-TS$ = -0.1481

G_{sol} = -26299.6272

C	-2.14866200	1.13900500	1.23597900
O	-1.03538500	0.58421400	0.78228500
Sm	0.84743700	-0.03401700	0.04503400
O	1.03600500	2.44645100	-0.00515000
C	0.65166200	3.25517800	1.12504000
C	0.90050200	4.69577800	-0.72432900
C	1.21613200	3.27479500	-1.17223000
I	2.13361600	-0.07213900	2.86311600
I	0.00877700	-0.11330700	-2.91738100
O	2.45303600	-1.97845600	-0.53810700
C	3.70233600	-2.26474100	0.11828300
C	4.02765900	-3.69853000	-0.25357700
C	3.45532800	-3.78776600	-1.66459900
C	2.17416000	-2.98396700	-1.53490700
O	-0.17213600	-2.23731900	0.43068800
C	0.22907900	-3.20931300	1.41753300
C	-1.02089800	-4.01631200	1.71222400
C	-1.74503000	-3.98381600	0.37046600
C	-1.48633300	-2.56414200	-0.09302800
O	3.15851700	0.77264800	-0.78353400
C	4.08650100	1.55468800	-0.00374700
C	5.38203600	1.54651800	-0.79392000
C	4.87336300	1.47635900	-2.23004900
C	3.70097800	0.52344900	-2.09562500
H	4.46792000	-1.56619900	-0.26250000
H	3.56964300	-2.08119600	1.19304300
H	1.33524100	-3.60185500	-1.17459100
H	1.85523100	-2.47266100	-2.45337000
H	3.50573300	-4.39783700	0.41955700
H	5.10406900	-3.91145100	-0.19712500
H	3.26893000	-4.81606500	-2.00354100
H	4.13905500	-3.31104500	-2.38580000
H	1.02491300	-3.83726900	0.98023600
H	0.64072000	-2.67061600	2.28237400
H	-2.20872500	-1.84818700	0.32219100
H	-1.45114600	-2.43523200	-1.18258100
H	-1.62460600	-3.51458100	2.48355300
H	-0.78517900	-5.03054500	2.06321200
H	-2.81951100	-4.19219100	0.44375600
H	-1.29591200	-4.70887100	-0.32845700
H	3.67695500	2.57347100	0.10114500
H	4.15241400	1.10489900	0.99615800
H	4.02649800	-0.52978900	-2.14473300
H	2.89599400	0.67004300	-2.82833200
H	5.97494300	0.64920300	-0.55290400
H	6.00276200	2.42874500	-0.58548400
H	5.62250200	1.11773200	-2.94928800
H	4.52768900	2.46788700	-2.56516000
H	-0.44503800	3.22436500	1.22772700
H	1.10773500	2.80526700	2.01735300
H	2.26258800	3.16499500	-1.49903500
H	0.56038400	2.90084800	-1.97115500
H	0.63400300	5.42791200	1.34024300
H	1.51662500	5.43985200	-1.24772400
H	-0.15620200	4.93331000	-0.92066800
C	-2.52598900	0.82198700	2.66548200
H	-3.58731800	1.09117600	2.79292900
C	-1.69601400	1.68132400	3.62518200
H	-1.98108100	1.48719400	4.67165000
H	-0.62553600	1.44516600	3.51823000
H	-1.83864500	2.75558100	3.42809200
C	-2.35736600	-0.65750800	2.99658500
H	-1.30791500	-0.95972900	2.86411200
H	-2.63594000	-0.85086800	4.04429800
H	-2.99084300	-1.28557000	2.35256700
C	-2.91773400	1.95842900	0.43088700
C	-2.44493100	2.57488000	-0.86416000
C	-4.05779800	2.83701400	0.94567000
C	-4.24314200	0.69358100	-0.87711300
C	-3.78839500	2.98482700	-1.46491200
H	-1.83292100	3.47353900	-0.65889000
H	-1.87103900	1.88554200	-1.49561200
H	-4.90601000	2.24192100	1.31363000
H	-3.72872700	3.46977800	1.78717300
C	-4.53210500	1.69400700	-1.69309700
H	-3.72273500	3.61083100	-2.36612600
H	-5.35326200	-0.35230000	1.34896800
H	-5.34398200	1.64375900	-2.43315600
H	2.23389700	4.72132300	0.99719800
C	1.15635800	4.64052900	0.77981500
C	-4.61243500	-0.66268800	-0.65951500
C	-4.37533000	-1.63796000	-1.65605800
C	-4.73513500	-2.96394500	-1.45206500
C	-5.31416700	-3.36641600	-0.24497400
C	-5.52355000	-2.42022600	0.76161700
C	-5.17733200	-1.08912700	0.56297600
H	-3.90734900	-1.32426900	-2.59095900

H	-4.56116100	-3.69621800	-2.24428700
H	-5.59885000	-4.40906600	-0.08940500
H	-5.97076700	-2.72413300	1.71096000
C	-4.45164200	3.70496000	-0.27162800
H	-5.54046000	3.80401300	-0.39049800
H	-4.03643100	4.72168600	-0.17368400

TS III'

E_{vacuum} = -26300.3210
E_{sol} = -26300.3359
$-TS$ = -0.1481
G_{sol} = -26299.6235

C	2.08996500	1.46341200	0.78744400
O	1.01304000	0.75691600	0.47078600
Sm	-0.89973500	-0.05474700	0.02102600
O	0.51320300	-2.04324900	-0.24138400
C	1.62340000	-2.28254400	0.65868800
C	1.94519600	-3.63947100	-1.22959800
C	0.63202300	-2.89406900	-1.40285000
I	-1.58804900	-0.65181700	2.97463900
I	-0.78284900	0.38630800	-3.03374100
O	-3.43092000	0.45319000	-0.25497600
C	-4.48347500	-0.06836900	0.58051000
C	-5.69473800	0.79426900	0.28337500
C	-5.48791500	1.11859400	-1.19211000
C	-3.98637700	1.32816500	-1.25926000
O	-1.43987700	2.34755400	0.35751800
C	-2.27201600	2.90992400	1.39323300
C	-1.83007200	4.35678200	1.52450500
C	-1.35882600	4.67817100	0.10910600
C	-0.66889300	3.38756000	-0.28462500
O	-2.30492000	-2.16529500	-0.47219700
C	-2.38315200	-3.31628000	0.39592700
C	-3.44232000	-4.21412400	-0.21787700
C	-3.30538100	-3.89957400	-1.70389000
C	-3.05370500	-2.40431700	-1.67995100
H	-4.66021800	-1.12227300	0.30458200
H	-4.13675800	-0.03881500	1.62191100
H	-3.70477900	2.36459300	-1.00787000
H	-3.52787500	1.07199400	-2.22357500
H	-5.66914700	1.71537600	0.88817300
H	-6.63940200	0.27498900	0.49596200
H	-6.04712800	1.99950700	-1.53618500
H	-5.78541200	0.26153800	-1.81802700
H	-3.32272900	2.83181900	1.06720300
H	-2.14091600	2.30895800	2.30398800
H	0.36372500	3.33685100	0.09451600
H	-0.66641600	3.17572200	-1.36265600
H	-0.99159900	4.43618000	2.23278200
H	-2.64203800	5.00707900	1.87791300
H	-0.68234000	5.54224300	0.05773600
H	-2.21877600	4.87556800	-0.55149000
H	-1.39122000	-3.79774000	0.41618100
H	-2.61378700	-2.96236600	1.40960000
H	-3.99881700	-1.83659000	-1.62532400
H	-2.46731400	-2.01834700	-2.52459700
H	-4.44408600	-3.92920700	0.14269100
H	-3.28295600	-5.27303500	0.02771200
H	-4.19213000	-4.16412400	-2.29626400
H	-2.43934400	-4.42903800	-2.13301900
H	2.38781000	-1.51113800	0.48379800
H	1.23857000	-2.19069600	1.68261500
H	-0.23158700	-3.57856900	-1.40579600
H	0.58633600	-2.26154800	-2.30046700
H	3.15703400	-3.82045300	0.60103000
H	1.91622200	-4.63782200	-1.68783900
H	2.76990000	-3.07275200	-1.68610800
C	2.37567000	1.63968300	2.26195500
H	3.37999200	2.08527000	2.34700700
C	2.36415900	0.31953400	3.02824400
H	2.62205200	0.49095100	4.08529300
H	1.36355300	-0.13531400	2.99985400
H	3.08775100	-0.39509100	2.61120400
C	1.36948900	2.61686600	2.87639500
H	0.34911000	2.21413200	2.78108000
H	1.57111900	2.76682700	3.94882000
H	1.41094000	3.60028100	2.38119300
C	2.90311700	2.01138700	-0.18980600
C	3.92873500	3.11509400	0.08190800
C	2.52803500	2.11262300	-1.64857900
C	4.47217700	0.53999400	-0.89240500
C	3.89095000	2.44457800	-2.25194500
H	3.47766000	3.94803600	0.64744000
H	4.78179200	2.75720700	0.67594800
H	2.07334000	1.20083600	-2.05179900
H	1.82296000	2.94758100	-1.81537500
C	4.76157700	1.24749200	-1.97487900
H	3.87034000	2.73110300	-3.31278400
H	4.87716700	-1.86913700	-1.97743500
H	5.66416000	1.06756500	-2.57803700
H	1.48277200	-4.43940100	0.74272300
C	2.11739300	-3.66219600	0.28570000
C	4.99018000	-0.61185800	-0.22652200
C	5.33545100	-0.58313800	1.14148000
C	5.81218800	-1.72035500	1.78162900
C	5.96052300	-2.92070200	1.08243600
C	5.62550500	-2.96672400	-0.27271900
C	5.13841700	-1.83536000	-0.91765600
H	5.22861800	0.35320000	1.69164100
H	6.07488300	-1.67095700	2.84075600
H	6.33720900	-3.81125500	1.58899400
H	5.74596900	-3.89684800	-0.83384300
C	4.36716500	3.58273200	-1.32414600
H	3.85636100	4.52049100	-1.59802900
H	5.44973600	3.76333200	-1.39527300

Int IV

E_{vacuum} = -26300.3615
E_{sol} = -26300.3762
$-TS$ = -0.1472
G_{sol} = -26299.6606

C	-2.30351000	0.83706700	1.30836900
O	-1.10801600	0.47821500	0.83402700
Sm	0.78280600	-0.00713400	0.06446500
O	0.91932100	2.47690000	-0.04422800
C	0.60977600	3.30628200	1.09630200
C	0.54887400	4.66371800	-0.83896600
C	1.03352700	3.28015700	-1.23520400
I	2.21930500	0.10685200	2.81157800
I	-0.21411600	-0.16326900	-2.84763200
O	2.47701500	-1.87293600	-0.59374400
C	3.76826900	-2.08427000	0.00441800
C	4.15009200	-3.50622200	-0.36169400
C	3.50669400	-3.65327600	-1.73715200

C	2.19381200	-2.91493200	-1.54792800
O	-0.01524600	-2.28255400	0.58064400
C	0.51549500	-3.19394700	1.56065700
C	-0.65966200	-4.05997000	1.97102500
C	-1.46206800	-4.13516400	0.67578800
C	-1.32316200	-2.72489000	0.13674800
O	3.03945900	0.87450900	-0.91929800
C	3.99293900	1.68064900	-0.19965000
C	5.23311200	1.71877900	-1.07485600
C	4.63439300	1.62254600	-2.47438600
C	3.50863200	0.62925700	-2.25806500
H	4.47793300	-1.35481300	-0.42359900
H	3.67915100	-1.88887300	1.08191500
H	1.41300100	-3.56850500	-1.12461600
H	1.79331800	-2.44308000	-2.45576400
H	3.70477800	-4.21992100	0.35024500
H	5.23793000	-3.66066700	-0.36197400
H	3.35757200	-4.69654800	-2.04772600
H	4.12299100	-3.15524400	-2.50335700
H	1.31257200	-3.79263800	1.08501000
H	0.95579200	-2.60054800	2.37380300
H	-2.07319600	-2.04099900	0.55586100
H	-1.34905300	-2.64675800	-0.95807900
H	-1.24509300	-3.55614100	2.75522900
H	-0.34053500	-5.03994500	2.35217400
H	-2.51498000	-4.40699400	0.82243800
H	-1.01045500	-4.86510300	-0.01631200
H	3.56003800	2.68554900	-0.05851200
H	4.14212000	1.23083200	0.79134200
H	3.87078100	-0.41170600	-2.31854300
H	2.65532400	0.73584700	-2.94143500
H	5.87495200	0.84581600	-0.87261400
H	5.83255500	2.62456000	-0.90934500
H	5.34749700	1.28663200	-3.23983300
H	4.23171700	2.59896900	-2.78962500
H	-0.45225500	3.16546900	1.34527000
H	1.22125400	2.95287200	1.93797400
H	2.09303900	3.29134600	-1.54111500
H	0.44439100	2.80194900	-2.03041300
H	0.34125400	5.46971500	1.20611800
H	1.02050600	5.45437200	-1.43871900
H	-0.54108500	4.73808500	-0.96887300
C	-2.52826200	0.70707800	2.78969300
H	-3.60590800	0.85722200	2.97649400
C	-1.75907400	1.77515800	3.58137100
H	-1.95043600	1.68569800	4.66348000
H	-0.67632500	1.65417400	3.42089500
H	-2.04702200	2.79181200	3.26904000
C	-2.14331400	-0.68391800	3.29699400
H	-1.07583800	-0.87260900	3.10592300
H	-2.31209100	-0.76862900	4.38212900
H	-2.73031300	-1.46711300	2.79311900
C	-3.22177700	1.53471100	0.35918700
C	-2.58538300	2.64134400	-0.51770600
C	-4.41194700	2.34041700	0.97024900
C	-3.85954200	0.68527500	-0.76148300
C	-3.85362000	2.92569400	-1.32094900
H	-2.25413400	3.50046400	0.08806400
H	-1.76233000	2.25123500	-1.12989200
H	-5.22706300	1.68388400	1.30577500
H	-4.06765900	2.91244300	1.84531800
C	-4.21340000	1.52618300	-1.75084400
H	-3.79057000	3.67518300	-2.12063800
H	-4.72241700	-0.66319700	1.42706700
H	-4.80773900	1.27302000	-2.63050500
H	1.98584800	4.94468300	0.76837700
C	0.91403000	4.72083700	0.64206300
C	-4.18290100	-0.73651000	-0.66421100
C	-4.07026600	-1.57158500	-1.78584700
C	-4.41754200	-2.91741600	-1.70876500
C	-4.87985800	-3.45832000	-0.50851900
C	-4.98278500	-2.64149500	0.61758600
C	-4.62991000	-1.29611400	0.54371800
H	-3.67500000	-1.15419900	-2.71373000
H	-4.31869600	-3.55189500	-2.59233600
H	-5.15566800	-4.51349600	-0.45025000
H	-5.34393900	-3.05382000	1.56253700
C	-4.84237000	3.28804900	-0.17600100
H	-5.88927300	3.14633700	-0.48017500
H	-4.72227500	4.34359700	0.11559400

Int IV'

E_{vacuum} = -26300.3595
E_{sol} = -26300.3743
$-TS$ = -0.1471
G_{sol} = -26299.6581

C	-2.29953900	-1.28258600	0.96486700
O	-1.13021800	-0.76242700	0.57548200
Sm	0.77012900	-0.02111400	0.03879600
O	-0.42308700	2.11204200	0.20726000
C	-1.27948200	2.44830500	1.31085600
C	-1.29326000	4.25039300	-0.28424500
C	-0.85823300	2.91586900	-0.89858900
I	1.84601100	0.45250600	2.90761200
I	0.26328400	-0.25597500	-2.99550900
O	3.24986000	-0.61720000	-0.55579800
C	4.41815500	-0.18050800	0.16435500
C	5.55090500	-1.05930000	-0.33378500
C	5.13375700	-1.31714200	-1.77807200
C	3.63189700	-1.48374100	-1.64122100
O	1.30897500	-2.43569900	0.29233600
C	2.24208000	-3.04858500	1.20511200
C	1.78900200	-4.49223300	1.33116600
C	1.17482000	-4.75422400	-0.04106100
C	0.46988400	-3.44057200	-0.31499200
O	2.23674200	2.04605400	-0.63058000
C	2.46598000	3.18171200	0.22882500
C	3.58420800	3.96433300	-0.43192100
C	3.30352000	3.71008200	-1.90900400
C	2.89396300	2.24901200	-1.89672900
H	4.59562300	0.88281500	-0.07110500
H	4.20598500	-0.26173100	1.23884600
H	3.36012300	-2.51943900	-1.37356400
H	3.05112900	-1.19256800	-2.52699700
H	5.58284500	-2.00511600	0.23131500
H	6.53112900	-0.57285300	-0.23478400
H	5.61266000	-2.19770600	-2.22805100
H	5.36840000	-0.44349800	-2.40785100
H	3.25097700	-2.97512200	0.76518300
H	2.22528600	-2.47877500	2.14447100
H	-0.51744900	-3.39476800	0.16546200
H	0.36206900	-3.18672900	-1.37875700
H	1.02226700	-4.58490700	2.11562600
H	2.61951800	-5.16715000	1.57983500

H	0.48265900	-5.60725400	-0.05714700
H	1.96203400	-4.93711000	-0.79061700
H	1.53644100	3.77104600	0.28326800
H	2.69653400	2.80264900	1.23379200
H	3.77207200	1.58284500	-1.94490700
H	2.19723000	1.95598400	-2.69369600
H	4.56605800	3.55009000	-0.14987700
H	3.57088000	5.02712100	-0.15339400
H	4.16479200	3.89710100	-2.56515900
H	2.46841700	4.34139000	-2.25400100
H	-2.24956200	1.94814300	1.17427200
H	-0.79470300	2.07596100	2.22166300
H	-0.01752400	2.98161300	-1.60004100
H	-1.69514700	2.40258000	-1.39691400
H	-2.34029500	4.31378900	1.66023000
H	-0.56491500	5.04802000	-0.49015000
H	-2.25856000	4.56403200	-0.70364000
C	-2.43727500	-1.67375600	2.41265400
H	-3.48374800	-1.98415700	2.57202400
C	-2.13568300	-0.51447200	3.36462600
H	-2.27884500	-0.82318500	4.41248000
H	-1.09170200	-0.18586700	3.25466200
H	-2.78804600	0.34920300	3.16878900
C	-1.53593900	-2.86650200	2.76329200
H	-0.48347400	-2.59776900	2.58128500
H	-1.63318500	-3.14633300	3.82500400
H	-1.78151900	-3.75103800	2.15363500
C	-3.27788100	-1.65163200	-0.10627200
C	-4.45019600	-2.60459300	0.29611900
C	-2.71650400	-2.44166900	-1.31614300
C	-3.96897200	-0.50114900	-0.87082500
C	-4.03105100	-2.46015500	-2.09258000
H	-4.06711600	-3.42210900	0.92576400
H	-5.23047900	-2.08455000	0.87010600
H	-1.91310000	-1.90038200	-1.83148700
H	-2.37975900	-3.44978300	-1.02538300
C	-4.39130300	-0.99894700	-2.04898600
H	-4.02626700	-2.92812700	-3.08517000
H	-4.12119700	1.83794300	-2.20133700
H	-5.04609500	-0.49526500	-2.76280600
H	-0.56812800	4.43631400	1.77237600
C	-1.39580800	3.96075400	1.22534000
C	-4.30577600	0.80881000	-0.31731500
C	-4.60939600	0.96362600	1.04473600
C	-4.97605400	2.20541900	1.55912500
C	-5.04214400	3.32186100	0.72649600
C	-4.74148700	3.18227600	-0.62938700
C	-4.37255700	1.94220100	-1.14350900
H	-4.57243000	0.09519400	1.70216100
H	-5.21484200	2.29943500	2.62078000
H	-5.33064300	4.29464600	1.12993800
H	-4.79561900	4.04840400	-1.29316300
C	-4.96612600	-3.15000600	-1.05749700
H	-4.85589900	-4.24457900	-1.11255000
H	-6.02470800	-2.91496300	-1.23950500

Product

E_{vacuum} = -26300.3750

E_{sol} = -26300.3961

$-TS$ = -0.1511

G_{sol} = -26299.6832

C	2.46848000	-0.82112400	1.30526000
O	1.42953600	-0.34736200	0.86231900
Sm	-0.87546900	0.04643000	-0.00225800
O	-0.64557100	-2.55465100	-0.15789300
C	-0.59186100	-3.48488500	0.93521100
C	-0.50598700	-4.71372300	-1.08047400
C	-0.78961700	-3.25734500	-1.40340800
I	-2.04629600	-0.37955600	2.98005500
I	0.19969400	0.33973500	-3.00704000
O	-2.68944100	1.86667200	-0.44135000
C	-3.94655900	1.98613900	0.23906900
C	-4.40718600	3.41006800	-0.01866000
C	-3.84269200	3.66790700	-1.41340100
C	-2.49288400	2.97611300	-1.33188700
O	-0.01994300	2.38742500	0.65219200
C	-0.52526300	3.16345200	1.74754600
C	0.60120300	4.11107800	2.11741700
C	1.24619300	4.36680700	0.75819600
C	1.17300400	2.99183400	0.11797500
O	-3.08045100	-1.01190000	-0.95470800
C	-3.96314100	-1.88372200	-0.23364800
C	-5.27725100	-1.84268800	-0.99349800
C	-4.79589500	-1.64944000	-2.42908100
C	-3.63684700	-0.68642000	-2.23824100
H	-4.65238700	1.25260600	-0.19091800
H	-3.78945100	1.73441600	1.29800400
H	-1.72143600	3.63687800	-0.90035100
H	-2.11446800	2.58424600	-2.28727000
H	-3.95270600	4.09848700	0.71241700
H	-5.49882300	3.51811700	0.04531300
H	-3.75438800	4.73367100	-1.66585700
H	-4.47654400	3.18827600	-2.17719100
H	-1.41922100	3.71785700	1.40659400
H	-0.82962300	2.47294300	2.54754100
H	2.03625500	2.36828300	0.39606600
H	1.09128000	2.99854300	-0.97786400
H	1.31408700	3.60912700	2.79093800
H	0.23939700	5.02101700	2.61619000
H	2.27815200	4.73680800	0.81939200
H	0.65178700	5.09671000	0.18401900
H	-3.53329200	-2.90192000	-0.23494900
H	-4.01615600	-1.53372800	0.80749000
H	-3.98019700	0.36247300	-2.21406900
H	-2.83689000	-0.76657600	-2.98831700
H	-5.88074600	-0.97833800	-0.67125300
H	-5.87828600	-2.75051700	-0.84493800
H	-5.56542000	-1.25254400	-3.10565000
H	-4.43802200	-2.60583900	-2.84441300
H	0.44596000	-3.52799200	1.30910200
H	-1.23529600	-3.10228400	1.74077500
H	-1.82150300	-3.11390100	-1.76623500
H	-0.10730100	-2.80325800	-2.13629700
H	-0.61163900	-5.67319100	0.90926300
H	-0.99932200	-5.40196300	-1.78076800
H	0.57752900	-4.91133800	-1.11334000
C	2.75835600	-0.74652700	2.78575300
H	3.84864500	-0.82589900	2.92895300
C	2.08905000	-1.96092700	3.44754500
H	2.33150500	-1.98152000	4.52038500
H	0.99527100	-1.88610700	3.34387000
H	2.42328500	-2.91328500	3.00696500
C	2.23894400	0.55415700	3.38544600
H	1.14840300	0.62292900	3.25987200

H	2.46554500	0.59032400	4.46120700
H	2.70294800	1.42768000	2.90287400
C	3.40343700	-1.51329900	0.34557800
C	2.72388200	-2.60842900	-0.50745800
C	4.64427900	-2.28560300	0.87851700
C	3.91993200	-0.60653700	-0.78185300
C	3.95364200	-2.83731200	-1.38851300
H	2.44022400	-3.48888900	0.09163600
H	1.86067800	-2.21102800	-1.05407100
H	5.45912700	-1.60627900	1.16497000
H	4.38343000	-2.88425200	1.76394000
C	4.24034100	-1.41758600	-1.80624600
H	3.85769900	-3.57015100	-2.19920500
H	4.80088900	0.77284700	1.39548400
H	4.75021100	-1.11978300	-2.72293400
H	-2.12085500	-4.90607900	0.35917900
C	-1.02205600	-4.81958000	0.35208000
C	4.15506400	0.83320500	-0.67004200
C	3.93059100	1.66997600	-1.77329400
C	4.19025300	3.03430900	-1.68860500
C	4.66799000	3.59104200	-0.50192700
C	4.87562600	2.77186100	0.60729800
C	4.61680000	1.40600900	0.52488800
H	3.50606200	1.24279600	-2.68334200
H	4.00080300	3.67167600	-2.55472400
H	4.87035100	4.66223200	-0.43902300
H	5.24591600	3.19701800	1.54256400
C	5.02020700	-3.19935900	-0.31537800
H	6.04216700	-3.02514600	-0.67952100
H	4.94210500	-4.26247200	-0.03976000

Product’

E_{vacuum} = -26300.3733
E_{sol} = -26300.3953
$-TS$ = -0.1515
G_{sol} = -26299.6824

C	-2.46573000	-1.22233600	0.99031700
O	-1.47797500	-0.59146100	0.63629000
Sm	0.92459600	0.01915900	0.06185800
O	-0.46537000	2.17896700	0.03822900
C	-1.25645800	2.70089800	1.10606300
C	-1.30604500	4.22365000	-0.76341700
C	-0.96090200	2.78311600	-1.15498400
I	1.81936400	0.69737900	3.06245400
I	0.37922700	-0.62978800	-3.06054500
O	3.39368000	-0.80787400	-0.30739500
C	4.53139500	-0.19003300	0.32325000
C	5.75383700	-0.85513800	-0.29122300
C	5.24426300	-1.24726400	-1.67543700
C	3.82606100	-1.68117600	-1.35943000
O	1.03106800	-2.53021100	0.63213200
C	1.97742900	-3.04128700	1.59140400
C	1.89234000	-4.55364700	1.47601200
C	1.53938800	-4.73811300	0.00261700
C	0.57441100	-3.58900700	-0.22479800
O	2.42467000	2.02907900	-0.73202200
C	2.56241000	3.24894800	0.02255900
C	3.32959400	4.20967000	-0.87618200
C	2.98728100	3.69379600	-2.27139900
C	2.98653700	2.19572800	-2.03938700
H	4.49163400	0.88888300	0.10416200
H	4.43677700	-0.31807100	1.41090500
H	3.80638600	-2.72441400	-0.99138800
H	3.11486700	-1.58440800	-2.19288400
H	6.03296100	-1.75591400	0.27915000
H	6.62544500	-0.18626200	-0.31288000
H	5.83303100	-2.04102000	-2.15590300
H	5.23240400	-0.37405400	-2.34763600
H	2.97547600	-2.66041900	1.32115600
H	1.71660400	-2.63819600	2.58003700
H	-0.44979200	-3.86785400	0.07928800
H	0.54509900	-3.20599200	-1.25594900
H	1.08315700	-4.94525100	2.11368000
H	2.82784800	-5.04799200	1.77230900
H	1.09092400	-5.71479600	-0.22636400
H	2.43495000	-4.61755700	-0.62774600
H	1.54860700	3.61021600	0.25176800
H	3.06394000	3.01762600	0.97357000
H	4.01803200	1.79473300	-2.04712100
H	2.37591900	1.61294700	-2.74419500
H	4.41395900	4.12640800	-0.69877000
H	3.04076100	5.25655600	-0.70803800
H	3.70209500	4.00450900	-3.04600300
H	1.98253200	4.03061900	-2.57556300
H	-2.23727900	2.19665200	1.11122700
H	-0.72360200	2.48170700	2.04143700
H	-0.18238200	2.68396200	-1.92241100
H	-1.85184200	2.22873700	-1.49369200
H	-2.34412800	4.59042500	1.15568400
H	-0.52687900	4.92548700	-1.09509500
H	-2.25209100	4.53695100	-1.22648500
C	-2.63041400	-1.62610100	2.43919800
H	-3.70011800	-1.81651200	2.62625400
C	-2.11427100	-0.55237700	3.38786500
H	-2.25739900	-0.87307100	4.43039600
H	-1.04169400	-0.36004800	3.23353300
H	-2.64807000	0.39921900	3.24584600
C	-1.86583900	-2.94436600	2.62885100
H	-0.80197000	-2.78732600	2.39879500
H	-1.95523900	-3.28224200	3.67178100
H	-2.24741100	-3.74589900	1.97728900
C	-3.46664500	-1.64511600	-0.05877900
C	-4.64027700	-2.59448000	0.32500400
C	-2.83273100	-2.42914100	-1.23145600
C	-4.11307300	-0.49218800	-0.84064900
C	-4.12439300	-2.46539400	-2.04922700
H	-4.29132400	-3.40174100	0.98572300
H	-5.44586500	-2.06103900	0.84914500
H	-2.01798700	-1.87279100	-1.71520900
H	-2.48734300	-3.42907700	-0.92358500
C	-4.49471100	-1.00625000	-2.02604800
H	-4.07500700	-2.93778200	-3.03755400
H	-4.33209900	1.81401200	-2.21505200
H	-5.11396500	-0.50561400	-2.77164900
H	-0.57646200	4.75560500	1.23240100
C	-1.39725000	4.18259300	0.77623100
C	-4.43022800	0.82889100	-0.29892100
C	-4.66145600	1.02061100	1.07112600
C	-4.99093400	2.27665400	1.57480500
C	-5.09376100	3.37100300	0.71841600
C	-4.86524100	3.19526900	-0.64710900
C	-4.53342400	1.94146800	-1.14950600
H	-4.60347200	0.17205900	1.75483000
H	-5.16922600	2.39864300	2.64503300

H	-5.34947000	4.35648300	1.11207000
H	-4.94035200	4.04637800	-1.32729300
C	-5.08928900	-3.16107200	-1.04538200
H	-4.95803400	-4.25337800	-1.08498900
H	-6.14304500	-2.94517600	-1.27060200
